# The complex evolutionary history of the tympanic middle ear in frogs and toads (Anura)

**DOI:** 10.1038/srep34130

**Published:** 2016-09-28

**Authors:** Martín O. Pereyra, Molly C. Womack, J. Sebastián Barrionuevo, Boris L. Blotto, Diego Baldo, Mariane Targino, Jhon Jairo Ospina-Sarria, Juan M. Guayasamin, Luis A. Coloma, Kim L. Hoke, Taran Grant, Julián Faivovich

**Affiliations:** 1 Museo Argentino de Ciencias Naturales “Bernardino Rivadavia”-CONICET, Buenos Aires, C1405DJR, Argentina; 2 Department of Biology, Colorado State University, Fort Collins, CO 80523, USA; 3 Departamento de Zoologia, Instituto de Biociências, Universidade de São Paulo, São Paulo, SP 05508-090, Brazil; 4 Laboratorio de Genética Evolutiva, Instituto de Biología Subtropical (CONICET-UNaM), Facultad de Ciencias Exactas Químicas y Naturales, Universidad Nacional de Misiones, Posadas, N3300LQF, Argentina; 5 Centro de Investigación de la Biodiversidad y Cambio Climático (BioCamb), Ingeniería en Biodiversidad y Cambio Climático, Facultad de Medio Ambiente, Universidad Tecnológica Indoamérica, Diego de Robles y Vía Interoceánica, 17-1200-841, Quito, EC170103, Ecuador; 6 Colegio de Ciencias Biológicas y Ambientales COCIBA, Laboratorio de Biología Evolutiva, Universidad San Francisco de Quito, Campus Cumbayá, Quito, 170901, Ecuador; 7 Centro Jambatu de Investigación y Conservación de Anfibios, Fundación Otonga, Geovanni Farina 566 y Baltra, San Rafael, Quito, Ecuador; 8 Universidad Regional Amazónica Ikiam, Muyuna, Tena, Ecuador; 9 Departamento de Biodiversidad y Biología Experimental, Facultad de Ciencias Exactas y Naturales, Universidad de Buenos Aires, Buenos Aires, C1428EGA, Argentina

## Abstract

Most anurans possess a tympanic middle ear (TME) that transmits sound waves to the inner ear; however, numerous species lack some or all TME components. To understand the evolution of these structures, we undertook a comprehensive assessment of their occurrence across anurans and performed ancestral character state reconstructions. Our analysis indicates that the TME was completely lost at least 38 independent times in Anura. The inferred evolutionary history of the TME is exceptionally complex in true toads (Bufonidae), where it was lost in the most recent common ancestor, preceding a radiation of >150 earless species. Following that initial loss, independent regains of some or all TME structures were inferred within two minor clades and in a radiation of >400 species. The reappearance of the TME in the latter clade was followed by at least 10 losses of the entire TME. The many losses and gains of the TME in anurans is unparalleled among tetrapods. Our results show that anurans, and especially bufonid toads, are an excellent model to study the behavioural correlates of earlessness, extratympanic sound pathways, and the genetic and developmental mechanisms that underlie the morphogenesis of TME structures.

The function of audition in frogs and toads (Anura) is primarily the perception of airborne sounds, including those involved in social communication^1^. Thus, hearing in anurans is thought to be a key trait for survival and reproduction. In most anurans, perception of airborne sounds is enabled by a tympanic middle ear (TME) composed minimally of a tympanic membrane, middle ear cavity, and middle ear bone (=columella, columella auris, stapes, plectrum) that conducts sound waves from the environment to the inner ear where they are transduced into electrical signals via hair cells^1^,^2^,^3^,^4^.

Among other tetrapods, a TME is absent in caecilians and salamanders^1^,^5^ but present in amniotes. Nevertheless, although the TME is primitively present in all extant amniotes, it is not homologous across amniote lineages, having evolved independently at least five times in turtles, lepidosaurs, archosaurs, an extinct lineage of parareptiles, and the synapsid ancestor of mammals^6^,^7^,^8^,^9^,^10^. TME losses are extremely rare in amniotes. All mammals, turtles, and archosaurs possess a complete TME, and even the amphisbaenians, snakes, and lizards that have lost the tympanic membrane and middle ear cavity retain a columella^11^, the sole exceptions being the pygopod lizard *Aprasia repens*^12^ and possibly the snakes *Atractaspis* and *Xenocalamus*^13^. In contrast, loss is widespread among anurans, with at least a few species of several families lacking the entire TME, a condition referred to as “earlessness”^14^.

Earlessness is especially common in the true toad family Bufonidae, in which the TME is completely lacking in more than 200 species. Bufonidae is one of the most diversified groups of amphibians, comprising more than 580 species in 51 genera naturally distributed over numerous ecoregions of the Americas, Africa, and Eurasia^15^. Several authors have noted the reduction and loss of TME structures (e.g.,^16^,^17^,^18^,^19^,^20^) or morphological variations in middle ear structure (e.g.,^1^,^21^,^22^,^23^) in bufonids, but the phylogenetic distribution of earlessness has never been studied either within Bufonidae or across Anura.

As such, the goals of this study are to explore the sequences of gains and losses of the TME evolution across all anurans and evaluate in a phylogenetic framework the patterns of diversification of TME in Bufonidae.

## Materials and Methods

### Tympanic middle ear morphology and data collection

Although many variations in the structure of the anuran auditory system exist, a generalized model can be described^1^,^2^,^3^: the lateral-most portion of this system is composed of a highly differentiated disc of thin, non-glandular skin, termed the tympanic membrane. The rim of the tympanic membrane is attached to a cartilaginous tympanic ring, the tympanic annulus. The middle ear cavity is a diverticulum of the pharynx that opens ventrally to the buccal cavity via the Eustachian tubes. The columella contacts the tympanic membrane laterally and the otic capsule medially and is divided into three portions^1^,^24^,^25^: (1) the pars externa plectri or extracolumella, a cartilaginous structure that contacts the tympanic membrane and often presents a slim, flattened strip of cartilage called the ascending process or pars ascendens plectri, that extends anterodorsally to contact the crista parotica of the prootic, (2) the pars media plectri or columellar shaft, an ossified, rod-shaped portion with a dilated medial end, and (3) the pars interna plectri, a mainly cartilaginous structure that is continuous with the pars media and extends posteriorly to lie medial to the operculum. The expanded medial end of the pars media and the entire pars interna constitute the stapedial footplate, which fills the rostral portion of the oval window of the otic capsule^1^,^26^. The footplate is connected to the suprascapula via the columellar muscle in some species^1^, although the individuality of this muscle has been questioned in at least some cases^27^,^28^. The otic operculum (operculum fenestrae ovalis), found only in caudates and anurans^5^, is an ovoid element that is usually cartilaginous or sometimes partially calcified^4^,^25^, that contacts the stapedial footplate and covers the caudal portion of the oval window. The operculum is present in all anurans. The opercularis muscle inserts on the operculum and originates on the suprascapular cartilage of the pectoral girdle^1^,^26^.

**Figure 1:**
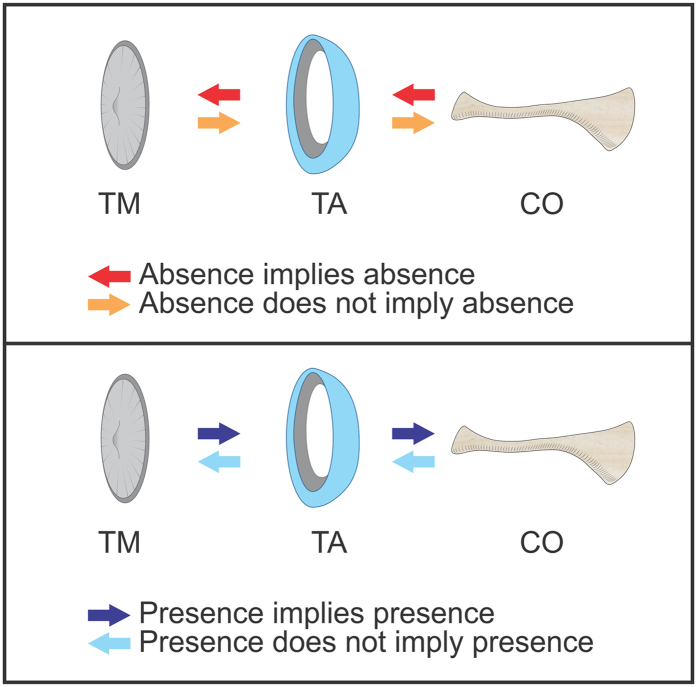
Schematic representation of tympanic middle ear structures in anurans, showing the assumptions followed here for coding absence and presence of the different elements. TM, tympanic membrane; TA, tympanic annulus; CO, columella

In all observed anuran species (also see^29^), the presence/absence of TME structures follows a consistent pattern. Absence of a medial structure is accompanied by the absence of the more lateral structures, such that absence of the columella entails absence of the tympanic annulus and tympanic membrane and absence of the tympanic annulus entails absence of the tympanic membrane but not the columella. Similarly, presence of a lateral structure is accompanied by the presence of the more medial structures, such that presence of the tympanic membrane entails presence of the tympanic annulus and columella and presence of the tympanic annulus entails presence of the columella but not the tympanic membrane. Consequently, we made the following assumptions when scoring the presence/absence of the tympanic structures (Fig. 1): (1) absence of the tympanic membrane and tympanic annulus when the columella is absent; (2) absence of the tympanic membrane when the tympanic annulus is absent; (3) presence of the tympanic annulus and columella when the tympanic membrane is present; and (4) presence of the columella when the tympanic annulus is present.

In total, we scored the condition of the TME for 556 species and 51 genera of Bufonidae, representing >94% of all described species in the family. Among the sampled species, 239 were included in Pyron’s^30^ phylogenetic analysis. We also scored the conditions of these structures for 1860 of the 2538 non-bufonid anuran species (representing 53 families; see^15^) included by Pyron^30^, as well as 147 non-bufonid anuran species not included in this analysis. The only frog family not sampled by Pyron^30^ is the recently described Odontobatrachidae^31^. Although our outgroup sampling is not exhaustive, we included data for the vast majority of genera of all the families sampled by Pyron^30^. Details on material examined, considerations about character coding and character states scored for each transformation series, and literature sources are listed as Supplementary Material (section S1 of the Supplementary Information).

### Ancestral state reconstructions

We employed the most recent and densely sampled phylogenetic hypothesis available for Anura, that of Pyron^30^, for ancestral state reconstruction, and we discuss bufonid species not included in Pyron’s^30^ study but present in other analyses (e.g., well-supported results of^32^,^33^,^34^). We focused original data collection primarily on Bufonidae and relied more extensively on literature accounts for non-bufonids, some of which were unclear or ambiguous about the occurrence of specific structures. In particular, taxonomic accounts often use imprecise terminology to describe the external morphology of the otic region^35^. Consequently, we analysed the phylogenetic distribution of each of the TME structures in Bufonidae but only the columella in analyses of Anura.

To test the homology of the middle ear structures individually and explain their variation among anurans, most parsimonious ancestral state reconstructions^36^ on Pyron’s^30^ phylogenetic hypothesis were performed using Mesquite v3.03^37^. We further explored alternative evolutionary scenarios for the complete loss of all TME structures within Bufonidae with maximum likelihood ancestral reconstructions using the package APE^38^ and stochastic character mapping^39^ using phytools^40^ in R^41^. We compared Akaike Information Criterion (AIC) values for a maximum likelihood model in which transition rates were allowed to vary (ARD) throughout the tree and a maximum likelihood model in which transition rates between states were equal (ER). We used the most supported transition rate from our maximum likelihood analyses (ER) to estimate the number of gains and losses across Bufonidae using stochastic character mapping. Stochastic character mapping allowed us to explore the probability of ear transitions under various evolutionary scenarios, giving us a better understanding of the likelihood of regains throughout this family. We considered three scenarios: (1) equal transition rates, no restrictions; (2) equal transition rates and restricting the ancestor to being eared; and (3) a Dollo’s model (no regains possible). We ran 10,000 simulations per scenario and counted a state change whenever nodes switched from greater than 50% support for one character state to greater than 50% support for the other character state.

## Results

### Tympanic middle ear evolution in Anura

The occurrence of a columella is plesiomorphic in Anura, although the sister clade of all other anurans (Ascaphidae + Leiopelmatidae) lacks this structure (see Discussion). Given that the tympanic membrane and tympanic annulus are not fossilizable structures, their occurrence in fossil material cannot be assessed, making it impossible to determine if the presence of those structures is also plesiomorphic in Anura.

The TME is completely absent in at least some species of no fewer than 20 anuran families: Ascaphidae, Alsodidae, Batrachylidae, Bombinatoridae, Brachycephalidae, Brevicepitidae, Bufonidae, Calyptocephalellidae, Craugastoridae, Dicroglossidae, Hemisotidae, Leiopelmatidae, Leptodactylidae, Megophryidae, Microhylidae, Myobatrachidae, Nasikabatrachidae, Rhinophrynidae, Sooglossidae, and Telmatobiidae (section S1 of the Supplementary Information).

Ancestral character state reconstructions and detailed description of the occurrence of the columella in anuran families other than Bufonidae are provided in section S2 of the Supplementary Information. Ancestral state reconstruction using Pyron’s^30^ phylogenetic hypothesis shows that the complete loss of the TME, as evidenced by lack of the columella, occurred independently at least 25 times outside Bufonidae, plus two additional losses when taxa not included in Pyron’s^30^ study but present in other phylogenetic analyses are considered.

### Tympanic middle ear evolution in Bufonidae

**Figure 2:**
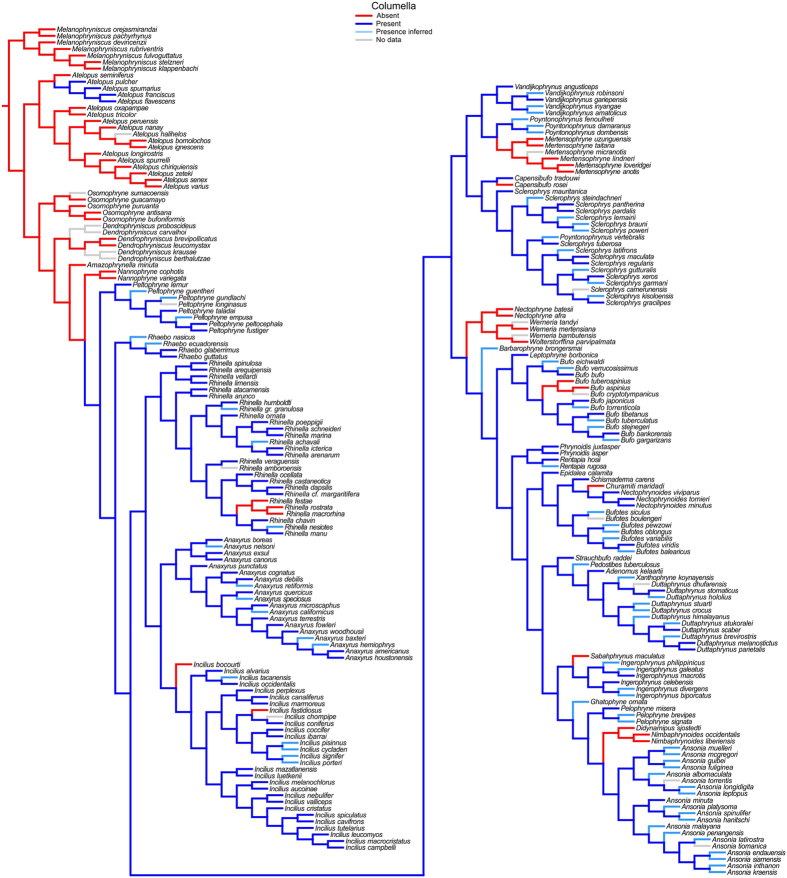
Partial phylogenetic tree of Pyron^30^ showing parsimony ancestral state reconstructions for the columella in Bufonidae. The absence of the columella is a synapomorphy of Bufonidae (see section S2 of the Supplementary Information), with independent regains in a subclade of *Atelopus, Frostius* (not included in this analysis, but see text), and the sister clade of *Nannophryne*, followed by 10 independent losses.

Based on the phylogenetic hypothesis of Pyron^30^ and the results of both the parsimony and probabilistic ancestral state reconstructions (Fig. 2 and section S3 of the Supplementary Information), the tympanic membrane, tympanic annulus, and columella were lost in the most recent common ancestor of bufonids, regained subsequently, and then repeatedly re-lost again. Below we summarize the results of the ancestral reconstructions of the tympanic membrane, tympanic annulus, and columella (the numbers of regains and re-losses of TME structures in Bufonidae differs somewhat when taxa not sampled by Pyron^30^ are considered; see sections S1 and S4 of the Supplementary Information and Discussion, below).

#### Parsimony ancestral state reconstruction

The tympanic membrane was lost in the most recent common ancestor of Bufonidae and reappeared in the sister clade of *Nannophryne*. Subsequent independent losses occurred at least 25 times (see sections S3 and S4 of the Supplementary Information). The absence of the tympanic annulus is the inferred ancestral condition in bufonids, with two independent regains: (a) within *Atelopus* in a clade composed of *A. flavescens*, *A. franciscus*, *A. pulcher*, and *A. spumarius*, and (b) in the sister clade of *Nannophryne*. The gain of the tympanic annulus in the latter clade was followed by 10 independent losses (see sections S3 and S4 of the Supplementary Information). Finally, the phylogenetic distribution of the gains and losses of the columella is identical to that of the tympanic annulus for the taxa included in the hypothesis of Pyron^30^ (see Fig. 2 and section S4 of the Supplementary Information). However, if bufonid species not included by Pyron^30^ are also considered, the columella sometimes occurs without a tympanic annulus (see Discussion and section S1 of the Supplementary Information).

#### Stochastic mapping of complete tympanic middle ear loss under various constraints

Our stochastic character mapping estimated similar patterns of TME loss and regain within Bufonidae under various evolutionary scenarios (see section S3 of the Supplementary Information). When we ran an equal rates model of evolution we found results similar to the parsimony reconstructions of the tympanic annulus and columella with strong support for an ancestor lacking these structures, two regains, and 10 losses within the tree. When we assumed the ancestor had these structures and a model of equal transition rates, we found support for 12 losses and still two regains. When restricting regains from occurring (Dollo’s model), we found a total of 17 losses across bufonids.

## Discussion

The lack of the columella in Ascaphidae and Leiopelmatidae, which together form the sister clade of all other extant anurans (=Lalagobatrachia), has generated much discussion about the plesiomorphic condition in Anura^29^,^42^,^43^,^44^. However, since most proanurans and stem anuran fossils have a columella (i.e. *Mesophryne beipiaoensis*, *Notobatrachus* spp., *Prosalirus bitis*, *Triadobatrachus massinoti*, *Yizhoubatrachus macilentus*^45^,^46^,^47^,^48^,^49^), the lack of this structure in Ascaphidae + Leiopelmatidae appears to be synapomorphic. A columella could not be identified in the stem anuran *Vieraella herbstii*; however, the state of preservation of the specimen is poor^50^, leading some authors to consider the occurrence of a columella to be unknown (e.g.,^47^) and others to consider it to be absent (e.g.,^43^). Regardless, given the phylogenetic position of *Vieraella herbstii*^46^,^47^,^50^, this controversy has no bearing on our inferences of the evolutionary history of this structure in anurans.

Although available evidence clearly indicates the plesiomorphic presence of the columella in Anura, it is unknown if the ancestral anuran also possessed a tympanic membrane and annulus. The tympanic membrane and tympanic annulus are not fossilizable structures, so their precise phylogenetic origin is unknown. As such, two scenarios are compatible with current evidence: the tympanic annulus and membrane might have been present in the most recent common ancestor of Anura and lost with the columella in Ascaphidae + Leiopelmatidae, or they might have arisen in Lalagobatrachia.

Among non-bufonid anurans, the TME was completely lost at least 27 times (see above). All these losses involve small clades scattered across the major lineages of Anura, implying several putative synapomorphies (e.g., *Atelognathus* + *Chaltenobatrachus*, *Brachycephalus*, Nasikabatrachidae + Sooglossidae, *Pseudophryne*, *Telmatobufo*) or autapomorphies (e.g., *Balebreviceps hilmani*, *Melanobatrachus indicus*, *Rhinophrynus dorsalis*). For other anuran clades (e.g., *Alsodes*, *Microhyla*, *Nanorana*, *Scutiger*, *Telmatobius*), denser taxon sampling is necessary to obtain adequate evidence to understand the evolution of the TME (see section S2 of the Supplementary Information for a more exhaustive discussion about the evolution of this structure in these and other non-bufonid species).

Although TME structures were lost repeatedly in Anura, TME evolution in Bufonidae is especially complex. All of the families that are closely related to Bufonidae^30^ (also see^51^,^52^) have a complete TME, making the absence of these structures a synapomorphy of Bufonidae. As such, the lack of a TME is plesiomorphic in the earliest diverging lineages (i.e., *Amazophrynella*, most species of *Atelopus*, *Dendrophryniscus*, *Oreophrynella*, *Osornophryne*, *Melanophryniscus*, and *Nannophryne*).

Independent of the methodological approach, available evidence indicates that the complete TME was regained within Bufonidae in *Frostius* and the sister clade of *Nannophryne*, whereas the tympanic annulus and columella were regained in a subclade of *Atelopus*. Thus, these structures are not homologous with the equivalent structures found in the TME of other anurans, although it seems likely that the underlying genetic basis for their development is homologous (i.e., deep homology^53^, see below). Subsequent losses occurred several times in different clades, indicating a complex evolutionary history of the TME in Bufonidae (see Fig. 2 and section S3 of the Supplementary Information).

The complex evolutionary history of the TME in Bufonidae is even more unusual when compared to other tetrapods. As noted above, although extant caecilians and salamanders do not possess a tympanic membrane or middle ear cavity, it has been hypothesized that a TME might have been present plesiomorphically and that the lateral elements might have been lost independently^5^,^54^. Regardless, although the remaining middle ear structures underwent extensive modification, the columella was lost only once in each group, having been greatly reduced or lost in salamandrid salamanders^1^,^55^ and lost in adult scolecomorphid caecilians (present as a cartilaginous element in fetal and juvenile *Scolecomorphus kirkii*^56^).

Among amniotes, TME loss is extremely rare. There are no documented losses among turtles (e.g.,^57^), archosaurs (e.g.,^58^,^59^,^60^), or mammals (e.g.,^61^), despite the remarkable middle ear transformations in fossorial and marine mammals^62^,^63^. Among lepidosaurs, numerous lineages have lost the lateral-most components of the TME, including Serpentes, Amphisbaenia, Agamidae, Diploglossidae, Gymnophthalmidae, Lanthonotidae, Phrynosomatidae, and Scincidae^11^,^60^,^64^,^65^,^66^, but the columella appears to be present in all but the pygopod lizard *Aprasia repens*^12^ and, possibly, the lamprophid snakes *Atractaspis* and *Xenocalamus*^13^.

With few exceptions, the development of TME structures in anurans follows a consistent sequence that might explain the consistent pattern of co-occurrence of middle ear structures and provides clues about the mechanisms involved in their loss and gain. First, the medial end of the pars media plectri develops as a chondrification within the connective tissue membrane spanning the fenestra ovalis adjacent to the already formed operculum^27^,^67^,^68^,^69^,^70^. Next, the pars interna plectri begins to chondrify and, with the incipient pars media, form the future stapedial footplate. Subsequently, a socket-like structure begins to be defined, articulating with the anterior edge of the operculum. The lateral-most portion of the stapedial footplate elongates to complete the formation of the shaft of the pars media plectri, which extends laterally towards the outside of the head. Meanwhile, the tympanic annulus and pars externa plectri develop as cartilaginous condensations associated with the posterior margin of the palatoquadrate. As the palatoquadrate swings posteriorly during metamorphosis, so too do the tympanic annulus and pars externa plectri. As the ontogenetic sequence of development of these structures progresses, they are positioned in the same medial-lateral plane. At this point, the partes media and externa plectri connect synchondrotically to each other and the tympanic annulus induces the differentiation of the tympanic membrane^27^,^70^.

The sequences of losses and gains appear to be related to the relative timing of the development of structures (heterochronies) and tissue differentiation phenomena. For example, Helff^69^ demonstrated the inductive effects of the tympanic annulus on the tegument to produce the differentiation of the tympanic membrane, which explains why the tympanic membrane never occurs in the absence of a tympanic annulus. Similarly, Hetherington^27^, Smirnov^71^, and Fabrezi and Goldberg^68^ emphasized the relatively late development of TME structures. Hetherington^27^ and Smirnov^71^ also observed that several species undergo post-metamorphic development of previously absent or undeveloped structures (e.g., *Sclerophrys regularis*, *Pseudacris crucifer*, *Bombina orientalis*). Meanwhile, Smirnov^72^ pointed out that developmental heterochronies (progenesis, neoteny, and post-displacement) seem to play a major role in the post-metamorphic development of these structures. All these events occur in specific sequences and their disruption in particular stages could produce the observed patterns of losses in the subsequent stages of development of the TME. Therefore, research into the genetic basis for the absence of induction of lateral elements promises to be a fruitful line of investigation.

Additionally, genetic mechanisms that directly regulate the expression of these ear structures might be involved. Knowledge of the origin of the components of the vertebrate auditory system is incipient generally and for anurans particularly. However, recent studies of *Xenopus laevis* support a model in which the cartilaginous elements of the TME are derived from three neural crest cell streams (see^73^): (1) the mandibular stream forms the tympanic annulus, (2) the hyoid stream gives rise to the partes media and externa plectri, and (3) the branchial stream forms the pars interna plectri. The consistent patterns of co-occurrences observed in anurans suggest that a direct role of regulatory genes and/or transcription factors might be involved in the tissular differentiation of the tympanic membrane due to inductive phenomena from the tympanic annulus. Also, it is likely that the development of the tympanic annulus and pars externa plectri (in the margins of the palatoquadrate) and the partes interna and media plectri (in the otic capsule) results from the initiation of a common developmental module, as in the morphogenesis of many other structures^74^,^75^. Unfortunately, information on the developmental control genes that lead to the formation of elements in the amphibian middle ear is unavailable. However, some genetic pathways involved in this differentiation process have been identified in other vertebrates and could be examined in frogs^76^.

The lateral–medial dependency between the presence and absence of tympanic middle ear structures appears also to be related to functional constraints: a tympanic membrane without a tympanic annulus or columella would have no acoustic function, as would a tympanic annulus without a columella. In contrast, the tympanic annulus retains its acoustic function in the absence of a tympanic membrane, and the columella remains acoustically functional even in the absence of both structures, as evidenced by the middle ears of salamanders^1^. This asymmetric functional dependency appears to have allowed these three structures to evolve sequentially rather than as a single transformation series (i.e., presence or absence of all the three structures), with losses and gains of each element occurring sequentially in a lateral–medial dependency, across the bufonid tree.

The losses, regains, and re-losses of TME structures in Bufonidae make true toads an excellent model to study the behavioural correlates of TME morphology. Previous studies have hypothesized a relationship between earlessness and aquatic or fossorial habitats and lack of acoustic communication or production of low-frequency calls^77^. Additionally, based on the limited evidence presently available (see section S5 of the Supplementary Information), the loss of TME structures in Bufonidae appears to be coincident with the origin of a scramble competition mating system in which males in dense aggregations attempt amplexus indiscriminately and struggle for possession of females^78^. In this mating system, acoustic territorial defence is absent and reliance on hearing for mate choice is greatly reduced or eliminated, as is the effectiveness of prezygotic isolating barriers like advertisement calls^78^, which presumably results in the natural interspecific hybridization observed in many bufonid species (see^79^ and references therein).

Nevertheless, although most of the species of early diverging clades of Bufonidae for which the mating system is known exhibit scramble competition (see section S5 of the Supplementary Information), the reproductive behaviour of most species is unknown, making the character state reconstruction of this behaviour at the root node of Bufonidae ambiguous. Similarly, both within Bufonidae and across Anura, many of the groups that lack a TME employ high frequency (>1 kHz) advertisement calls during reproductive communication (e.g., *Atelopus*^80^, *Bombina*^81^, *Brachycephalus*^82^, *Melanophryniscus*^83^, *Osornophryne*^84^, *Rhinophrynus*^85^, and *Sechellophryne* + *Sooglossus*^86^). Indeed, despite the absence of a TME and the occurrence of a scramble competition mating strategy, interspecific acoustic diversity is maintained in most genera (e.g.,^80^,^87^,^88^,^89^) suggesting that acoustic signals play a still unclear role in communication and/or mate choice. The maintenance of call diversity and widespread production of advertisement calls may be explained by extratympanic hearing pathways in earless frogs. Multiple extratympanic pathways, including a lung pathway (e.g., *Atelopus*^22^,^23^, *Bombina*^81^, *Nectophrynoides asperginis*^21^), an opercularis pathway (reviewed by^3^,^25^,^90^,^91^), and bone conduction enhanced by resonation of the oral cavity (*Sechellophryne*^92^) have been shown effective or hypothesized so far in a few earless species. Given that in at least some anurans airborne sounds are transferred via both tympanic and extratympanic pathways (reviewed by^3^,^25^), anurans may experience relaxed selective pressures on the TME if TME plasticity does not greatly affect acoustic acuity. If the generality of alternative sound transfer pathways for aerial sounds is corroborated across anuran diversity, then the pre-existence of alternative pathways for airborne sound transmission might explain the high rate of TME loss in anurans. Nevertheless, currently proposed sound localization pathways in anurans all require middle ear coupling^93^, leaving an alternative mechanism by which earless species localize audible sounds unknown.

## Concluding remarks

Since the tympanic annulus and membrane first arose in combination with the plesiomorphically present columella, either prior to the origin of Anura or in Lalagobatrachia (the clade formed by all anurans except Ascaphidae and Leiopelmatidae), our analysis indicates that the TME was completely lost at least 38 times in anurans, usually in small clades within diverse families. Bufonidae is exceptional within both Anura and among all tetrapods in that the loss of all TME structures preceded a radiation of more than 150 earless species followed by independent regains and many additional losses in most derived clades. In contrast, among the approximately 26500 species of amniotes the TME was completely lost only three times and was never regained. Available evidence suggests that losses/gains of each TME structure constitute independent transformation series that occur in a lateral-medial dependency, where heterochronic events and regulation via specific genetic mechanisms are implied during development. The complex pattern of TME evolution, extensive morphological and reproductive diversity, and maintenance of bioacoustic diversity despite the loss of TME structures make Bufonidae a promising model to study extratympanic pathways of sound transmission, the physiological and behavioural consequences of middle ear loss, and the underlying genetic and developmental mechanisms that shaped its remarkable TME diversity.

## Additional Information

**How to cite this article**: Pereyra, M. O. *et al*. The complex evolutionary history of the tympanic middle ear in frogs and toads (Anura). *Sci. Rep.* **6**, 34130; doi: 10.1038/srep34130 (2016).

## Supplementary Material

Supplementary Information
