# Supplementary material for: The complex evolutionary history of the tympanic middle ear in frogs and toads (Anura)
Source: Sci Rep. 2016 Sep 28;6:34130. doi: 10.1038/srep34130 (PMC5039693; doi:10.1038/srep34130)
Supplement: Supplementary Information [file srep34130-s1.pdf]

The complex evolutionary history of the tympanic middle ear in frogs and toads (Anura)

Authors

Martin O. Pereyra[1,†], Molly C. Womack[2,†], J. Sebastián Barrionuevo[1], Boris L. Blotto[1,3], Diego Baldo[4], Mariane Targino[3], Jhon Jairo Ospina-Sarria[3], Juan M. Guayasamin[5,6], Luis A. Coloma[7,8], Kim L. Hoke[2], Taran Grant[3,*], Julián Faivovich[1,9,*]

[1]Museo Argentino de Ciencias Naturales-CONICET, Buenos Aires, C1405DJR, Argentina

[2]Department of Biology, Colorado State University, Fort Collins, CO 80523, USA

[3]Departamento de Zoologia, Instituto de Biociências, Universidade de São Paulo, São Paulo, SP 05508-090, Brazil

[4]Laboratorio de Genética Evolutiva, Instituto de Biología Subtropical (CONICET-UNaM), Facultad de Ciencias Exactas Químicas y Naturales, Universidad Nacional de Misiones, Posadas, N3300LQF, Argentina

[5]Centro de Investigación de la Biodiversidad y Cambio Climático (BioCamb), Ingeniería en Biodiversidad y Cambio Climático, Facultad de Medio Ambiente, Universidad Tecnológica Indoamérica, Quito, EC170103, Ecuador.

[6]Colegio de Ciencias Biológicas y Ambientales COCIBA, Laboratorio de Biología Evolutiva, Universidad San Francisco de Quito, Quito, Ecuador.

[7]Centro Jambatu de Investigación y Conservación de Anfibios, Fundación Otonga, Geovanni Farina 566 y Baltra, San Rafael, Quito, Ecuador

[8]Ikiam, Universidad Regional Amazónica, Muyuna, Tena, Ecuador

[9]Departamento de Biodiversidad y Biología Experimental, Facultad de Ciencias Exactas y Naturales, Universidad de Buenos Aires, Buenos Aires, C1428EGA, Argentina.

[*]Correspondence: taran.grant@ib.usp.br; julian@macn.gov.ar

[†]These authors contributed equally to this work

Section S1. Specimens examined and details about character coding

1.1—Considerations about character coding

We scored the presence and absence of the tympanic membrane, tympanic annulus, and columella for Bufonidae and other families of Anura based on dissections, MicroCT images, osteological preparations, and bibliographic information. The morphology of the tympanic membrane and tympanic annulus were studied with the aid of a stereomicroscope. When differentiation of structures in the tympanic region was not evident externally, we performed dissections to ascertain the condition of the tympanic annulus. To study the condition of the columella, specimens were cleared and double-stained for bone and cartilage (Wassersug, 1976). MicroCT scans were performed in air with a desktop Skyscan 1173 (Bruker Skyscan, Aartselaar, Belgium) system at a scanning resolution of 10 μm. The images were reconstructed with NRecon (Bruker SkyScan, Aartselaar, Belgium) with a Feldkamp cone-beam algorithm (Feldkamp *et al.* 1984).

Lynch and Duellman (1997) reviewed some of the confusions and misinterpretations surrounding the conditions of the tympanic membrane and tympanic annulus in many taxonomic studies, and we followed their considerations when scoring the character states of both structures: the definition of a "tympanum" does not necessarily imply the presence of a tympanic membrane but can also refer only to a tympanic annulus. Thus, we only scored the tympanic membrane as present when we observed it ourselves or when published accounts explicitly describe it as a differentiated region of the skin or clearly depict it in the figures provided in publications. Regarding the condition of the columella, we scored it as present even if there are only rudiments of this structure in the adult forms. This latter situation is exceptional and was only reported for a few species of anurans (e.g., *Bombina* and *Telmatobius*).

1.2—Specimens examined.

Collection abbreviations are as follow: AMNH (American Museum of Natural History, New York, United States); CBF (Colección Boliviana de Fauna, La Paz, Bolivia); CENAI (Centro Nacional de Investigaciones Iológicas, Buenos Aires, Argentina); CFBH (Collection Célio F.B. Haddad, Universidade Estadual Paulista, Rio Claro, São Paulo, Brazil); DB (Diego Baldo field series); ICN (Instituto de Ciencias Naturales, Universidad Nacional de Colombia, Bogotá , Colombia); KU (University of Kansas Natural History Museum, Lawrence, United States); LGE (Instituto de Biología Subtropical, Universidad Nacional de Misiones, Posadas, Misiones, Argentina); MACN (Museo Argentino de Ciencias Naturales "Bernardino Rivadavia", Buenos Aires,

Argentina); MCP (Museu de Ciências e Tecnologia da PUCRS, Porto Alegre, Brazil.); MLP (Museo La Plata, La Plata, Argentina); MNCN (Museo Nacional de Ciencias Naturales, Madrid, Spain); MNRJ (Museu Nacional do Rio de Janeiro, Universidade Federal de Rio de Janeiro, Rio de Janeiro, Brazil); MZUSP (Museu de Zoologia, Universidade de São Paulo, São Paulo, Brazil); MNK (Museo de Historia Natural Noel Kempff, Santa Cruz de la Sierra, Bolivia); TG (Taran Grant field series); TNHC (Texas Memorial Museum, Austin, Texas, USA); UMMZ (University of Michigan Museum of Zoology, Ann Arbor, Michigan, United States); USNM (National Museum of Natural History, Washington, D.C., United States); ZUEC (Museu de História Natural, Universidade Estadual de Campinas, Campinas, São Paulo, Brazil).

BUFONIDAE

Amazophrynella minuta.—**Colombia**: Amazonas: Leticia (ICN 50301).

Anaxyrus canorus.—**United States**: California: Tioga Pass Mono-Tuolumne county border (USNM 311300); Tuolumne Yosemite National Park, Aspen Valley (USNM 311297).

Anaxyrus cognatus.—**United States**: New Mexico: Luna: Columbus (USNM 320094, 320103).

Anaxyrus houstonensis.—**United States**: Texas: Harris Fairbanks (USNM 542211); near Houston airport (USNM 542212).

Anaxyrus microscaphus.—**United States**: New Mexico: Grant: San Lorenzo (USNM 320140); near New Mexico Hwy 35, Mimbres River Drainage (USNM 320144).

Ansonia muelleri.—**Philippines**: Mindanao Island: Agusan Del Norte: Cabadbaran (USNM 305567, 305569).

Atelopus chiriquiensis.—**Costa Rica**: Limon: Pico Blanco (= Cerro Kamuk) (USNM 30643-4).

Atelopus cruciger.—**Venezuela**: Aragure (ICN 14387).

Atelopus flavescens.—**French Guiana**: Cayenne, 82 km S of, 3 km W of Kaw (USNM 331415, 331417).

Atelopus franciscus.—**French Guiana**: Cayenne: Crique Gregoire, Inini (USNM 192815).

Atelopus ignescens.—**Ecuador**: Imbabura: near Odovalo (= Otavalo) (USNM 236903–4).

Atelopus longirostris.—**Ecuador**: Pichincha: Llambo (USNM 193825); Tandayapa (USNM 193835).

Atelopus pulcher.—**Peru**: Amazonas: La Poza (USNM 569373); Shiringa (USNM 569381).

Atelopus varius.—**Colombia**: Chocó: Río Sucio (ICN 31489). **Costa Rica**: San Jose Desamparados, near, along Rio Jorco (USNM 279113, 279119).

Atelopus zeteki.—**Panama**: Coclé: El Valle, Cerro Gaital (USNM 129893, 129896).

Bufo bankorensis.—**Taiwan**: Kaohsiung Hsien: Kosempo (= Chiasien) (USNM 66082–3).

Bufo japonicus.—**Japan**: Honshu Island: Nagano: Karuizawa (USNM 51997–8).

Capensibufo rosei.—**South Africa**: Western Cape: Cape Town, Table Mountain (USNM 159110, 159113).

Dendrophryniscus leucomystax.—**Brazil**: São Paulo: Ilha Comprida (CFBH 19007).

Didynamipus sjostedti.—**Cameroon**: Southwest: Nguti, near, Banyang-Mbo Forest (USNM 505739).

Duttaphrynus scaber.—**Sri Lanka**: North Eastern Mannar: Marichchukkadi (USNM 254716, 254718).

Duttaphrynus stomaticus.—**Nepal**: Narayani: Chitwan Sauraha, vicinity of Smithsonian Institution Camp, Royal Chitwan National Park (USNM 266829, 266836).

Epidalea calamita.—**Spain**: Madrid (MACN 36530). **Germany**: Bavaria: Nuremberg (USNM 579882, 579890).

Frostius pernambucensis.—**Brazil**: Bahia (LGE 7566).

Incilius campbelli.—**Belize**: Toledo: Maya Mountain Forest Reserve, Snake Creek (USNM 498198, 498200).

Incilius canaliferus.—**Mexico**: Chiapas: La Esperanza (USNM 116005, 116019).

Incilius coniferus.—**Panama**: Bocas del Toro: Isla Cristobal Bocatorito camp (USNM 348058–9).

Incilius ibarrai.—**Honduras**: Ocotepeque: El Volcan (USNM 523715, 523723).

Incilius mazatlanensis.—**Mexico**: Sonora: Bahia Kino (USNM 214074, 214077).

Incilius occidentalis.—**Mexico**: Puebla: Tecamachalco (USNM 116539, 116541).

Ingerophrynus celebensis.—**Indonesia**: Celebes: Sulawesi Tengah: Toli Toli (USNM 52986); Bada Toeare (USNM 61189).

Leptophryne borbonica.—**Malaysia**: Selangor: Templer's Park (CENAI 7616).

Melanophryniscus atroluteus.—**Brazil**: Rio Grande do Sul: São Borja (MCP 12667).

Melanophryniscus cambaraensis.—**Brazil**: Rio Grande do Sul: (MCN 13473).

Melanophryniscus cupreuscapularis.—**Argentina**: Corrientes: Capital, Paraje Perichón (DB 1759, 1761).

Melanophryniscus devincenzii.—**Argentina**: Misiones: Candelaria, Ñu Pyahú (DB 753).

Melanophryniscus dorsalis.—**Brazil**: Rio Grande do Sul: Torres (MCP 235, 244).

Melanophryniscus estebani.—**Argentina**: San Luis: Carolina (MACN 35405).

Melanophryniscus fulvoguttatus.—**Brazil**: Mato Grosso Do Sul: Maracaju (USNM 107712, 107722).

Melanophryniscus klappenbachi.—**Argentina**: Chaco: Resistencia (MACN 38532).

Melanophryniscus macrogranulosus.—**Brazil**: Rio Grande do Sul: Maquiné, Barra do Ouro (MCP 8104).

Melanophryniscus montevidensis.—**Uruguay**: Maldonado: Laguna Garzón (DB 4120).

Melanophryniscus pachyrhynus.—**Uruguay**: Maldonado: Sierra de Ánimas (DB 3988).

Melanophryniscus setiba.—**Brazil**: Espírito Santo: Parque Estadual Paulo Cesar Vinha (CFBH 15735, 15745).

Melanophryniscus simplex.—**Brazil**: Santa Catarina: Campos Novos (MCP 9472–3).

Melanophryniscus spectabilis.—**Brazil**: Santa Catarina: (MLP 1808).

Melanophryniscus vilavelhensis.—**Brazil**: Parana: Parque Estadual de Vila Velha (DZUP 208).

Mertensophryne taitana.—**Malawi**: Rumphi (USNM 153417).

Nannophryne variegata.—**Argentina**: Santa Cruz: Lago del Desierto (MACN 41452). **Chile**: Magallanes: Mayne Harbor (USNM 15124).

Nectophryne batesii.—**Cameroon**: Littoral: Nguengue (USNM 571097, 571100).

Nectophrynoides tornieri.—**Tanzania**: Magrotto Mts. (CENAI 7674); Tanga: Muheza (USNM 226759).

Osornophryne bufoniformis.—**Ecuador**: Sucumbíos: Santa Bárbara (USNM 193537, 193540).

Osornophryne guacamayo.—**Colombia**: Putumayo: Santiago (ICN 47811).

Osornophryne talipes.—**Colombia**: Nariño (ICN 12256).

Peltophryne empusa.—**Cuba**: Isla de Pinos: near Santa Fe (MACN 39144).

Peltophryne fustiger.—**Cuba**: Pinar del Río: La Mulata (USNM 51864).

Peltophryne peltocephala.—**Cuba**: Camagüey: Camagüey (CENAI 8343).

Rentapia hosii.—**Malaysia**: Pahang: Ulu Tahan, Kuala Teku (CENAI 7716).

Rhaebo andinophrynoides.—**Colombia**: Nariño: Barbacoas (ICN 53535).

Rhaebo glaberrimus.—**Colombia**: Meta: Restrepo (ICN 36350).

Rhaebo guttatus.—**Colombia**: Vaupés: Mitú, Villa Fátima (ICN 34808).

Rhaebo haematiticus.—**Colombia**: Caldas: Samaná (ICN 3472, 43493).

Rhinella achalensis.—**Argentina**: Córdoba: Pampa de Achala (MACN 24953).

Rhinella arenarum.—**Argentina**: Salta: Santa Victoria, Lipeo (MACN 39220). **Uruguay**: Montevideo (USNM 70620, 70622).

Rhinella castaneotica.—**Brazil**: Para: Itaituba (USNM 518807, 518809).

Rhinella dapsilis.—**Ecuador**: Pastaza: Río Rutuno, tributary of Río Bobonaza (USNM 196951). **Brazil**: Amazonas: Borba, Río Madeira (USNM 201814).

Rhinella dorbignyi.—**Argentina**: Buenos Aires: Dolores (MACN 43695).

Rhinella fernandezae.—**Argentina**: Entre Rios: Chajarí (LGE 8716).

Rhinella icterica.—**Brazil**: Sâo Paulo, Quarter Parque Jabaquara (USNM 100954, 100957).

Rhinella major.—**Argentina**: Chaco: 9 de Julio: Las Breñas (LGE 8719).

Rhinella merianae.—**Guyana**: East Berbice: Dubulay Ranch (USNM 566017–8).

Rhinella ocellata.—**Brazil**: Goias: Río Araguaia USNM 130177); Mato Grosso: Utuariti, Río Papagaio (USNM 200548); Minas Gerais: Januaria (USNM 121334).

Rhinella ornata.—**Brazil**: Rio de Janeiro (USNM 70613–4).

Rhinella poeppigii.—**Peru**: San Martín (USNM 346829–30).

Rhinella schneideri.—**Bolivia**: Santa Cruz (USNM 281765). **Paraguay**: Boquerón: Filadelfia (USNM 340561).

Rhinella spinulosa.—**Argentina**: Mendoza: Uspallata (MACN 17574, 17567).

Schismaderma carens.—**Malawi**: Rumphi (USNM 153377, 153380). **South Africa**: Natal: Durban (CENAI 5984).

Sclerophrys capensis.—**South Africa**: Western Cape: Cape Town (CENAI 6851).

Sclerophrys gracilipes.—**Congo**: Likouala: Impongui (USNM 576239, 576391).

Sclerophrys mauritanica.—**Morocco**: Tetouan: Larache, 20 km SE of (USNM 346809, 346811).

Sclerophrys tuberosa.—**Cameroon**: Littoral: Ekomtolo (USNM 571083); Southwest: Nfainchang, near, Mt. Entali (USNM 324327).

Strauchbufo raddei.—**China**: Manchuria: Hei Sui (USNM 53371–2).

Vandijkophrynus angusticeps.—**South Africa**: Cape Of Good Hope: Kommetjie, near Cape Town (USNM 165087, 165089); Western Cape: Durbanville, near Cape Town (CENAI 6466).

Vandijkophrynus gariepensis.—**Lesotho**: Butha-Buthe (USNM 239871, 239873).

Werneria mertensiana.—**Cameroon**: Littoral: Nguengue (USNM 571101, 571105).

Wolterstorffina parvipalmata.—**Cameroon**: Littoral: Nguengue (USNM 571108–9).

OUTGROUPS

Adelphobates castaneoticus.—**Brazil**: Pará: near Cachoeira Juruá, Río Xingú (AMNH 133451–5).

Adelphobates galactonotus.—**Brazil**: Pará: Cachoeira do Limão, Río Tapajós (AMNH 128232–3).

Allobates femoralis.—**Colombia**: Putumayo: 10 km (airline) S Mocoa (AMNH 85258, 85260).

Allobates insperatus.—**Ecuador**: Napo: Santa Cecilia (KU 109310, 149671).

Allobates juanii.—**Colombia**: Meta: Acacias, Portachuelo (ICN 5097).

Allobates kingsburyi.—**Ecuador**: Pastaza: Abitagua Napo-Pastaza (UMMZ 217617).

Allobates talamancae.—**Costa Rica**: Puntarenas: Osa Peninsula, Corcovado National Park (UMMZ 193379).

Allobates undulatus.—**Venezuela**: Amazonas: Cerro Yutajé (AMNH 159141–2).

Alsodes gargola.—**Argentina**: Neuquén: Aluminé (MACN 37845)

Ameerega bassleri.—**Peru**: San Martín: Pachiza, Río Huallaga (AMNH 43402).

Ameerega bilinguis.—**Colombia**: Putumayo: 10 km (airline) S Mocoa, (AMNH 85215, 85219).

Ameerega hahneli.—**Peru**: Loreto: 5 rd km NE Previsto, near Boquerón del Padre Abad, upper Río Aguaytía (AMNH 118421).

Ameerega petersi.—**Peru**: Huánuco: Monte Alegre, Río Pachitea (AMNH 43016).

Ameerega picta.—**Bolivia**: Beni: Prov. Ballivian: Lago del Gringo, 10 km N of Puerto Salinas, 1 km from Beni River (UMMZ 184099).

Ameerega silverstonei.—**Peru**: Huánuco: 30 km NE Tingo María, Cordillera Azul (AMNH 91847–9).

Ameerega trivittata.—**Suriname**: Brokopondo: Brownsberg Nature Park, near Mazaroni Top (AMNH 118431).

Andinobates claudiae.—**Panama**: Bocas del Toro: Isla Colón, near La Gruta (AMNH 103514).

Andinobates fulguritus.—**Panama**: Panamá: km 12.8 on El Llano-Cartí Rd, 290 m (AMNH 89448).

Anomaloglossus beebei.—**Guyana**: near Kaieteur Falls (AMNH 18683).

Aplastodiscus perviridis.—**Argentina**: Misiones: Guaraní (MACN 37040).

Arcovomer passarelii.—**Brazil**: Espírito Santo: Linhares (CFBH 2181).

Aromobates nocturnus.—**Venezuela**: Trujillo: about 2 km (airline) ESE Agua de Obispos (AMNH 129940, 130014).

Atelognathus patagonicus.—**Argentina**: Neuquén: Laguna Blanca (CENAI 1070).

Batrachyla leptopus.—**Argentina**: Chubut: Desemboque (MACN 41291).

Ceratophrys cranwelli.—**Argentina**: Santa Fe: Vera (MACN 42340).

Colostethus panamensis.—**Panama**: Veraguas: 6.12 km N Santa Fe N of Altopiedra and Agricultural School in montane area called Buenos Aires (UMMZ 167459).

Colostethus pratti.—**Panama**: Darién: Río Jaque, 1.5 km above Río Imamadó (AMNH 118364, 118371).

Crossodactylus schmidtii.—**Argentina**: Misiones: Cainguás: Parque Provincial Salto Encantado (LGE 164), San Vicente (MACN 35122).

Chacophrys pierottii.—**Argentina**: Chaco: General Güemes, 10 km S Misión Nueva Pompeya (LGE 7716).

Chiasmocleis avilapiresae.—**Brazil**: Mato Grosso: Aripuanã (MNRJ 44231, 44283).

Chiasmocleis capixaba.—**Brazil**: Bahia: Nova Viçosa (MNRJ 18924, 19155).

Chiasmocleis carvalhoi.—**Brazil**: Rio de Janeiro: Magé (MNRJ 55166).

Chiasmocleis leucosticta.—**Brazil**: São Paulo: Ribeirão Branco (MNRJ 17901).

Chiasmocleis schubarti.—**Brazil**: Linhares: Espírito Santo (MNRJ 22961).

Ctenophryne aequatorialis.—**Ecuador**: Azuay (MNRJ 59150).

Ctenophryne aterrima.—**Costa Rica**: Cartago (MNRJ 59144).

Ctenophryne geayi.—**Brazil***:* Mato Grosso: Aripuanã (MNRJ 44208).

Cycloramphus boraceiensis.—**Brazil**: Rio de Janeiro: Paraty (CFBH 17308).

Dasypops schirchi.—**Brazil**: Espírito Santo: Linhares (MNRJ 22684).

Dendrobates auratus.—**Costa Rica**: Puntarenas: 8 km ENE Palmar Norte (AMNH 118524). **Panama**: Panama: Isla Tobago (AMNH 118528).

Dendrobates tinctorius.—**Brazil**: Amapá: Serra do Navio (KU 93147).

Dendrobates truncatus.—**Colombia**: Tolima: Shore of Río Gualí, 1–2 km above Mariquita (AMNH 118401, 118403).

Dendropsophus minutus.—**Argentina**: Salta: Isla de Cañas (MACN 1302).

Elachistocleis bicolor.—**Argentina**: Formosa: Bermejo, Ing. Enrique H. Faure (MACN 37324).

Dermatonotus muelleri.—**Brazil**: Bahia: Itajibá (MNRJ 19299).

Dyscophus guineti.—pet trade (MNRJ 59154).

Epipedobates boulengeri.—**Colombia**: Cauca: Isla Gorgona (USNM 145253).

Epipedobates espinosai.—**Ecuador**: Pichincha: Río Baba, 5–10 km SSW Santo Domingo de los Colorados (AMNH 118411, 118417).

Elachistocleis bicolor.—**Brazil**: Paraná: Bituruna (MNRJ 6932).

Espadarana prosoblepon.—**Colombia**: Antioquia: Urrao: Parque Natural Nacional Las Orquideas, Quebrada Honda (ICN 19657).

Eupsophus roseus.—**Argentina**: Neuquén: Huiliches: Lago Curruhue (MACN 37981).

Gastrophryne carolinensis.—**United States**: Florida (MZUSP 5753).

Gastrotheca christiani.—**Argentina**: Jujuy: Ledesma, Valle Grande (CENAI 3209).

Gastrotheca riobambae.—**Ecuador**: Imbabura: Cotacachi (MACN 34025).

Geocrinia victoriana.—**Australia**: Victoria: 96 km NW Melbourne (KU 186877).

Haddadus binotatus.—**Brazil**: São Paulo: Serra de Paranapiacaba (MACN 17042).

Hamptophryne boliviana.—**Brazil**: Acre: Marechal Thaumaturgo (MNRJ 28922).

Hylorina sylvatica.—**Chile**: Llanquihué: Chamiza (CENAI 1870).

Hyloxalus awa.—**Ecuador**: Pichincha: 8 km SE Santo Domingo de los Colorados, Hacienda Delta (UMMZ 217614).

Hyloxalus bocagei.—**Ecuador**: Pastaza: Hills N of Mera (UMMZ 182465).

Hyloxalus elachyhistus.—**Ecuador**: Loja (KU 120543).

Hyloxalus pulchellus.—**Ecuador**: Napo: Río Azuela, Quito-Lago Agrio Road, eastern base Volcán Reventador (AMNH 89538).

Hyloxalus sauli.—**Ecuador**: Napo: Río Nachiyacu S of Venecia (UMMZ 182477).

Hyloxalus subpunctatus.—**Colombia**: Cundinamarca: Bogotá, near Monserrate (UMMZ 221158–9).

Hyloxalus sylvaticus.—**Peru**: SW slope Abra de Porculla (KU 164093).

Hyloxalus vertebralis.—**Ecuador**: Cañar: Cuenca (KU 120633–4).

Hypopachus variolosus.—**San Salvador**: El Salvador (MZUSP 77593).

Hypsiboas picturatus.—**Colombia**: Antioquia: Dabeiba (ICN 9279, 9275).

Hypsiboas pulchellus.—**Argentina**: Corrientes: Estancia Celina (LGE 2757, 2759).

Kaloula pulchra.—pet trade (MNRJ 51715).

Lepidobatrachus laevis.—**Argentina**: Formosa: Bermejo: Laguna Yema (MACN 39062).

Leptodactylus bufonius.—**Argentina**: Chaco: General Güemes: Wichi (LGE 12152).

Leptodactylus chaquensis.—**Argentina**: Chaco: General Güemes: near to Misión Nueva Pompeya (LGE 12002).

Leptodactylus latinasus.—**Argentina**: Chaco: 25 km NE Fuerte Esperanza (LGE 10524).

Leptodactylus latrans.—**Argentina**: Corrientes: Capital: Paraje Perichón (LGE 11278).

Leptodactylus mystacinus.—**Argentina**: Misiones: Guaraní: San Vicente (MACN 43782).

Leptodactylus podicipinus.—**Argentina**: Chaco: Antequera (LGE 10523).

Leptodactylus syphax.—**Brazil**: Goiás: Minaçu, Usina Hidrelétrica Serra da Mesa
(LGE 10626–7).

Physalaemus ephippifer.—**Brazil**: Pará Santa Bárbara: Reserva Privada Gunma (LGE 2659).

Pseudopaludicola boliviana.—**Argentina**: Corrientes: General Paz: Itá Ibaté (LGE 3026).

Pseudopaludicola saltica.—**Brazil**: Goiás: Sitio D´Abadia (LGE 3066).

Limnomedusa macroglossa.—**Argentina**: Misiones: Colonia Delicia (LGE 10520).

Lysapsus limellum.—**Argentina**: Corrientes: San Miguel, 10 km N Loreto (MACN 39478).

Mannophryne collaris.—**Venezuela**: Mérida (UMMZ 217615).

Mannoprhyne herminae.—**Venezuela**: Aragua: Río Ocumare (UMMZ 210143–4).

Mannophryne trinitatis.—**Trinidad and Tobago**: Northern Range, 8 km (airline) N Arima (AMNH 118384, 118389).

Minyobates steyermarki.—**Venezuela**: Amazonas: SW sector Cerro Yapacana, 900 m (AMNH 118579).

Mixophyes fasciolatus.—**Australia**: Queensland: Bellthorpe State Forest (KU 179957).

Myersiella microps.—**Brazil**: Rio de Janeiro: Rio de Janeiro (MNRJ 27545).

Odontophrynus achalensis.—**Argentina**: Córdoba: Pampa de Achala (CENAI 2972)

Odontophrynus americanus.—**Argentina**: Misiones: Posadas (LGE 9094–5).

Oophaga arborea.—**Panama**: Chiriquí: continental divide above upper Quebrada de Arena (AMNH 116761).

Oophaga granulifera.—**Costa Rica**: 4.5 km W Rincón de Osa (KU 110223).

Oophaga histrionica.—**Colombia**: Chocó: Risaralda: 7 km (airline) SE Santa Cecilia, upper Río San Juan (AMNH 118458, 118461–2).

Oophaga lehmanni.—**Colombia**: Valle del Cauca: 13 km W Dagua, Río Anchicayá drainage (AMNH 88231, 118442).

Oophaga pumilio.—**Panama**: Bocas del Toro: East end Isla Escudo de Veraguas, (AMNH 118510).

Oophaga speciosa.—**Panama**: Chiriquí: Continental divide above upper Quebrada de Arena (AMNH 118447, 118454).

Oophaga sylvatica.—**Ecuador**: Pichincha: About 10 mi S of Santo Domingo de los Colorados, in banana plantation (AMNH 88225–6).

Oophaga vicentei.—**Panama**: Coclé: East shoulder Cerro Caracol (AMNH 114587).

Paratelmatobius poecilogaster.—**Brazil**: São Paulo: São Luis do Paraitinga (CFBH9877).

Phrynomantis microps.—pet trade (MNRJ 51711).

Phyllobates aurotaenia.—**Colombia**: Chocó: Vicinity of Playa de Oro, upper Río San Juan (AMNH 161108).

Phyllobates bicolor.—**Colombia**: Risaralda: about 7 km (airline) SE Santa Cecilia (AMNH 98256).

Phyllobates lugubris.—**Panama**: Bocas del Toro: ca. 5 km W Almirante (AMNH 118554, 118557).

Phyllobates terribilis.—**Colombia**: Cauca: Quebrada Guanguí, about .5 km above junction with Río Patia, in upper Río Saija drainage, 100–200 m, (AMNH 86319).

Phyllobates vittatus.—**Costa Rica**: Puntarenas: 8 km ENE Palmar Norte, 90 m, (AMNH 118542–5).

Phyllodytes luteolus.—**Brazil**: Bahia: Nova Viçosa (MNRJ 23275)

Phyllomedusa azurea.—**Argentina**: Chaco: Road between Resistencia and Sáenz Peña (CENAI 7772).

Phyllomedusa sauvagii.—**Argentina:** Formosa: Ramón Lista (MACN 38145)

Phyllomedusa tetraploidea.—**Argentina**: Misiones: General Manuel Belgrano, near to Leiten (LGE 2293).

Physalaemus albonotatus.—**Argentina**: Formosa: Tatané (LGE 10513, 10516).

Physalaemus biligonigerus.—**Argentina**: Santiago del Estero: Totora Pampa (LGE 10527).

Physalaemus cuvieri.—**Argentina**: Misiones: Villa Lanús (LGE 10512).

Physalaemus nattereri.—**Paraguay**: Amambay: Estancia Pirá Potrero, near Río Aquidabán (LGE 10518).

Pleurodema kriegi.—**Argentina**: Córdoba: Pampa de Achala (CENAI 5170).

Pleurodema marmoratum.—**Argentina**: Jujuy: El Quemado (LGE 6193).

Pleurodema tucumanum.—**Argentina**: Santiago del Estero: Totora Pampa (LGE 10636–7, 10514).

Pristimantis duellmani.—**Ecuador**: Carchi: 14 km SE Maldonado (KU 179268).

Pristimantis gentryi.—**Ecuador**: Cotopaxi: Pilalo, ca. 9 km (airline) E of Latacunga (USNM 239768)

Pristimantis juanchoi.—**Colombia**: Valle Del Cauca: Santiago De Cali (ICN 35063).

Pristimantis palmeri.—**Colombia**: Valle Del Cauca: Carretera El Cairo (ICN 29348).

Pristimantis thymalopsoides.—**Ecuador**: Cotopaxi: Pilaló (KU 177878).

Pristimantis surdus.—**Ecuador**: Pichincha: 7 km W Aloag, N slope Cerro Corazon (KU 109077).

Proceratophrys avelinoi.—**Argentina**: Misiones: Guaraní: San Vicente (MACN 36854).

Proceratophrys boiei.—**Brazil**: Santa Catarina: Florianopolis: Praia dos Naufragados (LGE 10564).

Pseudis minuta.—**Argentina**: Entre Ríos: Islas del Ibicuy (MACN 40425).

Pseudopaludicola falcipes.—**Argentina**: Corrientes: Perugorría (LGE 2969, 3105).

Pseudopaludicola mystacalis.—**Argentina**: Corrientes: Caá Catí (LGE 2842).

Ranitomeya imitator.—**Peru**: San Martín: Km 33, Carretera Tarapoto-Yurimagaus, Valle del Río Cainarache, 500–650 m (KU 209412–3).

Ranitomeya reticulata.—**Peru**: Loreto: 3 km airline SSW Mishana, on Río Nanay, 150 m (AMNH 103676, 103680–1).

Ranitomeya ventrimaculata.—**Peru**: Loreto: 3 km NE Pebas on Río Amazonas, 100 m (AMNH 103603–4).

Rheobates palmatus.—**Colombia**: Cundinamarca: Anolaima (AMNH 13472).

Rhinoderma darwinii.—**Chile**: Valdivia: Mehuín (CENAI 5491).

Scaphiophryne marmorata.—Pet trade (MNRJ 59152).

Scarthyla goinorum.—**Brazil**: Acre: Cruzeiro do Sul (TG 2774).

Silverstoneia flotator.—**Panama**: Coclé: Barro Colorado Island (KU 77678).

Silverstoneia nubicola.—**Panama**: Chiriquí: Río Frijoles (UMMZ 145585).

Scinax acuminatus.—**Argentina**: Chaco: Antequera (LGE 10534).

Scinax fuscovarius.—**Argentina**: Salta: Isla de Cañas (MCN 1301).

Scinax catharinae.—**Brazil** (MZUSP 55892).

Scinax cruentommus.—**Ecuador:** Sucumbios: Santa Cecilia (KU 111928).

Scinax garbei.—**Peru**: Loreto (AMNH 86792).

Scinax nebulosus.—**Bolivia** (MNK 886).

Scinax perpusillus.—**Brazil:** Rio de Janeiro: Recreio das Bandeirantes (THNC 37203).

Scinax ruber.—**Bolivia** (MNK 1640).

Scinax squalirostris.—**Argentina:** Santa Fe: Vera: Ea. Las Gamas (MACN 36970).

Sphaenorhynchus lacteus.—**Brazil**: Acre: Cruzeiro do Sul, Humaitá do Moa (ZUEC 5429).

Stereocyclops histrio.—**Brazil**: Bahia: Una (MZUSP 138155).

Stereocyclops incrassatus.—**Brazil***:* Espírito Santo: Linhares (MNRJ 22810).

Stereocyclops parkeri.—**Brazil**: Rio de Janeiro: Macaé (MNRJ 43987, 47478).

Strabomantis necerus.—**Colombia**: Valle Del Cauca: Restrepo: Vereda Campo Alegre (ICN 13229).

Synapturanus mirandaribeiroi.—**Brazil**: Amazonas: Oriximiná (MNRJ 52832).

Telmatobius culeus.—**Bolivia**: La Paz: Manco Kapac, Isla Tariquí, Lago Titicaca (CBF 4050, 4057).

Telmatobius hintoni.—**Bolivia**: Cochabamba: Cochabamba (MNCN 17361–2).

Telmatobius marmoratus.—**Bolivia**: La Paz: Charazani (CBF 3622). Huatajata, Lago Titicaca (CBF 2167).

Telmatobius niger.—**Ecuador**: Cañar: ca 8 km NW Biblián (KU 131795).

Telmatobius sibiricus.—**Bolivia**: Cochabamba: Río Chua Khocha, Sierra de la Siberia, P. N. Carrasco-Ichilo (MNCN 17364).

Telmatobius simonsi.—**Bolivia**: Chuquisaca: El Palmar (CBF 3081–2); Santa Cruz: 15 km from Mairana (MNCN 17366).

Telmatobius truebae.—**Peru**: Amazonas: E slope Abra Chanchillo, 44 km ENE Balsas (KU 212464); Pomacochas (=Florida) (KU 212477–80).

Telmatobius verrucosus.—**Bolivia**: La Paz: Zongo, Laguna Viscachani (CBF 2765); Apolobamba (CBF 5372).

Telmatobius yuracare.—**Bolivia**: Cochabamba: La Siberia (MNCN 16645); Río Apaza (MNCN 16646).

Thoropa taophora.—**Brazil**: São Paulo: Ubatuba (CFBH12734).

Vitreorana uranoscopa.—**Argentina**: Misiones: Parque Provincial Esmeralda (LGE 10521).

Xenohyla truncata.—**Brazil**: Rio de Janeiro: Maricá, Restinga de Maricá (MNRJ 33276).

1.3—Character states scored for each transformation series. 0: absent, 1: present, ?: unknown character state, [inf]: character state inferred following the lateral–medial dependency between the presence and absence of tympanic middle ear structures, [†]states codified from late larval stages (> Gosner Stage 42), *adults only have rudiments of columella

Bufonidae included in Pyron (2014)			
**Species**	**Tympanic membrane**	**Tympanic annulus**	**Columella**
Adenomus kelaartii	0	1	1
Amazophrynella minuta	0	0	0
Anaxyrus americanus	1	1	1
Anaxyrus baxteri	1	1	1[inf]
Anaxyrus boreas	1	1	1
Anaxyrus californicus	1	1	1[inf]
Anaxyrus canorus	1	1	1
Anaxyrus cognatus	1	1	1
Anaxyrus debilis	1	1	1
Anaxyrus exsul	0	1	1
Anaxyrus fowleri	1	1	1
Anaxyrus hemiophrys	1	1	1[inf]
Anaxyrus houstonensis	1	1	1
Anaxyrus microscaphus	1	1	1
Anaxyrus nelsoni	1	1	1[inf]
Anaxyrus punctatus	1	1	1
Anaxyrus quercicus	1	1	1
Anaxyrus retiformis	?	1	1[inf]
Anaxyrus speciosus	1	1	1[inf]
Anaxyrus terrestris	1	1	1
Anaxyrus woodhousii	1	1	1
Ansonia albomaculata	0	1	1[inf]
Ansonia endauensis	1	1	1[inf]
Ansonia fuliginea	1	1	1[inf]
Ansonia guibei	1	1	1[inf]
Ansonia hanitschi	1	1	1[inf]
Ansonia inthanon	1	1	1[inf]
Ansonia kraensis	1	1	1[inf]
Ansonia latirostra	1	1	1[inf]
Ansonia leptopus	1	1	1[inf]
Ansonia longidigita	1	1	1[inf]
Ansonia malayana	1	1	1[inf]
Ansonia mcgregori	0	1	1[inf]
Ansonia minuta	1	1	1
Ansonia muelleri	0	1	1
Ansonia penangensis	1	1	1[inf]
Ansonia platysoma	1	1	1[inf]
Ansonia siamensis	0	1	1[inf]

Ansonia spinulifer	1	1	1[inf]
Atelopus bomolochos	0	0	0
Atelopus chiriquiensis	0	0	0
Atelopus flavescens	0	1	1
Atelopus franciscus	0	0&1	1
Atelopus halihelos	0	?	?
Atelopus ignescens	0	0	0
Atelopus longirostris	0	0	0
Atelopus nanay	0	0	0
Atelopus oxapampae	0	0	0
Atelopus peruensis	0	0	0
Atelopus pulcher	0	1	1
Atelopus seminiferus	0	0	0
Atelopus senex	0	0	0
Atelopus spumarius	0	1	1
Atelopus spurrelli	0	0	0
Atelopus tricolor	0	0[inf]	0
Atelopus varius	0	0	0
Atelopus zeteki	0	0	0
Barbarophryne brongersmai	1	1	1[inf]
Bufo aspinius	0	0[inf]	0
Bufo bankorensis	1	1	1
Bufo bufo	0&1	1	1
Bufo cryptotympanicus	0	?	?
Bufo eichwaldi	1	1	1[inf]
Bufo gargarizans	1	1	1[inf]
Bufo japonicus	1	1	1
Bufo stejnegeri	0	1	1[inf]
Bufo torrenticola	0	1	1[inf]
Bufo tuberculatus	1	1	1[inf]
Bufo tuberospinius	0	0[inf]	0
Bufo verrucosissimus	1	1	1[inf]
Bufotes balearicus	?	1	1[inf]
Bufotes oblongus	1	1	1[inf]
Bufotes pewzowi	1	1	1[inf]
Bufotes siculus	1	1	1[inf]
Bufotes variabilis	1	1	1[inf]
Bufotes viridis	1	1	1
Capensibufo rosei	0	0	0
Capensibufo tradouwi	1	1	1
Churamiti maridadi	0	0	0
Dendrophryniscus berthalutzae	0	?	?
Dendrophryniscus brevipollicatus	0	0	0
Dendrophryniscus carvalhoi	0	?	?
Dendrophryniscus krausae	0	?	?
Dendrophryniscus leucomystax	0	0	0

Dendrophryniscus proboscideus	0	0	?
Didynamipus sjostedti	0	0	0
Duttaphrynus atukoralei	?	1	1^{inf}
Duttaphrynus brevirostris	?	1	1^{inf}
Duttaphrynus crocus	1	1	1^{inf}
Duttaphrynus himalayanus	0	1	1^{inf}
Duttaphrynus hololius	1	1	1^{inf}
Duttaphrynus melanostictus	1	1	1
Duttaphrynus parietalis	1	1	1
Duttaphrynus scaber	1	1	1
Duttaphrynus stomaticus	1	1	1
Duttaphrynus stuarti	1	1	1^{inf}
Epidalea calamita	0	1	1
Ghatophryne ornata	1	1	1^{inf}
Incilius alvarius	1	1	1
Incilius aucoinae	1	1	1
Incilius bocourti	0	0^{inf}	0
Incilius campbelli	1	1	1
Incilius canaliferus	1	1	1
Incilius cavifrons	1	1	1
Incilius chompipe	0	?	?
Incilius coccifer	1	1	1
Incilius coniferus	1	1	1
Incilius cristatus	0	1	1
Incilius cycladen	1	1^{inf}	1^{inf}
Incilius fastidiosus	0	0	0
Incilius ibarrai	1	1	1
Incilius leucomyos	1	1	1
Incilius luetkenii	1	1	1
Incilius macrocristatus	1	1	1
Incilius marmoreus	1	1	1
Incilius mazatlanensis	1	1	1
Incilius melanochlorus	1	1	1
Incilius nebulifer	?	?	1
Incilius occidentalis	0	1	1
Incilius perplexus	1	1	1
Incilius pisinnus	1	1	1^{inf}
Incilius porteri	1	1^{inf}	1^{inf}
Incilius signifer	1	1	1^{inf}
Incilius spiculatus	1	1	1
Incilius tacanensis	1	1^{inf}	1^{inf}
Incilius tutelarius	1	1	1
Incilius valliceps	1	1	1
Ingerophrynus biporcatus	1	1	1^{inf}
Ingerophrynus celebensis	1	1	1
Ingerophrynus divergens	1	1	1^{inf}

Ingerophrynus galeatus	1	1	1[inf]
Ingerophrynus macrotis	1	1	1
Ingerophrynus philippinicus	1	1	1[inf]
Leptophryne borbonica	0	1	1
Melanophryniscus devincenzii	0	0	0
Melanophryniscus fulvoguttatus	0	0	0
Melanophryniscus klappenbachi	0	0	0
Melanophryniscus pachyrhynus	0	0	0
Melanophryniscus rubriventris	0	0	0
Melanophryniscus stelzneri	0	0	0
Mertensophryne anotis	0	0	0
Mertensophryne lindneri	0	0	0
Mertensophryne loveridgei	0	0[inf]	0
Mertensophryne micranotis	0	?	?
Mertensophryne taitana	0	0	0
Mertensophryne uzunguensis	0	0	0
Nannophryne cophotis	0	0[inf]	0
Nannophryne variegata	0	0	0
Nectophryne afra	0	0	0
Nectophryne batesii	0	0[inf]	0
Nectophrynoides minutus	1	1	1
Nectophrynoides tornieri	1	1	1
Nectophrynoides viviparus	1	1	1
Nimbaphrynoides occidentalis	0	0	0
Osornophryne antisana	0	0	0
Osornophryne bufoniformis	0	0	0
Osornophryne guacamayo	0	0	0
Osornophryne puruanta	0	0[inf]	0
Osornophryne sumacoensis	0	?	?
Pedostibes tuberculosus	1	1	1[inf]
Pelophryne brevipes	1	1	1[inf]
Pelophryne misera	1	1	1
Pelophryne signata	1	1	1[inf]
Peltophryne empusa	1	1	1[inf]
Peltophryne fustiger	1	1	1
Peltophryne guentheri	1	1	1[inf]
Peltophryne gundlachi	1	1	1[inf]
Peltophryne lemur	1	1	1
Peltophryne longinasus	0	?	?
Peltophryne peltocephala	1	1	1
Peltophryne taladai	1	1	1
Phrynoidis asper	1	1	1
Phrynoidis juxtasper	1	1	1
Poyntonophrynus damaranus	0	1	1[inf]
Poyntonophrynus dombensis	1	1	1[inf]
Poyntonophrynus fenoulheti	1	1	1[inf]

Poyntonophrynus vertebralis	1	1	1^{inf}
Rentapia hosii	1	1	1
Rentapia rugosa	1	1	1^{inf}
Rhaebo ecuadorensis	0	1^{inf}	1^{inf}
Rhaebo glaberrimus	1	1	1
Rhaebo guttatus	1	1	1
Rhaebo nasicus	0	1	1^{inf}
Rhinella achavali	1	1	1^{inf}
Rhinella amboroensis	0	?	?
Rhinella arenarum	1	1	1
Rhinella arequipensis	1	1	1
Rhinella arunco	1	1	1
Rhinella atacamensis	1	1	1
Rhinella castaneotica	0	1	1
Rhinella chavin	1	1	1
Rhinella dapsilis	1	1	1
Rhinella festae	0	0	0
Rhinella granulosa	1	1	1^{inf}
Rhinella humboldti	1	1	1
Rhinella icterica	1	1	1
Rhinella limensis	1	1	1
Rhinella macrorhina	0	0	0
Rhinella manu	1	1	1
Rhinella margaritifera	1	1	1
Rhinella marina	1	1	1
Rhinella nesiotes	1	1	1^{inf}
Rhinella ocellata	1	1	1
Rhinella ornata	1	1	1
Rhinella poeppigii	1	1	1
Rhinella rostrata	0	0	0
Rhinella schneideri	1	1	1
Rhinella spinulosa	1	1	1
Rhinella vellardi	?	1	1
Rhinella veraguensis	0	?	1
Sabahphrynus maculatus	0	0	0
Schismaderma carens	1	1	1
Sclerophrys brauni	1	1	1^{inf}
Sclerophrys garmani	1	1	1^{inf}
Sclerophrys gracilipes	1	1	1
Sclerophrys gutturalis	1	1	1^{inf}
Sclerophrys kisoloensis	1	1	1^{inf}
Sclerophrys latifrons	1	1	1^{inf}
Sclerophrys lemairii	1	1	1^{inf}
Sclerophrys maculata	1	1	1
Sclerophrys mauritanica	1	1	1
Sclerophrys pantherina	1	1	1

Sclerophrys pardalis	1	1	1
Sclerophrys poweri	1	1	1^{inf}
Sclerophrys regularis	1	1	1
Sclerophrys steindachneri	1	1	1^{inf}
Sclerophrys tuberosa	1	1	1
Sclerophrys xeros	1	1	1
Strauchbufo raddei	1	1	1
Vandijkophrynus amatolicus	1	1	1^{inf}
Vandijkophrynus angusticeps	1	1	1
Vandijkophrynus gariepensis	1	1	1
Vandijkophrynus inyangae	1	1	1^{inf}
Vandijkophrynus robinsoni	0	1	1^{inf}
Werneria bambutensis	0	?	?
Werneria mertensiana	0	0^{inf}	0
Werneria tandyi	0	?	?
Wolterstorffina parvipalmata	0	0^{inf}	0
Xanthophryne koynayensis	1	1	1^{inf}

Bufonidae not included in Pyron (2014)			
**Species**	**Tympanic membrane**	**Tympanic annulus**	**Columella**
Adenomus kandianus	0	1	1
Altiphrynoides malcolmi	0	0	0
Altiphrynoides osgoodi	0	0	0
Amazophrynella amazonicola	0	?	?
Amazophrynella bokermanni	0	?	?
Amazophrynella javierbustamantei	0	?	?
Amazophrynella manaos	0	?	?
Amazophrynella matses	0	?	?
Amazophrynella vote	0	?	?
Anaxyrus compactilis	1	1	1[inf]
Anaxyrus kelloggi	?	1	1[inf]
Anaxyrus mexicanus	1	1	1[inf]
Ansonia echinata	0	1	1[inf]
Ansonia glandulosa	1	1	1[inf]
Ansonia jeetsukumarani	1	1	1[inf]
Ansonia latidisca	1	1	1
Ansonia latiffi	1	1	1[inf]
Ansonia lumut	1	1	1[inf]
Ansonia thinthinae	1	1	1[inf]
Ansonia vidua	1	1	1[inf]
Atelopus angelito	0	0	0
Atelopus ardila	0	0	0
Atelopus arthuri	0	0	0
Atelopus balios	0	0	0
Atelopus barbotini	0	?	1
Atelopus boulengeri	0	0	0
Atelopus carbonerensis	0	0	0
Atelopus carrikeri	0	0	0
Atelopus certus	0	0	0
Atelopus chirripoensis	0	0	?
Atelopus chrysocorallus	0	?	?
Atelopus coynei	0	0	0
Atelopus cruciger	0	0	0
Atelopus dimorphus	0	0	0
Atelopus ebenoides	0	0	0
Atelopus elegans	0	0	0
Atelopus epikeisthos	0	?	?
Atelopus eusebianus	0	0	0
Atelopus eusebiodiazi	0	0	?
Atelopus exiguus	0	0	0
Atelopus farci	0	0	0
Atelopus gigas	0	0	0
Atelopus glyphus	0	0	0

Atelopus guanujo	0	0	0
Atelopus guitarraensis	0	0	0
Atelopus hoogmoedi	0	?	1
Atelopus laetissimus	0	0	0
Atelopus limosus	0	0	?
Atelopus loettersi	0	0	0
Atelopus longibrachius	0	0	0
Atelopus lozanoi	0	0	0
Atelopus lynchi	0	0	0
Atelopus mandingues	0	0	0
Atelopus marinkellei	0	?	?
Atelopus mindoensis	0	0	0
Atelopus mittermeieri	0	0	?
Atelopus monohernandezii	0	0	0
Atelopus mucubajiensis	0	0	0
Atelopus muisca	0	0[inf]	0
Atelopus nahumae	0	0	0
Atelopus nepiozomus	0	?	?
Atelopus nicefori	0	0	0
Atelopus nocturnus	0	0	0
Atelopus onorei	0	0	?
Atelopus orcesi	0	0	0
Atelopus oxyrhynchus	0	0	0
Atelopus pachydermus	0	0	0
Atelopus palmatus	0	?	?
Atelopus pastuso	0	0	0
Atelopus patazensis	0	0	?
Atelopus pedimarmoratus	0	1	1[inf]
Atelopus petersi	0	0	0
Atelopus petriruizi	0	0	0
Atelopus pictiventris	0	?	?
Atelopus planispina	0	0	0
Atelopus podocarpus	0	0	0
Atelopus pyrodactylus	0	?	?
Atelopus reticulatus	0	?	?
Atelopus sanjosei	0	0	0
Atelopus sernai	0	0	0
Atelopus simulatus	0	0	0
Atelopus siranus	0	1	1[inf]
Atelopus sonsonensis	0	0	0
Atelopus sorianoi	0	0	0
Atelopus tamaense	0	?	?
Atelopus subornatus	0	0	0
Atelopus vogli	0	0	0
Atelopus walkeri	0	0	0
Blythophryne beryet	1	1	1

Bufo ailaoanus	0	?	?
Bufo pageoti	0	1	?
Bufoides meghalayana	0	?	?
Bufotes latastii	1	1	1[inf]
Bufotes luristanicus	1	1	1[inf]
Bufotes pseudoraddei	?	1	1[inf]
Bufotes surdus	0	?	?
Bufotes turanensis	1	1	1[inf]
Bufotes zugmayeri	1	1	1[inf]
Dendrophryniscus oreites	0	?	?
Dendrophryniscus organensis	0	?	?
Dendrophryniscus skuki	0	?	?
Dendrophryniscus stawiarskyi	0	?	?
Duttaphrynus beddomii	0	1	1[inf]
Duttaphrynus chandai	0	1	1[inf]
Duttaphrynus kiphirensis	1	1	1[inf]
Duttaphrynus kotagamai	1	1	1[inf]
Duttaphrynus mamitensis	1	1	1[inf]
Duttaphrynus manipurensis	1	1	1[inf]
Duttaphrynus microtympanum	?	1	1[inf]
Duttaphrynus mizoramensis	1	1	1[inf]
Duttaphrynus nagalandensis	1	1	1[inf]
Duttaphrynus noellerti	1	1	1[inf]
Duttaphrynus olivaceus	1	1[inf]	1[inf]
Duttaphrynus sumatranus	1	1	1[inf]
Duttaphrynus totol	1	1	1[inf]
Duttaphrynus valhallae	1	1	1[inf]
Duttaphrynus wokhaensis	1	1	1[inf]
Frostius erythrophthalmus	1	1[inf]	1[inf]
Frostius pernambucensis	1	1	1
Ghatophryne rubigina	1	1[inf]	1[inf]
Incilius aurarius	1	1	1[inf]
Incilius epioticus	0	0	0
Incilius guanacaste	0	?	?
Incilius holdridgei	0	0	0
Incilius intermedius	?	1	1[inf]
Incilius karenlipsae	1	1	1[inf]
Incilius majordomus	0	0	0
Incilius mccoyi	0	1	?
Incilius periglenes	0	0	0
Incilius peripatetes	0	0	0
Ingerophrynus claviger	1	1	1[inf]
Ingerophrynus gollum	1	1[inf]	1[inf]
Ingerophrynus kumquat	1	1[inf]	1[inf]
Ingerophrynus parvus	1	1	1[inf]
Ingerophrynus quadriporcatus	1	1	1[inf]

Laurentophryne parkeri	0	0	0
Leptophryne cruentata	0	1	1[inf]
Melanophryniscus admirabilis	0	?	?
Melanophryniscus alipioi	0	?	?
Melanophryniscus atroluteus	0	0	0
Melanophryniscus biancae	0	?	?
Melanophryniscus cambaraensis	0	?	?
Melanophryniscus cupreuscapularis	0	?	?
Melanophryniscus dorsalis	0	?	?
Melanophryniscus estebani	0	?	?
Melanophryniscus krauczuki	0	0	0
Melanophryniscus langonei	0	?	?
Melanophryniscus macrogranulosus	0	0	0
Melanophryniscus milanoi	0	?	?
Melanophryniscus montevidensis	0	0	0
Melanophryniscus moreirae	0	0	0
Melanophryniscus paraguayensis	0	?	?
Melanophryniscus peritus	0	?	?
Melanophryniscus sanmartini	0	?	?
Melanophryniscus setiba	0	0	0
Melanophryniscus simplex	0	?	?
Melanophryniscus spectabilis	0	?	?
Melanophryniscus tumifrons	0	0	0
Melanophryniscus vilavelhensis	0	?	?
Melanophryniscus xanthostomus	0	?	?
Mertensophryne howelli	0	0[inf]	0
Mertensophryne lonnbergi	0	0	0
Mertensophryne melanopleura	0	0	?
Mertensophryne mocquardi	0	?	?
Mertensophryne nairobiensis	0	?	?
Mertensophryne nyikae	0	?	?
Mertensophryne schmidti	0	0	0
Mertensophryne usambarae	0	0[inf]	0
Metaphryniscus sosai	0	0	?
Nannophryne apolobambica	0	0	?
Nannophryne corynetes	0	0	0
Nectophrynoides asperginis	0	?	1
Nectophrynoides cryptus	0&1	0	0
Nectophrynoides frontierei	0	0	0
Nectophrynoides laevis	0	?	?
Nectophrynoides laticeps	0	1	1[inf]
Nectophrynoides paulae	1	1	1[inf]
Nectophrynoides poyntoni	1	1	1
Nectophrynoides pseudotornieri	0	?	?
Nectophrynoides vestergaardi	1	1	1
Nectophrynoides wendyae	0	?	?

Oreophrynella cryptica	0	?	?
Oreophrynella dendronastes	0	?	?
Oreophrynella huberi	0	?	?
Oreophrynella nigra	0	?	?
Oreophrynella quelchii	0	0	0
Oreophrynella seegobini	0	?	?
Oreophrynella vasquezi	0	?	?
Oreophrynella weiassipuensis	0	?	?
Osornophryne angel	0	?	?
Osornophryne cofanorum	0	?	?
Osornophryne occidentalis	0	0	?
Osornophryne percrassa	0	0	0
Osornophryne simpsoni	0	0	0
Osornophryne talipes	0	?	?
Parapelophryne scalpta	0	1	1
Pedostibes kempi	0	?	?
Pelophryne albotaeniata	1	1	1
Pelophryne api	1	1	1[inf]
Pelophryne guentheri	1	1	1[inf]
Pelophryne lighti	1	1	1[inf]
Pelophryne linanitensis	1	1	1[inf]
Pelophryne murudensis	1	1	1[inf]
Pelophryne rhopophilia	1	1	1[inf]
Pelophryne saravacensis	0	1	1[inf]
Peltophryne cataulaciceps	0	1	1[inf]
Peltophryne florentinoi	1	1	1[inf]
Peltophryne fluviatica	?	1	1[inf]
Peltophryne fracta	?	1	1[inf]
Poyntonophrynus beiranus	0	1	1[inf]
Poyntonophrynus grandisonae	1	1	1
Poyntonophrynus hoeschi	0	1	1[inf]
Poyntonophrynus kavangensis	1	1	1[inf]
Poyntonophrynus lughensis	1	1[inf]	1[inf]
Poyntonophrynus parkeri	?	1	1[inf]
Pseudobufo subasper	1	1	1
Pedostibes everetti	1	1	1[inf]
Rhaebo andinophrynoides	1	1	1[inf]
Rhaebo atelopoides	1	1	1
Rhaebo blombergi	1	1	1
Rhaebo caeruleostictus	0	1	1
Rhaebo colomai	0	1	1
Rhaebo haematiticus	1	1	1
Rhaebo hypomelas	1	1	1[inf]
Rhaebo lynchi	1	1[inf]	1[inf]
Rhaebo olallai	0	1	1
Rhinella abei	1	1	1[inf]

Rhinella achalensis	1	1	1
Rhinella acrolopha	0	0	0
Rhinella acutirostris	1	1	1[inf]
Rhinella alata	1	1	1[inf]
Rhinella amabilis	1	1	1
Rhinella arborescandens	0	0	0
Rhinella azarai	1	1	1[inf]
Rhinella bergi	1	1	1[inf]
Rhinella bernardoi	1	1	1[inf]
Rhinella casconi	1	1	1[inf]
Rhinella centralis	1	1	1[inf]
Rhinella ceratophrys	1	1	1[inf]
Rhinella cerradensis	1	1	1[inf]
Rhinella chrysophora	0	1	1
Rhinella cristinae	0	0	0
Rhinella crucifer	1	1	1
Rhinella diptycha	?	1	1[inf]
Rhinella dorbignyi	0&1	1	1[inf]
Rhinella fernandezae	0&1	1	1
Rhinella fissipes	0	?	?
Rhinella gallardoi	1	1	1[inf]
Rhinella gildae	1	1	1[inf]
Rhinella gnustae	1	1	1[inf]
Rhinella henseli	1	1	1[inf]
Rhinella hoogmoedi	1	1	1[inf]
Rhinella inca	1	1	?
Rhinella inopina	1	1	1[inf]
Rhinella iserni	0	0	0
Rhinella jimi	1	1	1[inf]
Rhinella justinianoi	0	?	?
Rhinella leptoscelis	1	1	1[inf]
Rhinella lescurei	1	1	1[inf]
Rhinella lindae	0&1	0&1	1
Rhinella magnussoni	1	1	1[inf]
Rhinella major	1	1	1
Rhinella martyi	1	1	1[inf]
Rhinella merianae	1	1	1[inf]
Rhinella mirandaribeiroi	1	1	1
Rhinella multiverrucosa	1	1	1
Rhinella nattereri	1	1	1[inf]
Rhinella nicefori	0	0	0
Rhinella paraguas	0	0	1
Rhinella paraguayensis	0	1	1[inf]
Rhinella proboscidea	1	1	1
Rhinella pygmaea	1	1	1
Rhinella quechua	0	?	?

Rhinella roqueana	1	1[inf]	1[inf]
Rhinella rubescens	1	1	1
Rhinella rubropunctata	1	1	1
Rhinella ruizi	0	0	0
Rhinella rumbolli	1	1	1[inf]
Rhinella scitula	1	1	1[inf]
Rhinella sclerocephala	1	1	1[inf]
Rhinella sebbeni	1	1	1[inf]
Rhinella stanlaii	1	1	1
Rhinella sternosignata	1	1	1
Rhinella tacana	1	1	1[inf]
Rhinella tenrec	0	0	0
Rhinella truebae	1	1[inf]	1[inf]
Rhinella veredas	1	1	1[inf]
Rhinella yanachaga	1	1	1
Rhinella yunga	0	?	?
Sclerophrys arabica	1	1	1[inf]
Sclerophrys asmarae	1	1	1[inf]
Sclerophrys blanfordii	?	1	1[inf]
Sclerophrys buchneri	1	1	1[inf]
Sclerophrys capensis	1	1	1[inf]
Sclerophrys channingi	1	1	1[inf]
Sclerophrys chudeaui	0	1	1[inf]
Sclerophrys cristiglans	1	1	1[inf]
Sclerophrys danielae	1	1	1[inf]
Sclerophrys dodsoni	1	1	1[inf]
Sclerophrys fuliginata	1	1	1[inf]
Sclerophrys funerea	?	?	1
Sclerophrys kassasii	1	1	1[inf]
Sclerophrys kerinyagae	1	1	1[inf]
Sclerophrys pentoni	?	1	1[inf]
Sclerophrys perreti	1	1	1[inf]
Sclerophrys reesi	1	1	1[inf]
Sclerophrys superciliaris	1	1	1[inf]
Sclerophrys taiensis	1	1	1[inf]
Sclerophrys urunguensis	1	1	1[inf]
Sclerophrys vittata	1	1	1[inf]
Truebella skoptes	0	0	0
Truebella tothastes	0	0	0
Werneria iboundji	0	?	?
Werneria preussi	0	0	0
Werneria submontana	0	?	?
Wolterstorffina mirei	0	0	0
Xanthophryne tigerina	0	1	1[inf]

Anura included in Pyron (2014)				
**Family**	**Species**	**Tympanic membrane**	**Tympanic annulus**	**Columella**
Allophrynidae	*Allophryne ruthveni*	1	1	1
Alsodidae	*Alsodes barrioi*	0	0	0
	Alsodes coppingeri	0	0	0
	Alsodes gargola	0	0	0
	Alsodes nodosus	0	1	1
	Alsodes pehuenche	0[inf]	0[inf]	0
	Alsodes tumultuosus	0	0	0
	Alsodes valdiviensis	0	0	0
	Alsodes vanzolinii	0[inf]	0[inf]	0
	Eupsophus calcaratus	?	?	1
	Eupsophus contulmoensis	1	1[inf]	1[inf]
	Eupsophus emiliopugini	?	1	1[inf]
	Eupsophus roseus	1	1	1
	Eupsophus septentrionalis	1	1[inf]	1
	Eupsophus vertebralis	1	1	1
	Limnomedusa macroglossa	0	1	1
Alytidae	*Alytes cisternasii*	?	1	1[inf]
	Alytes obstetricans	1	1	1
	Discoglossus montalentii	?	1	1[inf]
	Discoglossus pictus	0	?	1
	Discoglossus sardus	?	1	1
	Discoglossus scovazzi	?	1	1[inf]
Aromobatidae	*Allobates brunneus*	1	1	1[inf]
	Allobates femoralis	?	1	1
	Allobates granti	?	1	1[inf]
	Allobates insperatus	?	1	1
	Allobates juanii	?	1	1
	Allobates kingsburyi	?	1	1
	Allobates talamancae	0	1	1
	Allobates undulatus	?	1	1
	Anomaloglossus baebatrachus	1	1	1[inf]
	Anomaloglossus beebei	?	1	1[inf]
	Anomaloglossus kaiei	0	1	?
	Aromobates nocturnus	?	1	1
	Mannophryne collaris	?	1	1
	Mannophryne herminae	1	1	1
	Mannophryne trinitatis	?	1	1
	Rheobates palmatus	?	1	1[inf]
Arthroleptidae	*Arthroleptis adolfifriederici*	?	?	1
	Arthroleptis affinis	1	1	1[inf]
	Arthroleptis poecilonotus	?	1	1
	Arthroleptis stenodactylus	0&1	1	1

Arthroleptidae (cont.)	*Arthroleptis taeniatus*	1	1	1
	Arthroleptis reichi	?	?	1
	Arthroleptis schubotzi	?	?	1
	Arthroleptis tanneri	1	1	1^{inf}
	Arthroleptis variabilis	0	1	1
	Arthroleptis wahlbergii	?	1	1^{inf}
	Arthroleptis xenodactyloides	1	1	1
	Astylosternus batesii	0	1	1^{inf}
	Astylosternus diadematus	1	1	1
	Cardioglossa gracilis	1	1	1
	Cardioglossa leucomystax	1	1	1^{inf}
	Cardioglossa manengouba	0	1	1^{inf}
	Cardioglossa oreas	0	1	1^{inf}
	Leptodactylodon bicolor	0	?	?
	Leptopelis argenteus	?	1	1^{inf}
	Leptopelis bocagii	0	1	1
	Leptopelis calcaratus	?	1	1
	Leptopelis kivuensis	1	1	1^{inf}
	Leptopelis millsoni	?	1	1
	Leptopelis vermiculatus	1	1	1
	Nyctibates corrugatus	0	1	1
	Scotobleps gabonicus	1	1	1
	Trichobatrachus robustus	0	1	1
Ascaphidae	*Ascaphus truei*	0	0	0
Batrachylidae	*Atelognathus patagonicus*	0^{inf}	0^{inf}	0
	Atelognathus salai	0^{inf}	0^{inf}	0
	Batrachyla leptopus	1	1	1
	Batrachyla taeniata	?	?	1
	Hylorina sylvatica	1	1	1
Bombinatoridae	*Barbourula busuangensis*	0	?	1
	Bombina bombina	0	0	0
	Bombina maxima	?	?	1^{*}
	Bombina orientalis	0	0	1^{*}
	Bombina pachypus	?	?	1^{*}
	Bombina variegata	0	0	1^{*}
Brachycephalidae	*Brachycephalus alipioi*	0	0	?
	Brachycephalus brunneus	0	0	0
	Brachycephalus didactylus	0	0	0
	Brachycephalus ephippium	0	0	0
	Brachycephalus ferruginus	0	0	0
	Brachycephalus hermogenesi	0	0	0
	Brachycephalus izeckshoni	0	0	0
	Brachycephalus nodoterga	0	0	0
	Brachycephalus pernix	0	0	0
	Brachycephalus pitanga	0	0	?

Brachycephalidae (cont.)	*Brachycephalus pombali*	0	0	0
	Brachycephalus vertebralis	0	0	0
	Ischnocnema bolbodactyla	1	1	1[inf]
	Ischnocnema erythromera	1	1	1[inf]
	Ischnocnema guentheri	1	1	1
	Ischnocnema hoehnei	1	1	1[inf]
	Ischnocnema holti	0	1	1[inf]
	Ischnocnema izecksohni	1	1	1[inf]
	Ischnocnema juipoca	1	1	1[inf]
	Ischnocnema lactea	1	1	1[inf]
	Ischnocnema nasuta	1	1	1
	Ischnocnema octavioi	1	1	1
	Ischnocnema oea	1	1	1[inf]
	Ischnocnema parva	1	1	1
	Ischnocnema sambaqui	1	1	1[inf]
	Ischnocnema spanios	0	?	?
	Ischnocnema venancioi	1	1	1
	Ischnocnema verrucosa	1	1	1[inf]
Brevicipitidae	*Balebreviceps hillmani*	0	0	0
	Breviceps fuscus	0	?	1
	Breviceps mossambicus	0	1	1
	Callulina kreffti	1	1	1
	Probreviceps loveridgei	0	1	1
	Probreviceps macrodactylus	0	1	1
	Probreviceps rungwensis	0	1	1
	Probreviceps uluguruensis	0	1	1
	Spelaeophryne methneri	?	1	1
Calyptocephalellidae	*Calyptocephalella gayi*	1	1	1
	Telmatobufo bullocki	0	0	0
	Telmatobufo venustus	0	0	0
Centrolenidae	*Celsiella revocata*	?	1	1[inf]
	Celsiella vozmedianoi	?	1	1[inf]
	Centrolene altitudinale	1	1	1[inf]
	Centrolene bacatum	1	1	1[inf]
	Centrolene ballux	?	1	1[inf]
	Centrolene buckleyi	0	1	1[inf]
	Centrolene condor	0	1	1[inf]
	Centrolene daidaleum	1	1	1[inf]
	Centrolene geckoideum	1	1	1[inf]
	Centrolene heloderma	?	1	1[inf]
	Centrolene peristictum	1	1	1[inf]
	Centrolene pipilatum	1	1	1[inf]
	Centrolene venezuelense	?	1	1[inf]
	Cochranella euknemos	1	1	1
	Cochranella litoralis	?	1	1[inf]

Centrolenidae (cont.)	*Cochranella nola*	?	1	1^{inf}
	Espadarana andina	1	1	1^{inf}
	Espadarana callistomma	1	1	1
	Espadarana prosoblepon	1	1	1^{inf}
	Hyalinobatrachium aureoguttatum	1	1	1
	Hyalinobatrachium bergeri	0	1	1^{inf}
	Hyalinobatrachium colymbiphyllum	0	1	1
	Hyalinobatrachium eccentricum	?	1	1^{inf}
	Hyalinobatrachium fleischmanni	0&1	1	1
	Hyalinobatrachium iaspidiense	0	1	1^{inf}
	Hyalinobatrachium ibama	?	1	1^{inf}
	Hyalinobatrachium ignioculis	?	1	1^{inf}
	Hyalinobatrachium mondolfii	0	1	1^{inf}
	Hyalinobatrachium munozorum	0	1	1^{inf}
	Hyalinobatrachium orocostale	0	1	1^{inf}
	Hyalinobatrachium talamancae	0	1	1^{inf}
	Hyalinobatrachium taylori	1	1	1^{inf}
	Ikakogi tayrona	0	1	1
	Nymphargus bejaranoi	1	1	1^{inf}
	Nymphargus cochranae	1	1	1^{inf}
	Nymphargus grandisonae	?	1	1^{inf}
	Nymphargus griffithsi	?	1	1^{inf}
	Nymphargus megacheirus	?	1	1^{inf}
	Nymphargus posadae	0	1	1^{inf}
	Nymphargus siren	1	1	1^{inf}
	Nymphargus wileyi	1	1	1^{inf}
	Rulyrana adiazeta	?	1	1^{inf}
	Rulyrana flavopunctata	?	1	1^{inf}
	Sachatamia albomaculata	1	1	1^{inf}
	Sachatamia ilex	0	1	1^{inf}
	Teratohyla midas	1	1	1^{inf}
	Teratohyla pulverata	1	1	1^{inf}
	Vitreorana antisthenesi	1	1	1^{inf}
	Vitreorana gorzulae	0	1	1^{inf}
	Vitreorana ritae	1	1	1^{inf}
	Vitreorana uranoscopa	1	1	1
Ceratobatrachidae	*Alcalus baluensis*	?	1	1^{inf}
	Cornufer guentheri	?	1	1
	Cornufer guppyi	?	1	1
	Cornufer papuensis	1	1	1^{inf}

Ceratobatrachidae (cont.)	*Cornufer vertebralis*	0	1	1
	Platymantis corrugatus	1	1	1
	Platymantis dorsalis	?	1	1
	Platymantis hazelae	?	1	1
Ceratophryidae	*Ceratophrys cornuta*	0	1	1^{inf}
	Ceratophrys ornata	1	1	1
	Chacophrys pierottii	1	1	1
	Lepidobatrachus laevis	1	1	1
Conrauidae	*Conraua crassipes*	0	1	1
	Conraua goliath	?	1	1
Craugastoridae	*Barycholos pulcher*	1	1	1
	Bryophryne cophites	0	0	0
	Ceuthomantis smaragdinus	1	1	1
	Craugastor alfredi	1	1	1^{inf}
	Craugastor andi	1	1	1^{inf}
	Craugastor angelicus	1	1	1^{inf}
	Craugastor augusti	1	1	1
	Craugastor bocourti	1	1	1^{inf}
	Craugastor bransfordii	1	1	1^{inf}
	Craugastor crassidigitus	1	1	1^{inf}
	Craugastor cuaquero	1	1	1^{inf}
	Craugastor daryi	1	1	1^{inf}
	Craugastor emcelae	1	1	1^{inf}
	Craugastor fitzingeri	1	1	1
	Craugastor fleischmanni	1	1	1^{inf}
	Craugastor laticeps	1	1	1^{inf}
	Craugastor lineatus	1	1	1^{inf}
	Craugastor loki	1	1	1^{inf}
	Craugastor longirostris	1	1	1
	Craugastor megacephalus	1	1	1^{inf}
	Craugastor melanostictus	1	1	1^{inf}
	Craugastor mexicanus	1	1	1^{inf}
	Craugastor montanus	1	1	1^{inf}
	Craugastor obesus	1	1	1^{inf}
	Craugastor podiciferus	1	1	1
	Craugastor punctariolus	1	1	1^{inf}
	Craugastor pygmaeus	1	1	1^{inf}
	Craugastor raniformis	1	1	1^{inf}
	Craugastor ranoides	1	1	1^{inf}
	Craugastor rhodopis	1	1	1
	Craugastor rugulosus	1	1	1^{inf}
	Craugastor rupinius	1	1	1^{inf}
	Craugastor sandersoni	1	1	1^{inf}
	Craugastor spatulatus	1	1	1^{inf}
	Craugastor stuarti	1	1	1^{inf}

	Craugastor tabasarae	1	1	1^{inf}
	Craugastor talamancae	1	1	1^{inf}
	Craugastor tarahumaraensis	1	1	1^{inf}
	Craugastor uno	1	1	1^{inf}
	Euparkerella brasiliensis	0	0	0
	Haddadus binotatus	1	1	1
	Holoaden bradei	0	0	0
	Holoaden luederwaldti	0	0	?
	Hypodactylus brunneus	1	1	1^{inf}
	Hypodactylus dolops	1	1	1
	Hypodactylus elassodiscus	1	1	1^{inf}
	Hypodactylus peraccai	1	1	1^{inf}
	Lynchius flavomaculatus	1	1	1
	Lynchius nebulanastes	1	1	1
	Lynchius parkeri	0	1	1^{inf}
	Lynchius simmonsi	1	1	1^{inf}
	Noblella heyeri	1	1^{inf}	1
	Noblella lochites	1	1	1^{inf}
	Noblella myrmecoides	1	1	1
	Noblella peruviana	1	1	1
	Oreobates barituensis	1	1	1^{inf}
	Oreobates choristolemma	1	1	1^{inf}
Craugastoridae (cont.)	*Oreobates cruralis*	1	1	1^{inf}
	Oreobates discoidalis	1	1	1
	Oreobates granulosus	1	1	1^{inf}
	Oreobates heterodactylus	1	1	1^{inf}
	Oreobates ibischi	1	1	1^{inf}
	Oreobates lehri	1	1	1^{inf}
	Oreobates madidi	1	1	1^{inf}
	Oreobates pereger	1	1	1^{inf}
	Oreobates quixensis	1	1	1
	Oreobates sanctaecrucis	1	1	1^{inf}
	Oreobates sanderi	1	1	1^{inf}
	Oreobates saxatilis	1	1	1^{inf}
	Phrynopus barthlenae	0	0	?
	Phrynopus bracki	0	0	?
	Phrynopus bufoides	0	0	?
	Phrynopus horstpauli	0	0	?
	Phrynopus juninensis	0	0	0
	Phrynopus kauneorum	0	0	?
	Phrynopus pesantesi	0	0	?
	Phrynopus tautzorum	0	0	?
	Pristimantis acatallelus	0	1	1^{inf}
	Pristimantis acerus	1	1	1^{inf}
	Pristimantis achatinus	1	1	1

	Pristimantis actites	1	1	1[inf]
	Pristimantis acuminatus	0	1	1[inf]
	Pristimantis altae	1	1	1[inf]
	Pristimantis altamazonicus	0	1	1
	Pristimantis angustilineatus	1	1	1[inf]
	Pristimantis aniptopalmatus	?	1	1[inf]
	Pristimantis appendiculatus	1	1	1[inf]
	Pristimantis ardalonychus	0	1	1[inf]
	Pristimantis bipunctatus	?	1	1[inf]
	Pristimantis bogotensis	1	1	1
	Pristimantis brevifrons	1	1	1[inf]
	Pristimantis bromeliaceus	1	1	1[inf]
	Pristimantis buccinator	1	1	1[inf]
	Pristimantis buckleyi	1	1	1[inf]
	Pristimantis cajamarcensis	1	1	1[inf]
	Pristimantis calcaratus	1	1	1[inf]
	Pristimantis calcarulatus	1	1	1[inf]
	Pristimantis caprifer	1	1	1[inf]
	Pristimantis caryophyllaceus	?	?	1
	Pristimantis celator	?	1	1[inf]
	Pristimantis cerasinus	1	1[inf]	1
	Pristimantis ceuthospilus	1	1	1[inf]
Craugastoridae (cont.)	*Pristimantis chalceus*	0	1	1
	Pristimantis chiastonotus	1	1	1[inf]
	Pristimantis chloronotus	1	1	1
	Pristimantis citriogaster	1	1	1[inf]
	Pristimantis colomai	1	1	1[inf]
	Pristimantis condor	1	1	1[inf]
	Pristimantis conspicillatus	1	1	1
	Pristimantis cremnobates	1	1	1[inf]
	Pristimantis crenunguis	1	1	1[inf]
	Pristimantis croceoinguinis	0	1	1
	Pristimantis crucifer	1	1	1[inf]
	Pristimantis cruentus	0	1	1
	Pristimantis cryophilius	1	1	1[inf]
	Pristimantis curtipes	0	1	1
	Pristimantis danae	1	1	1[inf]
	Pristimantis devillei	1	1	1
	Pristimantis diadematus	1	1	1[inf]
	Pristimantis dissimulatus	1	1	1[inf]
	Pristimantis duellmani	0	0	0
	Pristimantis eriphus	1	1	1[inf]
	Pristimantis erythropleura	0	1	1[inf]
	Pristimantis euphronides	1	1	1[inf]
	Pristimantis fenestratus	1	1	1[inf]

Craugastoridae (cont.)	*Pristimantis frater*	1	1	1
	Pristimantis gaigei	1	1	1
	Pristimantis galdi	1	1	1
	Pristimantis gentry	0	1	1[inf]
	Pristimantis glandulosus	1	1	1[inf]
	Pristimantis gutturalis	1	1	1[inf]
	Pristimantis hectus	1	1	1[inf]
	Pristimantis imitatrix	0	0	?
	Pristimantis inguinalis	?	1	1[inf]
	Pristimantis inusitatus	1	1	1[inf]
	Pristimantis juanchoi	1	1	1[inf]
	Pristimantis jubatus	1	1	1[inf]
	Pristimantis kelephus	1	1	1[inf]
	Pristimantis koehleri	1	1	1[inf]
	Pristimantis labiosus	1	1	1[inf]
	Pristimantis lanthanites	1	1	1[inf]
	Pristimantis latidiscus	1	1	1[inf]
	Pristimantis leoni	0	1	1[inf]
	Pristimantis librarius	?	1	1[inf]
	Pristimantis lirellus	0	0	?
	Pristimantis llojsintuta	1	1	1[inf]
	Pristimantis luteolateralis	1	1	1[inf]
	Pristimantis lymani	1	1	1[inf]
	Pristimantis malkini	1	1	1[inf]
	Pristimantis marmoratus	1	1	1[inf]
	Pristimantis martiae	0	1	1[inf]
	Pristimantis melanogaster	0	1	1[inf]
	Pristimantis miyatai	0	1	1[inf]
	Pristimantis moro	0	?	?
	Pristimantis museosus	1	1	1[inf]
	Pristimantis myops	1	1	1[inf]
	Pristimantis nervicus	1	1	1[inf]
	Pristimantis nyctophylax	?	1	1[inf]
	Pristimantis ockendeni	1	1	1[inf]
	Pristimantis ocreatus	0	1	1[inf]
	Pristimantis orcesi	1	1	1[inf]
	Pristimantis orestes	1	1	1[inf]
	Pristimantis paisa	0	1	1[inf]
	Pristimantis palmeri	1	1	1
	Pristimantis pardalis	1	1	1[inf]
	Pristimantis parvillus	1	1	1[inf]
	Pristimantis peruvianus	1	1	1[inf]
	Pristimantis petrobardus	1	1	1[inf]
	Pristimantis phoxocephalus	1	1	1[inf]
	Pristimantis pirrensis	1	1	1[inf]

Craugastoridae (cont.)	*Pristimantis platydactylus*	1	1	1[inf]
	Pristimantis prolatus	1	1	1[inf]
	Pristimantis ptochus	?	1	1[inf]
	Pristimantis pulvinatus	1	1	1[inf]
	Pristimantis pycnodermis	1	1	1[inf]
	Pristimantis pyrrhomerus	1	1	1[inf]
	Pristimantis quantus	1	1	1[inf]
	Pristimantis quaquaversus	0	0	?
	Pristimantis quinquagesimus	1	1	1[inf]
	Pristimantis ramagii	1	1	1[inf]
	Pristimantis reichlei	1	1	1[inf]
	Pristimantis rhabdocnemus	0	0	?
	Pristimantis rhabdolaemus	1	1	1[inf]
	Pristimantis rhodoplichus	1	1	1[inf]
	Pristimantis ridens	1	1	1
	Pristimantis riveti	1	1	1[inf]
	Pristimantis rozei	1	1	1[inf]
	Pristimantis sagittulus	1	1	1[inf]
	Pristimantis samaipatae	1	1	1[inf]
	Pristimantis savagei	1	1	1[inf]
	Pristimantis schultei	1	1	1[inf]
	Pristimantis shrevei	1	1	1[inf]
	Pristimantis simonbolivari	1	1	1[inf]
	Pristimantis simonsii	0	0	0
	Pristimantis skydmainos	1	1	1[inf]
	Pristimantis spinosus	1	1	1[inf]
	Pristimantis stictogaster	1	1	1[inf]
	Pristimantis subsigillatus	1	1	1[inf]
	Pristimantis suetus	1	1	1[inf]
	Pristimantis supernatis	1	1	1[inf]
	Pristimantis surdus	0	0	0
	Pristimantis taeniatus	1	1	1[inf]
	Pristimantis terraebolivaris	1	1	1[inf]
	Pristimantis thectopternus	1	1	1[inf]
	Pristimantis thymalopsoides	1	1	1[inf]
	Pristimantis thymelensis	0	1	1[inf]
	Pristimantis toftae	1	1	1[inf]
	Pristimantis truebae	0	1	1[inf]
	Pristimantis unistrigatus	1	1	1
	Pristimantis urichi	1	1	1[inf]
	Pristimantis ventrimarmoratus	0	1	1[inf]
	Pristimantis verecundus	1	1	1[inf]
	Pristimantis versicolor	1	1	1[inf]
	Pristimantis vertebralis	1	1	1[inf]
	Pristimantis viejas	1	1	1[inf]

Craugastoridae (cont.)	*Pristimantis walkeri*	1	1	1[inf]
	Pristimantis wiensi	?	1	1[inf]
	Pristimantis wnigrum	1	1	1
	Pristimantis zophus	1	1	1[inf]
	Psychrophrynella iatamasi	0	?	?
	Psychrophrynella wettsteini	1	1[inf]	1
	Strabomantis anomalus	1	1	1[inf]
	Strabomantis biporcatus	1	1	1[inf]
	Strabomantis bufoniformis	1	1	1
	Strabomantis necerus	1	1	1
	Strabomantis sulcatus	1	1	1
	Yunganastes ashkapara	1	1	1[inf]
	Yunganastes bisignatus	1	1	1[inf]
	Yunganastes fraudator	1	1	1[inf]
	Yunganastes mercedesae	1	1	1[inf]
	Yunganastes pluvicanorus	1	1	1[inf]
Cycloramphidae	*Cycloramphus boraceiensis*	0	?	?
	Cycloramphus eleutherodactylus	0	1	1
	Thoropa miliaris	?	1	1
	Thoropa taophora	1	1	1[inf]
	Zachaenus parvulus	0	1	1
Dendrobatidae	*Adelphobates castaneoticus*	?	1	1
	Adelphobates galactonotus	?	1	1
	Ameerega bassleri	?	1	1
	Ameerega bilinguis	1	1	1
	Ameerega hahneli	?	1	1
	Ameerega macero	?	1	1[inf]
	Ameerega parvula	?	1	1[inf]
	Ameerega petersi	?	1	1
	Ameerega picta	?	1	1
	Ameerega silverstonei	?	1	1
	Ameerega simulans	1	1	1
	Ameerega trivittata	?	1	1
	Andinobates claudiae	?	1	1
	Andinobates fulguritus	?	1	1
	Colostethus fugax	?	1	1[inf]
	Colostethus inguinalis	1	1	1
	Colostethus latinasus	?	1	1[inf]
	Colostethus panamensis	?	1	1
	Colostethus pratti	?	1	1
	Dendrobates auratus	1	1	1
	Dendrobates tinctorius	?	1	1
	Dendrobates truncatus	?	1	1
	Epipedobates anthonyi	?	?	1

Dendrobatidae (cont.)	*Epipedobates boulengeri*	?	1	1
	Epipedobates espinosai	?	1	1
	Excidobates captivus	?	1	1[inf]
	Excidobates mysteriosus	?	1	1[inf]
	Hyloxalus awa	?	1	1
	Hyloxalus bocagei	1	1	1
	Hyloxalus chlorocraspedus	?	1	1[inf]
	Hyloxalus elachyhistus	?	1	1
	Hyloxalus nexipus	?	1	1[inf]
	Hyloxalus pulchellus	?	1	1
	Hyloxalus sauli	?	1	1
	Hyloxalus subpunctatus	1	1	1
	Hyloxalus sylvaticus	?	1	1
	Hyloxalus vertebralis	?	1	1
	Minyobates steyermarki	?	1	1
	Oophaga arborea	?	1	1
	Oophaga granulifera	?	1	1
	Oophaga histrionica	?	1	1
	Oophaga lehmanni	?	1	1
	Oophaga pumilio	0	1	1
	Oophaga speciosa	?	1	1
	Oophaga sylvatica	?	1	1
	Oophaga vicentei	?	1	1
	Phyllobates aurotaenia	?	1	1
	Phyllobates bicolor	?	1	1
	Phyllobates lugubris	?	1	1
	Phyllobates terribilis	?	1	1
	Phyllobates vittatus	?	1	1
	Ranitomeya amazonica	?	1	1[int]
	Ranitomeya imitator	?	1	1
	Ranitomeya reticulata	?	1	1
	Ranitomeya ventrimaculata	1	1	1
	Silverstoneia flotator	?	1	1
	Silverstoneia nubicola	?	1	1
Dicroglossidae	*Euphlyctis cyanophlyctis*	0	1	1
	Euphlyctis hexadactylus	?	1	1[inf]
	Fejervarya cancrivora	1	1	1[inf]
	Fejervarya caperata	0	1	1[inf]
	Fejervarya granosa	0	1	1[inf]
	Fejervarya kudremukhensis	0	1	1[inf]
	Fejervarya iskandari	?	1	1[inf]
	Fejervarya limnocharis	?	1	1[inf]
	Fejervarya mudduraja	0	1	1[inf]
	Fejervarya rufescens	?	1	1[inf]
	Fejervarya sahyadris	?	1	1

Dicroglossidae (cont.)	*Hoplobatrachus occipitalis*	1	1	1
	Hoplobatrachus rugulosus	?	1	1[inf]
	Hoplobatrachus tigerinus	1	1	1
	Ingerana tenasserimensis	?	1	1[inf]
	Limnonectes asperatus	?	1	1[inf]
	Limnonectes blythii	0	1	1
	Limnonectes dabanus	?	1	1[inf]
	Limnonectes doriae	?	1	1[inf]
	Limnonectes finchi	1	1	1[inf]
	Limnonectes hascheanus	1	1	1[inf]
	Limnonectes ibanorum	?	1	1[inf]
	Limnonectes kuhlii	0	1	1
	Limnonectes laticeps	0	1	1[inf]
	Limnonectes leporinus	1	1	1[inf]
	Limnonectes leytensis	1	1	1[inf]
	Limnonectes macrodon	?	1	1[inf]
	Limnonectes magnus	?	1	1[inf]
	Limnonectes malesianus	1	1	1[inf]
	Limnonectes microdiscus	?	1	1[inf]
	Limnonectes microtympanum	?	1	1[inf]
	Limnonectes modestus	?	1	1[inf]
	Limnonectes palavanensis	1	1	1[inf]
	Limnonectes paramacrodon	1	1	1[inf]
	Limnonectes plicatellus	1	1	1[inf]
	Limnonectes woodworthi	?	1	1[inf]
	Nannophrys ceylonensis	1	1	1
	Nannophrys marmorata	?	?	1
	Nanorana aenea	?	1	1[inf]
	Nanorana arnoldi	?	1	1[inf]
	Nanorana liebigii	0	1	1
	Nanorana parkeri	0	0	0
	Nanorana pleski	0	1	1
	Nanorana quadranus	?	1	1[inf]
	Nanorana ventripunctata	0	0[inf]	0
	Nanorana yunnanensis	0	1	1[inf]
	Occidozyga baluensis	?	1	1[inf]
	Occidozyga laevis	0&1	1	1
	Occidozyga lima	0	?	1
	Quasipaa delacouri	?	1	1[inf]
	Quasipaa shini	0	1	1[inf]
	Quasipaa spinosa	?	?	1
	Sphaerotheca breviceps	?	1	1[inf]
	Sphaerotheca dobsonii	?	1	1[inf]
Eleutherodactylidae	*Adelophryne adiastola*	1	1	1[inf]
	Adelophryne baturitensis	1	1	1[inf]

Eleutherodactylidae (cont.)	*Adelophryne gutturosa*	1	1	1[inf]
	Adelophryne maranguapensis	1	1	1[inf]
	Adelophryne pachydactyla	1	1	1[inf]
	Adelophryne patamona	1	1	1[inf]
	Diasporus diastema	1	1	1
	Diasporus hylaeformis	1	1	1[inf]
	Diasporus vocator	1	1	1[inf]
	Eleutherodactylus abbotti	1	1	1
	Eleutherodactylus acmonis	1	1	1[inf]
	Eleutherodactylus adelus	1	1	1[inf]
	Eleutherodactylus albipes	1	1	1[inf]
	Eleutherodactylus alcoae	1	1	1[inf]
	Eleutherodactylus alticola	1	1	1[inf]
	Eleutherodactylus amadeus	1	1	1[inf]
	Eleutherodactylus amplinympha	1	1	1[inf]
	Eleutherodactylus andrewsi	1	1	1[inf]
	Eleutherodactylus antillensis	1	1	1
	Eleutherodactylus apostates	1	1	1[inf]
	Eleutherodactylus armstrongi	1	1	1
	Eleutherodactylus atkinsi	1	1	1
	Eleutherodactylus audanti	1	1	1
	Eleutherodactylus auriculatoides	1	1	1
	Eleutherodactylus auriculatus	1	1	1[inf]
	Eleutherodactylus bakeri	1	1	1[inf]
	Eleutherodactylus barlagnei	1	1	1
	Eleutherodactylus bartonsmithi	1	1	1[inf]
	Eleutherodactylus blairhedgesi	1	1	1[inf]
	Eleutherodactylus bothroboans	1	1	1[inf]
	Eleutherodactylus bresslerae	1	1	1[inf]
	Eleutherodactylus brevirostris	1	1	1[inf]
	Eleutherodactylus brittoni	1	1	1[inf]
	Eleutherodactylus caribe	1	1	1[inf]
	Eleutherodactylus casparii	1	1	1[inf]
	Eleutherodactylus cavernicola	1	1	1[inf]
	Eleutherodactylus chlorophenax	1	1	1[inf]
	Eleutherodactylus cochranae	1	1	1
	Eleutherodactylus cooki	1	1	1[inf]
	Eleutherodactylus coqui	1	1	1
	Eleutherodactylus corona	1	1	1[inf]
	Eleutherodactylus counouspeus	1	1	1[inf]
	Eleutherodactylus cubanus	1	1	1[inf]
	Eleutherodactylus cundalli	1	1	1

Eleutherodactylidae (cont.)	*Eleutherodactylus cuneatus*	1	1	1[inf]
	Eleutherodactylus darlingtoni	1	1	1[inf]
	Eleutherodactylus dimidiatus	1	1	1[inf]
	Eleutherodactylus dolomedes	1	1	1[inf]
	Eleutherodactylus eileenae	1	1	1[inf]
	Eleutherodactylus emiliae	1	1	1[inf]
	Eleutherodactylus eneidae	1	1	1
	Eleutherodactylus etheridgei	1	1	1[inf]
	Eleutherodactylus eunaster	1	1	1[inf]
	Eleutherodactylus flavescens	1	1	1[inf]
	Eleutherodactylus fowleri	1	1	1[inf]
	Eleutherodactylus furcyensis	1	1	1
	Eleutherodactylus fuscus	1	1	1[inf]
	Eleutherodactylus glamyrus	1	1	1[inf]
	Eleutherodactylus glandulifer	1	1	1[inf]
	Eleutherodactylus glanduliferoides	1	1	1[inf]
	Eleutherodactylus glaphycompus	1	1	1[inf]
	Eleutherodactylus glaucoreius	1	1	1[inf]
	Eleutherodactylus goini	1	1	1[inf]
	Eleutherodactylus gossei	1	1	1
	Eleutherodactylus grabhami	1	1	1[inf]
	Eleutherodactylus grahami	1	1	1[inf]
	Eleutherodactylus greyi	1	1	1[inf]
	Eleutherodactylus griphus	1	1	1[inf]
	Eleutherodactylus gryllus	1	1	1[inf]
	Eleutherodactylus guanahacabibes	1	1	1[inf]
	Eleutherodactylus guantanamera	1	1	1[inf]
	Eleutherodactylus gundlachi	1	1	1[inf]
	Eleutherodactylus haitianus	1	1	1
	Eleutherodactylus hedricki	1	1	1[inf]
	Eleutherodactylus heminota	1	1	1
	Eleutherodactylus hypostenor	1	1	1[inf]
	Eleutherodactylus iberia	1	1	1[inf]
	Eleutherodactylus inoptatus	1	1	1
	Eleutherodactylus intermedius	1	1	1[inf]
	Eleutherodactylus ionthus	1	1	1[inf]
	Eleutherodactylus jamaicensis	1	1	1[inf]
	Eleutherodactylus jaumei	1	1	1[inf]
	Eleutherodactylus johnstonei	1	1	1[inf]
	Eleutherodactylus jugans	1	1	1
	Eleutherodactylus junori	1	1	1[inf]
	Eleutherodactylus klinikowskii	1	1	1[inf]

Eleutherodactylidae (cont.)	*Eleutherodactylus lamprotes*	1	1	1[inf]
	Eleutherodactylus leberi	1	1	1[inf]
	Eleutherodactylus lentus	1	1	1
	Eleutherodactylus leoncei	1	1	1[inf]
	Eleutherodactylus limbatus	1	1	1
	Eleutherodactylus locustus	1	1	1
	Eleutherodactylus luteolus	1	1	1[inf]
	Eleutherodactylus maestrensis	1	1	1[inf]
	Eleutherodactylus mariposa	1	1	1[inf]
	Eleutherodactylus marnockii	1	1	1
	Eleutherodactylus martinicensis	1	1	1
	Eleutherodactylus melacara	1	1	1[inf]
	Eleutherodactylus minutus	1	1	1
	Eleutherodactylus monensis	1	1	1[inf]
	Eleutherodactylus nitidus	1	1	1
	Eleutherodactylus nortoni	1	1	1[inf]
	Eleutherodactylus nubicola	1	1	1
	Eleutherodactylus orcutti	1	1	1
	Eleutherodactylus orientalis	1	1	1[inf]
	Eleutherodactylus oxyrhyncus	1	1	1[inf]
	Eleutherodactylus pantoni	1	1	1
	Eleutherodactylus parabates	1	1	1[inf]
	Eleutherodactylus parapelates	1	1	1[inf]
	Eleutherodactylus patriciae	1	1	1
	Eleutherodactylus paulsoni	1	1	1[inf]
	Eleutherodactylus pentasyringos	1	1	1[inf]
	Eleutherodactylus pezopetrus	1	1	1[inf]
	Eleutherodactylus pictissimus	1	1	1
	Eleutherodactylus pinarensis	1	1	1[inf]
	Eleutherodactylus pinchoni	1	1	1[inf]
	Eleutherodactylus pipilans	1	1	1
	Eleutherodactylus pituinus	1	1	1[inf]
	Eleutherodactylus planirostris	1	1	1
	Eleutherodactylus poolei	1	1	1[inf]
	Eleutherodactylus portoricensis	1	1	1
	Eleutherodactylus principalis	1	1	1[inf]
	Eleutherodactylus probalaeus	1	1	1[inf]
	Eleutherodactylus rhodesi	1	1	1[inf]
	Eleutherodactylus richmondi	1	1	1
	Eleutherodactylus ricordii	1	1	1
	Eleutherodactylus riparius	1	1	1[inf]
	Eleutherodactylus rivularis	1	1	1[inf]
	Eleutherodactylus rogersi	1	1	1[inf]

Eleutherodactylidae (cont.)	*Eleutherodactylus ronaldi*	1	1	1[inf]
	Eleutherodactylus rufifemoralis	1	1	1[inf]
	Eleutherodactylus ruthae	1	1	1
	Eleutherodactylus schmidti	1	1	1[inf]
	Eleutherodactylus schwartzi	1	1	1[inf]
	Eleutherodactylus sciagraphus	1	1	1[inf]
	Eleutherodactylus simulans	1	1	1[inf]
	Eleutherodactylus sisyphodemus	1	1	1[inf]
	Eleutherodactylus sommeri	1	1	1[inf]
	Eleutherodactylus symingtoni	1	1	1[inf]
	Eleutherodactylus thomasi	1	1	1[inf]
	Eleutherodactylus thorectes	1	1	1[inf]
	Eleutherodactylus toa	1	1	1[inf]
	Eleutherodactylus tonyi	1	1	1[inf]
	Eleutherodactylus turquinensis	1	1	1[inf]
	Eleutherodactylus unicolor	1	1	1[inf]
	Eleutherodactylus varians	1	1	1[inf]
	Eleutherodactylus varleyi	1	1	1
	Eleutherodactylus ventrilineatus	1	1	1[inf]
	Eleutherodactylus weinlandi	1	1	1
	Eleutherodactylus wetmorei	1	1	1[inf]
	Eleutherodactylus wightmanae	1	1	1
	Eleutherodactylus zeus	1	1	1[inf]
	Eleutherodactylus zugi	1	1	1
	Phyzelaphryne miriamae	1	1	1[inf]
Heleophrynidae	*Hadromophryne natalensis*	0	?	1
	Heleophryne purcelli	0	1	1
Hemiphractidae	*Flectonotus fitzgeraldi*	?	1	1[inf]
	Flectonotus pygmaeus	?	1	1[inf]
	Gastrotheca argenteovirens	?	1	1[inf]
	Gastrotheca atympana	0	0	1
	Gastrotheca aureomaculata	1	1	1[inf]
	Gastrotheca christiani	1	1	1
	Gastrotheca chrysosticta	1	1	1[inf]
	Gastrotheca cornuta	1	1	1[inf]
	Gastrotheca dendronastes	?	1	1
	Gastrotheca dunni	?	1	1
	Gastrotheca excubitor	1	1	1[inf]
	Gastrotheca fissipes	1	1	1[inf]
	Gastrotheca galeata	?	1	1
	Gastrotheca gracilis	1	1	1[inf]
	Gastrotheca griswoldi	?	1	1
	Gastrotheca guentheri	?	1	1

	Gastrotheca helenae	0	1	1
	Gastrotheca litonedis	?	1	1
	Gastrotheca longipes	?	1	1
	Gastrotheca marsupiata	?	1	1
	Gastrotheca monticola	?	1	1
	Gastrotheca nicefori	1	1	1
	Gastrotheca ochoai	?	1	1
	Gastrotheca orophylax	?	1	1
	Gastrotheca peruana	?	1	1
	Gastrotheca plumbea	?	1	1[inf]
	Gastrotheca pseustes	?	1	1
	Gastrotheca psychrophila	?	1	1
	Gastrotheca riobambae	1	1	1[inf]
Hemiphractidae (cont.)	*Gastrotheca ruizi*	?	1	1
	Gastrotheca stictopleura	?	1	1
	Gastrotheca trachyceps	?	1	1
	Gastrotheca weinlandii	?	1	1
	Gastrotheca zeugocystis	?	1	1
	Gastrotheca walkeri	0	1	1
	Hemiphractus bubalus	?	1	1
	Hemiphractus helioi	?	1	1
	Hemiphractus proboscideus	?	1	1
	Hemiphractus scutatus	?	1	1
	Stefania evansi	1	1	1
	Stefania ginesi	1	1	1
	Stefania scalae	?	1	1
	Stefania schuberti	1	1	1
Hemisotidae	*Hemisus marmoratus*	0	0	0
	Acris crepitans	?	1	1
	Acris gryllus	?	1	1[inf]
	Agalychnis annae	?	1	1[inf]
	Agalychnis aspera	?	1	1[inf]
	Agalychnis callidryas	1	1	1
	Agalychnis dacnicolor	1	1	1
	Agalychnis granulosa	?	1	1
	Agalychnis hulli	0	1	1[inf]
Hylidae	*Agalychnis lemur*	?	1	1
	Agalychnis litodryas	?	1	1[inf]
	Agalychnis moreletii	?	1	1
	Agalychnis saltator	?	1	1[inf]
	Agalychnis spurrelli	?	1	1
	Anotheca spinosa	1	1	1[inf]
	Aparasphenodon brunoi	?	?	1
	Aplastodiscus albofrenatus	1	1	1[inf]
	Aplastodiscus albosignatus	1	1	1[inf]

Hylidae (cont.)	*Aplastodiscus arildae*	?	1	1[inf]
	Aplastodiscus cavicola	?	1	1[inf]
	Aplastodiscus eugenioi	?	1	1[inf]
	Aplastodiscus leucopygius	?	1	1[inf]
	Aplastodiscus perviridis	1	1	1
	Aplastodiscus weygoldti	?	1	1[inf]
	Argenteohyla siemersi	1	1	1
	Bokermannohyla astartea	1	1	1[inf]
	Bokermannohyla circumdata	1	1	1[inf]
	Bokermannohyla hylax	1	1	1[inf]
	Bokermannohyla martinsi	?	1	1[inf]
	Bromeliohyla bromeliacia	?	1	1
	Charadrahyla nephila	?	1	1[inf]
	Charadrahyla taeniopus	?	1	1[inf]
	Corythomantis greeningi	?	?	1
	Cruziohyla calcarifer	1	1	1[inf]
	Dendropsophus anceps	1	1	1[inf]
	Dendropsophus berthalutzae	1	1	1[inf]
	Dendropsophus bifurcus	1	1	1[inf]
	Dendropsophus bipunctatus	1	1	1[inf]
	Dendropsophus branneri	1	1	1[inf]
	Dendropsophus brevifrons	?	1	1[inf]
	Dendropsophus ebraccatus	1	1[inf]	1
	Dendropsophus elegans	?	1	1[inf]
	Dendropsophus gaucheri	?	1	1[inf]
	Dendropsophus juliani	?	1	1[inf]
	Dendropsophus koechlini	?	1	1[inf]
	Dendropsophus labialis	?	1	1[inf]
	Dendropsophus marmoratus	1	1	1[inf]
	Dendropsophus melanargyreus	?	1	1[inf]
	Dendropsophus microcephalus	1	1	1
	Dendropsophus minutus	1	1	1
	Dendropsophus miyatai	?	1	1[inf]
	Dendropsophus nanus	1	1	1[inf]
	Dendropsophus parviceps	?	1	1[inf]
	Dendropsophus pelidna	1	1	1[inf]
	Dendropsophus rhodopeplus	?	1	1[inf]
	Dendropsophus robertmertensi	?	1	1[inf]
	Dendropsophus rubicundulus	?	1	1[inf]
	Dendropsophus sarayacuensis	?	1	1[inf]
	Dendropsophus sartoti	?	1	1[inf]
	Dendropsophus schubarti	0&1	1	1[inf]
	Dendropsophus seniculus	1	1	1[inf]
	Dendropsophus timbeba	0	1	1[inf]
	Dendropsophus triangulum	?	1	1[inf]

Hylidae (cont.)	*Dendropsophus tritaeniatus*	?	1	1[inf]
	Diaglena spatulata	?	1	1
	Duellmanohyla rufioculis	?	1	1[inf]
	Duellmanohyla soralia	1	1	1[inf]
	Ecnomiohyla miliaria	1	1	1[inf]
	Ecnomiohyla miotympanum	?	1	1
	Exerodonta abdivita	?	1	1[inf]
	Exerodonta chimalaà	?	1	1[inf]
	Exerodonta melanomma	?	?	1
	Exerodonta perkinsi	?	1	1[inf]
	Exerodonta smaragdina	?	1	1[inf]
	Exerodonta sumichrasti	?	1	1
	Exerodonta xera	?	1	1[inf]
	Hyla annectans	?	1	1[inf]
	Hyla andersonii	1	1	1[inf]
	Hyla arborea	?	1	1
	Hyla arenicolor	?	?	1
	Hyla avivoca	1	1	1[inf]
	Hyla chinensis	?	1	1
	Hyla chrysoscelis	1	1	1[inf]
	Hyla cinerea	1	1	1
	Hyla euphorbiacea	?	1	1[inf]
	Hyla eximia	?	?	1
	Hyla femoralis	1	1	1[inf]
	Hyla gratiosa	1	1	1[inf]
	Hyla meridionalis	1	1	1[inf]
	Hyla plicata	?	1	1[inf]
	Hyla squirella	1	1	1[inf]
	Hyla versicolor	1	1[inf]	1
	Hyla walkeri	1	1	1[inf]
	Hyla wrightorum	1	1	1[inf]
	Hyloscirtus alytolylax	?	1	1[inf]
	Hyloscirtus armatus	?	1	1[inf]
	Hyloscirtus colymba	?	1	1
	Hyloscirtus larinopygion	1	1	1[inf]
	Hyloscirtus lindae	?	1	1
	Hyloscirtus pacha	?	?	1
	Hyloscirtus palmeri	0	1	1[inf]
	Hyloscirtus pantostictus	1	1	1
	Hyloscirtus phyllognathus	?	1	1[inf]
	Hyloscirtus psarolaimus	1	1	1
	Hyloscirtus ptychodactylus	1	1	1
	Hyloscirtus simmonsi	?	1	1[inf]
	Hyloscirtus staufferorum	1	1	1
	Hyloscirtus tapichalaca	?	1	1

Hylidae (cont.)	*Hyloscirtus tigrinus*	1	1	1[inf]
	Hypsiboas albomarginatus	1	1	1
	Hypsiboas albopunctatus	?	1	1[inf]
	Hypsiboas andinus	?	1	1[inf]
	Hypsiboas balzani	?	1	1[inf]
	Hypsiboas bischoffi	1	1	1[inf]
	Hypsiboas boans	1	1	1[inf]
	Hypsiboas caingua	1	1	1[inf]
	Hypsiboas calcaratus	?	1	1[inf]
	Hypsiboas cinerascens	0	1	1[inf]
	Hypsiboas cordobae	?	1	1[inf]
	Hypsiboas crepitans	?	1	1[inf]
	Hypsiboas dentei	?	1	1[inf]
	Hypsiboas ericae	?	1	1[inf]
	Hypsiboas faber	1	1	1[inf]
	Hypsiboas fasciatus	?	1	1[inf]
	Hypsiboas geographicus	1	1	1[inf]
	Hypsiboas guentheri	?	1	1[inf]
	Hypsiboas heilprini	?	?	1
	Hypsiboas joaquini	0	1	1[inf]
	Hypsiboas lanciformis	1	1	1[inf]
	Hypsiboas latistriatus	?	1	1[inf]
	Hypsiboas leptolineatus	?	1	1[inf]
	Hypsiboas lundii	?	1	1[inf]
	Hypsiboas marginatus	0	1	1[inf]
	Hypsiboas marianitae	?	1	1[inf]
	Hypsiboas melanopleura	?	1	1[inf]
	Hypsiboas microderma	?	1	1[inf]
	Hypsiboas multifasciatus	?	1	1[inf]
	Hypsiboas nympha	?	1	1[inf]
	Hypsiboas ornatissimus	1	1	1[inf]
	Hypsiboas pardalis	?	1	1[inf]
	Hypsiboas pellucens	?	1	1[inf]
	Hypsiboas picturatus	1	1	1[inf]
	Hypsiboas polytaenius	1	1	1[inf]
	Hypsiboas prasinus	?	1	1[inf]
	Hypsiboas pulchellus	1	1	1[inf]
	Hypsiboas punctatus	1	1	1[inf]
	Hypsiboas raniceps	1	1	1[inf]
	Hypsiboas riojanus	1	1	1[inf]
	Hypsiboas roraima	?	1	1[inf]
	Hypsiboas rosenbergi	1	1	1
	Hypsiboas rufitelus	?	?	1
	Hypsiboas semiguttatus	?	1	1[inf]
	Hypsiboas semilineatus	1	1	1[inf]

Hylidae (cont.)	*Hypsiboas sibleszi*	?	1	1^{inf}
	Isthmohyla pseudopuma	1	1	1^{inf}
	Isthmohyla rivularis	0	1	1^{inf}
	Isthmohyla tica	?	1	1
	Itapothyla langsdorfii	1	1	1^{inf}
	Litoria adelaidensis	1	1	1^{inf}
	Litoria alboguttata	1	1	1^{inf}
	Litoria amboinensis	1	1	1^{inf}
	Litoria andiirrmalin	1	1	1^{inf}
	Litoria angiana	?	?	1
	Litoria arfakiana	?	1	1^{inf}
	Litoria aurea	1	1	1^{inf}
	Litoria australis	1	1	1
	Litoria barringtonensis	1	1	1^{inf}
	Litoria bicolor	1	1	1^{inf}
	Litoria booroolongensis	1	1	1^{inf}
	Litoria brevipes	1	1	1
	Litoria burrowsi	1	1	1^{inf}
	Litoria caerulea	1	1	1
	Litoria cavernicola	1	1	1^{inf}
	Litoria chloris	?	1	1^{inf}
	Litoria citropa	1	1	1^{inf}
	Litoria congenita	1	1	1^{inf}
	Litoria coplandi	1	1	1^{inf}
	Litoria cryptotis	?	1	1^{inf}
	Litoria cultripes	1	1	1
	Litoria cyclorhyncha	1	1	1^{inf}
	Litoria dahlii	1	1	1
	Litoria darlingtoni	1	1	1^{inf}
	Litoria daviesae	?	1	1^{inf}
	Litoria dayi	0	1	1^{inf}
	Litoria dentata	1	1	1^{inf}
	Litoria electrica	1	1	1^{inf}
	Litoria eucnemis	1	1	1^{inf}
	Litoria ewingii	1	1	1
	Litoria fallax	1	1	1^{inf}
	Litoria freycineti	1	1	1^{inf}
	Litoria genimaculata	1	1	1^{inf}
	Litoria gilleni	1	1	1^{inf}
	Litoria gracilenta	?	1	1^{inf}
	Litoria havina	1	1	1^{inf}
	Litoria impura	1	1	1^{inf}
	Litoria inermis	?	1	1^{inf}
	Litoria jervisiensis	1	1	1^{inf}
	Litoria jungguy	1	1	1^{inf}

Hylidae (cont.)	*Litoria kumae*	1	1	1[inf]
	Litoria latopalmata	?	?	1
	Litoria lesueurii	1	1	1
	Litoria longipes	1	1	1[inf]
	Litoria longirostris	1	1	1[inf]
	Litoria maculosa	1	1	1[inf]
	Litoria maini	1	1	1[inf]
	Litoria manya	?	1	1[inf]
	Litoria meiriana	1	1	1[inf]
	Litoria microbelos	1	1	1[inf]
	Litoria micromembrana	?	?	1
	Litoria modica	?	?	1
	Litoria moorei	1	1	1
	Litoria nannotis	0	?	?
	Litoria nasuta	1	1	1[inf]
	Litoria nigrofrenata	1	1	1[inf]
	Litoria novaehollandiae	1	1	1[inf]
	Litoria nudidigita	?	1	1[inf]
	Litoria nyakalensis	0	1	1[inf]
	Litoria pallida	?	1	1[inf]
	Litoria paraewingi	1	1	1[inf]
	Litoria pearsoniana	1	1	1[inf]
	Litoria peronii	1	1	1[inf]
	Litoria personata	1	1	1[inf]
	Litoria phyllochroa	0	1	1
	Litoria platycephala	1	1	1
	Litoria pronimia	1	1	1[inf]
	Litoria raniformis	1	1	1[inf]
	Litoria revelata	1	1	1[inf]
	Litoria rheocola	?	1	1[inf]
	Litoria rothi	1	1	1[inf]
	Litoria rubella	1	1	1[inf]
	Litoria splendida	1	1	1[inf]
	Litoria subglandulosa	0	1	1[inf]
	Litoria tornieri	1	1	1[inf]
	Litoria tyleri	1	1	1[inf]
	Litoria vagitus	1	1	1[inf]
	Litoria verreauxii	1	1	1
	Litoria verrucosa	1	1	1[inf]
	Litoria watjulumensis	1	1	1[inf]
	Litoria wilcoxii	1	1	1[inf]
	Litoria xanthomera	1	1	1[inf]
	Lysapsus limellum	1	1	1[inf]
	Megastomatohyla mixe	0	1	1[inf]
	Myersiohyla imparquesi	1	1	1[inf]

Hylidae (cont.)	*Myersiohyla kanaima*	?	1	1^{inf}
	Nyctimantis rugiceps	?	1	1^{inf}
	Nyctimystes brevipalmatus	1	1	1^{inf}
	Nyctimystes foricula	?	?	1
	Nyctimystes infrafrenatus	1	1	1
	Nyctimystes kubori	?	?	1
	Nyctimystes narinosus	?	?	1
	Nyctimystes papua	?	?	1
	Nyctimystes pulcher	?	1	1
	Nyctymistes zweifeli	?	?	1
	Osteocephalus alboguttatus	?	1	1^{inf}
	Osteocephalus buckleyi	1	1	1^{inf}
	Osteocephalus cabrerai	1	1	1^{inf}
	Osteocephalus deridens	1	1	1^{inf}
	Osteocephalus fuscifascies	1	1	1^{inf}
	Osteocephalus leprieurii	1	1	1
	Osteocephalus mutabor	1	1	1^{inf}
	Osteocephalus planiceps	1	1	1^{inf}
	Osteocephalus oophagus	1	1	1^{inf}
	Osteocephalus taurinus	?	?	1
	Osteocephalus verruciger	1	1	1^{inf}
	Osteocephalus yasuni	1	1	1^{inf}
	Osteopilus brunneus	?	1	1^{inf}
	Osteopilus crucialis	?	1	1^{inf}
	Osteopilus dominicensis	?	1	1^{inf}
	Osteopilus marianae	?	1	1^{inf}
	Osteopilus pulchrilineatus	?	1	1^{inf}
	Osteopilus septentrionalis	1	1	1
	Osteopilus vastus	?	1	1^{inf}
	Osteopilus wilderi	?	?	1
	Phasmahyla cochranae	?	1	1^{inf}
	Phasmahyla cruzi	?	1	1^{inf}
	Phasmahyla exilis	?	1	1^{inf}
	Phasmahyla guttata	?	?	1
	Phasmahyla jandaia	?	1	1^{inf}
	Phrynomedusa marginata	?	1	1^{inf}
	Phyllodytes auratus	?	1	1^{inf}
	Phyllodytes luteolus	?	?	1
	Phyllomedusa atelopoides	?	1	1
	Phyllomedusa ayeaye	?	1	1^{inf}
	Phyllomedusa azurea	1	1	1
	Phyllomedusa bahiana	?	1	1^{inf}
	Phyllomedusa baltea	?	1	1^{inf}
	Phyllomedusa bicolor	0	1	1
	Phyllomedusa boliviana	?	1	1^{inf}

Hylidae (cont.)	*Phyllomedusa burmeisteri*	?	1	1^{inf}
	Phyllomedusa camba	0	1	1^{inf}
	Phyllomedusa centralis	?	1	1^{inf}
	Phyllomedusa duellmani	?	1	1^{inf}
	Phyllomedusa hypochondrialis	?	1	1^{inf}
	Phyllomedusa iheringii	?	1	1^{inf}
	Phyllomedusa megacephala	?	1	1^{inf}
	Phyllomedusa neildi	?	1	1^{inf}
	Phyllomedusa nordestina	?	1	1^{inf}
	Phyllomedusa oreades	?	1	1^{inf}
	Phyllomedusa palliata	1	1	1^{inf}
	Phyllomedusa perinesos	?	1	1^{inf}
	Phyllomedusa rohdei	?	1	1^{inf}
	Phyllomedusa sauvagii	1	1	1
	Phyllomedusa tarsius	1	1	1^{inf}
	Phyllomedusa tetraploidea	?	1	1^{inf}
	Phyllomedusa tomopterna	0	1	1^{inf}
	Phyllomedusa trinitatis	?	1	1^{inf}
	Phyllomedusa vaillanti	?	1	1^{inf}
	Plectrohyla ameibothalame	?	1	1^{inf}
	Plectrohyla arborescandens	?	1	1^{inf}
	Plectrohyla bistincta	?	1	1^{inf}
	Plectrohyla calthula	?	1	1^{inf}
	Plectrohyla chrysopleura	?	1	1^{inf}
	Plectrohyla cyclada	?	1	1^{inf}
	Plectrohyla glandulosa	?	1	1^{inf}
	Plectrohyla guatemalensis	?	1	1^{inf}
	Plectrohyla mutadai	?	1	1^{inf}
	Plectrohyla pentheter	?	1	1^{inf}
	Plectrohyla siopela	?	1	1
	Pseudacris brachypona	1	1	1^{inf}
	Pseudacris brimleyi	1	1	1^{inf}
	Pseudacris cadaverina	1	1	1^{inf}
	Pseudacris clarkii	?	?	1
	Pseudacris crucifer	1	1	1^{inf}
	Pseudacris feriarum	1	1	1^{inf}
	Pseudacris fouquetti	1	1	1^{inf}
	Pseudacris illinoensis	1	1	1^{inf}
	Pseudacris kalmi	1	1	1^{inf}
	Pseudacris maculata	1	1	1^{inf}
	Pseudacris nigrita	?	1	1^{inf}
	Pseudacris ocularis	?	1	1
	Pseudacris ornata	?	1	1
	Pseudacris regilla	?	1	1
	Pseudacris streckeri	1	1^{inf}	1

Hylidae (cont.)	*Pseudacris triseriata*	?	1	1[inf]
	Pseudis bolbodactyla	?	1	1[inf]
	Pseudis cardosoi	?	1	1[inf]
	Pseudis fusca	?	1	1[inf]
	Pseudis minuta	1	1	1
	Pseudis paradoxa	1	1	1
	Pseudis tocantins	?	1	1[inf]
	Ptychohyla dendrophasma	?	1	1[inf]
	Ptychohyla euthysanota	?	1	1[inf]
	Ptychohyla hypomykter	?	1	1[inf]
	Ptychohyla leonhardschultzei	?	1	1[inf]
	Ptychohyla spinipollex	?	1	1
	Ptychohyla zophodes	?	1	1[inf]
	Scarthyla goinorum	1	1	1
	Scinax acuminatus	1	1	1[inf]
	Scinax berthae	1	1	1[inf]
	Scinax boesemani	1	1	1[inf]
	Scinax boulengeri	1	1[inf]	1
	Scinax catharinae	?	1	1
	Scinax crospedospilus	?	1	1
	Scinax cruentommus	?	1	1
	Scinax elaeochrous	?	1	1
	Scinax faivovichi	1	1	1[inf]
	Scinax fuscovarius	1	1	1[inf]
	Scinax garbei	1	1	1
	Scinax nasicus	?	1	1[inf]
	Scinax nebulosus	?	1	1
	Scinax perpusillus	1	1	1
	Scinax rostratus	?	1	1
	Scinax ruber	?	1	1
	Scinax squalirostris	?	1	1
	Scinax staufferi	?	1	1[inf]
	Scinax uruguayus	1	1	1[inf]
	Smilisca baudinii	1	1	1[inf]
	Smilisca cyanosticta	?	1	1[inf]
	Smilisca fodiens	?	1	1
	Smilisca phaeota	1	1	1
	Smilisca puma	?	1	1[inf]
	Smilisca sila	?	1	1[inf]
	Smilisca sordida	?	1	1
	Sphaenorhynchus lacteus	1	1	1
	Tepuhyla talbergae	1	1	1[inf]
	Tepuihyla aecii	?	1	1[inf]
	Tepuihyla edelcae	?	1	1[inf]
	Tepuihyla exophthalma	1	1	1

Hylidae (cont.)	*Tepuihyla rodriguezi*	?	1	1[inf]
	Tlalocohyla godmani	?	1	1[inf]
	Tlalocohyla loquax	1	1	1
	Tlalocohyla picta	?	1	1[inf]
	Tlalocohyla smithtii	?	1	1[inf]
	Trachycephalus hadroceps	1	1	1[inf]
	Trachycephalus jordani	?	?	1
	Trachycephalus mesophaeus	1	1	1[inf]
	Trachycephalus nigromaculatus	?	?	1
	Trachycephalus resinifictrix	1	1	1[inf]
	Trachycephalus typhonius	1	1	1
	Triprion petasatus	?	1	1
	Xenohyla truncata	1	1	1
Hylodidae	*Crossodactylus schmidti*	1	1	1
	Hylodes ornatus	?	1	1[inf]
	Megaelosia goeldii	?	?	1
Hyperoliidae	*Acanthixalus spinosus*	0	1	1[inf]
	Afrixalus dorsalis	?	1	1[inf]
	Afrixalus fornasini	0	1	1
	Afrixalus laevis	?	?	1
	Alexteroon obstetricans	?	1	1[inf]
	Cryptothylax greshoffi	?	1	1
	Heterixalus alboguttatus	?	1	1[inf]
	Heterixalus betsileo	0	?	1
	Heterixalus boettgeri	0	1	1[inf]
	Heterixalus carbonei	?	1	1[inf]
	Heterixalus madagascariensis	0	1	1
	Heterixalus punctatus	?	1	1[inf]
	Heterixalus tricolor	?	1	1[inf]
	Hyperolius benguellensis	0	?	1
	Hyperolius castaneus	?	1	1
	Hyperolius chlorosteus	?	1	1[inf]
	Hyperolius concolor	?	1	1[inf]
	Hyperolius guttulatus	?	1	1[inf]
	Hyperolius nasutus	0	1	1
	Hyperolius ocellatus	?	1	1
	Hyperolius phantasticus	?	1	1
	Hyperolius puncticulatus	?	1	1[inf]
	Hyperolius pusillus	0	?	1
	Hyperolius tuberilinguis	0	1	1[inf]
	Hyperolius viridiflavus	1	1	1
	Kassina maculata	0	1	1[inf]
	Kassina senegalensis	0	1	1
	Morerella cyanophthalma	1	1	1

Hyperoliidae (cont.)	*Opisthotylax immaculatus*	0	?	1
	Phlyctimantis leonardi	?	1	1^{inf}
	Phlyctimantis verrucosus	0	1	1
	Semnodactylus wealii	0	1	1^{inf}
	Tachycnemis seychellensis	?	1	1^{inf}
Leiopelmatidae	*Leiopelma archeyi*	0^{inf}	0^{inf}	0
	Leiopelma hochstetteri	0	0	0
Leptodactylidae	*Adenomera andreae*	1	1	1^{inf}
	Adenomera heyeri	1	1	1
	Adenomera hylaedactyla	1	1	1
	Edalorhina perezi	1	1	1
	Engystomops coloradum	1	1^{inf}	1^{inf}
	Engystomops guayaco	1	1^{inf}	1^{inf}
	Engystomops montubio	1	1^{inf}	1^{inf}
	Engystomops petersi	1	1	1
	Engystomops pustulatus	1	1	1
	Engystomops pustulosus	1	?	1
	Engystomops randi	1	1^{inf}	1^{inf}
	Leptodactylus albilabris	1	1	1
	Leptodactylus bolivianus	1	1	1
	Leptodactylus bufonius	1	1	1
	Leptodactylus chaquensis	1	1	1
	Leptodactylus diedrus	1	1	1^{inf}
	Leptodactylus discodactylus	1	1	1^{inf}
	Leptodactylus didymus	1	1	1^{inf}
	Leptodactylus elenae	1	1	1^{inf}
	Leptodactylus fallax	1	1	1^{inf}
	Leptodactylus fuscus	1	1	1
	Leptodactylus gracilis	1	1	1
	Leptodactylus griseigularis	1	1	1^{inf}
	Leptodactylus knudseni	1	1	1^{inf}
	Leptodactylus labyrinthicus	1	1	1^{inf}
	Leptodactylus latrans	1	1	1
	Leptodactylus leptodactyloides	1	1	1^{inf}
	Leptodactylus longirostris	1	1	1^{inf}
	Leptodactylus melanonotus	1	1	1
	Leptodactylus myersi	1	1	1^{inf}
	Leptodactylus mystaceus	1	1	1
	Leptodactylus mystacinus	1	1	1
	Leptodactylus notoaktites	1	1	1^{inf}
	Leptodactylus pentadactylus	1	1	1
	Leptodactylus plaumanni	1	1	1^{inf}
	Leptodactylus podicipinus	1	1	1
	Leptodactylus rhodonotus	1	1	1^{inf}
	Leptodactylus rhodomystax	1	1	1^{inf}

Leptodactylidae (cont.)	*Leptodactylus riveroi*	1	1	1[inf]
	Leptodactylus silvanimbus	1	1	1[inf]
	Leptodactylus spixi	1	1	1[inf]
	Leptodactylus stenodema	1	1	1[inf]
	Leptodactylus syphax	1	1	1
	Leptodactylus validus	1	1	1[inf]
	Leptodactylus vastus	1	1	1[inf]
	Leptodactylus wagneri	1	1	1
	Lithodytes lineatus	1	1	1
	Paratelmatobius cardosoi	?	?	1
	Paratelmatobius poecilogaster	?	?	1
	Physalaemus albonotatus	1	1	1
	Physalaemus biligonigerus	0	?	1
	Physalaemus centralis	1	1	1
	Physalaemus cuvieri	0&1	1	1
	Physalaemus gracilis	1	1	1
	Physalaemus nattereri	0	1	1
	Physalaemus signifer	?	?	1
	Pleurodema brachyops	1	1	1
	Pleurodema cinereum	1	1	1
	Pleurodema diplolister	?	1	1
	Pleurodema kriegi	?	1	1
	Pleurodema marmoratum	?	?	1
	Pleurodema tucumanum	?	1	1
	Pseudopaludicola falcipes	0	0	1
	Pseudopaludicola mystacalis	0	0	1
	Scythrophrys sawayae	0	?	1
Limnodynastidae	*Adelotus brevis*	0	?	1
	Heleioporus australiacus	1	1	1
	Lechriodus fletcheri	0	1	1
	Lechriodus melanopyga	1	1	1[inf]
	Limnodynastes depressus	0	1	1[inf]
	Limnodynastes dorsalis	0	?	1
	Limnodynastes dumerilii	0&1	1	1[inf]
	Limnodynastes fletcheri	0	?	1
	Limnodynastes lignarius	1	1	1[inf]
	Limnodynastes peronii	0	1	1
	Limnodynastes salmini	0	1	1
	Limnodynastes tasmaniensis	0	?	1
	Neobatrachus pelobatoides	?	1	?
	Neobatrachus pictus	0	?	1
	Neobatrachus sudelli	0	?	1
	Notaden bennettii	0	?	1
	Philoria sphagnicolus	0	?	1
	Platyplectrum ornatum	0	1	1

Mantellidae	*Aglyptodactylus madagascariensis*	1	1	1[inf]
	Blommersia blommersae	0	1	1[inf]
	Blommersia domerguei	0	1	1[inf]
	Blommersia grandisonae	0	1	1[inf]
	Blommersia madinika	0	1	1[inf]
	Blommersia sarotra	0	1	1[inf]
	Boehmantis microtympanum	0	1	1[inf]
	Boophis ankaratra	0	1	1[inf]
	Boophis axelmeyeri	0	1	1[inf]
	Boophis bottae	0	1	1[inf]
	Boophis englaenderi	0	1	1[inf]
	Boophis goudotii	1	1	1
	Boophis idae	0	1	1[inf]
	Boophis madagascariensis	?	1	1[inf]
	Boophis mandraka	0	1	1[inf]
	Boophis marojezensis	0	1	1[inf]
	Boophis pauliani	0	1	1[inf]
	Boophis phyrrus	0	1	1[inf]
	Boophis picturatus	0	1	1[inf]
	Boophis rufioculis	0	1	1[inf]
	Boophis sambirano	?	1	1[inf]
	Boophis sibilans	0	1	1[inf]
	Boophis tephraeomystax	1	1	1[inf]
	Boophis viridis	0	1	1[inf]
	Boophis vittatus	0	1	1[inf]
	Boophis williamsi	0	1	1[inf]
	Gephyromantis asper	1	1	1[inf]
	Gephyromantis azurrae	1	1	1[inf]
	Gephyromantis blanci	?	1	1[inf]
	Gephyromantis boulengeri	1	1	1[inf]
	Gephyromantis cornutus	1	1	1[inf]
	Gephyromantis corvus	1	1	1[inf]
	Gephyromantis eiselti	1	1	1[inf]
	Gephyromantis granulatus	?	1	1[inf]
	Gephyromantis horridus	0	1	1[inf]
	Gephyromantis klemmeri	1	1	1[inf]
	Gephyromantis luecocephalus	1	1	1[inf]
	Gephyromantis leucomaculatus	1	1	1[inf]
	Gephyromantis luteus	0	1	1[inf]
	Gephyromantis malagasius	0	1	1[inf]
	Gephyromantis plicifer	1	1	1[inf]
	Gephyromantis pseudoasper	1	1	1[inf]
	Gephyromantis redimitus	1	1	1[inf]

Mantellidae (cont.)	*Gephyromantis rivicola*	1	1	1[inf]
	Gephyromantis salegy	1	1	1[inf]
	Gephyromantis sculpturatus	1	1	1[inf]
	Gephyromantis striatus	0	1	1[inf]
	Gephyromantis ventrimaculatus	1	1	1[inf]
	Gephyromantis zavona	1	1	1[inf]
	Guibemantis albolineatus	0	1	1[inf]
	Guibemantis punctatus	0	1	1[inf]
	Guibemantis liber	0	1	1[inf]
	Guibemantis pulcher	?	1	1[inf]
	Guibemantis punctatus	0	1	1[inf]
	Guibemantis tornieri	1	1	1[inf]
	Laliostoma labrosum	1	1	1[inf]
	Mantella aurantiaca	0	1	1
	Mantella baroni	0	1	1[inf]
	Mantella bernhardi	0	1	1[inf]
	Mantella betsileo	?	1	1[inf]
	Mantella crocea	?	1	1[inf]
	Mantella ebenaui	?	1	1[inf]
	Mantella expectata	0	1	1[inf]
	Mantella laevigata	?	1	1[inf]
	Mantella madagascariensis	?	1	1[inf]
	Mantella nigricans	?	1	1[inf]
	Mantidactylus aerumnalis	1	1	1[inf]
	Mantidactylus ambreensis	1	1	1[inf]
	Mantidactylus argenteus	1	1	1[inf]
	Mantidactylus betsileanus	1	1	1[inf]
	Mantidactylus biporus	1	1	1[inf]
	Mantidactylus charlotteae	1	1	1[inf]
	Mantidactylus curtus	1	1	1[inf]
	Mantidactylus femoralis	0&1	1	1
	Mantidactylus lugubris	?	1	1[inf]
	Mantidactylus majori	1	1	1[inf]
	Mantidactylus mocquardi	1	1	1[inf]
	Mantidactylus opiparis	1	1	1[inf]
	Mantidactylus ulcerosus	1	1	1[inf]
	Spinomantis aglavei	?	1	1[inf]
	Spinomantis guibei	?	1	1[inf]
	Tsingymantis antitra	1	1	1[inf]
Megophryidae	*Brachytarsophrys feae*	0	?	?
	Leptobrachium banae	?	1	1[inf]
	Leptobrachium chapaense	1	1	1[inf]
	Leptobrachium echinatum	0	1	1[inf]
	Leptobrachium hasseltii	1	1	1

Megophryidae (cont.)	*Leptobrachium hendricksoni*	1	1	1^{inf}
	Leptobrachium mouhoti	?	1	1^{inf}
	Leptobrachium montanum	1	1	1^{inf}
	Leptobrachium nigrops	1	1	1^{inf}
	Leptobrachium pullum	1	1	1^{inf}
	Leptobrachium smithi	1	1	1^{inf}
	Leptolalax arayai	1	1	1^{inf}
	Leptolalax bourreti	0	1	1^{inf}
	Leptolalax oshanensis	?	1	1^{inf}
	Leptolalax pelodytoides	0	1	1^{inf}
	Leptolalax pictus	1	1	1^{inf}
	Leptolalax pluvialis	1	1	1^{inf}
	Leptolalax ventripunctatus	1	1	1^{inf}
	Megophrys baluensis	?	1	1^{inf}
	Megophrys longipes	?	1	1^{inf}
	Megophrys major	?	1	1^{inf}
	Megophrys minor	?	1	1^{inf}
	Megophrys nasuta	0	1	1
	Megophrys parva	?	1	1^{inf}
	Megophrys shapingensis	?	1	1^{inf}
	Megophrys spinata	?	1	1^{inf}
	Ophryophryne han si	?	1	1^{inf}
	Ophryophryne microstoma	?	1	1^{inf}
	Oreolalax chuanbeiensis	0	1	1
	Oreolalax jiandongensis	0	?	?
	Oreolalax liangbeiensis	0	1	1
	Oreolalax lichuanensis	0	1	1
	Oreolalax major	0	1	1
	Oreolalax multipunctatus	0	1	1
	Oreolalax nanjiangensis	0	1	1
	Oreolalax omeimontis	0	1	1
	Oreolalax pingii	0	1	1
	Oreolalax popei	0	1	1
	Oreolalax rhodostigmatus	1	1	1
	Oreolalax rugosus	0	0	0
	Oreolalax schmidti	0	1	1
	Oreolalax xiangchengensis	0	?	?
	Scutiger boulengeri	?	?	1
	Scutiger chintingensis	0^{inf}	0^{inf}	0
	Scutiger mammatus	0	1	1
	Scutiger muliensis	0^{inf}	0^{inf}	0
	Scutiger tuberculatus	0^{inf}	0^{inf}	0
Micrixalidae	*Micrixalus fuscus*	?	1	1
	Micrixalus kottigeharensis	?	1	1^{inf}
	Micrixalus saxicola	?	1	1

Microhylidae	*Anodonthyla boulengerii*	0	1	1
	Anodonthyla montana	1	1	1
	Anodonthyla moramora	0	1	1^{inf}
	Anodonthyla rouxae	0	1	1^{inf}
	Apantophryne pansa	?	1	1
	Asterophrys turpicola	1	1	1^{inf}
	Austrochaperina palmipes	0	1	1
	Barygenys flavigularis	0	1	1^{inf}
	Calluela guttulata	0	1	1
	Calluela minuta	?	1	1
	Calluela yunnanensis	0	1	1
	Callulops robustus	0	1	1
	Chaperina fusca	0	1	1
	Chiasmocleis albopunctata	?	1	1
	Chiasmocleis hudsoni	0	1	1^{inf}
	Choerophryne rostellifer	?	1	1
	Cophixalus ornatus	?	?	1
	Cophyla berara	?	1	1
	Cophyla phyllodactyla	0	1	1
	Copiula derongo	0	1	1^{inf}
	Copiula guttata	?	?	1
	Ctenophryne aequatorialis	?	?	1
	Ctenophryne geayi	0	1	1
	Dasypops schirchi	0	1	1
	Dermatonotus muelleri	0	?	1
	Dyscophus antongilii	0	1	1
	Dyscophus guineti	0	1	1
	Dyscophus insularis	0	1	1
	Elachistocleis bicolor	0	1	1
	Gastrophryne carolinensis	0	1	1
	Gastrophryne elegans	?	1	1
	Genyophryne thomsoni	?	1	1
	Glyphoglossus molossus	1	1	1
	Hamptophryne boliviana	0	1	1
	Hoplophryne rogersi	0	0	0
	Hoplophryne uluguruensis	0	0	0
	Hylophorbus picoides	0	1	1^{inf}
	Hylophorbus rufescens	0	1	1
	Hypopachus variolosus	0	1	1
	Kalophrynus baluensis	?	1	1
	Kalophrynus interlineatus	?	1	1
	Kalophrynus intermedius	?	1	1^{inf}
	Kalophrynus pleurostigma	0	1	1
	Kaloula borealis	?	1	1
	Kaloula conjuncta	?	1	1

Microhylidae (cont.)	*Kaloula mediolineata*	?	1	1
	Kaloula picta	?	1	1
	Kaloula pulchra	1	1	1
	Liophryne rhododactyla	?	1	1
	Liophryne schlaginhaufeni	?	1	1
	Mantophryne lateralis	0	1	1^{inf}
	Melanobatrachus indicus	0	0	0
	Metamagnusia slateri	?	1	1^{inf}
	Metaphrynella pollicaris	?	1	1
	Metaphrynella sundana	?	1	1
	Microhyla achatina	?	1	1
	Microhyla annectens	0	1	1
	Microhyla berdmorei	0	1	1
	Microhyla borneensis	0	1	1
	Microhyla butleri	0	0&1	0&1
	Microhyla heymonsi	0^{inf}	0&1	0&1
	Microhyla okinavensis	0	1	1
	Microhyla ornata	?	1	1
	Microhyla palmipes	0	1	1
	Microhyla pulchra	0	1	1
	Microhyla superciliaris	0	1	1
	Micryletta inornata	?	1	1
	Oreophryne monticola	?	1	1
	Oreophryne sibilans	0	1	1^{inf}
	Oreophryne unicolor	0	1	1^{inf}
	Otophryne pyburni	1	1	1^{inf}
	Oxydactyla crassa	?	?	1
	Paradoxophyla palmata	0	1	1^{inf}
	Phrynella pulchra	?	1	1
	Phrynomantis bifasciatus	0	1	1
	Phrynomantis microps	0	1	1
	Platypelis grandis	?	1	1
	Platypelis milloti	0	1	1^{inf}
	Platypelis pollicaris	0	1	1
	Platypelis tuberifera	?	1	1
	Plethodonthyla bipunctata	?	1	1^{inf}
	Plethodonthyla guentheri	?	1	1^{inf}
	Plethodonthyla inguinalis	0	1	1
	Plethodonthyla mihanika	0	1	1^{inf}
	Plethodonthyla notosticta	0	1	1
	Plethodonthyla ocellata	0	1	1
	Plethodonthyla tuberata	?	1	1
	Pseudocallulops eurydactylus	1	1	1^{inf}
	Ramanella montana	?	1	1
	Ramanella obscura	?	1	1

Microhylidae (cont.)	*Ramanella variegata*	?	1	1
	Rhombophryne alluaudi	0	1	1[inf]
	Rhombophryne coronata	0	1	1[inf]
	Rhombophryne coudreaui	0	1	1[inf]
	Rhombophryne grandis	?	1	1[inf]
	Rhombophryne helenae	?	1	1[inf]
	Rhombophryne psologlossa	?	1	1
	Rhombophryne serratopalpebrosa	0	1	1
	Rhombophryne testudo	0	1	1
	Rhombophryne tetradactyla	1	1	1[inf]
	Rhombophryne tridactyla	0	1	1[inf]
	Scaphiophryne calcarata	0	1	1
	Scaphiophryne marmorata	0	1	1
	Scaphiophryne menabensis	0	1	1[inf]
	Sphenophryne cornuta	?	1	1
	Synapturanus mirandaribeiroi	0	1	1
	Uperodon systoma	0	?	1
	Xenorhina bouwensi	?	1	1
	Xenorhina obesa	0	1	1[inf]
	Xenorhina oxycephala	?	1	1[inf]
Myobatrachidae	*Assa darlingtoni*	0	1	1
	Crinia deserticola	?	?	1
	Crinia parinsignifera	?	?	1
	Crinia riparia	?	?	0
	Crinia signifera	0	?	1
	Crinia tinnula	?	?	1
	Geocrinia victoriana	0	1	1
	Metacrinia nichollsi	?	?	1
	Mixophyes carbinensis	1	1	1[inf]
	Mixophyes coggeri	1	1	1[inf]
	Mixophyes fasciolatus	1	1	1
	Mixophyes schevilli	1	1	1
	Myobatrachus gouldii	?	?	1
	Pseudophryne bibronii	0	0	0
	Pseudophryne coriacea	0	0	0
	Rheobatrachus silus	0	?	1
	Spicospina flammocaerulea	1	1	1
	Taudactylus acutirostris	0	?	1
	Uperoleia aspera	0	?	1
	Uperoleia borealis	?	?	1
	Uperoleia crassa	?	?	1
	Uperoleia fusca	?	?	1
	Uperoleia glandulosa	?	?	1
	Uperoleia inundata	?	?	1

Myobatrachidae (cont.)	*Uperoleia laevigata*	0	?	1
	Uperoleia lithomoda	?	?	1
	Uperoleia littlejohni	?	?	1
	Uperoleia micromeles	?	?	1
	Uperoleia minima	?	?	1
	Uperoleia mjobergii	?	?	1
	Uperoleia rugosa	0	?	1
	Uperoleia russelli	0	?	1
	Uperoleia talpa	?	?	1
	Uperoleia trachyderma	?	?	1
	Uperoleia tyleri	?	?	1
Nasikabatrachidae	*Nasikabatrachus sahyadrensis*	0	?	0[†]
Nyctibatrachidae	*Nyctibatrachus beddomii*	?	1	1[inf]
	Nyctibatrachus deccanensis	0	?	?
	Nyctibatrachus kempholeyensis	?	1	1[inf]
	Nyctibatrachus major	0	?	?
	Nyctibatrachus minimus	?	1	1[inf]
	Nyctibatrachus minor	?	1	1[inf]
	Nyctibatrachus sylvaticus	?	1	1
Odontophrynidae	*Macrogenioglottus alipioi*	0	1	1
	Odontophrynus achalensis	0	1	1
	Odontophrynus americanus	0	1	1
	Odontophrynus carvalhoi	0	1	1
	Odontophrynus cultripes	0	1	1
	Proceratophrys appendiculata	0	1	1
	Proceratophrys avelinoi	0	1	1
	Proceratophrys boiei	0	1	1
	Proceratophrys concavitympanum	?	1	1[inf]
	Proceratophrys cristiceps	0	1	1
	Proceratophrys laticeps	?	?	1
	Proceratophrys melanopogon	?	?	1
Pelodytidae	*Pelobates cultripes*	?	?	1
	Pelobates fuscus	0	?	1
	Pelobates syriacus	?	?	1
	Pelobates varaldii	?	?	1
	Pelodytes caucasicus	?	?	1
	Pelodytes ibericus	?	?	1
	Pelodytes punctatus	?	?	1
Petropedetidae	*Arthroleptides martiensseni*	1	1	1
	Arthroleptides yakusini	1	1	1[inf]
	Aubria subsigillata	1	1	1
	Petropedetes cameronensis	1	1	1
	Petropedetes newtoni	1	1	1
	Petropedetes parkeri	1	1	1

Phrynobatrachidae	*Phrynobatrachus acridoides*	1	1	1
	Phrynobatrachus africanus	0	1	1
	Phrynobatrachus calcaratus	0	?	?
	Phrynobatrachus cricogaster	0	1	1
	Phrynobatrachus dispar	?	1	1[inf]
	Phrynobatrachus krefftii	0	1	1
	Phrynobatrachus natalensis	0	1	1
	Phrynobatrachus sandersoni	0	1	1
Pipidae	*Hymenochyrus boettgeri*	?	1	1
	Pipa carvalhoi	?	?	1
	Pipa parva	?	1	1
	Pipa pipa	0	1	1
	Xenopus borealis	0	1	1
	Xenopus epitropicalis	?	1	1
	Xenopus fraseri	?	1	1
	Xenopus laevis	0	1	1
	Xenopus muelleri	?	1	1
	Xenopus tropicalis	1	1	1
Ptychadenidae	*Hildebrandtia ornata*	1	1	1
	Ptychadena aequiplicata	?	1	1[inf]
	Ptychadena anchietae	1	1	1
	Ptychadena mascareniensis	1	1	1
	Ptychadena oxyrhynchus	1	1	1[inf]
	Ptychadena porosissima	1	1	1[inf]
	Ptychadena taenioscelis	1	1	1[inf]
Pyxicephalidae	*Amietia angolensis*	1	1	1
	Amietia fuscigula	1	1	1
	Anhydrophryne rattrayi	1	1	1
	Arthroleptella landdrosia	0	1	1
	Cacosternum boettgeri	0	1	1
	Cacosternum capense	0	1	1
	Cacosternum nanum	0	1	1
	Microbatrachella capensis	0	1	1
	Natalobatrachus bonebergi	1	1	1
	Poyntonia paludicola	0	1	1
	Pyxicephalus adspersus	1	1	1
	Pyxicephalus edulis	1	1	1
	Strongylopus fasciatus	1	1	1[inf]
	Strongylopus grayii	1	1	1
	Tomopterna delalandii	0	1	1[inf]
	Tomopterna marmorata	0	1	1
	Tomopterna natalensis	0	1	1[inf]
	Tomopterna tandyi	0	1	1
	Tomopterna tuberculosa	1	1	1[inf]

Ranidae	*Abavorana luctuosa*	1	1	1^{inf}
	Amnirana albolabris	1	1	1
	Amnirana galamensis	1	1	1
	Amolops chunganensis	?	1	1^{inf}
	Amolops daorum	1	1	1^{inf}
	Amolops granulosus	?	1	1^{inf}
	Amolops hainanensis	?	1	1^{inf}
	Amolops hongkongensis	?	1	1^{inf}
	Amolops jinjiangensis	?	1	1^{inf}
	Amolops larutensis	?	1	1^{inf}
	Amolops loloensis	?	1	1^{inf}
	Amolops mantzorum	?	1	1^{inf}
	Amolops ricketti	0	1	1
	Amolops spinapectoralis	1	1	1^{inf}
	Amolops viridimaculatus	0	1	1^{inf}
	Babina adenopleura	?	1	1^{inf}
	Babina okinavana	?	1	1^{inf}
	Babina pleuraden	?	1	1^{inf}
	Chalcorana chalconota	1	1	1^{inf}
	Chalcorana eschatia	?	1	1^{inf}
	Chalcorana megalonesa	?	1	1^{inf}
	Chalcorana parvaccola	?	1	1^{inf}
	Chalcorana raniceps	?	1	1^{inf}
	Clinotarsus alticola	?	1	1^{inf}
	Clinotarsus curtipes	?	1	1^{inf}
	Glandirana rugosa	?	1	1^{inf}
	Glandirana tientaiensis	1	1	1^{inf}
	Huia cavitympanum	1	1	1^{inf}
	Huia masonii	?	1	1^{inf}
	Hydrophylax gracilis	1	1	1
	Hydrophylax malabaricus	1	1	1^{inf}
	Hylarana erythrea	1	1	1^{inf}
	Hylarana macrodactyla	?	1	1^{inf}
	Hylarana taipehensis	1	1	1
	Indosylvirana aurantiaca	1	1	1^{inf}
	Indosylvirana milleti	?	1	1^{inf}
	Lithobates areolatus	1	1	1^{inf}
	Lithobates berlandieri	?	1	1^{inf}
	Lithobates blairi	1	1	1^{inf}
	Lithobates bwana	1	1	1^{inf}
	Lithobates capito	?	1	1^{inf}
	Lithobates catesbeianus	1	1	1
	Lithobates chiricahuensis	1	1	1^{inf}
	Lithobates clamitans	1	1	1
	Lithobates fisheri	?	1	1^{inf}

Ranidae (cont.)	*Lithobates forreri*	1	1	1^{inf}
	Lithobates grylio	1	1	1^{inf}
	Lithobates heckscheri	1	1	1^{inf}
	Lithobates juliani	1	1	1^{inf}
	Lithobates macroglossa	?	1	1^{inf}
	Lithobates maculatus	?	1	1^{inf}
	Lithobates montezumae	?	1	1^{inf}
	Lithobates okaloosae	1	1	1^{inf}
	Lithobates onca	1	1	1^{inf}
	Lithobates palmipes	1	1	1^{inf}
	Lithobates palustris	1	1	1^{inf}
	Lithobates pipiens	1	1	1
	Lithobates septentrionalis	1	1	1^{inf}
	Lithobates sevosus	1	1	1^{inf}
	Lithobates sierramadrensis	?	1	1^{inf}
	Lithobates sphenocephalus	1	1	1^{inf}
	Lithobates sylvaticus	?	1	1^{inf}
	Lithobates tarahumarae	?	1	1^{inf}
	Lithobates taylori	1	1	1^{inf}
	Lithobates vaillanti	1	1	1^{inf}
	Lithobates vibicarius	1	1	1^{inf}
	Lithobates virgatipes	1	1	1
	Lithobates warszewitschii	1	1	1
	Lithobates yavapaiensis	1	1	1^{inf}
	Meristogenys jerboa	?	1	1^{inf}
	Meristogenys kinabaluensis	1	1	1^{inf}
	Meristogenys phaeomerus	1	1	1^{inf}
	Meristogenys poecilus	?	1	1^{inf}
	Meristogenys whiteheadi	?	1	1^{inf}
	Odorrana andersonii	?	1	1^{inf}
	Odorrana bacboensis	1	1	1^{inf}
	Odorrana chloronota	1	1	1
	Odorrana grahami	?	1	1^{inf}
	Odorrana hosii	?	1	1^{inf}
	Odorrana ishikawae	?	1	1^{inf}
	Odorrana jingdongensis	?	1	1^{inf}
	Odorrana junlianensis	?	1	1^{inf}
	Odorrona khalam	1	1	1^{inf}
	Odorrona livida	1	1	1^{inf}
	Odorrona margaretae	1	1	1^{inf}
	Odorrana morafkai	?	1	1^{inf}
	Odorrana narina	?	1	1^{inf}
	Odorrana tiannanensis	1	1	1^{inf}
	Odorrana tormota	1	1	1
	Odorrana schmackeri	1	1	1^{inf}

Ranidae (cont.)	*Odorrana swinhoana*	1	1	1[inf]
	Papurana arfaki	?	1	1[inf]
	Papurana daemeli	1	1	1[inf]
	Pelophylax cerigensis	1	1	1[inf]
	Pelophylax cretensis	1	1	1[inf]
	Pelophylax epeiroticus	?	1	1[inf]
	Pelophylax esculentus	1	1	1
	Pelophylax fukienensis	1	1	1[inf]
	Pelophylax nigromaculatus	1	1	1[inf]
	Pelophylax plancyi	1	1	1
	Pelophylax ridibundus	1	1	1
	Pelophylax saharicus	1	1	1[inf]
	Pulchrana baramica	?	1	1[inf]
	Pulchrana glandulosa	?	1	1[inf]
	Pulchrana signata	?	1	1[inf]
	Rana arvalis	1	1	1[inf]
	Rana aurora	1	1	1[inf]
	Rana boylii	?	1	1[inf]
	Rana cascadae	1	1	1[inf]
	Rana chaochiaoensis	?	1	1[inf]
	Rana chensinensis	?	1	1[inf]
	Rana dalmatina	?	1	1[inf]
	Rana hanluica	?	1	1[inf]
	Rana iberica	?	1	1[inf]
	Rana japonica	?	1	1[inf]
	Rana johnsi	?	1	1[inf]
	Rana latastei	?	1	1[inf]
	Rana longicrus	1	1	1[inf]
	Rana luteiventris	1	1	1[inf]
	Rana macrocnemis	?	1	1[inf]
	Rana muscosa	?	1	1[inf]
	Rana pirica	1	1	1[inf]
	Rana pretiosa	?	1	1[inf]
	Rana sauteri	1	1	1[inf]
	Rana shuchinae	?	1	1[inf]
	Rana tagoi	?	1	1[inf]
	Rana temporaria	?	1	1[inf]
	Sanguina sanguinea	1	1	1[inf]
	Staurois latopalmatus	?	1	1[inf]
	Staurois natator	1	1	1
	Staurois tuberilinguis	?	1	1[inf]
	Sylvirana guentheri	1	1	1[inf]
	Sylvirana maosonensis	?	1	1[inf]
	Sylvirana nigrovittata	?	1	1[inf]

Ranixalidae	*Indirana beddomi*	?	1	1[inf]
	Indirana brachytarsus	?	1	1[inf]
	Indirana diplosticta	?	1	1[inf]
	Indirana semipalmata	?	1	1
Rhacophoridae	*Buergeria buergeri*	?	1	1[inf]
	Buergeria japonica	1	1	1[inf]
	Buergeria robusta	1	1	1[inf]
	Chiromantis doriae	?	1	1[inf]
	Chiromantis petersii	?	1	1
	Chiromantis rufescens	?	1	1
	Chiromantis vittatus	?	1	
	Chiromantis xerampelina	0	1	1
	Feihyla kajau	?	1	1[inf]
	Feihyla palpebralis	?	1	1[inf]
	Ghatixalus variabilis	?	1	1
	Gracixalus gracilipes	?	1	1[inf]
	Gracixalus supercornutus	?	1	1[inf]
	Kurixalus appendiculatus	?	1	1[inf]
	Kurixalus banaensis	?	1	1[inf]
	Kurixalus eiffingeri	1	1	1[inf]
	Kurixalus idiooticus	1	1	1[inf]
	Kurixalus odontotarsus	?	1	1[inf]
	Liuixalus romeri	1	1	1[inf]
	Nyctixalus pictus	1	1	1[inf]
	Philautus acutirostris	0	?	?
	Philautus acutus	0	?	?
	Philautus aurantius	?	1	1[inf]
	Philautus aurifasciatus	?	1	1[inf]
	Philautus bunitus	1	1	1[inf]
	Philautus hosii	?	1	1[inf]
	Philautus petersi	0	1	1[inf]
	Philautus surdus	0	1	1
	Philautus umbra	?	1	1[inf]
	Polypedates colletti	?	1	1[inf]
	Polypedates leucomystax	?	1	1
	Polypedates macrotis	?	1	1[inf]
	Polypedates maculatus	1	1	1
	Polypedates otilophus	?	1	1
	Pseudophilautus alto	?	1	1[inf]
	Pseudophilautus amboli	1	1	1[inf]
	Pseudophilautus cavirostris	?	1	1[inf]
	Pseudophilautus decoris	1	1	1[inf]
	Psudophilautus femoralis	?	1	1[inf]
	Pseudophilautus folicola	?	1	1[inf]
	Pseudophilautus hoffmanni	1	1	1[inf]

Rhacophoridae (cont.)	*Pseudophilautus jayarami*	0	1	1[inf]
	Pseudophilautus kani	1	1	1[inf]
	Pseudophilautus leucorhinus	?	1	1[inf]
	Pseudophilautus lunatus	?	1	1[inf]
	Pseudophilautus microtympanum	?	1	1[inf]
	Pseudophilautus mooreorum	0	?	?
	Pseudophilautus ocularis	?	1	1[inf]
	Pseudophilautus papillosus	?	1	1[inf]
	Pseudophilautus pleurotaenia	?	1	1[inf]
	Pseudophilautus popularis	?	1	1[inf]
	Pseudophilautus reticulatus	?	1	1[inf]
	Pseudophilautus sarasinorum	?	1	1[inf]
	Pseudophilautus schmarda	?	1	1[inf]
	Pseudophilautus tanu	1	1	1[inf]
	Pseudophilautus zorro	?	1	1[inf]
	Raorchestes anili	?	1	1[inf]
	Raorchestes beddomii	?	1	1[inf]
	Raorchestes charius	?	1	1[inf]
	Raorchestes chlorosoma	?	1	1[inf]
	Raorchestes chotta	0	?	?
	Raorchestes chromasynchysi	?	1	1[inf]
	Raorchestes coonoorensis	?	1	1[inf]
	Raorchestes dubois	?	1	1[inf]
	Raorchestes glandulosus	0	1	1
	Raorchestes graminirupes	?	1	1[inf]
	Raorchestes griet	?	1	1[inf]
	Raorchestes luteolus	?	1	1[inf]
	Raorchestes marki	?	1	1[inf]
	Raorchestes munnarensis	?	1	1[inf]
	Raorchestes nerostagona	?	1	1[inf]
	Raorchestes ponmudi	?	1	1[inf]
	Raorchestes sushili	?	1	1[inf]
	Raorchestes tinniens	?	1	1[inf]
	Raorchestes tuberohumerus	?	1	1[inf]
	Rhacophorus annamensis	?	1	1[inf]
	Rhacophorus angulirostris	1	1	1[inf]
	Rhacophorus calcaneus	?	1	1[inf]
	Rhacophorus chenfui	0	1	1[inf]
	Rhacophorus dennysi	?	1	1
	Rhacophorus dugritei	0	1	1[inf]
	Rhacophorus dulitensis	?	1	1[inf]
	Rhacophorus fasciatus	?	1	1[inf]
	Rhacophorus gauni	?	1	1[inf]
	Rhacophorus harrisoni	1	1	1[inf]

Rhacophoridae (cont.)	*Rhacophorus maximus*	?	1	1[inf]
	Rhacophorus minimus	1	1	1[inf]
	Rhacophorus nigropalmatus	1	1	1
	Rhacophorus omeimontis	1	1	1[inf]
	Rhacophorus orlovi	1	1	1[inf]
	Rhacophorus pardalis	1	1	1[inf]
	Rhacophorus reindwartii	1	1	1[inf]
	Rhacophorus rhodopus	0	1	1[inf]
	Rhacophorus rufipes	1	1	1[inf]
	Rhacophorus schlegelii	?	1	1
	Taruga eques	?	1	1
	Taruga fastigo	?	1	1[inf]
	Taruga longinasus	?	1	1[inf]
	Theloderma asperum	1	1	1[inf]
	Theloderma corticale	?	1	1[inf]
	Theloderma moloch	?	1	1[inf]
	Theloderma stellatum	?	1	1[inf]
Rhinodermatidae	*Insuetophrynus acarpicus*	1	1	1
	Rhinoderma darwinii	1	1	1
Rhinophrynidae	*Rhinophrynus dorsalis*	0	0	0
Scaphiopodidae	*Scaphiopus couchii*	0	1	1
	Scaphiopus holbrookii	1	1	1
	Scaphiopus hurteri	1	1	1
	Spea bombifrons	0	1	1
	Spea hammondi	0	1	1
	Spea intermontana	?	1	1
	Spea multiplicata	?	?	1
Sooglossidae	*Sechellophryne gardineri*	0	0	0
	Sooglosus sechellensis	0	0	0
	Sooglosus thomasseti	0	0	0
Telmatobiidae	*Telmatobius bolivianus*	0	0	1
	Telmatobius culeus	0	0&1	1
	Telmatobius gigas	0	?	?
	Telmatobius hintoni	0	0&1	1
	Telmatobius huayra	0	0	0
	Telmatobius marmoratus	0	0	0&1[*]
	Telmatobius niger	0	0&1	0&1
	Telmatobius sibiricus	0	1	1
	Telmatobius simonsi	0	1	1
	Telmatobius truebae	0	1	1
	Telmatobius vellardi	0	0	1
	Telmatobius verrucosus	0	1	1
	Telmatobius vilamensis	0	0	0
	Telmatobius yuracare	0	0	1[*]
	Telmatobius zapahuirensis	0	1	1[inf]

**Anura not included in Pyron (2014)**				
**Family**	**Species**	**Tympanic membrane**	**Tympanic annulus**	**Columella**
Alsodidae	*Eupsophus altor*	?	?	1
Arthroleptidae	*Letodactylodon ventrimarmoratus*	0	1	1
	Leptopelis mossambicus	1	1	1
	Cardioglossa escalaerae	1	1	1
Batrachylidae	*Atelognathus nitoi*	0^{inf}	0^{inf}	0
	Atelognathus praebasalticus	0^{inf}	0^{inf}	0
	Atelognathus reverberii	0^{inf}	0^{inf}	0
	Atelognathus solitarius	0^{inf}	0^{inf}	0
	Chaltenobatrachus grandisonae	0	0	0
Brachycephalidae	*Ischnocnema nigriventris*	?	?	1
Callyptocephalellidae	*Telmatobufo australis*	0	0	0
Ceratobatrachidae	*Alcalus mariae*	?	1	1
	Cornufer bufoniformis	0	1	1
Ceratophryidae	*Ceratophrys aurita*	1	1	1
	Ceratophrys calcarata	1	1	1
	Ceratophrys cranwelli	1	1	1
Craugastoridae	*Bryophryne abramalagae*	0	0	?
	Bryophryne bakersfield	0	0	?
	Bryophryne bustamantei	0	0	?
	Bryophryne flammiventris	1	1	1^{inf}
	Bryophryne gymnotis	1	1	1^{inf}
	Bryophryne hanssaueri	0	0	?
	Bryophryne nubilosus	0	0	?
	Bryophryne zonalis	0	0	?
	Euparkerella cochranae	0^{inf}	0^{inf}	0
	Euparkerella cryptica	0^{inf}	0^{inf}	0
	Euparkerella robusta	0^{inf}	0^{inf}	0
	Euparkerella tridactyla	0^{inf}	0^{inf}	0
	Hypodactylus nigrovittatus	?	?	1
Cycloramphidae	*Cycloramphus asper*	0	1	1
	Cycloramphus dubius	?	?	1
	Cycloramphus granulosus	?	1	1
	Cycloramphus ohausi	0	1	1
	Cycloramphus stejnegeri	0	1	1
	Thoropa lutzi	?	?	1
	Thoropa petropolitana	?	?	1
Eleutherodactylidae	*Eleutherodactylus albolabris*	?	?	1
	Eleutherodactylus grandis	?	?	1
	Eleutherodactylus guttilatus	?	?	1
	Eleutherodactylus karlschmidti	?	?	1
	Eleutherodactylus leprus	?	?	1

Eleutherodactylidae (cont.)	*Eleutherodactylus pallidus*	?	?	1
	Eleutherodactylus rubrimaculatus	?	?	1
	Phrynopus montium	0[inf]	0[inf]	0
	Pristimantis festae	?	?	1
	Pristimantis ornatissimus	?	?	1
	Pristimantis variabilis	?	?	1
Hemiphractidae	*Cryptobatrachus conditus*	?	?	1
	Cryptobatrachus fuhrmanni	?	1	1
	Fritziana fissilis	?	1	1
	Fritziana goeldii	?	1	1
	Fritziana ohausi	?	1	1
	Fritziana ulei	?	1	1
Hylidae	*Duellmanohyla uranochroa*	?	1	1
	Hyla hallowellii	?	1	1
	Hyloscirtus bogotensis	1	1	1[inf]
Hylodidae	*Crossodactylus dispar*	1	1	1
	Crossodactylus grandis	?	1	1
	Crossodactylus gaudichaudii	?	1	1
	Hylodes asper	?	?	1
	Hylodes dactylocinus	1	1	1[inf]
	Hylodes glaber	?	?	1
	Hylodes lateristrigatus	?	?	1
	Hylodes magalhaesi	?	1	1
	Hylodes nasus	?	1	1
	Hylodes perplicatus	?	1	1[inf]
	Hylodes phyllodes	1	1	1[inf]
Hyperoliidae	*Callixalus pictus*	?	1	1
	Hyperolius horstockii	0	?	?
	Kassinula wittei	?	1	1
Leptodactylidae	*Crossodactylodes bokermanni*	0	0[inf]	0
	Crossodactylodes izecksohni	0	0[inf]	0
	Crossodactylodes pintoi	0	0[inf]	0
	Crossodactylodes septentrionalis	0	0[inf]	0
	Hydrolaetare schmidti	1	1	1
	Leptodactylus latinasus	1	1	1
	Leptodactylus macrosternum	?	?	1
	Leptodactylus poecilochilus	?	1	1
	Leptodactylus pustulatus	?	?	1
	Paratelmatobius lutzii	0	0[inf]	0
	Paratelmatobius mantiqueira	?	1	1[inf]
	Paratelmatobius yepiranga	1	1	1[inf]
	Physalaemus ephippifer	1	1	1
	Physalaemus maculiventris	?	?	1

Leptodactylidae (cont.)	*Physalaemus nanus*	?	?	1
	Pseudopaludicola boliviana	0	0	1
	Pseudopaludicola saltica	0	0	1
	Pseudopaludicola pusilla	?	?	1
	Rupirana cardosoi	?	?	1
Limnodynastidae	*Heleioporus albopunctatus*	?	?	1
	Heleioporus eyrei	?	?	1
	Notaden nichollsi	0	1	1
	Philoria frosti	?	?	1
Mantellidae	*Boophis rhodoscelis*	?	?	1
	Tsingymantis antitra	1	1	1[inf]
Megophryidae	*Leptobrachella mjobergi*	1	1	1[inf]
Microhylidae	*Adelastes hylonomus*	?	1	1
	Arcovomer passarelii	0	0	0
	Callulops glandulosus	1	1	1[inf]
	Chiasmocleis avilapiresae	0	1	1
	Chiasmocleis capixaba	0	1	1
	Chiasmocleis carvalhoi	0	1	1
	Chiasmocleis leucosticta	0	1	1
	Chiasmocleis schubarti	0	1	1
	Cophixalus saxatilis	?	?	1
	Ctenophryne aterrima	0	1	1
	Myersiella microps	0	1	1
	Otophryne pyburni	?	?	1
	Otophryne steyermarki	1	1	1[inf]
	Oxydactyla coggeri	?	?	1
	Stereocyclops histrio	0	1	1
	Stereocyclops incrassatus	0	1	1
	Stereocyclops parkeri	0	1	1
	Uperodon mormorata	?	1	1
Myobatrachidae	*Arenophryne rotunda*	0[inf]	0[inf]	0
	Crinia bilingua	?	?	1
	Crinia georgiana	?	1	1
	Crinia glauerti	?	?	1
	Crinia insignifera	?	?	1
	Crinia tasmaniensis	?	?	1
	Crinia subinsignifera	?	?	1
	Pseudophryne australis	0[inf]	0[inf]	0
	Pseudophryne corroboree	0[inf]	0[inf]	0
	Pseudophryne dendyi	0[inf]	0[inf]	0
	Pseudophryne douglasi	0[inf]	0[inf]	0
	Pseudophryne guentheri	0[inf]	0[inf]	0
	Pseudophryne major	0[inf]	0[inf]	0
	Pseudophryne occidentalis	0[inf]	0[inf]	0
	Pseudophryne semimarmorata	0	0	0
	Taudactylus diurnus	0	1	1

Odontobatrachidae	*Odontobatrachus natator*	0	1	1
Phrynobatrachidae	*Phrynobatrachus plicatus*	1	1	1
Pyxicephalidae	*Anhydrophryne hewitti*	1	1	1
	Arthroleptella lightfooti	1	1	1
	Ericabatrachus baleensis	?	1	1
	Nothophryne broadleyi	0	1	1
Ranidae	*Hydrophylax gracilis*	1	1	1^{inf}
	Indosylvirana temporalis	1	1	1^{inf}
	Indosylvirana flavescens	?	1	1
Ranixalidae	*Indirana temporalis*	?	1	1^{inf}
Rhacophoridae	*Pseudophilautus silus*	1	1^{inf}	1
	Theloderma pictum	?	?	1
Telmatobiidae	*Telmatobius brachydactylus*	?	?	1
	Telmatobius laticeps	?	?	1
	Telmatobius macrostomus	?	?	1
Incertae sedis (Brachycephaloidea)	*Atopophrynus syntomopus*	0^{inf}	0^{inf}	0
	Geobatrachus walkeri	0	1	1

1.4—Sources for each character scored by species. Abbreviations: BLB, Boris L. Blotto; DB, Diego Baldo; JJOS, Jhon Jairo Ospina-Sarria; JF, Julián Faivovich; KAV, Katyuscia Araujo-Vieira; MOP, Martín O. Pereyra; MT, Mariane Targino; MCW, Molly C. Womack; JSB, J. Sebastián Barrionuevo; TG, Taran Grant.

**Bufonidae included in Pyron (2014)**			
**Species**	**Tympanic membrane**	**Tympanic annulus**	**Columella**
Adenomus kelaartii	Günther, 1858; Boulenger, 1882, 1890; Manamendra-Arachchi & Pethiyagoda, 1998	Günther, 1858; Boulenger, 1882, 1890; Manamendra-Arachchi & Pethiyagoda, 1998; Meegaskumbura *et al.*, 2015	Meegaskumbura *et al.*, 2015
Amazophrynella minuta	MOP pers. obs.; Melin, 1941; McDiarmid, 1971	McDiarmid, 1971	McDiarmid, 1971
Anaxyrus americanus	Cope, 1889; Wright & Wright, 1949	Wright & Wright, 1949; Pramuk, 2006	Mendelson, 1997c, Pramuk, 2006
Anaxyrus baxteri	Smith *et al.*, 1998	Smith *et al.*, 1998	----
Anaxyrus boreas	Boulenger, 1882; Cope, 1889; Wright & Wright, 1949	Wright & Wright, 1949; Pramuk, 2006	Gaudin, 1978; Pramuk, 2006; Mendelson *et al.*, 2011
Anaxyrus californicus	Wright & Wright, 1949	Camp, 1915; Wright & Wright, 1949	----
Anaxyrus canorus	Wright & Wright, 1949	Camp, 1916; Wright & Wright, 1949	MCW pers. obs.
Anaxyrus cognatus	Cope, 1889; Wright & Wright, 1949	Wright & Wright, 1949; Pramuk, 2006	MCW pers. obs.
Anaxyrus debilis	Girard, 1854; Boulenger, 1882; Cope, 1889; Wright & Wright, 1949	Wright & Wright, 1949; Pramuk, 2006	Baldauf, 1959
Anaxyrus exsul	Wright & Wright, 1949	Wright & Wright, 1949	----
Anaxyrus fowleri	Cope, 1889; Wright & Wright, 1949	Wright & Wright, 1949; Pramuk, 2006	----
Anaxyrus hemiophrys	Cope, 1889	Cope, 1887; Wright & Wright, 1949	----
Anaxyrus houstonensis	Sanders, 1953	Sanders, 1953	MCW pers. obs.; Sanders, 1953
Anaxyrus microscaphus	Cope, 1867	Cope, 1867	MCW pers. obs.; Baldauf, 1959

Anaxyrus nelsoni	Wright & Wright, 1949	Wright & Wright, 1949	----
Anaxyrus punctatus	Cope, 1889; Wright & Wright, 1949	Cope, 1889; Wright & Wright, 1949	Baldauf, 1959; Pramuk, 2006
Anaxyrus quercicus	Cope, 1889; Wright & Wright, 1949	Pramuk, 2006	Baldauf, 1959; Pramuk, 2006
Anaxyrus retiformis	----	Barrera & Rodríguez, 2004	----
Anaxyrus speciosus	Girard, 1854	Girard, 1854	----
Anaxyrus terrestris	Cope, 1889; Wright & Wright, 1949	Pramuk, 2006	Baldauf, 1959; Pramuk, 2006
Anaxyrus woodhousii	Cope, 1867; Girard, 1854; Wright & Wright, 1949; Baldauf, 1955	Baldauf, 1955; Wright & Wright, 1949; Pramuk, 2006	Baldauf, 1955; Pramuk, 2006
Ansonia albomaculata	Inger, 1960, 1966	Inger, 1960	----
Ansonia endauensis	Grismer, 2006	Grismer, 2006	----
Ansonia fuliginea	van Kampen, 1923; Inger, 1966	van Kampen, 1923	----
Ansonia guibei	Inger, 1966	Inger, 1966	----
Ansonia hanitschi	Inger, 1960, 1966 Grismer, 2006	Inger, 1960; Grismer, 2006	----
Ansonia inthanon	Matsui *et al.*, 1998	Matsui *et al.*, 1998	----
Ansonia kraensis	Matsui *et al.*, 1998; Grismer, 2006	Matsui *et al.*, 1998; Grismer, 2006	----
Ansonia latirostra	Grismer, 2006	Grismer, 2006	----
Ansonia leptopus	Boulenger, 1882; van Kampen, 1923; Inger, 1960, 1966; Grismer, 2006	van Kampen, 1923; Inger, 1960; Grismer, 2006	----
Ansonia longidigita	Inger, 1960, 1966; Grismer, 2006	Inger, 1960; Grismer, 2006	----
Ansonia malayana	Inger, 1960, 1966; Grismer, 2006	Inger, 1960; Grismer, 2006	----
Ansonia mcgregori	Inger, 1960; Grismer, 2006	Inger, 1960; Grismer, 2006	----
Ansonia minuta	Inger, 1960, 1966; Grismer, 2006	Inger, 1960; Grismer, 2006	Tihen, 1960
Ansonia muelleri	Inger, 1960; Grismer, 2006	Inger, 1960; Grismer, 2006	MCW pers. obs.; Tihen, 1960
Ansonia penangensis	Boulenger, 1882; van Kampen, 1923; Inger, 1960, 1966; Taylor, 1962; Quah *et al.*, 2011; Grismer, 2006	Boulenger, 1882; van Kampen, 1923; Inger, 1960, 1966; Taylor, 1962; Quah *et al.*, 2011; Grismer, 2006	----
Ansonia platysoma	Inger, 1960, 1966	Inger, 1960	----

Ansonia siamensis	Matsui *et al.*, 1998; Grismer, 2006	Grismer, 2006	----
Ansonia spinulifer	van Kampen, 1923	van Kampen, 1923	----
Atelopus bomolochos	Peters, 1973; Coloma, 1997	Coloma, 1997	Coloma, 1997; Lötters *et al.*, 2011
Atelopus chiriquiensis	Jaslow & Lombard, 1996; McDiarmid, 1971	Jaslow & Lombard, 1996; McDiarmid, 1971	MCW pers. obs.; McDiarmid, 1971; Jaslow & Lombard, 1996; Lindquist & Hetherington, 1996
Atelopus flavescens	McDiarmid, 1971; Lescure & Marty, 2000	McDiarmid, 1971, 1973; Coloma, 1997	MCW pers. obs.; McDiarmid, 1971, 1973; Coloma, 1997
Atelopus franciscus	Lescure & Marty, 2000; Boistel *et al.*, 2011	Coloma, 1997 present; Boistel *et al.*, 2011 absent	MCW pers. obs.; Coloma, 1997; Boistel *et al.*, 2011
Atelopus halihelos	Peters, 1973	----	----
Atelopus ignescens	McDiarmid, 1971; Peters, 1973; Coloma *et al.*, 2000	McDiarmid, 1971; Coloma *et al.*, 2000	MCW pers. obs.; McDiarmid, 1971; Coloma *et al.*, 2000
Atelopus longirostris	Cochran & Goin, 1970	McDiarmid, 1971	MCW pers. obs.; McDiarmid, 1971
Atelopus nanay	Coloma, 2002	Coloma, 2002	Coloma, 2002
Atelopus oxapampae	Lehr *et al.*, 2008	Lehr *et al.*, 2008	Lötters *et al.*, 2011
Atelopus peruensis	Gray & Cannatella, 1985	Gray & Cannatella, 1985	Gray & Cannatella, 1985; Lötters *et al.*, 2011
Atelopus pulcher	Peters, 1973; Lötters *et al.*, 2002b	de la Riva *et al.*, 2011	MCW pers. obs.; de la Riva *et al.*, 2011; Lötters *et al.*, 2011
Atelopus seminiferus	Boulenger, 1882	Lötters *et al.*, 2011	Lötters *et al.*, 2011
Atelopus senex	McDiarmid, 1971	McDiarmid, 1971	McDiarmid, 1971
Atelopus spumarius	Cochran & Goin, 1970; McDiarmid, 1971	McDiarmid, 1971; Coloma, 1997	McDiarmid, 1971; Lindquist & Hetherington, 1996; Coloma, 1997
Atelopus spurrelli	McDiarmid, 1971	McDiarmid, 1971	McDiarmid, 1971
Atelopus tricolor	Lötters & de la Riva, 1998	----	Lötters *et al.*, 2011
Atelopus varius	Parker, 1881; Lötters *et al.*, 1998; Nussbaum & Wu, 2007	MOP pers. obs.; Parker, 1881; Nussbaum & Wu, 2007	MOP & MCW pers. obs.; Parker, 1881; Laurent, 1942; McDiarmid, 1971; Nussbaum & Wu, 2007
Atelopus zeteki	McDiarmid, 1971	McDiarmid, 1971	MCW pers. obs.; McDiarmid, 1971
Barbarophryne brongersmai	Hoogmoed, 1972	Hoogmoed, 1972	----
Bufo aspinius	Yang *et al.*, 1996	----	Yang *et al.*, 1996

Bufo bankorensis	Nussbaum & Wu, 2007	----	MCW pers. obs.; Nussbaum & Wu, 2007
Bufo bufo	Boulenger, 1882, 1897	Boulenger, 1897; Ecke, 1934	Boulenger, 1897; Ecke, 1934; Vorobieva & Smirnov, 1987
Bufo cryptotympanicus	Kou, 1984	----	----
Bufo eichwaldi	Litvinchuck *et al.*, 2008	Litvinchuck *et al.*, 2008	----
Bufo gargarizans	Liu, 1950	Liu, 1950	----
Bufo japonicus	Matsui, 1984	Yamazaki *et al.*, 2008	MCW pers. obs.;
Bufo stejnegeri	Matsui, 1980	Matsui, 1980	----
Bufo torrenticola	Matsui, 1976	Yamazaki *et al.*, 2008	----
Bufo tuberculatus	Milto & Barabanov, 2011	Milto & Barabanov, 2011	----
Bufo tuberospinius	Yang *et al.*, 1996	----	Yang *et al.*, 1996
Bufo verrucosissimus	Litvinchuck *et al.*, 2008	Litvinchuck *et al.*, 2008	----
Bufotes balearicus	----	Boettger, 1880	----
Bufotes oblongus	Stock *et al.*, 2001	Stock *et al.*, 2001	----
Bufotes pewzowi	Stock *et al.*, 2001	Stock *et al.*, 2001	----
Bufotes siculus	Stock *et al.*, 2008	Stock *et al.*, 2008	----
Bufotes variabilis	Andren & Nilson, 1979	Andren & Nilson, 1979	----
Bufotes viridis	Boulenger, 1882, 1890, 1897; Pramuk, 2006	Boulenger, 1882, 1890, 1897; Pramuk, 2006	Boulenger, 1897; Pramuk, 2006; Vorobieva & Smirnov, 1987
Capensibufo rosei	Hewitt, 1926a; du Preez & Carruthers, 2009; Tolley *et al.*, 2010	Tolley *et al.*, 2010	MCW pers. obs.; Grandison, 1980; Grandison, 1981; Tolley *et al.*, 2010
Capensibufo tradouwi	du Preez & Carruthers, 2009; Grandison, 1980; Hewitt, 1926b	du Preez & Carruthers, 2009; Hewitt, 1926b	Grandison, 1980, 1981
Churamiti maridadi	Channing & Stanley, 2002	Channing & Stanley, 2002	Channing & Stanley, 2002
Dendrophryniscus berthalutzae	Izecksohn, 1993b	----	----
Dendrophryniscus brevipollicatus	MOP pers. obs.; Cochran, 1955; Izecksohn, 1968; McDiarmid, 1971; Nussbaum & Wu, 2007	McDiarmid, 1971	MCW pers. obs.; McDiarmid, 1971; Nussbaum & Wu, 2007
Dendrophryniscus carvalhoi	Izecksohn, 1993b	----	----

Dendrophryniscus krausae	Cruz & Fusinatto, 2008	----	----
Dendrophryniscus leucomystax	Izecksohn, 1968	MOP pers. obs.	MOP pers. obs.
Dendrophryniscus proboscideus	Izecksohn, 1976	Izecksohn, 1976	----
Didynamipus sjostedti	Andersson, 1903	Grandison, 1981	MCW pers. obs.; Grandison, 1978, 1981
Duttaphrynus atukoralei	----	Bogert & Senanayake, 1966; Manamendra-Arachchi & Pethiyagoda, 1998	----
Duttaphrynus brevirostris	----	Rao, 1937	----
Duttaphrynus crocus	Wogan *et al*., 2003; Das *et al*., 2013	Wogan *et al*., 2003	----
Duttaphrynus himalayanus	Boulenger, 1882, 1890; Das *et al*., 2013	Boulenger, 1882, 1890; Das *et al*., 2013	----
Duttaphrynus hololius	Günther, 1875; Boulenger, 1882, 1890	Günther, 1875	----
Duttaphrynus melanostictus	Boulenger, 1882, 1890; van Kampen, 1923; Taylor, 1962; Inger, 1966; Manamendra-Arachchi & Pethiyagoda, 1998	Parker, 1881; Taylor, 1962; Pramuk, 2006; Das *et al*., 2013	Parker, 1881; Ramaswami, 1936; Vorobieva & Smirnov, 1987; Mendelson, 1997c; Pramuk, 2006
Duttaphrynus parietalis	Boulenger, 1882, 1890	Ramaswami, 1936	Ramaswami, 1936
Duttaphrynus scaber	Das *et al*., 2013	Das *et al*., 2013	MCW pers. obs.
Duttaphrynus stomaticus	Boulenger, 1890; Das *et al*., 2013	Boulenger, 1890; Das *et al*., 2013	MCW pers. obs.
Duttaphrynus stuarti	Wogan *et al*., 2003; Das *et al*., 2013	Das *et al*., 2013	----
Epidalea calamita	MOP pers. obs.; Boulenger, 1882, 1897	MOP pers. obs.; Boulenger, 1882, 1897	MCW pers. obs.; Parker, 1881; Boulenger, 1897; Mendelson, 1997c
Ghatophryne ornata	Günther, 1875; Boulenger, 1882; Inger, 1960; Biju *et al*., 2009	Inger, 1960; Biju *et al*., 2009	----
Incilius alvarius	Cope, 1889; Wright & Wright, 1949	Cope, 1889; Wright & Wright, 1949; Pramuk, 2006	Pramuk, 2006; Mendelson *et al*., 2011
Incilius aucoinae	O’Neill & Mendelson, 2004	O’Neill & Mendelson, 2004	Mendelson *et al*., 2011
Incilius bocourti	Brocchi, 1877; Boulenger, 1882	----	Mendelson *et al*., 2011
Incilius campbelli	Mendelson, 1994	Mendelson, 1994	MCW pers. obs.; Mendelson *et al*., 2011

Incilius canaliferus	Boulenger, 1882	Boulenger, 1882	MCW pers. obs.; Mendelson *et al.*, 2011
Incilius cavifrons	Firschein, 1950	Firschein, 1950	Mendelson *et al.*, 2011
Incilius chompipe	Vaughan & Mendelson, 2007	----	----
Incilius coccifer	Taylor, 1951; Savage, 2002; Mendelson *et al.*, 2005	Savage, 2002; Pramuk, 2006	Pramuk, 2006; Mendelson *et al.*, 2011
Incilius coniferus	Boulenger, 1882; Taylor, 1951; Cochran & Goin, 1970	Pramuk, 2006	MCW pers. obs.; Pramuk, 2006; Mendelson *et al.*, 2011
Incilius cristatus	Boulenger, 1882; Firschein, 1950; Mendelson, 1997a	Boulenger, 1882; Firschein, 1950; Mendelson, 1997a	Mendelson *et al.*, 2011
Incilius cycladen	Lynch & Smith, 1966; Mendelson *et al.*, 2005	----	----
Incilius fastidiosus	Boulenger, 1882; Savage, 1972	Savage, 1972	Mendelson *et al.*, 2011; Savage, 1972
Incilius ibarrai	Mendelson, 2001; Mendelson *et al.*, 2005	Mendelson, 2001; Mendelson *et al.*, 2005	MCW pers. obs.; Mendelson *et al.*, 2011
Incilius leucomyos	McCranie & Wilson, 2000	McCranie & Wilson, 2000	Mendelson *et al.*, 2011
Incilius luetkenii	Taylor, 1951	Pramuk, 2006	Pramuk, 2006; Mendelson *et al.*, 2011
Incilius macrocristatus	Mendelson, 1997a, b	Mendelson, 1997a, b	Mendelson, 1997a, b
Incilius marmoreus	Taylor, 1943	Taylor, 1943	Mendelson *et al.*, 2011
Incilius mazatlanensis	Taylor, 1943	Taylor, 1943	MCW pers. obs.; Mendelson *et al.*, 2011
Incilius melanochlorus	Boulenger, 1882; Taylor, 1951; Gredin, 1972	Boulenger, 1882; Taylor, 1951; Gredin, 1972	Mendelson *et al.*, 2011
Incilius nebulifer	----	----	Mendelson *et al.*, 2011
Incilius occidentalis	Santos-Barrera, 2014	Santos-Barrera, 2014	MCW pers. obs.; Mendelson *et al.*, 2011
Incilius perplexus	Taylor, 1943	----	Mendelson, 1997c; Mendelson *et al.*, 2011
Incilius pisinnus	Mendelson *et al.*, 2005	Mendelson *et al.*, 2005	----
Incilius porteri	Mendelson *et al.*, 2005	----	----
Incilius signifer	Mendelson *et al.*, 2005	Mendelson *et al.*, 2005	----

Incilius spiculatus	Mendelson, 1997a	Mendelson, 1997a	Mendelson *et al.*, 2011
Incilius tacanensis	Mendelson, 1997b	----	----
Incilius tutelarius	Mendelson, 1997b	Mendelson, 1997b	Mendelson *et al.*, 2011
Incilius valliceps	Boulenger, 1882; Cope, 1889; Taylor, 1936; Wright & Wright, 1949; Firschein, 1950; Mendelson, 1997b	Wright & Wright, 1949; Mendelson, 1997b; Pramuk, 2006	Baldauf, 1959; Pramuk, 2006; Mendelson *et al.*, 2011
Ingerophrynus biporcatus	Boulenger, 1882, 1890; van Kampen, 1923; Inger, 1966	Boulenger, 1890; van Kampen, 1923; Inger, 1966	----
Ingerophrynus celebensis	Boulenger, 1882; van Kampen, 1923	van Kampen, 1923	MCW pers. obs.
Ingerophrynus divergens	Boulenger, 1882; Inger, 1966	Inger, 1966	----
Ingerophrynus galeatus	Boulenger, 1882; Inger *et al.*, 1999	Inger *et al.*, 1999	----
Ingerophrynus macrotis	Boulenger, 1890; van Kampen, 1923; Taylor, 1962; Inger, 1966; Inger *et al.*, 1999; Das *et al.*, 2013	Boulenger, 1890; van Kampen, 1923; Taylor, 1962; Inger, 1966; Inger *et al.*, 1999; Das *et al.*, 2013	Pramuk, 2006
Ingerophrynus philippinicus	van Kampen, 1923	van Kampen, 1923	----
Leptophryne borbonica	MOP pers. obs.; Boulenger, 1882; van Kampen, 1923; Davis, 1935; Taylor, 1962; Inger, 1966	MOP pers. obs.; Boulenger, 1882; van Kampen, 1923; Davis, 1935; Taylor, 1962; Inger, 1966	Davis, 1935
Melanophryniscus devincenzii	DB pers. obs.; Klappenbach, 1968	DB pers. obs.	DB pers. obs.
Melanophryniscus fulvoguttatus	DB pers. obs.	DB pers. obs.	DB & MCW pers. obs.
Melanophryniscus klappenbachi	MOP pers. obs.; Prigioni & Langone, 2000; Nussbaum & Wu, 2007	MOP pers. obs.	MOP pers. obs.
Melanophryniscus pachyrhynus	DB pers. obs.; Caramaschi & Cruz, 2002	Baldo *et al.*, 2012	Baldo *et al.*, 2012
Melanophryniscus rubriventris	DB pers. obs.; McDiarmid, 1971; Cei, 1980	McDiarmid, 1971; Pramuk, 2006	McDiarmid, 1971; Pramuk, 2006
Melanophryniscus stelzneri	McDiarmid, 1971; Cei, 1980	McDiarmid, 1971	McDiarmid, 1971

Mertensophryne anotis	Loveridge, 1932; Poynton, 1964, 1991; Poynton & Broadley, 1988; Poynton & Clarke, 1999; du Preez & Carruthers, 2009	Poynton, 1991	Tihen, 1960; Poynton & Broadley, 1988; Poynton, 1991; Poynton & Clarke, 1999
Mertensophryne lindneri	Poynton & Broadley, 1988; Clarke, 1989	Poynton & Broadley, 1988; Clarke, 1989	Clarke, 1989
Mertensophryne loveridgei	Poynton & Clarke, 1999; Harper *et al.*, 2010	----	Poynton & Clarke, 1999
Mertensophryne micranotis	Loveridge, 1932; Harper *et al.*, 2010	----	----
Mertensophryne taitana	Boulenger, 1882; Loveridge, 1932; Schmidt & Inger, 1959; Poynton & Broadley, 1988; Harper *et al.*, 2010	Schmidt & Inger, 1959; Grandison, 1972; Poynton & Broadley, 1988	MCW pers. obs.; Grandison, 1972
Mertensophryne uzunguensis	Loveridge, 1932; Harper *et al.*, 2010	Grandison, 1972	Grandison, 1972
Nannophryne cophotis	Boulenger, 1900b, Vellard, 1959	----	Pramuk, 2006
Nannophryne variegata	MOP pers. obs.; Boulenger, 1882; Gallardo, 1962b; Cei, 1980	MOP pers. obs.; Pramuk, 2006	MOP & MCW pers. obs.; Pramuk, 2006
Nectophryne afra	Boulenger, 1882; Tihen, 1960	Grandison, 1981; Tihen, 1960	Grandison, 1978, 1981; Tihen, 1960
Nectophryne batesii	Boulenger, 1913	----	MCW pers. obs.; Grandison, 1978, 1981
Nectophrynoides minutus	Menegon *et al.*, 2004; Perret, 1972	Grandison, 1978; Menegon *et al.*, 2004	Grandison, 1978; Menegon *et al.*, 2004
Nectophrynoides tornieri	MOP pers. obs.; Perret, 1972; Menegon *et al.*, 2004	Grandison, 1978; Menegon *et al.*, 2004	Grandison, 1978, 1981; Menegon *et al.*, 2004
Nectophrynoides viviparus	Perret, 1972; Menegon *et al.*, 2004; Nussbaum & Wu, 2007; Loader *et al.*, 2009; Harper *et al.*, 2010	Perret, 1972; Grandison, 1978; Menegon *et al.*, 2004	Tihen, 1960; Grandison, 1978, 1981; Menegon *et al.*, 2004; Nussbaum & Wu, 2007
Nimbaphrynoides occidentalis	Perret, 1972; Sandberg *et al.*, 2010	Grandison, 1978	Grandison, 1978,1981
Osornophryne antisana	Hoogmoed, 1987	Hoogmoed, 1987	Hoogmoed, 1987
Osornophryne bufoniformis	Cochran & Goin, 1970; Ruiz-Carranza & Hernandez-Camacho, 1976	Ruiz-Carranza & Hernandez-Camacho, 1976	MCW pers. obs.; Ruiz-Carranza & Hernandez-Camacho, 1976; Pramuk, 2006
Osornophryne guacamayo	MOP pers. obs.	MOP pers. obs.	MOP pers. obs.

Osornophryne puruanta	Gluesenkamp & Guayasamin, 2008	----	Gluesenkamp & Guayasamin, 2008
Osornophryne sumacoensis	Gluesenkamp, 1995	----	----
Pedostibes tuberculosus	Günther, 1875; Boulenger, 1882, 1890; Dahanakur *et al.*, 2004	Boulenger, 1882	----
Pelophryne brevipes	Inger, 1966; Das, 2008	Inger, 1966	----
Pelophryne misera	van Kampen, 1923; Inger, 1966	van Kampen, 1923; Ramaswami, 1936; Inger, 1966	Ramaswami, 1936
Pelophryne signata	van Kampen, 1923; Das, 2008	van Kampen, 1923	----
Peltophryne empusa	MOP pers. obs.; Boulenger, 1882; Ruibal, 1959; Diaz & Cadiz, 2008	MOP pers. obs.; Diaz & Cadiz, 2008	----
Peltophryne fustiger	Schwartz, 1960	Schwartz, 1960	MCW pers. obs.;
Peltophryne guentheri	Boulenger, 1882; Ruibal, 1959	Ruibal, 1959	----
Peltophryne gundlachi	Ruibal, 1959	Ruibal, 1959	----
Peltophryne lemur	Ruibal, 1959	Pramuk, 2006	Pramuk, 2006
Peltophryne longinasus	Ruibal, 1959; Valdes de la Osa & Ruiz, 1980	----	----
Peltophryne peltocephala	MOP pers. obs.; Boulenger, 1882; Ruibal, 1959	MOP pers. obs.	Pramuk, 2002
Peltophryne taladai	Schwartz, 1960	Schwartz, 1960	Pramuk, 2002
Phrynoidis asper	Boulenger, 1882, 1890; van Kampen, 1923; Taylor, 1962; Inger, 1966	van Kampen, 1923; Taylor, 1962; Inger, 1966; Pramuk, 2006	Griffiths, 1954; Mendelson, 1997c; Pramuk, 2006
Phrynoidis juxtasper	Inger, 1964, 1966	Inger, 1964, 1966; Pramuk, 2006	Pramuk, 2006
Poyntonophrynus damaranus	Channing & Vences, 1999; du Preez & Carruthers, 2009	du Preez & Carruthers, 2009	----
Poyntonophrynus dombensis	Poynton, 1964; Channing & Vences, 1999; du Preez & Carruthers, 2009	Poynton, 1964; du Preez & Carruthers, 2009	----
Poyntonophrynus fenoulheti	Poynton, 1964; Poynton & Broadley, 1988	Poynton, 1964; Poynton & Broadley, 1988	----
Poyntonophrynus vertebralis	Poynton, 1964; du Preez & Carruthers, 2009	Poynton, 1964; du Preez & Carruthers, 2009	----

Rentapia hosii	MOP pers. obs.; van Kampen, 1923; Taylor, 1962; Inger, 1966	MOP pers. obs.; van Kampen, 1923; Taylor, 1962	Tihen, 1960; Pramuk, 2006
Rentapia rugosa	Inger, 1966	Inger, 1966	----
Rhaebo ecuadorensis	Mueses-Cisneros *et al.*, 2012	Mueses-Cisneros *et al.*, 2012	----
Rhaebo glaberrimus	MOP pers. obs.; Cochran & Goin, 1970; Mueses-Cisneros *et al.*, 2012	MOP pers. obs.; Mueses-Cisneros *et al.*, 2012	----
Rhaebo guttatus	MOP pers. obs.; Jimenez de la Espada, 1875; Boulenger, 1882; Melin, 1941	MOP pers. obs.; Pramuk, 2006	Pramuk, 2006
Rhaebo nasicus	Kok & Kalamandeen, 2008	Kok & Kalamandeen, 2008	----
Rhinella achavali	Maneyro *et al.*, 2004	Maneyro *et al.*, 2004	----
Rhinella amboroensis	Harvey & Smith, 1993	----	----
Rhinella arenarum	MOP pers. obs.; Gallardo, 1965; Cei, 1980	MOP pers. obs.; Pramuk, 2006	MCW pers. obs.; Pramuk, 2006
Rhinella arequipensis	Vellard, 1959;Urra, 2013	Pramuk, 2006	Pramuk, 2006
Rhinella arunco	Urra, 2013	Pramuk, 2006	Pramuk, 2006
Rhinella atacamensis	Correa-Quesada *et al.*, 2008	Pramuk, 2006	----
Rhinella castaneotica	Caldwell, 1991; Avila *et al.*, 2010	Caldwell, 1991; Avila *et al.*, 2010	MCW pers. obs.
Rhinella chavin	Lehr *et al.*, 2001	Lehr *et al.*, 2001	Pramuk & Lehr, 2005; Pramuk, 2006
Rhinella dapsilis	Myers & Carvalho, 1945	Myers & Carvalho, 1945	MCW pers. obs.
Rhinella festae	Trueb, 1971	Trueb, 1971	Trueb, 1971
Rhinella granulosa	Narvaes & Rodrigues, 2009	Narvaes & Rodrigues, 2009	----
Rhinella humboldti	Kenny, 1969; Cochran & Goin, 1970; Narvaes & Rodrigues, 2009	Pramuk, 2006	Pramuk, 2006
Rhinella icterica	Cei, 1980; Heyer *et al.*, 1990	Pramuk, 2006	MCW pers. obs.; Pramuk, 2006
Rhinella limensis	Vellard, 1959	Pramuk, 2006	Pramuk, 2006
Rhinella macrorhina	Trueb, 1971	Trueb, 1971	Trueb, 1971
Rhinella manu	Chaparro *et al.*, 2007	Chaparro *et al.*, 2007	Chaparro *et al.*, 2007
Rhinella margaritifera	Lavilla *et al.*, 2013	Lavilla *et al.*, 2013	Pramuk, 2006; Mendelson *et al.*, 2011

Rhinella marina	Boulenger, 1882; Melin, 1941; Kenny, 1969; Cochran & Goin, 1970	Pramuk, 2006	Goodrich, 1930; Baldauf, 1959; Pramuk, 2006; Mendelson *et al.*, 2011
Rhinella nesiotes	Duellman & Toft, 1979	Duellman & Toft, 1979	----
Rhinella ocellata	Leão & Cochran, 1952	Pramuk, 2006	MCW pers. obs.
Rhinella ornata	Jimenez de la Espada, 1875; Heyer *et al.*, 1990; Baldissera *et al.*, 2004	Parker, 1881; Baldissera *et al.*, 2004; Pramuk, 2006	MCW pers. obs.; Parker, 1881; Pramuk, 2006
Rhinella poeppigii	de la Riva, 2002b	de la Riva, 2002b; Pramuk, 2006	MCW pers. obs.; Pramuk, 2006
Rhinella rostrata	Cochran & Goin, 1970; Noble, 1920; Trueb, 1971	Trueb, 1971	Trueb, 1971; Trueb, 1973
Rhinella schneideri	Cei, 1980	Pramuk, 2006	MCW pers. obs.; Pramuk, 2006
Rhinella spinulosa	MOP pers. obs.; Vellard, 1959; Gallardo, 1965; Cei, 1972, 1980	MOP pers. obs.; Pramuk, 2006	MOP pers. obs.; Mendelson, 1997c; Pramuk, 2006
Rhinella vellardi	----	Pramuk, 2006; Vellard, 1959	Pramuk, 2006
Rhinella veraguensis	Boulenger, 1882; Harvey & Smith, 1993; Savage, 1969	----	Pramuk & Lehr, 2005; Pramuk, 2006
Sabahphrynus maculatus	van Kampen, 1923; Inger, 1966; Grismer, 2006; Matsui *et al.*, 2007; Matsui *et al.*, 2012	Inger, 1966 Grismer, 2006;; Matsui *et al.*, 2007	Matsui *et al.*, 2007
Schismaderma carens	MOP pers. obs.; Boulenger, 1882; Poynton, 1964; Poynton & Broadley, 1988	MOP pers. obs.; Poynton, 1964; Poynton & Broadley, 1988; Pramuk, 2006	MCW pers. obs.; Pramuk, 2006
Sclerophrys brauni	Harper *et al.*, 2010	Harper *et al.*, 2010	----
Sclerophrys garmani	Keith, 1968; Poynton & Broadley, 1988	Keith, 1968; Poynton & Broadley, 1988	----
Sclerophrys gracilipes	Boulenger, 1899	Boulenger, 1899	MCW pers. obs.
Sclerophrys gutturalis	Poynton & Broadley, 1988	Poynton & Broadley, 1988	----
Sclerophrys kisoloensis	Keith, 1968; Loveridge, 1932; Poynton & Broadley, 1988	Keith, 1968; Poynton & Broadley, 1988	----
Sclerophrys latifrons	Boulenger, 1900c	Boulenger, 1900c; Inger & Menzies, 1961; Perret & Amiet, 1971	----

Sclerophrys lemairii	Inger & Menzies, 1961; Poynton & Broadley, 1988	Inger & Menzies, 1961; Poynton & Broadley, 1988	----
Sclerophrys maculata	Perret & Amiet, 1971; Poynton & Broadley, 1988	Perret & Amiet, 1971; Poynton & Broadley, 1988	Mendelson, 1997c, Pramuk, 2006
Sclerophrys mauritanica	Boulenger, 1882	Boulenger, 1882	MCW pers. obs.
Sclerophrys pantherina	Parker, 1981	Parker, 1981	Parker, 1981
Sclerophrys pardalis	du Preez & Carruthers, 2009	Pramuk, 2006; du Preez & Carruthers, 2009	----
Sclerophrys poweri	du Preez & Carruthers, 2009	du Preez & Carruthers, 2009	----
Sclerophrys regularis	Boulenger, 1882; Sedra & Michael, 1959; Inger & Menzies, 1961; Poynton, 1964	Sedra & Michael, 1959; Inger & Menzies, 1961	Sedra & Michael, 1959; Pramuk, 2006
Sclerophrys steindachneri	Pfeffer, 1893	Pfeffer, 1893	----
Sclerophrys tuberosa	Boulenger, 1882	Boulenger, 1882	MCW pers. obs.
Sclerophrys xeros	Tandy *et al.*, 1976	Pramuk, 2006; Channing *et al.*, 2012	Pramuk, 2006
Strauchbufo raddei	Boulenger, 1882	Vorobieva & Smirnov, 1987	MCW pers. obs.; Vorobieva & Smirnov, 1987
Vandijkophrynus amatolicus	du Preez & Carruthers, 2009	du Preez & Carruthers, 2009	----
Vandijkophrynus angusticeps	MOP pers. obs.; Boulenger, 1882; du Preez & Carruthers, 2009	Schoonees, 1930; du Preez & Carruthers, 2009	MCW pers. obs.; Schoonees, 1930
Vandijkophrynus gariepensis	du Preez & Carruthers, 2009	du Preez & Carruthers, 2009	MCW pers. obs.
Vandijkophrynus inyangae	Poynton & Broadley, 1988; du Preez & Carruthers, 2009	Poynton & Broadley, 1988; du Preez & Carruthers, 2009	----
Vandijkophrynus robinsoni	du Preez & Carruthers, 2009	du Preez & Carruthers, 2009	----
Werneria bambutensis	Rödel *et al.*, 2004	----	----
Werneria mertensiana	Rödel *et al.*, 2004	----	MCW pers. obs.
Werneria tandyi	Rödel *et al.*, 2004	----	----
Wolterstorffina parvipalmata	Tihen, 1960; Perret, 1972; Boistel & Amiet, 2001	----	MCW pers. obs.; Grandison, 1978, 1981; Tihen, 1960
Xanthophryne koynayensis	Biju *et al.*, 2009	Biju *et al.*, 2009	----

Bufonidae not included in Pyron (2014)			
**Species**	**Tympanic membrane**	**Tympanic annulus**	**Columella**
Adenomus kandianus	Günther, 1872; Manamendra-Arachchi & Pethiyagoda, 1998; Gabadage *et al.*, 2014	Günther, 1872; Manamendra-Arachchi & Pethiyagoda, 1998; Gabadage *et al.*, 2014; Meegaskumbura *et al.*, 2015	Meegaskumbura *et al.*, 2015
Altiphrynoides malcolmi	----	Grandison, 1978	Grandison, 1978, 1981
Altiphrynoides osgoodi	Loveridge, 1932	Grandison, 1978	Grandison, 1978, 1981
Amazophrynella amazonicola	Rojas *et al.*, 2015	----	----
Amazophrynella bokermanni	Izecksohn, 1993a	----	----
Amazophrynella javierbustamantei	Rojas *et al.*, 2016	----	----
Amazophrynella manaos	Rojas *et al.*, 2014	----	----
Amazophrynella matses	Rojas *et al.*, 2015	----	----
Amazophrynella vote	Avila *et al.*, 2012	----	----
Anaxyrus compactilis	Cope, 1889; Brocchi, 1877; Wright & Wright, 1949	Cope, 1889; Wright & Wright, 1949	----
Anaxyrus kelloggi	----	Taylor, 1936	----
Anaxyrus mexicanus	Brocchi, 1879	Brocchi, 1879	----
Ansonia echinata	Inger & Stuebing, 2009	Inger & Stuebing, 2009	----
Ansonia glandulosa	Iskandar & Mumpuni, 2004	Iskandar & Mumpuni, 2004	----
Ansonia jeetsukumarani	Wood *et al.*, 2008	Wood *et al.*, 2008	----
Ansonia latidisca	Inger, 1966; Matsui *et al.*, 2012	Matsui *et al.*, 2012	Matsui *et al.*, 2012
Ansonia latiffi	Wood *et al.*, 2008	Wood *et al.*, 2008	----
Ansonia lumut	Chan *et al.*, 2014	Chan *et al.*, 2014	----
Ansonia thinthinae	Wilkinson *et al.*, 2012	Wilkinson *et al.*, 2012	----
Ansonia vidua	Hertwig *et al.*, 2014	Hertwig *et al.*, 2014	----
Atelopus angelito	Ardila-Robayo & Ruiz-Carranza, 1998	Ardila-Robayo & Ruiz-Carranza, 1998	Ardila-Robayo & Ruiz-Carranza, 1998
Atelopus ardila	Coloma *et al.*, 2010	Coloma *et al.*, 2010	Coloma *et al.*, 2010
Atelopus arthuri	Peters, 1973; Coloma, 1997	Coloma, 1997	Coloma, 1997
Atelopus balios	Peters, 1973; Coloma, 1997	Coloma, 1997	Coloma, 1997

Atelopus barbotini	Lescure & Marty, 2000	----	Lötters *et al.*, 2011
Atelopus boulengeri	McDiarmid, 1971; Peters, 1973	McDiarmid, 1971	McDiarmid, 1971
Atelopus carbonerensis	Rivero, 1974	Coloma, 1997	Coloma, 1997
Atelopus carrikeri	Ruthven, 1916; McDiarmid, 1971; Ruiz-Carranza *et al.*, 1994	McDiarmid, 1971; Ruiz-Carranza *et al.*, 1994	McDiarmid, 1971; Ruiz-Carranza *et al.*, 1994;
Atelopus certus	McDiarmid, 1971	McDiarmid, 1971	McDiarmid, 1971
Atelopus chirripoensis	Savage & Bolaños, 2009	Savage & Bolaños, 2009	----
Atelopus chrysocorallus	La Marca, 1996 "1994"	----	----
Atelopus coynei	Miyata, 1980; Coloma, 1997	Coloma, 1997	Coloma, 1997
Atelopus cruciger	McDiarmid, 1971; Lötters *et al.*, 2004	MOP pers. obs.; McDiarmid, 1971; Lötters *et al.*, 2004	MOP pers. obs.; McDiarmid, 1971; Lötters *et al.*, 2004
Atelopus dimorphus	Lötters, 2003	Lötters, 2003	Lötters, 2003
Atelopus ebenoides	Cochran & Goin, 1970; McDiarmid, 1971	McDiarmid, 1971	McDiarmid, 1971
Atelopus elegans	McDiarmid, 1971; Peters, 1973	McDiarmid, 1971	McDiarmid, 1971
Atelopus epikeisthos	Lötters *et al.*, 2005 "2004"	----	----
Atelopus eusebianus	Rivero & Granados Diaz, 1983	Rivero & Granados Diaz, 1983	Rivero & Granados Diaz, 1983
Atelopus eusebiodiazi	Venegas *et al.*, 2008	Venegas *et al.*, 2008	----
Atelopus exiguus	McDiarmid, 1971; Coloma *et al.*, 2000	McDiarmid, 1971; Coloma *et al.*, 2000	McDiarmid, 1971; Coloma *et al.*, 2000
Atelopus farci	Lynch, 1993	Lynch, 1993	Lynch, 1993
Atelopus gigas	Coloma *et al.*, 2010	Coloma *et al.*, 2010	Coloma *et al.*, 2010
Atelopus glyphus	McDiarmid, 1971	McDiarmid, 1971	McDiarmid, 1971
Atelopus guanujo	Coloma, 2002	Coloma, 2002	Coloma, 2002
Atelopus guitarraensis	Osorno-Muñoz *et al.*, 2001	Osorno-Muñoz *et al.*, 2001	Osorno-Muñoz *et al.*, 2001
Atelopus hoogmoedi	Lescure & Marty, 2000	----	Lötters *et al.*, 2011
Atelopus laetissimus	Ruiz-Carranza *et al.*, 1994	Ruiz-Carranza *et al.*, 1994	Ruiz-Carranza *et al.*, 1994
Atelopus limosus	Ibañez *et al.*, 1995	Ibañez *et al.*, 1995	
Atelopus loettersi	de la Riva *et al.*, 2011	de la Riva *et al.*, 2011	de la Riva *et al.*, 2011
Atelopus longibrachius	Rivero, 1963; Coloma, 1997	Coloma, 1997	Coloma, 1997
Atelopus lozanoi	Osorno-Muñoz *et al.*, 2001	Osorno-Muñoz *et al.*, 2001	Osorno-Muñoz *et al.*, 2001

Atelopus lynchi	Cannatella, 1981	Cannatella, 1981	Cannatella, 1981
Atelopus mandingues	Osorno-Muñoz *et al.*, 2001	Osorno-Muñoz *et al.*, 2001	Osorno-Muñoz *et al.*, 2001
Atelopus marinkellei	Cochran & Goin, 1970	----	----
Atelopus mindoensis	Peters, 1973; Coloma, 1997	Coloma, 1997	Coloma, 1997
Atelopus mittermeieri	Acosta Galvis *et al.*, 2006	Acosta Galvis *et al.*, 2006	----
Atelopus monohernandezii	Ardila-Robayo *et al.*, 2002	Ardila-Robayo *et al.*, 2002	Ardila-Robayo *et al.*, 2002
Atelopus mucubajiensis	Rivero, 1974; Coloma, 1997	Coloma, 1997	Coloma, 1997
Atelopus muisca	Rueda-Almonacid & Hoyos, 1992 "1991"	----	Hoyos *et al.*, 2015
Atelopus nahumae	Ruiz-Carranza *et al.*, 1994	Ruiz-Carranza *et al.*, 1994	Ruiz-Carranza *et al.*, 1994
Atelopus nepiozomus	Peters, 1973	----	----
Atelopus nicefori	Rivero, 1963; Cochran & Goin, 1970; Coloma, 1997	Coloma, 1997	Coloma, 1997
Atelopus nocturnus	Bravo-Valencia & Rivera-Correa, 2011	Bravo-Valencia & Rivera-Correa, 2011	Bravo-Valencia & Rivera-Correa, 2011
Atelopus onorei	Coloma *et al.*, 2007	Coloma *et al.*, 2007	----
Atelopus orcesi	Coloma *et al.*, 2010	Coloma *et al.*, 2010	Coloma *et al.*, 2010
Atelopus oxyrhynchus	McDiarmid, 1971; Rivero, 1974	McDiarmid, 1971	McDiarmid, 1971
Atelopus pachydermus	McDiarmid, 1971; Peters, 1973; Coloma *et al.*, 2007	McDiarmid, 1971	McDiarmid, 1971
Atelopus palmatus	Peters, 1973	----	----
Atelopus pastuso	Coloma *et al.*, 2010	Coloma *et al.*, 2010	Coloma *et al.*, 2010
Atelopus patazensis	Venegas *et al.*, 2008	Venegas *et al.*, 2008	----
Atelopus pedimarmoratus	Cochran & Goin, 1970	Cochran & Goin, 1970	----
Atelopus petersi	Coloma *et al.*, 2007	Coloma *et al.*, 2007	Coloma *et al.*, 2007
Atelopus petriruizi	Ardila-Robayo, 1999	Ardila-Robayo, 1999	Ardila-Robayo, 1999
Atelopus pictiventris	Kattan, 1986	----	----
Atelopus planispina	Jimenez de la Espada, 1875; Peters, 1973; Coloma, 1997	Coloma, 1997	Coloma, 1997
Atelopus podocarpus	Coloma *et al.*, 2010	Coloma *et al.*, 2010	Coloma *et al.*, 2010
Atelopus pyrodactylus	Venegas & Barrio, 2006	----	----

Atelopus reticulatus	Lötters *et al.*, 2002a	----	----
Atelopus sanjosei	Rivero & Serna, 1989; Coloma, 1997	Coloma, 1997	Coloma, 1997
Atelopus sernai	Ruiz-Carranza & Osorno-Muñoz, 1994	Ruiz-Carranza & Osorno-Muñoz, 1994	Ruiz-Carranza & Osorno-Muñoz, 1994
Atelopus simulatus	Ruiz-Carranza & Osorno-Muñoz, 1994	Ruiz-Carranza & Osorno-Muñoz, 1994	Ruiz-Carranza & Osorno-Muñoz, 1994
Atelopus siranus	Lötters & Henzl, 2000	Lötters & Henzl, 2000	----
Atelopus sonsonensis	Vélez-Rodríguez & Ruiz-Carranza, 1997	Vélez-Rodríguez & Ruiz-Carranza, 1997	Vélez-Rodríguez & Ruiz-Carranza, 1997
Atelopus sorianoi	La Marca, 1983; Coloma, 1997	Coloma, 1997	Coloma, 1997
Atelopus subornatus	Coloma, 1997	Coloma, 1997	Coloma, 1997
Atelopus tamaense	La Marca *et al.*, 1990 "1989"	----	----
Atelopus vogli	Lötters *et al.*, 2004	Lötters *et al.*, 2004	Lötters *et al.*, 2004
Atelopus walkeri	Rivero, 1963; Cochran & Goin, 1970; McDiarmid, 1971	McDiarmid, 1971	McDiarmid, 1971
Blythophryne beryet	Chandramouli *et al.*, 2016	Chandramouli *et al.*, 2016	Chandramouli *et al.*, 2016
Bufo ailaoanus	Kou, 1984	----	----
Bufo pageoti	Wogan *et al.*, 2003; Das *et al.*, 2013	Wogan *et al.*, 2003; Das *et al.*, 2013	----
Bufoides meghalayana	Pillai & Yazdani, 1973	----	----
Bufotes latastii	Boulenger, 1882, 1890; Stock *et al.*, 2001	Stock *et al.*, 2001	----
Bufotes luristanicus	Stock *et al.*, 2001c, Javari & Torki, 2009	Stock *et al.*, 2001c, Javari & Torki, 2009	----
Bufotes pseudoraddei	----	Stock *et al.*, 1999; Stock *et al.*, 2001	----
Bufotes surdus	Stock *et al.*, 2001	----	----
Bufotes turanensis	Stock *et al.*, 2001	Stock *et al.*, 2001	----
Bufotes zugmayeri	Stock *et al.*, 2001	Stock *et al.*, 2001	----
Dendrophryniscus oreites	Recoder *et al.*, 2010	----	----
Dendrophryniscus organensis	Carvalho-e-Silva *et al.*, 2010	----	----
Dendrophryniscus skuki	Caramaschi, 2012	----	----

Dendrophryniscus stawiarskyi	Izecksohn, 1993b	----	----
Duttaphrynus beddomii	Günther, 1875; Boulenger, 1882, 1890	Günther, 1875; Boulenger, 1882, 1890	----
Duttaphrynus chandai	Das *et al.*, 2013	Das *et al.*, 2013	----
Duttaphrynus kiphirensis	Das *et al.*, 2013	Das *et al.*, 2013	----
Duttaphrynus kotagamai	Fernando *et al.*, 1994; Manamendra-Arachchi & Pethiyagoda, 1998	Fernando *et al.*, 1994	----
Duttaphrynus mamitensis	Das *et al.*, 2013	Das *et al.*, 2013	----
Duttaphrynus manipurensis	Mathew & Sen, 2009	Mathew & Sen, 2009	----
Duttaphrynus microtympanum	----	Boulenger, 1882, 1890; Shah & Gruber, 1994; Manamendra-Arachchi & Pethiyagoda, 1998	----
Duttaphrynus mizoramensis	Das *et al.*, 2013	Das *et al.*, 2013	----
Duttaphrynus nagalandensis	Das *et al.*, 2013	Das *et al.*, 2013	----
Duttaphrynus noellerti	Manamendra-Arachchi & Pethiyagoda, 1998	Manamendra-Arachchi & Pethiyagoda, 1998	----
Duttaphrynus olivaceus	Boulenger, 1882, 1890	----	----
Duttaphrynus sumatranus	Boulenger, 1882; Teynie *et al.*, 2010; Das *et al.*, 2013	Das *et al.*, 2013	----
Duttaphrynus totol	Teynie *et al.*, 2010	Teynie *et al.*, 2010	----
Duttaphrynus valhallae	Meade-Waldo, 1909; van Kampen, 1923	Meade-Waldo, 1909; van Kampen, 1923	----
Duttaphrynus wokhaensis	Boulenger, 1890	Boulenger, 1890	----
Frostius erythrophthalmus	Pimenta & Caramaschi, 2007	----	----
Frostius pernambucensis	MOP pers. obs.; Cannatella, 1986	MOP pers. obs.	Cannatella, 1986
Ghatophryne rubigina	Pillai & Pattabiraman, 1981	----	----
Incilius aurarius	Mendelson *et al.*, 2012	Mendelson *et al.*, 2012	----
Incilius epioticus	Boulenger, 1882; Savage & Kluge, 1961; Savage, 1972; Vaughan & Mendelson, 2007	Savage & Kluge, 1961; Savage, 1972	Savage & Kluge, 1961; Savage, 1972; Pramuk, 2006; Mendelson *et al.*, 2011
Incilius guanacaste	Vaughan & Mendelson, 2007	----	----

Incilius holdridgei	Savage, 1972	Savage, 1972	Savage, 1972
Incilius intermedius	Boulenger, 1882	Boulenger, 1882	----
Incilius karenlipsae	Mendelson & Mulcahy, 2010	Mendelson & Mulcahy, 2010	----
Incilius majordomus	Savage *et al.*, 2013	Savage *et al.*, 2013	Savage *et al.*, 2013
Incilius mccoyi	Santos-Barrera & Flores-Villela, 2011	Santos-Barrera & Flores-Villela, 2011	----
Incilius periglenes	Savage, 1972; Savage, 2002	Savage, 1972; Savage, 2002	Savage, 1972; Savage, 2002
Incilius peripatetes	Taylor, 1951; Savage, 1972	Taylor, 1951; Savage, 1972	Savage, 1972
Ingerophrynus claviger	Boulenger, 1882; van Kampen, 1923	van Kampen, 1923	----
Ingerophrynus gollum	Grismer, 2007	----	----
Ingerophrynus kumquat	Grismer, 2007	----	----
Ingerophrynus parvus	van Kampen, 1923; Taylor, 1962	van Kampen, 1923; Taylor, 1962	----
Ingerophrynus quadriporcatus	van Kampen, 1923; Inger, 1966	van Kampen, 1923; Inger, 1966	----
Laurentophryne parkeri	Tihen, 1960	Tihen, 1960	Tihen, 1960; Grandison, 1978, 1981
Leptophryne cruentata	van Kampen, 1923	van Kampen, 1923	----
Melanophryniscus admirabilis	di Bernardo *et al.*, 2006	----	----
Melanophryniscus alipioi	Langone *et al.*, 2008	----	----
Melanophryniscus atroluteus	DB pers. obs.	DB pers. obs.	DB pers. obs.
Melanophryniscus biancae	Bornschein *et al.*, 2015	----	----
Melanophryniscus cambaraensis	DB pers. obs.	----	----
Melanophryniscus cupreuscapularis	DB pers. obs.; Cespedez & Alvarez, 2000	----	----
Melanophryniscus dorsalis	DB pers. obs.	----	----
Melanophryniscus estebani	DB pers. obs.; Cespedez, 2008	----	----
Melanophryniscus krauczuki	Baldo & Basso, 2004	Baldo & Basso, 2004	Baldo & Basso, 2004
Melanophryniscus langonei	Maneyro *et al.*, 2008	----	----
Melanophryniscus macrogranulosus	DB pers. obs.	DB pers. obs.	DB pers. obs.
Melanophryniscus milanoi	Bornschein *et al.*, 2015	----	----
Melanophryniscus montevidensis	DB pers. obs.	DB pers. obs.	DB pers. obs.

Melanophryniscus moreirae	Badenhorst, 1945; Cochran, 1955; McDiarmid, 1971	Badenhorst, 1945; McDiarmid, 1971	Badenhorst, 1945; McDiarmid, 1971; Mendelson, 1997c
Melanophryniscus paraguayensis	Cespedez & Motte, 2007	----	----
Melanophryniscus peritus	Caramaschi & Cruz, 2011	----	----
Melanophryniscus sanmartini	Klappenbach, 1968	----	----
Melanophryniscus setiba	MOP pers. obs.; Peloso *et al.*, 2012	Peloso *et al.*, 2012	Peloso *et al.*, 2012
Melanophryniscus simplex	DB pers. obs.; Caramaschi & Cruz, 2002	----	----
Melanophryniscus spectabilis	DB pers. obs.; Caramaschi & Cruz, 2002	----	----
Melanophryniscus tumifrons	McDiarmid, 1971; Caramaschi & Cruz, 2002	McDiarmid, 1971	McDiarmid, 1971
Melanophryniscus vilavelhensis	DB pers. obs.	----	----
Melanophryniscus xanthostomus	Bornschein *et al.*, 2015	----	----
Mertensophryne howelli	Poynton & Clarke, 1999	----	Poynton & Clarke, 1999
Mertensophryne lonnbergi	Loveridge, 1932; Poynton & Broadley, 1988	Grandison, 1972; Poynton & Broadley, 1988	Grandison, 1972
Mertensophryne melanopleura	Schmidt & Inger, 1959; Poynton & Broadley, 1988	Poynton & Broadley, 1988	----
Mertensophryne mocquardi	Loveridge, 1932	----	----
Mertensophryne nairobiensis	Loveridge, 1932	----	----
Mertensophryne nyikae	Loveridge, 1953	----	----
Mertensophryne schmidti	Grandison, 1972	Grandison, 1972	Grandison, 1972
Mertensophryne usambarae	Poynton & Clarke, 1999; Harper *et al.*, 2010	----	Poynton & Clarke, 1999
Metaphryniscus sosai	Señaris *et al.*, 1994	Señaris *et al.*, 1994	----
Nannophryne apolobambica	de la Riva *et al.*, 2005	de la Riva *et al.*, 2005	----
Nannophryne corynetes	Duellman & Ochoa, 1991	Duellman & Ochoa, 1991	Duellman & Ochoa, 1991; Pramuk, 2006
Nectophrynoides asperginis	Arch *et al.*, 2011	----	Arch *et al.*, 2011
Nectophrynoides cryptus	Perret, 1972	Grandison, 1978	Perret, 1972; Grandison, 1978, 1981
Nectophrynoides frontierei	Menegon *et al.*, 2004	Menegon *et al.*, 2004	Menegon *et al.*, 2004

Nectophrynoides laevis	Menegon *et al.*, 2004	----	----
Nectophrynoides laticeps	Harper *et al.*, 2010	Harper *et al.*, 2010	----
Nectophrynoides paulae	Menegon *et al.*, 2007	Menegon *et al.*, 2007	----
Nectophrynoides poyntoni	Menegon *et al.*, 2004	Menegon *et al.*, 2004	Menegon *et al.*, 2004
Nectophrynoides pseudotornieri	Menegon *et al.*, 2004	----	----
Nectophrynoides vestergaardi	Menegon *et al.*, 2004	Menegon *et al.*, 2004	Menegon *et al.*, 2004
Nectophrynoides wendyae	Menegon *et al.*, 2004	----	----
Oreophrynella cryptica	Señaris, 1995 "1993"	----	----
Oreophrynella dendronastes	Lathrop & MacCulloch, 2007	----	----
Oreophrynella huberi	Diego-Aransay & Gorzula, 1990	----	----
Oreophrynella nigra	Señaris *et al.*, 1994	----	----
Oreophrynella quelchii	Boulenger, 1895; McDiarmid, 1971; Nussbaum & Wu, 2007	McDiarmid, 1971; Nussbaum & Wu, 2007	McDiarmid, 1971; Nussbaum & Wu, 2007
Oreophrynella seegobini	Kok, 2009	----	----
Oreophrynella vasquezi	Señaris *et al.*, 1994	----	----
Oreophrynella weiassipuensis	Señaris *et al.*, 2005	----	----
Osornophryne angel	Yanez-Munoz *et al.*, 2011	----	----
Osornophryne cofanorum	Mueses-Cisneros *et al.*, 2010	----	----
Osornophryne occidentalis	Cisneros-Heredia & Gluesenkamp, 2010	Cisneros-Heredia & Gluesenkamp, 2010	----
Osornophryne percrassa	Ruiz-Carranza & Hernandez-Camacho, 1976	Ruiz-Carranza & Hernandez-Camacho, 1976	Ruiz-Carranza & Hernandez-Camacho, 1976
Osornophryne simpsoni	Páez-Moscoso *et al.*, 2011	Páez-Moscoso *et al.*, 2011	Páez-Moscoso *et al.*, 2011
Osornophryne talipes	MOP pers. obs.	----	----
Parapelophryne scalpta	Liu *et al.*, 1973	Fei *et al.*, 2003	Fei *et al.*, 2003
Pedostibes kempi	Boulenger, 1919	----	----
Pelophryne albotaeniata	Tihen, 1960; Das, 2008	Tihen, 1960	Tihen, 1960
Pelophryne api	Das, 2008	Das, 2008	----
Pelophryne guentheri	Boulenger, 1882; van Kampen, 1923; Inger, 1966; Das, 2008	Boulenger, 1882; van Kampen, 1923; Inger, 1966	----
Pelophryne lighti	Das, 2008	Das, 2008	----

Pelophryne linanitensis	Das, 2008	Das, 2008	----
Pelophryne murudensis	Das, 2008	Das, 2008	----
Pelophryne rhopophilia	Das, 2008	Das, 2008	----
Pelophryne saravacensis	Inger & Stuebing, 2009	Inger & Stuebing, 2009	----
Peltophryne cataulaciceps	Schwartz, 1959	Schwartz, 1959	----
Peltophryne florentinoi	Moreno & Rivalta, 2007	Moreno & Rivalta, 2007	----
Peltophryne fluviatica	----	Schwartz, 1972	----
Peltophryne fracta	----	Schwartz, 1972	----
Poyntonophrynus beiranus	Loveridge, 1932; Poynton & Broadley, 1988; du Preez & Carruthers, 2009	Poynton & Broadley, 1988; du Preez & Carruthers, 2009	----
Poyntonophrynus grandisonae	Poynton & Haacke, 1993	Poynton & Haacke, 1993	Poynton & Haacke, 1993
Poyntonophrynus hoeschi	Poynton, 1964; du Preez & Carruthers, 2009	du Preez & Carruthers, 2009	----
Poyntonophrynus kavangensis	Poynton & Broadley, 1988; Channing & Vences, 1999; du Preez & Carruthers, 2009	Poynton & Broadley, 1988; du Preez & Carruthers, 2009	----
Poyntonophrynus lughensis	Loveridge, 1932	----	----
Poyntonophrynus parkeri	----	Loveridge, 1932; Poynton *et al.*, 2005	----
Pseudobufo subasper	Boulenger, 1882; van Kampen, 1923; Inger, 1966	van Kampen, 1923; Inger, 1966	Tihen, 1960
Rentapia everetti	van Kampen, 1923; Inger, 1966	van Kampen, 1923	----
Rhaebo andinophrynoides	MOP pers. obs.; Mueses-Cisneros, 2009	MOP pers. obs.; Mueses-Cisneros, 2009	----
Rhaebo atelopoides	Lynch & Ruiz-Carranza, 1981	Lynch & Ruiz-Carranza, 1981	Lynch & Ruiz-Carranza, 1981
Rhaebo blombergi	Myers & Funkhouser, 1951; Cochran & Goin, 1970	Pramuk, 2006	Pramuk, 2006
Rhaebo caeruleostictus	Boulenger, 1882; Hoogmoed, 1989; Pramuk & Kadivar, 2003	Hoogmoed, 1989; Pramuk, 2006	Hoogmoed, 1989; Pramuk, 2006
Rhaebo colomai	Hoogmoed, 1985; Ron *et al.*, 2015	Hoogmoed, 1985; Ron *et al.*, 2015	Hoogmoed, 1985

Rhaebo haematiticus	MOP pers. obs.; Boulenger, 1882; Cochran & Goin, 1970; Pramuk, 2006; Mueses-Cisneros, 2009	MOP pers. obs.; Pramuk, 2006	MOP pers. obs.; Pramuk, 2006; Mendelson *et al.*, 2011
Rhaebo hypomelas	Hoogmoed, 1989; Mueses-Cisneros, 2009	Hoogmoed, 1989; Mueses-Cisneros, 2009	----
Rhaebo lynchi	Mueses-Cisneros, 2007	----	----
Rhaebo olallai	Hoogmoed, 1985; Ron *et al.*, 2015	Hoogmoed, 1985; Ron *et al.*, 2015	Hoogmoed, 1985
Rhinella abei	Baldissera *et al.*, 2004	Baldissera *et al.*, 2004	----
Rhinella achalensis	Cei, 1972, 1980	MOP pers. obs.; Cei, 1972	MOP pers. obs.
Rhinella acrolopha	Trueb, 1971	Trueb, 1971	Trueb, 1971
Rhinella acutirostris	Lötters & Köhler, 2000	Lötters & Köhler, 2000	----
Rhinella alata	Santos *et al.*, 2015	Santos *et al.*, 2015	----
Rhinella amabilis	Pramuk & Kadivar, 2003	Pramuk & Kadivar, 2003; Pramuk, 2006	Pramuk & Kadivar, 2003; Pramuk, 2006
Rhinella arborescandens	Duellman & Schulte, 1992	Duellman & Schulte, 1992	Duellman & Schulte, 1992
Rhinella azarai	Narvaes & Rodrigues, 2009	Narvaes & Rodrigues, 2009	----
Rhinella bergi	Narvaes & Rodrigues, 2009	Narvaes & Rodrigues, 2009	----
Rhinella bernardoi	Sanabria *et al.*, 2010	Sanabria *et al.*, 2010	----
Rhinella casconi	Roberto *et al.*, 2014	Roberto *et al.*, 2014	----
Rhinella centralis	Narvaes & Rodrigues, 2009	Narvaes & Rodrigues, 2009	----
Rhinella ceratophrys	Boulenger, 1882; Cochran & Goin, 1970; Fenolio *et al.*, 2012	Fenolio *et al.*, 2012	----
Rhinella cerradensis	Maciel *et al.*, 2007	Maciel *et al.*, 2007	----
Rhinella chrysophora	McCranie *et al.*, 1989	McCranie *et al.*, 1989	McCranie *et al.*, 1989; Pramuk & Lehr, 2005
Rhinella cristinae	Vélez-Rodríguez & Ruiz-Carranza, 2002	Vélez-Rodríguez & Ruiz-Carranza, 2002	Vélez-Rodríguez & Ruiz-Carranza, 2002
Rhinella crucifer	Baldissera *et al.*, 2004	Baldissera *et al.*, 2004	Mendelson, 1997c
Rhinella diptycha	----	Cope, 1862a	----
Rhinella dorbignyi	MOP pers. obs.; Boulenger, 1882; Cei, 1980; Narvaes & Rodrigues, 2009	MOP pers. obs.	----

Rhinella fernandezae	MOP pers. obs.; Cei, 1980; Narvaes & Rodrigues, 2009	MOP pers. obs.	----
Rhinella fissipes	Boulenger, 1903b, Gallardo, 1967	----	----
Rhinella gallardoi	Carrizo, 1992	Carrizo, 1992	----
Rhinella gildae	Vaz-Silva *et al.*, 2015	Vaz-Silva *et al.*, 2015	----
Rhinella gnustae	Gallardo, 1967; Cei, 1980	Gallardo, 1967; Cei, 1980	----
Rhinella henseli	Baldissera *et al.*, 2004	Baldissera *et al.*, 2004	----
Rhinella hoogmoedi	Caramaschi & Pombal, 2006	Caramaschi & Pombal, 2006	----
Rhinella inca	Gallardo, 1967	Gallardo, 1967	----
Rhinella inopina	Vaz-Silva *et al.*, 2012	Vaz-Silva *et al.*, 2012	----
Rhinella iserni	Jimenez de la Espada, 1875	Jimenez de la Espada, 1875	Jimenez de la Espada, 1875
Rhinella jimi	Stevaux, 2002	Stevaux, 2002	----
Rhinella justinianoi	Harvey & Smith, 1994	----	----
Rhinella leptoscelis	Vellard, 1959; Gallardo, 1967; Padial *et al.*, 2009	Vellard, 1959; Gallardo, 1967; Padial *et al.*, 2009	----
Rhinella lescurei	Fouquet *et al.*, 2007	Fouquet *et al.*, 2007	----
Rhinella lindae	Rivero & Castaño, 1990	Rivero & Castaño, 1990	Rivero & Castaño, 1990
Rhinella magnussoni	Lima *et al.*, 2007	Lima *et al.*, 2007	----
Rhinella major	MOP pers. obs.; Cei, 1980; Narvaes & Rodrigues, 2009	MOP pers. obs.; Pramuk, 2006	Pramuk, 2006
Rhinella martyi	Fouquet *et al.*, 2007	Fouquet *et al.*, 2007	----
Rhinella merianae	Narvaes & Rodrigues, 2009	Narvaes & Rodrigues, 2009	MCW pers. obs.
Rhinella mirandaribeiroi	Narvaes & Rodrigues, 2009	Pramuk, 2006	Pramuk, 2006
Rhinella multiverrucosa	Lehr *et al.*, 2005	Lehr *et al.*, 2005	Lehr *et al.*, 2005; Pramuk, 2006
Rhinella nattereri	Narvaes & Rodrigues, 2009	Narvaes & Rodrigues, 2009	----
Rhinella nicefori	Cochran & Goin, 1970; Trueb, 1971	Trueb, 1971	Trueb, 1971, 1973
Rhinella paraguas	Grant & Bolivar-G, 2014	Grant & Bolivar-G, 2014	Grant & Bolivar-G, 2014
Rhinella paraguayensis	Avila *et al.*, 2010	Avila *et al.*, 2010	----
Rhinella proboscidea	Jimenez de la Espada, 1875	Jimenez de la Espada, 1875	Jimenez de la Espada, 1875
Rhinella pygmaea	Narvaes & Rodrigues, 2009	Narvaes & Rodrigues, 2009	Mendelson, 1997c

Rhinella quechua	Gallardo, 1961; Harvey & Smith, 1993	----	----
Rhinella roqueana	Melin, 1941	----	----
Rhinella rubescens	Cei, 1980	Pramuk, 2006	Pramuk, 2006
Rhinella rubropunctata	Vellard, 1959; Gallardo, 1962a, Cei, 1980	Pramuk, 2006	Pramuk, 2006
Rhinella ruizi	Grant, 2000	Grant, 2000	Grant, 2000
Rhinella rumbolli	Carrizo, 1992	Carrizo, 1992	----
Rhinella scitula	Caramaschi & Niemeyer, 2003; Avila *et al.*, 2010	----	----
Rhinella sclerocephala	Mijares-Urrutia & Arends, 2001	Mijares-Urrutia & Arends, 2001	----
Rhinella sebbeni	Vaz Silva *et al.*, 2015	Vaz Silva *et al.*, 2015	----
Rhinella stanlaii	Lötters & Köhler, 2000	Lötters & Köhler, 2000	Lötters & Köhler, 2000
Rhinella sternosignata	Boulenger, 1882; Cochran & Goin, 1970; La Marca & Mijares-Urrutia, 1996	La Marca & Mijares-Urrutia, 1996; Vélez-Rodríguez, 2005	Vélez-Rodríguez, 2005
Rhinella tacana	Padial *et al.*, 2006	Padial *et al.*, 2006	
Rhinella tenrec	Lynch & Renjifo, 1990	Lynch & Renjifo, 1990	Lynch & Renjifo, 1990
Rhinella truebae	Lynch & Renjifo, 1990	----	----
Rhinella veredas	Brandao *et al.*, 2007	Brandao *et al.*, 2007	----
Rhinella yanachaga	Lehr *et al.*, 2007	Lehr *et al.*, 2007	Lehr *et al.*, 2007
Rhinella yunga	Moravec *et al.*, 2014	----	----
Sclerophrys arabica	Stock *et al.*, 2001	Stock *et al.*, 2001	----
Sclerophrys asmarae	Tandy *et al.*, 1982	Tandy *et al.*, 1982	----
Sclerophrys blanfordii	----	Boulenger, 1882	----
Sclerophrys buchneri	Peters, 1882	Peters, 1882	----
Sclerophrys capensis	MOP pers. obs.; Poynton, 1964; du Preez & Carruthers, 2009	MOP pers. obs.; Poynton, 1964; du Preez & Carruthers, 2009	----
Sclerophrys channingi	Barej *et al.*, 2011	Barej *et al.*, 2011	----
Sclerophrys chudeaui	Chabanaud, 1919	Chabanaud, 1919	----
Sclerophrys cristiglans	Inger & Menzies, 1961	Inger & Menzies, 1961	----

Sclerophrys danielae	Perret, 1977	Perret, 1977	----
Sclerophrys dodsoni	Boulenger, 1895	Boulenger, 1895	----
Sclerophrys fuliginata	Poynton & Broadley, 1988	Poynton & Broadley, 1988	----
Sclerophrys funerea	----	----	Mendelson, 1997c
Sclerophrys kassasii	Baha El Din, 1993	Baha El Din, 1993	----
Sclerophrys kerinyagae	Keith, 1968	Keith, 1968	----
Sclerophrys pentoni	----	Anderson, 1893	----
Sclerophrys perreti	Onadeko *et al.*, 2014	Onadeko *et al.*, 2014	----
Sclerophrys reesi	Poynton, 1977; Harper *et al.*, 2010	Poynton, 1977	----
Sclerophrys superciliaris	Perret & Amiet, 1971; Barej *et al.*, 2011	Perret & Amiet, 1971; Barej *et al.*, 2011	----
Sclerophrys taiensis	Rödel & Ernst, 2000	Rödel & Ernst, 2000	----
Sclerophrys urunguensis	Loveridge, 1932; Poynton & Broadley, 1988; Poynton *et al.*, 2005	Poynton & Broadley, 1988	----
Sclerophrys vittata	Loveridge, 1932	Loveridge, 1932	----
Truebella skoptes	Graybeal & Cannatella, 1995	Graybeal & Cannatella, 1995	Graybeal & Cannatella, 1995; Pramuk, 2006
Truebella tothastes	Graybeal & Cannatella, 1995	Graybeal & Cannatella, 1995	Graybeal & Cannatella, 1995; Pramuk, 2006
Werneria iboundji	Rödel *et al.*, 2004	----	----
Werneria preussi	Perret & Amiet, 1971; Tandy & Keith, 1972; Rödel *et al.*, 2004	Perret & Amiet, 1971; Tandy & Keith, 1972	Grandison, 1978; Perret & Amiet., 1971
Werneria submontana	Rödel *et al.*, 2004	----	----
Wolterstorffina mirei	Perret, 1972; Boistel & Amiet, 2001	Perret, 1972	Perret, 1972; Grandison, 1978, 1981
Xanthophryne tigerina	Biju *et al.*, 2009	Biju *et al.*, 2009	----

Anura included in Pyron (2014)				
**Family**	**Species**	**Tympanic membrane**	**Tympanic annulus**	**Columella**
Allophrynidae	*Allophryne ruthveni*	Kok & Kalamandeen, 2008	Kok & Kalamandeen, 2008	Fabrezi & Langone, 2000
Alsodidae	*Alsodes barrioi*	Veloso *et al.*, 1981	Veloso *et al.*, 1981	Veloso *et al.*, 1981
	Alsodes coppingeri	Grandison, 1961; Formas *et al.*, 2008	Grandison, 1961; Formas *et al.*, 2008	Grandison, 1961; Formas *et al.*, 2008
	Alsodes gargola	MOP pers. obs.	MOP pers. obs.	MOP pers. obs.
	Alsodes nodosus	Grandison, 1961; Penna *et al.*, 1983	Grandison, 1961; Penna *et al.*, 1983	Grandison, 1961; Penna *et al.*, 1983
	Alsodes pehuenche	----	----	Lynch, 1978
	Alsodes tumultuosus	Penna *et al.*, 1983	Penna *et al.*, 1983	Penna *et al.*, 1983
	Alsodes valdiviensis	Formas *et al.*, 2002	Formas *et al.*, 2002	Formas *et al.*, 2002
	Alsodes vanzolinii	----	----	Lynch, 1978
	Eupsophus calcaratus	----	----	Lynch, 1971
	Eupsophus contulmoensis	Ortiz *et al.*, 1989	----	----
	Eupsophus emiliopugini	----	Formas, 1989	----
	Eupsophus roseus	Grandison, 1961	Grandison, 1961	Grandison, 1961; Lynch, 1971; BLB pers. obs.
	Eupsophus septentrionalis	Ibarra-Vidal *et al.*, 2004	-----	Ibarra-Vidal *et al.*, 2004
	Eupsophus vertebralis	Grandison, 1961	Grandison, 1961	Grandison, 1961
	Limnomedusa macroglossa	DB pers. obs.	DB pers. obs.; Boulenger, 1882	Lynch, 1971
Alytidae	*Alytes cisternasii*	----	Boulenger, 1882	----
	Alytes obstetricans	Wever, 1985	Wever, 1985; Boulenger, 1882	Wever, 1985
	Discoglossus montalentii	----	Lanza *et al.*, 1984	----
	Discoglossus pictus	Beukema *et al.*, 2013	Smirnov, 1991; Beukema *et al.*, 2013	Smirnov, 1991
	Discoglossus sardus	----	Lanza *et al.*, 1984	Maglia, 1998
	Discoglossus scovazzi	----	Lanza *et al.*, 1984	----

Aromobatidae	*Allobates brunneus*	Lima *et al.*, 2009	Lima *et al.*, 2009	TG pers. obs.
	Allobates femoralis	----	TG pers. obs.	TG pers. obs.
	Allobates granti	----	Kok *et al.*, 2006	----
	Allobates insperatus	----	TG pers. obs.	TG pers. obs.
	Allobates juanii	----	TG pers. obs.	TG pers. obs.
	Allobates kingsburyi	----	TG pers. obs.	TG pers. obs.
	Allobates talamancae	Savage, 2002	Savage, 2002; TG pers. obs.	TG pers. obs.
	Allobates undulatus	----	----	TG pers. obs.
	Anomaloglossus baebatrachus	Boistel & de Massary, 1999	Boistel & de Massary, 1999	----
	Anomaloglossus beebei	----	TG pers. obs.	----
	Anomaloglossus kaiei	Kok & Kalamandeen, 2008	Kok & Kalamandeen, 2008	----
	Aromobates nocturnus	----	TG pers. obs.	Myers *et al.*, 1991; TG pers. obs.
	Mannophryne collaris	----	TG pers. obs.	TG pers. obs.
	Mannophryne herminae	Nussbaum & Wu, 2007	Nussbaum & Wu, 2007; TG pers. obs.	Nussbaum & Wu, 2007; TG pers. obs.
	Mannophryne trinitatis	----	TG pers. obs.	TG pers. obs.
	Rheobates palmatus	----	TG pers. obs.	----
Arthroleptidae	*Arthroleptis adolfifriederici*	----	-----	Laurent, 1940
	Arthroleptis affinis	Spawls *et al.*, 2006	Spawls *et al.*, 2006	----
	Arthroleptis poecilonotus	----	Boulenger, 1882	Laurent, 1940
	Arthroleptis reichi	----	-----	Laurent, 1940
	Arthroleptis schubotzi	----	-----	Laurent, 1940
	Arthroleptis stenodactylus	Scott, 2005; Spawls *et al.*, 2006; Nussbaum & Wu, 2007; du Preez & Carruthers, 2009	Scott, 2005; Nussbaum & Wu, 2007; du Preez & Carruthers, 2009	Laurent, 1940; Scott, 2005; Nussbaum & Wu, 2007
	Arthroleptis taeniatus	Scott, 2005	Scott, 2005	Scott, 2005
	Arthroleptis tanneri	Spawls *et al.*, 2006	Spawls *et al.*, 2006	----
	Arthroleptis variabilis	Scott, 2005	Scott, 2005	Laurent 1940; Scott, 2005

Aromobatidae (cont.)	*Arthroleptis wahlbergii*	----	Boulenger, 1882	----
	Arthroleptis xenodactyloides	du Preez & Carruthers, 2009	du Preez & Carruthers, 2009	Laurent, 1940
	Astylosternus batesii	Boulenger, 1900c	Boulenger, 1900c	----
	Astylosternus diadematus	Scott, 2005	Scott, 2005	Scott, 2005
	Cardioglossa gracilis	Scott, 2005	Scott, 2005	Laurent 1940; Scott, 2005
	Cardioglossa leucomystax	Boulenger, 1903a	Boulenger, 1903a	----
	Cardioglossa manengouba	Blackburn, 2008	Blackburn, 2008	----
	Cardioglossa oreas	Blackburn, 2008	Blackburn, 2008	----
	Leptodactylodon bicolor	Amiet, 1980	----	----
	Leptopelis argenteus	----	Spawls *et al.*, 2006	----
	Leptopelis bocagii	du Preez & Carruthers, 2009	Drewes, 1984; du Preez & Carruthers, 2009	Laurent, 1941b
	Leptopelis calcaratus	----	Laurent, 1941b	Laurent, 1941b
	Leptopelis kivuensis	Spawls *et al.*, 2006	Spawls *et al.*, 2006	----
	Leptopelis millsoni	----	Laurent, 1941b	Laurent, 1941b
	Leptopelis vermiculatus	Scott, 2005	Scott, 2005	Scott, 2005
	Nyctibates corrugatus	Scott, 2005	Scott, 2005	Scott, 2005
	Scotobleps gabonicus	Scott, 2005	Scott, 2005	Scott, 2005
	Trichobatrachus robustus	Scott, 2005	Scott, 2005	Scott, 2005
Ascaphidae	*Ascaphus truei*	Wever, 1985	Wever, 1985	Wever, 1985
Batrachylidae	*Atelognathus patagonicus*	----	----	Lynch, 1971; BLB pers. obs.
	Atelognathus salai	----	----	Meriggio *et al.*, 2004
	Batrachyla leptopus	BLB pers. obs.	BLB pers. obs.	Lynch, 1971, 1978; BLB pers. obs.
	Batrachyla taeniata	----	----	Lynch, 1971
	Hylorina sylvatica	BLB pers. obs.	Boulenger, 1882; BLB pers. obs.	Lynch, 1971; BLB pers. obs.

Bombinatoridae	*Barbourula busuangensis*	Lynch, 1973; Laurent, 1986	----	Lynch, 1973; Laurent, 1986
	Bombina bombina	Wever, 1985	Wever, 1985	Parker, 1881; Stadtmüller, 1931; Wever, 1985; Smirnov, 1991
	Bombina maxima	----	----	Stadtmüller, 1931
	Bombina orientalis	Wever, 1985	Wever, 1985	Hetherington & Lindquist, 1999; Smirnov, 1991; Wever, 1985; Maglia, 1998
	Bombina pachypus	----	----	Stadtmüller, 1931
	Bombina variegata	Wever, 1985	Wever, 1985	Wever, 1985
Brachycephalidae	*Brachycephalus alipioi*	Pombal & Gasparini, 2006	Pombal & Gasparini, 2006	----
	Brachycephalus brunneus	Nussbaum & Wu, 2007; da Silva *et al*., 2007	Nussbaum & Wu, 2007; da Silva *et al*., 2007	Nussbaum & Wu, 2007; da Silva *et al*., 2007
	Brachycephalus didactylus	da Silva *et al*., 2007	da Silva *et al*., 2007	da Silva *et al*., 2007
	Brachycephalus ephippium	da Silva *et al*., 2007; Haddad *et al*., 2010	da Silva *et al*., 2007; Haddad *et al*., 2010	da Silva *et al*., 2007; Haddad *et al*., 2010
	Brachycephalus ferruginus	Alves *et al*., 2006	Alves *et al*., 2006	Alves *et al*., 2006
	Brachycephalus hermogenesi	Haddad *et al*., 2010; Giaretta & Sawaya, 1998	Haddad *et al*., 2010; Giaretta & Sawaya, 1998	Haddad *et al*., 2010; Giaretta & Sawaya, 1998; da Silva *et al*., 2007
	Brachycephalus izeckshoni	Haddad *et al*., 2010; Ribeiro *et al*., 2005	Haddad *et al*., 2010; Ribeiro *et al*., 2005	Haddad *et al*., 2010; Ribeiro *et al*., 2005
	Brachycephalus nodoterga	da Silva *et al*., 2007; Haddad *et al*., 2010	da Silva *et al*., 2007	da Silva *et al*., 2007
	Brachycephalus pernix	da Silva *et al*., 2007; Haddad *et al*., 2010	Haddad *et al*., 2010; da Silva *et al*., 2007	Haddad *et al*., 2010; da Silva *et al*., 2007
	Brachycephalus pitanga	Alves *et al*., 2009	Alves *et al*., 2009	----
	Brachycephalus pombali	Alves *et al*., 2006	Alves *et al*., 2006	Alves *et al*., 2006
	Brachycephalus vertebralis	da Silva *et al*., 2007; Pombal, 2001	da Silva *et al*., 2007	da Silva *et al*., 2007
	Ischnocnema bolbodactyla	Cochran, 1955	Cochran, 1955	----
	Ischnocnema erythromera	Heyer, 1984	Heyer, 1984	----

Brachycephalidae (cont.)	*Ischnocnema guentheri*	Heyer *et al.*, 1990	Heyer *et al.*, 1990; Lynch, 1971	Lynch, 1971
	Ischnocnema hoehnei	Heyer *et al.*, 1990	Heyer *et al.*, 1990	----
	Ischnocnema holti	Targino & Carvalho-e-Silva, 2008	Targino & Carvalho-e-Silva, 2008	----
	Ischnocnema izecksohni	Caramaschi & Kisteumacher, 1989	Caramaschi & Kisteumacher, 1989	----
	Ischnocnema juipoca	Haddad *et al.*, 2013	Haddad *et al.*, 2013	----
	Ischnocnema lactea	Miranda-Ribeiro, 1926	Miranda-Ribeiro, 1926	----
	Ischnocnema nasuta	Haddad *et al.*, 2013	Haddad *et al.*, 2013	Lynch, 1971
	Ischnocnema octavioi	Vrcibradic *et al.*, 2008	Vrcibradic *et al.*, 2008	Lynch, 1971
	Ischnocnema oea	Heyer, 1984	Heyer, 1984	----
	Ischnocnema parva	Nussbaum & Wu, 2007; Heyer *et al.*, 1990	Nussbaum & Wu, 2007; Heyer *et al.*, 1990	Lynch, 1971; Nussbaum & Wu, 2007
	Ischnocnema sambaqui	Castanho & Haddad, 2000	Castanho & Haddad, 2000	----
	Ischnocnema spanios	Heyer, 1985	----	----
	Ischnocnema venancioi	Lutz, 1958	Lutz, 1958	Lynch, 1971
	Ischnocnema verrucosa	Lynch, 1972	Lynch, 1972	----
Brevicipitidae	*Balebreviceps hillmani*	Largen & Drewes, 1989	Largen & Drewes, 1989	Largen & Drewes, 1989
	Breviceps fuscus	du Preez & Carruthers, 2009	----	----
	Breviceps mossambicus	Scott, 2005	Parker, 1934; Scott, 2005	Parker, 1934; Scott, 2005
	Callulina kreffti	----	Parker, 1934	Parker, 1934
	Probreviceps loveridgei	Harper *et al.*, 2010	Harper *et al.*, 2010; Parker, 1934	Parker, 1934
	Probreviceps macrodactylus	Harper *et al.*, 2010	Harper *et al.*, 2010; Parker, 1934	Parker, 1934
	Probreviceps rungwensis	Harper *et al.*, 2010	Harper *et al.*, 2010; Parker, 1934	Parker, 1934
	Probreviceps uluguruensis	Harper *et al.*, 2010	Harper *et al.*, 2010; Parker, 1934	Parker, 1934
	Spelaeophryne methneri	----	Harper *et al.*, 2010; Parker, 1934	Parker, 1934

Calyptocephalellidae	*Calyptocephalella gayi*	----	Parker, 1881; Boulenger, 1882	Lynch, 1971, 1978; Parker, 1881
	Telmatobufo bullocki	Formas *et al.*, 2001	Formas *et al.*, 2001	Lynch, 1971; Formas *et al.*, 2001
	Telmatobufo venustus	Formas *et al.*, 2001	Formas *et al.*, 2001	Lynch, 1978; Formas *et al.*, 2001
Centrolenidae	*Celsiella revocata*	----	Guayasamin *et al.*, 2009	----
	Celsiella vozmedianoi	----	Ayarzagüena & Señaris, 1997; Guayasamin *et al.*, 2009	----
	Centrolene altitudinale	Guayasamin *et al.*, 2006	Guayasamin *et al.*, 2006	----
	Centrolene bacatum	Guayasamin *et al.*, 2006	Guayasamin *et al.*, 2006	----
	Centrolene ballux	----	Duellman & Burrowes, 1989	----
	Centrolene buckleyi	Guayasamin *et al.*, 2006	Boulenger, 1882; Lynch & Renjifo, 2001; Guayasamin *et al.*, 2006	----
	Centrolene condor	Cisneros-Heredia & Morales-Mite, 2008	Cisneros-Heredia & Morales-Mite, 2008	----
	Centrolene daidaleum	Guayasamin *et al.*, 2009	Guayasamin *et al.*, 2009	----
	Centrolene geckoideum	Rueda-Almonacid, 1994; Lynch & Renjifo, 2001	Boulenger, 1882; Rueda-Almonacid, 1994; Lynch & Renjifo, 2001	Rueda-Almonacid, 1994
	Centrolene heloderma	----	Duellman, 1981	----
	Centrolene peristictum	Lynch & Duellman, 1973	Lynch & Duellman, 1973	----
	Centrolene pipilatum	Lynch & Duellman, 1973	Lynch & Duellman, 1973	----
	Centrolene venezuelense	----	Rivero, 1968b	----
	Cochranella euknemos	Wever, 1985	Wever, 1985	Wever, 1985
	Cochranella litoralis	----	Ruiz-Carranza & Lynch, 1996	----
	Cochranella nola	----	Guayasamin *et al.*, 2009	----
	Espadarana andina	Ruiz-Carranza & Lynch, 1995	Ruiz-Carranza & Lynch, 1995; Lynch & Renjifo, 2001	----
	Espadarana callistomma	Guayasamin & Trueb, 2007	Guayasamin & Trueb, 2007	Guayasamin & Trueb, 2007

Centrolenidae (cont.)	*Espadarana prosoblepon*	MOP pers. obs.; Ruiz-Carranza & Lynch, 1995	MOP pers. obs.; Ruiz-Carranza & Lynch, 1995	----
	Hyalinobatrachium aureoguttatum	Barrera-Rodríguez, 1999	Barrera-Rodríguez, 1999; Cisneros & McDiarmid, 2007	Barrera-Rodríguez, 1999
	Hyalinobatrachium bergeri	Cannatella, 1980	Cannatella, 1980	----
	Hyalinobatrachium colymbiphyllum	Savage, 2002	Barrera-Rodríguez, 1999; Savage, 2002	Barrera-Rodríguez, 1999
	Hyalinobatrachium eccentricum	----	Cisneros & McDiarmid, 2007	----
	Hyalinobatrachium fleischmanni	Wever, 1985 present, Savage, 2002 absent	Wever, 1985	Wever, 1985
	Hyalinobatrachium iaspidiense	Castroviejo-Fisher *et al.*, 2011	Castroviejo-Fisher *et al.*, 2011	----
	Hyalinobatrachium ibama	----	Ruiz-Carranza & Lynch, 1998	----
	Hyalinobatrachium ignioculus	----	Guayasamin *et al.*, 2009	----
	Hyalinobatrachium mondolfii	Castroviejo-Fisher *et al.*, 2011	Castroviejo-Fisher *et al.*, 2011	----
	Hyalinobatrachium munozorum	Lynch & Duellman, 1973	Lynch & Duellman, 1973	----
	Hyalinobatrachium orocostale	Rivero, 1968b; Castroviejo-Fisher *et al.*, 2011	Castroviejo-Fisher *et al.*, 2011	----
	Hyalinobatrachium talamancae	Savage, 2002	Savage, 2002	----
	Hyalinobatrachium taylori	Castroviejo-Fisher *et al.*, 2011	Castroviejo-Fisher *et al.*, 2011	----
	Ikakogi tayrona	Ruiz-Carranza & Lynch, 1991	Ruiz-Carranza & Lynch, 1991; Cisneros & McDiarmid, 2007	Ruiz-Carranza & Lynch, 1991
	Nymphargus bejaranoi	Cannatella, 1980	Cannatella, 1980	----
	Nymphargus cochranae	Lynch & Duellman, 1973	Lynch & Duellman, 1973	----
	Nymphargus grandisonae	----	Lynch & Duellman, 1973; Cisneros & McDiarmid, 2007	----
	Nymphargus griffithsi	----	Lynch & Duellman, 1973	----

Centrolenidae (cont.)	*Nymphargus megacheirus*	----	Lynch & Duellman, 1973	----
	Nymphargus posadae	Guayasamin *et al.*, 2006	Guayasamin *et al.*, 2006	----
	Nymphargus siren	Lynch & Duellman, 1973	Lynch & Duellman, 1973; Cisneros & McDiarmid, 2007	----
	Nymphargus wileyi	Guayasamin *et al.*, 2006	Guayasamin *et al.*, 2006	----
	Rulyrana adiazeta	----	Guayasamin *et al.*, 2009	----
	Rulyrana flavopunctata	----	Lynch & Duellman, 1973	----
	Sachatamia albomaculata	Savage, 2002	Savage, 2002	----
	Sachatamia ilex	Savage, 2002	Savage, 2002	----
	Teratohyla midas	Lynch & Duellman, 1973	Lynch & Duellman, 1973; Beirne & Witworth, 2011	----
	Teratohyla pulverata	Savage, 2002	Boulenger, 1882; Savage, 2002	----
	Vitreorana antisthenesi	Rivero, 1968b	Rivero, 1968b	----
	Vitreorana gorzulae	Kok & Kalamandeen, 2008	Kok & Kalamandeen, 2008	----
	Vitreorana ritae	Lima *et al.*, 2006	Lima *et al.*, 2006	----
	Vitreorana uranoscopa	DB pers. obs.; Wever, 1985	DB pers. obs.; Wever, 1985	Wever, 1985
Ceratobatrachidae	*Alcalus baluensis*	----	Inger, 1966	----
	Cornufer guentheri	----	Fuiten, 2012	Laurent, 1943b, Fuiten, 2012
	Cornufer guppyi	----	Fuiten, 2012	Fuiten, 2012
	Cornufer papuensis	Menzies, 2006	Menzies, 2006	----
	Cornufer vertebralis	Scott, 2005	Scott, 2005 Fuiten, 2012	Scott, 2005 Fuiten, 2012
	Platymantis corrugatus	Scott, 2005	Scott, 2005	Scott, 2005
	Platymantis dorsalis	----	Fuiten, 2012	Fuiten, 2012
	Platymantis hazelae	----	Fuiten, 2012 Inger, 1954	Fuiten, 2012
Ceratophryidae	*Ceratophrys cornuta*	Lima *et al.*, 2006	Boulenger, 1882; Lima *et al.*, 2006	----
	Ceratophrys ornata	DB pers. obs.	DB pers. obs.	Lynch, 1971
	Chacophrys pierottii	DB pers. obs.	DB pers. obs.	Wild, 1999
	Lepidobatrachus laevis	MOP pers. obs.	MOP pers. obs.	MOP pers. obs.

Conrauidae	*Conraua crassipes*	Boulenger, 1882; Scott, 2005	Scott, 2005	Scott, 2005
	Conraua goliath	----	Scott, 2005	Scott, 2005
Craugastoridae	*Barycholos pulcher*	Heyer, 1969	Heyer, 1969	Heyer, 1969; Lynch, 1971
	Bryophryne cophites	Lynch, 1975b	Lynch, 1975b	Lynch, 1975b
	Ceuthomantis smaragdinus	Heinicke *et al.*, 2009	Heinicke *et al.*, 2009	Heinicke *et al.*, 2009
	Craugastor alfredi	Boulenger, 1898	Boulenger, 1898	----
	Craugastor andi	Savage, 2002	Savage, 2002	----
	Craugastor angelicus	Savage, 1975	Savage, 1975	----
	Craugastor augusti	Zweifel, 1956b	Zweifel, 1956b	Lynch, 1971
	Craugastor bocourti	Hedges *et al.*, 2008	Hedges *et al.*, 2008	----
	Craugastor bransfordii	Savage, 2002	Savage, 2002	----
	Craugastor crassidigitus	Lynch & Myers, 1983	Lynch & Myers, 1983	----
	Craugastor cuaquero	Savage, 2002	Savage, 2002	----
	Craugastor daryi	Ford & Savage, 1984	Ford & Savage, 1984	----
	Craugastor emcelae	Lynch, 1985	Lynch, 1985	----
	Craugastor fitzingeri	Lynch & Myers, 1983	Lynch & Myers, 1983	Lynch, 1971
	Craugastor fleischmanni	Campbell & Savage, 2000	Campbell & Savage, 2000	----
	Craugastor laticeps	Savage, 1987	Savage, 1987	----
	Craugastor lineatus	Savage, 1987	Savage, 1987	----
	Craugastor loki	Lynch, 2000	Lynch, 2000	----
	Craugastor longirostris	Wever, 1985; Lynch & Myers, 1983	Wever, 1985; Lynch & Myers, 1983	Wever, 1985
	Craugastor megacephalus	Savage & Myers, 2002	Savage & Myers, 2002	----
	Craugastor melanostictus	Savage & DeWeese, 1981	Savage & DeWeese, 1981	----
	Craugastor mexicanus	Lynch, 2000	Lynch, 2000	----
	Craugastor montanus	Taylor, 1942	Taylor, 1942	----
	Craugastor obesus	Campbell & Savage, 2000	Campbell & Savage, 2000	----
	Craugastor podiciferus	Savage, 2002	Savage, 2002	Lynch, 1971
	Craugastor punctariolus	Campbell & Savage, 2000	Campbell & Savage, 2000	----

Craugastoridae (cont.)	*Craugastor pygmaeus*	Taylor, 1937 "1936"	Taylor, 1937 "1936"	----
	Craugastor raniformis	Lynch & Myers, 1983	Lynch & Myers, 1983	----
	Craugastor ranoides	Campbell & Savage, 2000	Campbell & Savage, 2000	----
	Craugastor rhodopis	Lynch, 2000	Lynch, 2000	Lynch, 1971
	Craugastor rugulosus	Campbell & Savage, 2000	Campbell & Savage, 2000	----
	Craugastor rupinius	Campbell & Savage, 2000	Campbell & Savage, 2000	----
	Craugastor sandersoni	Campbell & Savage, 2000	Campbell & Savage, 2000	----
	Craugastor spatulatus	Smith, 1939	Smith, 1939	----
	Craugastor stuarti	Lynch, 1965b	Lynch, 1965b	----
	Craugastor tabasarae	Savage *et al.*, 2004	Savage *et al.*, 2004	----
	Craugastor talamancae	Savage, 2002	Savage, 2002	----
	Craugastor tarahumaraensis	Taylor, 1940a	Taylor, 1940a	----
	Craugastor uno	Streicher *et al.*, 2011	Streicher *et al.*, 2011	----
	Euparkerella brasiliensis	Izecksohn & de Carvalho-e-Silva, 2001	Izecksohn & de Carvalho-e-Silva, 2001	Lynch, 1971
	Haddadus binotatus	MOP pers. obs.	MOP pers. obs.	Lynch, 1971
	Holoaden bradei	Hedges *et al.*, 2008; Lutz, 1958	Hedges *et al.*, 2008;	Lynch, 1971
	Holoaden luederwaldti	Hedges *et al.*, 2008; Miranda-Ribeiro, 1920	Hedges *et al.*, 2008	----
	Hypodactylus brunneus	Lynch, 1975b	Lynch, 1975b	----
	Hypodactylus dolops	Lynch & Duellman, 1980	Lynch & Duellman, 1980	Lynch, 1989
	Hypodactylus elassodiscus	Lynch, 1973	Lynch, 1973	----
	Hypodactylus peraccai	Lynch, 1975b	Lynch, 1975b	----
	Lynchius flavomaculatus	Parker, 1938	Lynch, 1975b	Lynch, 1971
	Lynchius nebulanastes	Cannatella, 1984	Cannatella, 1984	Cannatella, 1984
	Lynchius parkeri	Lynch, 1975b	Lynch, 1975b	----
	Lynchius simmonsi	Lynch, 1974b	Lynch, 1974b	----
	Noblella heyeri	Lynch, 1986	Lynch, 1986	Lynch, 1986
	Noblella lochites	Lynch, 1976b	Lynch, 1976b	Lynch, 1976b

Craugastoridae (cont.)	*Noblella myrmecoides*	Lynch, 1976b	Lynch, 1976b	Lynch, 1976b
	Noblella peruviana	Bokermann, 1975; Noble, 1921	Bokermann, 1975; Noble, 1921	Lynch, 1971
	Oreobates barituensis	Pereyra *et al.*, 2014; Vaira & Ferrari, 2008	Pereyra *et al.*, 2014; Vaira & Ferrari, 2008	----
	Oreobates choristolemma	Harvey & Sheehy, 2005	Harvey & Sheehy, 2005	----
	Oreobates cruralis	Boulenger, 1902	Boulenger, 1902	----
	Oreobates discoidalis	Lynch, 1989; Pereyra *et al.*, 2014	Lynch, 1989; Pereyra *et al.*, 2014	Lynch, 1989;
	Oreobates granulosus	Duellman & Lehr, 2009	Duellman & Lehr, 2009	----
	Oreobates heterodactylus	Padial & de la Riva, 2005	Padial & de la Riva, 2005	----
	Oreobates ibischi	Reichle *et al.*, 2001	Reichle *et al.*, 2001	----
	Oreobates lehri	Padial *et al.*, 2007	Padial *et al.*, 2007	----
	Oreobates madidi	Padial *et al.*, 2005a	Padial *et al.*, 2005a	----
	Oreobates pereger	Duellman & Lehr, 2009	Duellman & Lehr, 2009	----
	Oreobates quixensis	Duellman & Lehr, 2009	Duellman & Lehr, 2009	Lynch, 1971
	Oreobates sanctaecrucis	Harvey & Keck, 1995	Harvey & Keck, 1995	----
	Oreobates sanderi	Padial *et al.*, 2005b	Padial *et al.*, 2005b	----
	Oreobates saxatilis	Duellman, 1990	Duellman, 1990	----
	Phrynopus barthlenae	Duellman & Lehr, 2009	Duellman & Lehr, 2009	----
	Phrynopus bracki	Duellman & Lehr, 2009	Duellman & Lehr, 2009	----
	Phrynopus bufoides	Duellman & Lehr, 2009	Duellman & Lehr, 2009	----
	Phrynopus horstpauli	Duellman & Lehr, 2009	Duellman & Lehr, 2009	----
	Phrynopus juninensis	Duellman & Lehr, 2009	Duellman & Lehr, 2009	Lynch, 1971
	Phrynopus kauneorum	Duellman & Lehr, 2009	Duellman & Lehr, 2009	----
	Phrynopus pesantesi	Duellman & Lehr, 2009	Duellman & Lehr, 2009	----
	Phrynopus tautzorum	Duellman & Lehr, 2009	Duellman & Lehr, 2009	----
	Pristimantis acatallelus	Lynch & Ruiz-Carranza, 1983	Lynch & Ruiz-Carranza, 1983	----
	Pristimantis acerus	Lynch & Duellman, 1980	Lynch & Duellman, 1980	----

Craugastoridae (cont.)	*Pristimantis achatinus*	Lynch & Myers, 1983	Lynch & Myers, 1983	Lynch, 1971
	Pristimantis actites	Lynch, 1979c	Lynch, 1979c	----
	Pristimantis acuminatus	Duellman & Lehr, 2009	Duellman & Lehr, 2009	----
	Pristimantis altae	Savage, 2002	Savage, 2002	----
	Pristimantis altamazonicus	Duellman & Lehr, 2009	Duellman & Lehr, 2009	Lynch, 1971
	Pristimantis angustilineatus	Lynch, 1998	Lynch, 1998	----
	Pristimantis aniptopalmatus	----	Duellman & Hedges, 2005	----
	Pristimantis appendiculatus	Lynch & Duellman, 1997	Lynch & Duellman, 1997	----
	Pristimantis ardalonychus	Duellman & Pramuk, 1999	Duellman & Pramuk, 1999	----
	Pristimantis bipunctatus	----	Duellman & Hedges, 2005	----
	Pristimantis bogotensis	Avilan & Hoyos, 2006	Avilan & Hoyos, 2006	Lynch, 1971; Avilan & Hoyos, 2006
	Pristimantis brevifrons	Lynch, 1981b	Lynch, 1981b	----
	Pristimantis bromeliaceus	Lynch, 1979b	Lynch, 1979b	----
	Pristimantis buccinator	Duellman & Lehr, 2009	Duellman & Lehr, 2009	----
	Pristimantis buckleyi	Lynch, 1981a	Lynch, 1981a	----
	Pristimantis cajamarcensis	Duellman & Lehr, 2009	Duellman & Lehr, 2009	----
	Pristimantis calcaratus	Lynch, 1996	Ospina-Sarria *et al*., 2011	----
	Pristimantis calcarulatus	Lynch & Duellman, 1997	Lynch & Duellman, 1997	----
	Pristimantis caprifer	Lynch & Duellman, 1997	Lynch & Duellman, 1997	----
	Pristimantis caryophyllaceus	Savage, 2002	Savage, 2002	Lynch, 1971
	Pristimantis celator	Lynch, 1976a	Lynch, 1976a	----
	Pristimantis cerasinus	Savage, 1981	----	Lynch, 1971
	Pristimantis ceuthospilus	Duellman & Wild, 1993	Duellman & Wild, 1993	----
	Pristimantis chalceus	Lynch & Duellman, 1997	Lynch & Duellman, 1997	Lynch, 1971
	Pristimantis chiastonotus	Lynch & Hoogmoed, 1977	Lynch & Hoogmoed, 1977	----
	Pristimantis chloronotus	Lynch, 1969	Lynch, 1969	Lynch, 1971
	Pristimantis citriogaster	Duellman & Lehr, 2009	Duellman & Lehr, 2009	----
	Pristimantis colomai	Lynch & Duellman, 1997	Lynch & Duellman, 1997	----

Craugastoridae (cont.)	*Pristimantis condor*	Duellman & Lehr, 2009	Duellman & Lehr, 2009	----
	Pristimantis conspicillatus	Duellman & Lehr, 2009	Duellman & Lehr, 2009	Lynch, 1971
	Pristimantis cremnobates	Lynch & Duellman, 1980	Lynch & Duellman, 1980	----
	Pristimantis crenunguis	Lynch, 1976a	Lynch, 1976a	----
	Pristimantis croceoinguinis	Lynch, 1968	Lynch, 1968	Lynch, 1971
	Pristimantis crucifer	Lynch, 1976a	Lynch, 1976a	----
	Pristimantis cruentus	Savage, 1981	Savage, 1981	Lynch, 1971
	Pristimantis cryophilius	Lynch, 1979b	Lynch, 1979b	----
	Pristimantis curtipes	Lynch, 1981a	Lynch, 1981a	Lynch, 1971
	Pristimantis danae	Duellman & Lehr, 2009	Duellman & Lehr, 2009	----
	Pristimantis devillei	Lynch, 1969	Lynch, 1969	Lynch, 1971
	Pristimantis diadematus	Duellman & Lehr, 2009	Duellman & Lehr, 2009	----
	Pristimantis dissimulatus	Lynch & Duellman, 1997	Lynch & Duellman, 1997	----
	Pristimantis duellmani	Lynch, 1980c	Lynch, 1980c	MT pers. obs.
	Pristimantis eriphus	Lynch & Duellman, 1980	Lynch & Duellman, 1980	----
	Pristimantis erythropleura	Lynch, 1994	Lynch, 1994	----
	Pristimantis euphronides	Kaiser *et al.*, 1994b	Kaiser *et al.*, 1994b	----
	Pristimantis fenestratus	Duellman & Lehr, 2009	Duellman & Lehr, 2009	----
	Pristimantis frater	Pyburn & Lynch, 1981	Pyburn & Lynch, 1981	Lynch, 1971
	Pristimantis gaigei	Wever, 1985; Lynch, 1980a	Wever, 1985; Lynch, 1980a	Lynch, 1971; Wever, 1985
	Pristimantis galdi	Lynch & Duellman, 1980	Lynch & Duellman, 1980	Lynch, 1971
	Pristimantis gentry	----	MTR pers. obs.	MTR pers. obs.
	Pristimantis glandulosus	Lynch, 1969	Lynch, 1969	----
	Pristimantis gutturalis	Hoogmoed *et al.*, 1977	Hoogmoed *et al.*, 1977	----
	Pristimantis hectus	Lynch & Duellman, 1997	Lynch & Duellman, 1997	----
	Pristimantis imitatrix	Duellman, 1978	Duellman, 1978	----
	Pristimantis inguinalis	----	Parker, 1940	----
	Pristimantis inusitatus	Lynch & Duellman, 1980	Lynch & Duellman, 1980	----

Craugastoridae (cont.)	*Pristimantis juanchoi*	JJOS pers. obs.	Lynch, 1996	----
	Pristimantis jubatus	García-R & Lynch, 2006	García-R & Lynch, 2006	----
	Pristimantis kelephus	Lynch, 1998	Lynch, 1998	----
	Pristimantis koehleri	Padial & de la Riva, 2009	Padial & de la Riva, 2009	----
	Pristimantis labiosus	Lynch & Duellman, 1997	Lynch & Duellman, 1997	----
	Pristimantis lanthanites	Lynch, 1975a	Lynch, 1975a	----
	Pristimantis latidiscus	Lynch & Duellman, 1997	Lynch & Duellman, 1997	----
	Pristimantis leoni	Lynch & Duellman, 1997	Lynch & Duellman, 1997	----
	Pristimantis librarius	----	Flores & Vigle, 1994	----
	Pristimantis lirellus	Duellman & Pramuk, 1999	Duellman & Pramuk, 1999	----
	Pristimantis llojsintuta	Köhler & Lötters, 1999	Köhler & Lötters, 1999	----
	Pristimantis luteolateralis	----	Lynch, 1976a	----
	Pristimantis lymani	Lynch & Duellman, 1997	Lynch & Duellman, 1997	----
	Pristimantis malkini	Lynch, 1980b	Lynch, 1980b	----
	Pristimantis marmoratus	Ouboter & Jairam, 2012	Ouboter & Jairam, 2012	----
	Pristimantis martiae	Lynch, 1974a	Lynch, 1974a	----
	Pristimantis melanogaster	Duellman & Lehr, 2009	Duellman & Lehr, 2009	----
	Pristimantis miyatai	Lynch, 1984	Lynch, 1984	----
	Pristimantis moro	Savage, 2002	----	----
	Pristimantis museosus	Ibañez *et al.*, 1994	Ibañez *et al.*, 1994	----
	Pristimantis myops	Lynch, 1998	Lynch, 1998	----
	Pristimantis nervicus	Lynch, 1994	Lynch, 1994	----
	Pristimantis nyctophylax		Lynch, 1976a	----
	Pristimantis ockendeni	Duellman & Lehr, 2009	Duellman & Lehr, 2009	----
	Pristimantis ocreatus	Lynch, 1981a	Lynch, 1981a	----
	Pristimantis orcesi	Lynch, 1981a	Lynch, 1981a	----
	Pristimantis orestes	Lynch, 1979b	Lynch, 1979b	----
	Pristimantis paisa	Lynch & Ardila-Robayo, 1999	Lynch & Ardila-Robayo, 1999	----

Craugastoridae (cont.)	*Pristimantis palmeri*	JJOS pers. obs.	Lynch, 1996	Lynch, 1971
	Pristimantis pardalis	Savage, 2002	Savage, 2002	----
	Pristimantis parvillus	Lynch, 1976a	Lynch, 1976a	----
	Pristimantis peruvianus	Duellman & Lehr, 2009	Duellman & Lehr, 2009	----
	Pristimantis petrobardus	Duellman & Lehr, 2009	Duellman & Lehr, 2009	----
	Pristimantis phoxocephalus	Lynch, 1979b	Lynch, 1979b	----
	Pristimantis pirrensis	Ibañez & Crawford, 2004	Ibañez & Crawford, 2004	----
	Pristimantis platydactylus	Duellman & Lehr, 2009	Duellman & Lehr, 2009	----
	Pristimantis prolatus	Lynch & Duellman, 1980	Lynch & Duellman, 1980	----
	Pristimantis ptochus	----	Lynch, 1998	----
	Pristimantis pulvinatus	Rivero, 1968a	Rivero, 1968a	----
	Pristimantis pycnodermis	Lynch, 1979b	Lynch, 1979b	----
	Pristimantis pyrrhomerus	Lynch & Duellman, 1997	Lynch & Duellman, 1997	----
	Pristimantis quantus	Lynch, 1998	Lynch, 1998	----
	Pristimantis quaquaversus	Lynch, 1974a	Lynch, 1974a	----
	Pristimantis quinquagesimus	Lynch & Duellman, 1997	Lynch & Duellman, 1997	----
	Pristimantis ramagii	Boulenger, 1888	Boulenger, 1888	----
	Pristimantis reichlei	Padial & de la Riva, 2009	Padial & de la Riva, 2009	----
	Pristimantis rhabdocnemus	Duellman & Hedges, 2005	Duellman & Hedges, 2005	----
	Pristimantis rhabdolaemus	Duellman & Lehr, 2009	Duellman & Lehr, 2009	----
	Pristimantis rhodoplichus	Duellman & Wild, 1993	Duellman & Wild, 1993	----
	Pristimantis ridens	Savage, 2002	Savage, 2002	Lynch, 1971
	Pristimantis riveti	Lynch, 1979b	Lynch, 1979b	----
	Pristimantis rozei	Rivero, 1961	Rivero, 1961	----
	Pristimantis sagittulus	Duellman & Lehr, 2009	Duellman & Lehr, 2009	----
	Pristimantis samaipatae	Köhler & Jungfer, 1995	Köhler & Jungfer, 1995	----
	Pristimantis savagei	Pyburn & Lynch, 1981	Pyburn & Lynch, 1981	----
	Pristimantis schultei	Duellman & Lehr, 2009	Duellman & Lehr, 2009	----

Craugastoridae (cont.)	*Pristimantis shrevei*	Kaiser *et al.*, 1994b	Kaiser *et al.*, 1994b	----
	Pristimantis simonbolivari	Lynch & Duellman, 1997	Lynch & Duellman, 1997	----
	Pristimantis simonsii	Lynch, 1975b	Lynch, 1975b	Lynch, 1975b
	Pristimantis skydmainos	Duellman & Lehr, 2009	Duellman & Lehr, 2009	----
	Pristimantis spinosus	Lynch, 1979b	Lynch, 1979b	----
	Pristimantis stictogaster	Duellman & Hedges, 2005	Duellman & Hedges, 2005	----
	Pristimantis subsigillatus	Lynch & Duellman, 1997	Lynch & Duellman, 1997	----
	Pristimantis suetus	Lynch & Rueda-Almonacid, 1998	Lynch & Rueda-Almonacid, 1998	----
	Pristimantis supernatis	Lynch, 1979a	Lynch, 1979a	----
	Pristimantis surdus	Lynch, 1980c	Lynch, 1980c	MT pers. obs.
	Pristimantis taeniatus	Lynch, 1980a	Lynch, 1980a	----
	Pristimantis terraebolivaris	Rivero, 1961	Rivero, 1961	----
	Pristimantis thectopternus	Lynch, 1975a	Lynch, 1975a	----
	Pristimantis thymalopsoides	LAC pers. obs.	Lynch, 1976a	----
	Pristimantis thymelensis	Lynch, 1981a	Lynch, 1981a	----
	Pristimantis toftae	Duellman & Lehr, 2009	Duellman & Lehr, 2009	----
	Pristimantis truebae	Lynch & Duellman, 1997	Lynch & Duellman, 1997	----
	Pristimantis unistrigatus	Lynch & Duellman, 1980	Lynch & Duellman, 1980	Lynch, 1971
	Pristimantis urichi	Kaiser *et al.*, 1994b	Kaiser *et al.*, 1994b	----
	Pristimantis ventrimarmoratus	Lynch & Duellman, 1980	Lynch & Duellman, 1980	----
	Pristimantis verecundus	Lynch & Burrowes, 1990	Lynch & Burrowes, 1990	----
	Pristimantis versicolor	Lynch, 1979b	Lynch, 1979b	----
	Pristimantis vertebralis	Lynch, 1979a	Lynch, 1979a	----
	Pristimantis viejas	Lynch & Rueda-Almonacid, 1999	Lynch & Rueda-Almonacid, 1999	----
	Pristimantis walkeri	Lynch, 1974a	Lynch, 1974a	----
	Pristimantis wiensi	----	Duellman & Wild, 1993	----
	Pristimantis wnigrum	Lynch & Duellman, 1997	Lynch & Duellman, 1997	Lynch, 1971

Craugastoridae (cont.)	*Pristimantis zophus*	Lynch & Ardila-Robayo, 1999	Lynch & Ardila-Robayo, 1999	----
	Psychrophrynella iatamasi	Aguayo-Vedia & Harvey, 2001	----	----
	Psychrophrynella wettsteini	Lehr, 2006	----	Lynch, 1971
	Strabomantis anomalus	Lynch & Myers, 1983	Lynch & Myers, 1983	----
	Strabomantis biporcatus	Savage & Myers, 2002	Savage & Myers, 2002	----
	Strabomantis bufoniformis	Lynch, 2000	Lynch, 2000	Lynch, 1971
	Strabomantis necerus	Lynch, 1975c	Lynch, 1975c	JJSO pers. obs.
	Strabomantis sulcatus	Duellman & Lehr, 2009	Duellman & Lehr, 2009	Lynch, 1971
	Yunganastes ashkapara	Köhler, 2000b	Köhler, 2000b	----
	Yunganastes bisignatus	Köhler, 2000a	Köhler, 2000a	----
	Yunganastes fraudator	Lynch & McDiarmid, 1987	Lynch & McDiarmid, 1987	----
	Yunganastes mercedesae	Lynch & McDiarmid, 1987	Padial *et al.*, 2007	----
	Yunganastes pluvicanorus	de la Riva & Lynch, 1997	Padial *et al.*, 2007	----
Cycloramphidae	*Cycloramphus boraceiensis*	MOP pers. obs.; Heyer *et al.*, 1990	----	----
	Cycloramphus eleutherodactylus	Verdade, 2005	Verdade, 2005	Lynch, 1971
	Thoropa miliaris	----	Boulenger, 1882	Lynch, 1971
	Thoropa taophora	MOP pers. obs.	MOP pers. obs.	----
	Zachaenus parvulus	Verdade, 2005	Verdade, 2005	Lynch, 1971
Dendrobatidae	*Adelphobates castaneoticus*	----	----	TG pers. obs.
	Adelphobates galactonotus	----	----	TG pers. obs.
	Ameerega bassleri	----	TG pers. obs.	TG pers. obs.
	Ameerega bilinguis	Beirne & Witworth, 2011	TG pers. obs.; Beirne & Witworth, 2011	TG pers. obs.
	Ameerega hahneli	----	TG pers. obs.	TG pers. obs.
	Ameerega macero	----	Rodríguez & Myers, 1993	----
	Ameerega parvula	----	Boulenger, 1882	----

Dendrobatidae (cont.)	*Ameerega petersi*	----	TG pers. obs.; Lynch & Renjifo, 2001	TG pers. obs.
	Ameerega picta	----	TG pers. obs.	TG pers. obs.
	Ameerega silverstonei	TG pers. obs.	TG pers. obs.	TG pers. obs.
	Ameerega simulans	Myers *et al.*, 1998	Myers *et al.*, 1998	Myers *et al.*, 1998
	Ameerega trivittata	----	TG pers. obs.; Boulenger, 1882	TG pers. obs.; Laurent, 1942
	Andinobates claudiae	----	TG pers. obs.	TG pers. obs.
	Andinobates fulguritus	----	TG pers. obs.	TG pers. obs.
	Colostethus fugax	----	Coloma, 1995	----
	Colostethus inguinalis	Wever, 1985	Wever, 1985; Boulenger, 1882	Wever, 1985
	Colostethus latinasus	----	Boulenger, 1882	----
	Colostethus panamensis	----	TG pers. obs.	TG pers. obs.
	Colostethus pratti	----	TG pers. obs.	TG pers. obs.
	Dendrobates auratus	Nussbaum & Wu, 2007; Wever, 1985; Savage, 2002	Nussbaum & Wu, 2007; Wever, 1985	Nussbaum & Wu, 2007; Wever, 1985; TG pers. obs.
	Dendrobates tinctorius	----	TG pers. obs.; Boulenger, 1882	Parker, 1881; TG pers. obs.
	Dendrobates truncatus	----	TG pers. obs.	TG pers. obs.
	Epipedobates anthonyi	----	----	Haas, 1995
	Epipedobates boulengeri	----	TG pers. obs.	TG pers. obs.
	Epipedobates espinosai	----	TG pers. obs.	TG pers. obs.
	Excidobates captivus	----	TG pers. obs.	----
	Excidobates mysteriosus	----	Myers, 1982	----
	Hyloxalus awa	----	TG pers. obs.	TG pers. obs.
	Hyloxalus bocagei	Beirne & Witworth, 2011	TG pers. obs.; Boulenger, 1882; Beirne & Witworth, 2011	TG pers. obs.
	Hyloxalus chlorocraspedus	----	Caldwell, 2005	----
	Hyloxalus elachyhistus	----	TG pers. obs.	TG pers. obs.

(cont.)	*Hyloxalus nexipus*	----	Frost, 1986	----
	Hyloxalus pulchellus	----	TG pers. obs.; Boulenger, 1882	TG pers. obs.
	Hyloxalus sauli	----	TG pers. obs.	TG pers. obs.
	Hyloxalus subpunctatus	Lynch & Renjifo, 2001	TG pers. obs.	TG pers. obs.
	Hyloxalus sylvaticus	----	TG pers. obs.	TG pers. obs.
	Hyloxalus vertebralis	----	TG pers. obs.	TG pers. obs.
	Minyobates steyermarki	----	TG pers. obs.	TG pers. obs.
	Oophaga arborea	----	TG pers. obs.	TG pers. obs.
	Oophaga granulifera	----	TG pers. obs.	TG pers. obs.
	Oophaga histrionica	----	TG pers. obs.	TG pers. obs.; Laurent, 1942
	Oophaga lehmanni	----	TG pers. obs.	TG pers. obs.
	Oophaga pumilio	Savage, 2002	TG, Boulenger, 1882; Savage, 2002	TG pers. obs.
	Oophaga speciosa	----	TG pers. obs.	TG pers. obs.
	Oophaga sylvatica	----	TG pers. obs.	TG pers. obs.
	Oophaga vicentei	----	TG pers. obs.	TG pers. obs.
	Phyllobates aurotaenia	----	TG pers. obs.	TG pers. obs.
	Phyllobates bicolor	----	TG pers. obs.; Boulenger, 1882	Haas, 1995; TG pers. obs.
	Phyllobates lugubris	----	TG pers. obs.	TG pers. obs.
	Phyllobates terribilis	----	TG pers. obs.	TG pers. obs.
	Phyllobates vittatus	----	TG pers. obs.	TG pers. obs.
	Ranitomeya amazonica	----	Brown *et al.*, 2011	----
	Ranitomeya imitator	----	TG pers. obs.	TG pers. obs.
	Ranitomeya reticulata	----	TG pers. obs.	TG pers. obs.
	Ranitomeya ventrimaculata	Beirne & Witworth, 2011	TG pers. obs.; Beirne & Witworth, 2011	TG pers. obs.
	Silverstoneia flotator	----	TG pers. obs.	TG pers. obs.
	Silverstoneia nubicola	----	TG pers. obs.	TG pers. obs.

Dicroglossidae	*Euphlyctis cyanophlyctis*	Scott, 2005	Scott, 2005; Parker, 1881; Boulenger, 1882	Scott, 2005; Parker, 1881
	Euphlyctis hexadactylus	Boulenger, 1882	Boulenger, 1882	----
	Fejervarya cancrivora	Zhao & Adler, 1993	Inger, 1966; Zhao & Adler, 1993	----
	Fejervarya caperata	Kuramoto *et al.*, 2007	Kuramoto *et al.*, 2007	----
	Fejervarya granosa	Kuramoto *et al.*, 2007	Kuramoto *et al.*, 2007	----
	Fejervarya kudremukhensis	Kuramoto *et al.*, 2007	Kuramoto *et al.*, 2007	----
	Fejervarya iskandari	----	Veith *et al.*, 2001	----
	Fejervarya limnocharis	----	Liu, 1950; Inger, 1966	----
	Fejervarya mudduraja	Kuramoto *et al.*, 2007	Kuramoto *et al.*, 2007; Chandramouli & Dutta, 2015	----
	Fejervarya rufescens	----	Boulenger, 1882	----
	Fejervarya sahyadris	----	Chandramouli & Dutta, 2015	Chandramouli & Dutta, 2015
	Hoplobatrachus occipitalis	Scott, 2005	Boulenger, 1882; Scott, 2005	Scott, 2005
	Hoplobatrachus rugulosus	----	Inger & Stuebing, 1997	----
	Hoplobatrachus tigerinus	Glaw & Vences, 2007	Boulenger, 1882; Glaw & Vences, 2007	Parker, 1881
	Ingerana tenasserimensis	----	Iskandar *et al.*, 2011	----
	Limnonectes asperatus	----	Inger *et al.*, 1996	----
	Limnonectes blythii	Scott, 2005; Emerson & Berrigan, 1993	Scott, 2005; Emerson & Berrigan, 1993	Scott, 2005
	Limnonectes dabanus	----	Stuart *et al.*, 2006	----
	Limnonectes doriae	----	Boulenger, 1920	----
	Limnonectes finchi	Emerson & Berrigan, 1993	Emerson & Berrigan, 1993	----
	Limnonectes hascheanus	Emerson & Berrigan, 1993	Emerson & Berrigan, 1993	----
	Limnonectes ibanorum	----	Inger, 1966	----
	Limnonectes kuhlii	Inger, 1966; Boulenger, 1882	Inger, 1966	Parker, 1881
	Limnonectes laticeps	Inger, 1966; Boulenger, 1882	Inger, 1966	----
	Limnonectes leporinus	Inger & Stuebing, 1997	Inger & Stuebing, 1997	----

Dicroglossidae (cont.)	*Limnonectes leytensis*	Emerson & Berrigan, 1993	Emerson & Berrigan, 1993	----
	Limnonectes macrodon	----	Inger, 1966; Boulenger, 1882	----
	Limnonectes magnus	----	Boulenger, 1920	----
	Limnonectes malesianus	Inger & Stuebing, 1997	Inger & Stuebing, 1997	----
	Limnonectes microdiscus	----	Inger, 1966	----
	Limnonectes microtympanum	----	Boulenger, 1920	----
	Limnonectes modestus	----	Boulenger, 1882	----
	Limnonectes palavanensis	Inger & Stuebing, 1997	Inger & Stuebing, 1997	----
	Limnonectes paramacrodon	Inger & Stuebing, 1997	Inger & Stuebing, 1997	----
	Limnonectes plicatellus	Emerson & Berrigan, 1993	Emerson & Berrigan, 1993	----
	Limnonectes woodworthi	----	Inger, 1954	----
	Nannophrys ceylonensis	Scott, 2005	Scott, 2005	Clarke, 1983; Scott, 2005
	Nannophrys marmorata	----	----	Clarke, 1983
	Nanorana aenea	----	Dubois & Ohler, 2005	----
	Nanorana arnoldi	----	Dubois, 1975	----
	Nanorana liebigii	Ohler & Dubois, 2006	Boulenger, 1882; Günther, 1860	Dubois, 1975
	Nanorana parkeri	Scott, 2005	Scott, 2005	Scott, 2005
	Nanorana pleski	Günther, 1896; Liu, 1950; Lu & Yang, 1995	Günther, 1896; Liu, 1950; Lu & Yang, 1995	Lu & Yang, 1995
	Nanorana quadranus	----	Ohler & Dubois, 2006	----
	Nanorana ventripunctata	Lu & Yang, 1995	----	Lu & Yang, 1995
	Nanorana yunnanensis	Zhao & Adler, 1993	Boulenger, 1882	----
	Occidozyga baluensis		Inger, 1966	----
	Occidozyga laevis	Boulenger, 1882 absent; Scott, 2005 present	Inger, 1966; Scott, 2005	Scott, 2005
	Occidozyga lima	Zhao & Adler, 1993	----	Laurent, 1943b
	Quasipaa delacouri	Dubois, 1975	----	----
	Quasipaa shini	Zhao & Adler, 1993	Zhao & Adler, 1993	----

Dicroglossidae (cont.)	*Quasipaa spinosa*	----	----	Dubois, 1975
	Sphaerotheca breviceps	----	Boulenger, 1882	----
	Sphaerotheca dobsonii	----	Boulenger, 1882	----
Eleutherodactylidae	*Adelophryne adiastola*	Hoogmoed & Lescure, 1984	Hoogmoed & Lescure, 1984	----
	Adelophryne baturitensis	Hoogmoed *et al.*, 1994	Hoogmoed *et al.*, 1994	----
	Adelophryne gutturosa	Hoogmoed & Lescure, 1984; Kok & Kalamandeen, 2008	Hoogmoed & Lescure, 1984; Kok & Kalamandeen, 2008	----
	Adelophryne maranguapensis	Hoogmoed *et al.*, 1994	Hoogmoed *et al.*, 1994	----
	Adelophryne pachydactyla	Hoogmoed *et al.*, 1994	Hoogmoed *et al.*, 1994	----
	Adelophryne patamona	MacCulloch *et al.*, 2008; Kok & Kalamandeen, 2008	MacCulloch *et al.*, 2008; Kok & Kalamandeen, 2008	----
	Diasporus diastema	Savage, 2002	Savage, 2002	Lynch, 1971
	Diasporus hylaeformis	Savage, 2002	Savage, 2002	----
	Diasporus vocator	Savage, 2002; Taylor, 1955	Savage, 2002	----
	Eleutherodactylus abbotti	Cochran, 1923	Cochran, 1923	Lynch, 1971
	Eleutherodactylus acmonis	Díaz & Cádiz, 2008	Díaz & Cádiz, 2008	----
	Eleutherodactylus adelus	Diaz *et al.*, 2003	Diaz *et al.*, 2003	----
	Eleutherodactylus albipes	Barbour & Shreve, 1937	Barbour & Shreve, 1937	----
	Eleutherodactylus alcoae	Schwartz, 1976b	Schwartz, 1976b	----
	Eleutherodactylus alticola	Hedges *et al.*, 2008	Hedges *et al.*, 2008	----
	Eleutherodactylus amadeus	Hedges *et al.*, 1987	Hedges *et al.*, 1987	----
	Eleutherodactylus amplinympha	Kaiser *et al.*, 1994a	Kaiser *et al.*, 1994a	----
	Eleutherodactylus andrewsi	Hedges *et al.*, 2008	Hedges *et al.*, 2008	----
	Eleutherodactylus antillensis	Hedges *et al.*, 2008	Hedges *et al.*, 2008	Lynch, 1971
	Eleutherodactylus apostates	Schwartz, 1973	Schwartz, 1973	----
	Eleutherodactylus armstrongi	Noble & Hassler, 1933	Noble & Hassler, 1933	Lynch, 1971
	Eleutherodactylus atkinsi	Dunn, 1925; Nussbaum & Wu, 2007	Dunn, 1925; Nussbaum & Wu, 2007	Lynch, 1971
	Eleutherodactylus audanti	Schwartz, 1966	Schwartz, 1966	Lynch, 1971

Eleutherodactylidae (cont.)	*Eleutherodactylus auriculatoides*	Cope, 1862b, Noble, 1923	Cope, 1862b, Noble, 1923	Lynch, 1971
	Eleutherodactylus auriculatus	Cope, 1862b	Cope, 1862b	----
	Eleutherodactylus bakeri	Cochran, 1935	Cochran, 1935	----
	Eleutherodactylus barlagnei	Lynch, 1965a	Lynch, 1965a	Lynch, 1971
	Eleutherodactylus bartonsmithi	Díaz & Cádiz, 2008	Díaz & Cádiz, 2008	----
	Eleutherodactylus blairhedgesi	Estrada *et al.*, 1998	Estrada *et al.*, 1998	----
	Eleutherodactylus bothroboans	Schwartz, 1965b	Schwartz, 1965b	----
	Eleutherodactylus bresslerae	Díaz & Cádiz, 2008	Díaz & Cádiz, 2008	----
	Eleutherodactylus brevirostris	Hedges *et al.*, 2008	Hedges *et al.*, 2008	----
	Eleutherodactylus brittoni	Schmidt, 1920	Schmidt, 1920	----
	Eleutherodactylus caribe	Hedges & Thomas, 1992a	Hedges & Thomas, 1992a	----
	Eleutherodactylus casparii	Díaz & Cádiz, 2008	Díaz & Cádiz, 2008	----
	Eleutherodactylus cavernicola	Lynn, 1954	Lynn, 1954	----
	Eleutherodactylus chlorophenax	Schwartz, 1976a	Schwartz, 1976a	----
	Eleutherodactylus cochranae	Hedges *et al.*, 2008	Hedges *et al.*, 2008	Lynch, 1971
	Eleutherodactylus cooki	Hedges *et al.*, 2008	Hedges *et al.*, 2008	----
	Eleutherodactylus coqui	Zug, 2013	Zug, 2013	Lynch, 1971
	Eleutherodactylus corona	Hedges & Thomas, 1992b	Hedges & Thomas, 1992b	----
	Eleutherodactylus counouspeus	Schwartz, 1964	Schwartz, 1964	----
	Eleutherodactylus cubanus	Barbour & Shreve, 1937	Barbour & Shreve, 1937	----
	Eleutherodactylus cundalli	Hedges *et al.*, 2008	Hedges *et al.*, 2008	Lynch, 1971
	Eleutherodactylus cuneatus	Cope, 1862b	Cope, 1862b	----
	Eleutherodactylus darlingtoni	Cochran, 1935	Cochran, 1935	----
	Eleutherodactylus dimidiatus	Cope, 1862b	Cope, 1862b	----

Eleutherodactylidae (cont.)	*Eleutherodactylus dolomedes*	Hedges & Thomas, 1992b	Hedges & Thomas, 1992b	----
	Eleutherodactylus eileenae	Dunn, 1926	Dunn, 1926	----
	Eleutherodactylus emiliae	Dunn, 1926	Dunn, 1926	----
	Eleutherodactylus eneidae	Rivero, 1959	Rivero, 1959	Lynch, 1971
	Eleutherodactylus etheridgei	Schwartz, 1958b	Schwartz, 1958b	----
	Eleutherodactylus eunaster	Schwartz, 1973	Schwartz, 1973	----
	Eleutherodactylus flavescens	Noble, 1923	Noble, 1923	----
	Eleutherodactylus fowleri	Schwartz, 1973	Schwartz, 1973	----
	Eleutherodactylus furcyensis	Hedges *et al.*, 2008	Hedges *et al.*, 2008	Lynch, 1971
	Eleutherodactylus fuscus	Hedges *et al.*, 2008	Hedges *et al.*, 2008	----
	Eleutherodactylus glamyrus	Estrada & Hedges, 1997b	Estrada & Hedges, 1997b	----
	Eleutherodactylus glandulifer	Hedges *et al.*, 2008	Hedges *et al.*, 2008	----
	Eleutherodactylus glanduliferoides	Hedges *et al.*, 2008	Hedges *et al.*, 2008	----
	Eleutherodactylus glaphycompus	Schwartz, 1973	Schwartz, 1973	----
	Eleutherodactylus glaucoreius	Hedges *et al.*, 2008	Hedges *et al.*, 2008	----
	Eleutherodactylus goini	Díaz & Cádiz, 2008	Díaz & Cádiz, 2008	----
	Eleutherodactylus gossei	Hedges *et al.*, 2008	Hedges *et al.*, 2008	Lynch, 1971
	Eleutherodactylus grabhami	Hedges *et al.*, 2008	Hedges *et al.*, 2008	----
	Eleutherodactylus grahami	Schwartz, 1979	Schwartz, 1979	----
	Eleutherodactylus greyi	Díaz & Cádiz, 2008	Díaz & Cádiz, 2008	----
	Eleutherodactylus griphus	Crombie, 1986	Crombie, 1986	----
	Eleutherodactylus gryllus	Schmidt, 1920	Schmidt, 1920	----
	Eleutherodactylus guanahacabibes	Díaz & Cádiz, 2008	Díaz & Cádiz, 2008	----
	Eleutherodactylus guantanamera	Hedges *et al.*, 1992	Hedges *et al.*, 1992	----
	Eleutherodactylus gundlachi	Barbour, 1914	Barbour, 1914	----
	Eleutherodactylus haitianus	Cochran, 1941	Cochran, 1941	Lynch, 1971

Eleutherodactylidae (cont.)	*Eleutherodactylus hedricki*	Rivero, 1963	Rivero, 1963	----
	Eleutherodactylus heminota	Hedges *et al.*, 2008; Nussbaum & Wu, 2007	Hedges *et al.*, 2008; Nussbaum & Wu, 2007	Nussbaum & Wu, 2007
	Eleutherodactylus hypostenor	Schwartz, 1965b	Schwartz, 1965b	----
	Eleutherodactylus iberia	Estrada & Hedges, 1996	Estrada & Hedges, 1996	----
	Eleutherodactylus inoptatus	Barbour, 1914	Barbour, 1914	Lynch, 1971
	Eleutherodactylus intermedius	Díaz & Cádiz, 2008	Díaz & Cádiz, 2008	----
	Eleutherodactylus ionthus	Díaz & Cádiz, 2008	Díaz & Cádiz, 2008	----
	Eleutherodactylus jamaicensis	Barbour, 1910	Barbour, 1910	----
	Eleutherodactylus jaumei	Estrada & Alonso, 1997	Estrada & Alonso, 1997	----
	Eleutherodactylus johnstonei	Barbour, 1914	Barbour, 1914	----
	Eleutherodactylus jugans	Cochran, 1935	Cochran, 1935	Lynch, 1971
	Eleutherodactylus junori	Hedges *et al.*, 2008	Hedges *et al.*, 2008	----
	Eleutherodactylus klinikowskii	Díaz & Cádiz, 2008	Díaz & Cádiz, 2008	----
	Eleutherodactylus lamprotes	Schwartz, 1973	Schwartz, 1973	----
	Eleutherodactylus leberi	Díaz & Cádiz, 2008	Díaz & Cádiz, 2008	----
	Eleutherodactylus lentus	Hedges *et al.*, 2008	Hedges *et al.*, 2008	Lynch, 1971
	Eleutherodactylus leoncei	Hedges, 1992	Hedges, 1992	----
	Eleutherodactylus limbatus	Cope, 1862b, Nussbaum & Wu, 2007	Cope, 1862b, Nussbaum & Wu, 2007	Lynch, 1971; Nussbaum & Wu, 2007
	Eleutherodactylus locustus	Schmidt, 1920	Schmidt, 1920	Lynch, 1971
	Eleutherodactylus luteolus	Hedges *et al.*, 2008	Hedges *et al.*, 2008	----
	Eleutherodactylus maestrensis	Diaz *et al.*, 2005	Diaz *et al.*, 2005	----
	Eleutherodactylus mariposa	Hedges *et al.*, 1992	Hedges *et al.*, 1992	----
	Eleutherodactylus marnockii	Hedges *et al.*, 1992	Hedges *et al.*, 1992	Lynch, 1971
	Eleutherodactylus martinicensis	Lynch, 1965a	Lynch, 1965a	Lynch, 1971
	Eleutherodactylus melacara	Hedges *et al.*, 1992	Hedges *et al.*, 1992	----
	Eleutherodactylus minutus	Noble, 1923	Noble, 1923	Lynch, 1971

Eleutherodactylidae (cont.)	*Eleutherodactylus monensis*	Hedges *et al.*, 2008	Hedges *et al.*, 2008	----
	Eleutherodactylus nitidus	Hedges *et al.*, 2008	Hedges *et al.*, 2008	Lynch, 1971
	Eleutherodactylus nortoni	Hedges *et al.*, 2008	Hedges *et al.*, 2008	----
	Eleutherodactylus nubicola	Hedges *et al.*, 2008	Hedges *et al.*, 2008	Lynch, 1971
	Eleutherodactylus orcutti	Hedges *et al.*, 2008	Hedges *et al.*, 2008	Lynch, 1971
	Eleutherodactylus orientalis	Díaz & Cádiz, 2008	Díaz & Cádiz, 2008	----
	Eleutherodactylus oxyrhyncus	Hedges *et al.*, 2008	Hedges *et al.*, 2008	----
	Eleutherodactylus pantoni	Hedges *et al.*, 2008	Hedges *et al.*, 2008	Lynch, 1971
	Eleutherodactylus parabates	Schwartz, 1964	Schwartz, 1964	----
	Eleutherodactylus parapelates	Hedges & Thomas, 1987	Hedges & Thomas, 1987	----
	Eleutherodactylus patriciae	Schwartz, 1965 "1964"	Schwartz, 1965 "1964"	Lynch, 1971
	Eleutherodactylus paulsoni	Schwartz, 1964	Schwartz, 1964	----
	Eleutherodactylus pentasyringos	Hedges *et al.*, 2008	Hedges *et al.*, 2008	----
	Eleutherodactylus pezopetrus	Hedges *et al.*, 2008	Hedges *et al.*, 2008	----
	Eleutherodactylus pictissimus	Cochran, 1935	Cochran, 1935	Lynch, 1971
	Eleutherodactylus pinarensis	Dunn, 1926	Dunn, 1926	----
	Eleutherodactylus pinchoni	Hedges *et al.*, 2008	Hedges *et al.*, 2008	----
	Eleutherodactylus pipilans	Taylor, 1940b	Taylor, 1940b	Lynch, 1971
	Eleutherodactylus pituinus	Schwartz, 1965a	Schwartz, 1965a	----
	Eleutherodactylus planirostris	Cope, 1862b	Cope, 1862b	Lynch, 1971
	Eleutherodactylus poolei	Cochran, 1938	Cochran, 1938	----
	Eleutherodactylus portoricensis	Schmidt, 1927	Schmidt, 1927	Lynch, 1971
	Eleutherodactylus principalis	Estrada & Hedges, 1997c	Estrada & Hedges, 1997c	----
	Eleutherodactylus probalaeus	Schwartz, 1976b	Schwartz, 1976b	----
	Eleutherodactylus rhodesi	Hedges *et al.*, 2008	Hedges *et al.*, 2008	----
	Eleutherodactylus richmondi	Schmidt, 1920	Schmidt, 1920	Lynch, 1971
	Eleutherodactylus ricordii	Díaz & Cádiz, 2008	Díaz & Cádiz, 2008	Lynch, 1971

Eleutherodactylidae (cont.)	*Eleutherodactylus riparius*	Diaz *et al.*, 2001	Diaz *et al.*, 2001	----
	Eleutherodactylus rivularis	Diaz *et al.*, 2001	Diaz *et al.*, 2001	----
	Eleutherodactylus rogersi	Goin, 1955	Goin, 1955	----
	Eleutherodactylus ronaldi	Diaz *et al.*, 2001	Diaz *et al.*, 2001	----
	Eleutherodactylus rufifemoralis	Noble & Hassler, 1933	Noble & Hassler, 1933	----
	Eleutherodactylus ruthae	Cochran, 1941	Cochran, 1941	Lynch, 1971
	Eleutherodactylus schmidti	Noble, 1923	Noble, 1923	----
	Eleutherodactylus schwartzi	Hedges *et al.*, 2008	Hedges *et al.*, 2008	----
	Eleutherodactylus sciagraphus	Schwartz, 1973	Schwartz, 1973	----
	Eleutherodactylus simulans	Díaz & Fong, 2001	Díaz & Fong, 2001	----
	Eleutherodactylus sisyphodemus	Crombie, 1977	Crombie, 1977	----
	Eleutherodactylus sommeri	Schwartz, 1977	Schwartz, 1977	----
	Eleutherodactylus symingtoni	Schwartz, 1957	Schwartz, 1957	----
	Eleutherodactylus thomasi	Schwartz, 1959	Schwartz, 1959	----
	Eleutherodactylus thorectes	Hedges, 1988	Hedges, 1988	----
	Eleutherodactylus toa	Estrada & Hedges, 1991	Estrada & Hedges, 1991	----
	Eleutherodactylus tonyi	Estrada & Hedges, 1997a	Estrada & Hedges, 1997a	----
	Eleutherodactylus turquinensis	Barbour & Shreve, 1937	Barbour & Shreve, 1937	----
	Eleutherodactylus unicolor	Hedges *et al.*, 2008	Hedges *et al.*, 2008	----
	Eleutherodactylus varians	Díaz & Cádiz, 2008	Díaz & Cádiz, 2008	----
	Eleutherodactylus varleyi	Dunn, 1925; Nussbaum & Wu, 2007	Dunn, 1925; Nussbaum & Wu, 2007	Nussbaum & Wu, 2007
	Eleutherodactylus ventrilineatus	Hedges *et al.*, 2008	Hedges *et al.*, 2008	----
	Eleutherodactylus weinlandi	Barbour, 1914	Barbour, 1914	Lynch, 1971
	Eleutherodactylus wetmorei	Cochran, 1932	Cochran, 1932	----

Eleutherodactylidae (cont.)	*Eleutherodactylus wightmanae*	Schmidt, 1920	Schmidt, 1920	Lynch, 1971
	Eleutherodactylus zeus	Schwartz, 1958a	Schwartz, 1958a	----
	Eleutherodactylus zugi	Schwartz, 1958a	Schwartz, 1958a	Lynch, 1971
	Phyzelaphryne miriamae	Heyer, 1977	Heyer, 1977	----
Heleophrynidae	*Hadromophryne natalensis*	du Preez & Carruthers, 2009	----	Lynch, 1971
	Heleophryne purcelli	Scott, 2005; du Preez & Carruthers, 2009	Scott, 2005	Ramaswami, 1944; Scott, 2005
Hemiphractidae	*Flectonotus fitzgeraldi*			
	Flectonotus pygmaeus	----	Duellman *et al.*, 2011	----
	Gastrotheca argenteovirens	----	Cochran & Goin, 1970	----
	Gastrotheca atympana	Duellman, 2015	Duellman, 2015	Duellman, 2015
	Gastrotheca aureomaculata	Duellman, 2015	Cochran & Goin, 1970; Duellman, 2015	----
	Gastrotheca christiani	Laurent *et al.*, 1986	Laurent *et al.*, 1986	MOP pers. obs.
	Gastrotheca chrysosticta	Laurent *et al.*, 1986	Laurent *et al.*, 1986	----
	Gastrotheca cornuta	Cochran & Goin, 1970	Cochran & Goin, 1970	----
	Gastrotheca dendronastes	----	Duellman, 2015	Duellman, 2015
	Gastrotheca dunni	----	Duellman, 2015	Duellman, 2015
	Gastrotheca excubitor	Catenazzi *et al.*, 2013	Catenazzi *et al.*, 2013	----
	Gastrotheca fissipes	Mendes *et al.*, 2012	Mendes *et al.*, 2012	----
	Gastrotheca galeata	----	Duellman, 2015	Duellman, 2015
	Gastrotheca gracilis	Laurent *et al.*, 1986	Laurent *et al.*, 1986	----
	Gastrotheca griswoldi	----	Duellman, 2015	Duellman, 2015
	Gastrotheca guentheri	----	Duellman, 2015	Duellman, 2015
	Gastrotheca helenae	Duellman, 2015	Cochran & Goin, 1970	Duellman, 2015
	Gastrotheca litonedis	----	Duellman, 2015	Duellman, 2015
	Gastrotheca longipes	----	Duellman, 2015	Duellman, 2015
	Gastrotheca marsupiata	----	Boulenger, 1882	Parker, 1881

Hemiphractidae (cont.)	*Gastrotheca monticola*	----	Duellman, 2015	Duellman, 2015
	Gastrotheca nicefori	Lynch & Renjifo, 2001; Cochran & Goin, 1970	Lynch & Renjifo, 2001; Cochran & Goin, 1970	----
	Gastrotheca ochoai	----	Duellman, 2015	Duellman, 2015
	Gastrotheca orophylax	----	Duellman, 2015	Duellman, 2015
	Gastrotheca peruana	----	Duellman, 2015	Duellman, 2015
	Gastrotheca plumbea	----	Boulenger, 1882	----
	Gastrotheca pseustes	----	Duellman, 2015	Duellman, 2015
	Gastrotheca psychrophila	----	Duellman, 2015	Duellman, 2015
	Gastrotheca riobambae	MOP pers. obs.; Duellman, 2015	MOP pers. obs.; Duellman, 2015	----
	Gastrotheca ruizi	----	Duellman, 2015	Duellman, 2015
	Gastrotheca stictopleura	----	Duellman, 2015	Duellman, 2015
	Gastrotheca trachyceps	----	Duellman, 2015	Duellman, 2015
	Gastrotheca weinlandii	----	Duellman, 2015	Duellman, 2015
	Gastrotheca zeugocystis	----	Duellman, 2015	Duellman, 2015
	Gastrotheca walkeri	Duellman, 2015	Mendelson *et al.*, 2000	Mendelson *et al.*, 2000
	Hemiphractus bubalus	----	----	Duellman, 2015
	Hemiphractus helioi	----	Duellman, 2015	Duellman, 2015
	Hemiphractus proboscideus	----	Trueb, 1974	Trueb, 1974
	Hemiphractus scutatus	----	Trueb, 1974	Trueb, 1974
	Stefania evansi	Kok & Kalamandeen, 2008	Kok & Kalamandeen, 2008	Duellman & Hoogmoed, 1984
	Stefania ginesi	Señaris *et al.*, 2014	Señaris *et al.*, 2014	----
	Stefania scalae	----	----	Duellman, 2015
	Stefania schuberti	Señaris *et al.*, 2014	Señaris *et al.*, 2014	----
Hemisotidae	*Hemisus marmoratus*	Scott, 2005; du Preez & Carruthers, 2009	Scott, 2005	Scott, 2005
Hylidae	*Acris crepitans*	----	Boulenger, 1882	Duellman, 1970b, Maglia *et al.*, 2007
	Acris gryllus	----	Boulenger, 1882	----

Hylidae (cont.)	*Agalychnis annae*	----	Duellman, 1963	----
	Agalychnis aspera	----	Boulenger, 1882	----
	Agalychnis callidryas	Wever, 1985	Boulenger, 1882	Duellman, 1970a, Wever, 1985
	Agalychnis dacnicolor	Wever, 1985; Boulenger, 1882	Boulenger, 1882	Duellman, 1970a, Wever, 1985
	Agalychnis granulosa	----	----	Cruz, 1990
	Agalychnis hulli	Beirne & Witworth, 2011	Beirne & Witworth, 2011	----
	Agalychnis lemur	----	Boulenger, 1882	Duellman, 1970a
	Agalychnis litodryas	----	Duellman & Trueb, 1967	----
	Agalychnis moreletii	----	Boulenger, 1882	Duellman, 1970a
	Agalychnis saltator	----	Savage & Heyer, 1967	----
	Agalychnis spurrelli	----	----	Duellman, 1970a
	Anotheca spinosa	Savage, 2002	Boulenger, 1882; Savage, 2002	----
	Aparasphenodon brunoi	----	----	Trueb, 1970b
	Aplastodiscus albofrenatus	Lutz, 1973	Lutz, 1973	----
	Aplastodiscus albosignatus	Lutz, 1973	Lutz, 1973	----
	Aplastodiscus arildae	----	Cruz & Peixoto, 1987 "1985"	----
	Aplastodiscus cavicola	----	Cruz & Peixoto, 1985 "1984"	----
	Aplastodiscus eugenioi	----	Carvalho-e-Silva & Carvalho-e-Silva, 2006	----
	Aplastodiscus leucopygius	----	Cruz & Peixoto, 1985 "1984"	----
	Aplastodiscus perviridis	MOP pers. obs.	MOP pers. obs.	MOP pers. obs.
	Aplastodiscus weygoldti	----	Cruz & Peixoto, 1987 "1985"	----
	Argenteohyla siemersi	Trueb, 1970c	Trueb, 1970c	Trueb, 1970c
	Bokermannohyla astartea	----	Bokermann, 1967a	----
	Bokermannohyla circumdata	Lutz, 1973	Boulenger, 1882; Lutz, 1973	----
	Bokermannohyla hylax	----	Heyer, 1985	----
	Bokermannohyla martinsi	----	Bokermann, 1964a	----

Hylidae (cont.)	*Bromeliohyla bromeliacia*	----	Duellman, 1970b	Duellman, 1970b
	Charadrahyla nephila	----	Mendelson & Campbell, 1999	----
	Charadrahyla taeniopus	----	Duellman, 1970b	----
	Corythomantis greeningi	----	----	Trueb, 1970b
	Cruziohyla calcarifer	McCranie & Wilson, 2002	Boulenger, 1882; McCranie & Wilson, 2002	----
	Dendropsophus anceps	Lutz, 1973	Lutz, 1973	----
	Dendropsophus berthalutzae	Lutz, 1973	Lutz, 1973	----
	Dendropsophus bifurcus	----	Duellman, 1974	----
	Dendropsophus bipunctatus	Lutz, 1973	Boulenger, 1882; Lutz, 1973	----
	Dendropsophus branneri	Lutz, 1973	Lutz, 1973	----
	Dendropsophus brevifrons	----	Duellman & Crump, 1974	----
	Dendropsophus ebraccatus	Wever, 1985	----	Wever, 1985
	Dendropsophus elegans	----	Lutz, 1973	----
	Dendropsophus gaucheri	----	Lescure & Marty, 2000	----
	Dendropsophus juliani	----	Moravec *et al.*, 2006	----
	Dendropsophus koechlini	----	Duellman & Trueb, 1989	----
	Dendropsophus labialis	----	Boulenger, 1882	----
	Dendropsophus marmoratus	Lutz, 1973	Boulenger, 1882; Lutz, 1973	----
	Dendropsophus melanargyreus	----	Bokermann, 1964b	----
	Dendropsophus microcephalus	Wever, 1985	Wever, 1985	Wever, 1985
	Dendropsophus minutus	Lima *et al.*, 2006	Lima *et al.*, 2006	MOP pers. obs.
	Dendropsophus miyatai	----	Vigle & Goberdhan-Vigle, 1990	----
	Dendropsophus nanus	Lutz, 1973	Lutz, 1973	----
	Dendropsophus parviceps	----	Boulenger, 1882	----
	Dendropsophus pelidna	----	Duellman, 1989	----

Hylidae (cont.)	*Dendropsophus rhodopeplus*	----	Duellman, 1972a	----
	Dendropsophus robertmertensi	----	Duellman, 1970b	----
	Dendropsophus rubicundulus	----	Boulenger, 1882	----
	Dendropsophus sarayacuensis	----	Duellman, 1974	----
	Dendropsophus sartoti	----	Duellman, 1970b	----
	Dendropsophus schubarti	Duellman, 2005	Duellman, 2005	Duellman, 2005
	Dendropsophus seniculus	Lutz, 1973	Boulenger, 1882; Lutz, 1973	----
	Dendropsophus timbeba	Duellman, 2005	Duellman, 2005	----
	Dendropsophus triangulum	----	Boulenger, 1882	----
	Dendropsophus tritaeniatus	----	Bokermann, 1965	----
	Diaglena spatulata	----	Duellman, 1970b	Duellman, 1970b
	Duellmanohyla rufioculis	----	Duellman, 1970b	----
	Duellmanohyla soralia	McCranie & Wilson, 2002	McCranie & Wilson, 2002	----
	Ecnomiohyla miliaria	Savage, 2002	Savage, 2002	----
	Ecnomiohyla miotympanum	----	Duellman, 1970b	Duellman, 1970b
	Exerodonta abdivita	----	Duellman, 2001	----
	Exerodonta chimalaà	----	Mendelson & Campbell, 1994	----
	Exerodonta melanomma	----	----	Duellman, 1970b
	Exerodonta perkinsi	----	Duellman, 2001	----
	Exerodonta smaragdina	----	Duellman, 1970b	----
	Exerodonta sumichrasti	----	Boulenger, 1882	Duellman, 1970b
	Exerodonta xera	----	Duellman, 2001	----
	Hyla annectans	----	Liu, 1950	----
	Hyla andersonii	Dood, 2013	Dood, 2013	----
	Hyla arborea	----	Boulenger, 1882	Parker, 1881
	Hyla arenicolor	----	----	Duellman, 1970b
	Hyla avivoca	Dood, 2013	Dood, 2013	----
	Hyla chinensis	----	Boulenger, 1882	Gaudin, 1974

Hylidae (cont.)	*Hyla chrysoscelis*	Dood, 2013	Dood, 2013	----
	Hyla cinerea	Wever, 1985	Boulenger, 1882	Wever, 1985; Gaudin1974
	Hyla euphorbiacea	----	Duellman, 1970b	----
	Hyla eximia	----	----	Duellman, 1970b
	Hyla femoralis	Dood, 2013	Dood, 2013	----
	Hyla gratiosa	Dood, 2013	Dood, 2013	----
	Hyla meridionalis	Schleich *et al.*, 1996	Schleich *et al.*, 1996	----
	Hyla plicata	----	Duellman, 1970b	----
	Hyla squirella	Dood, 2013	Dood, 2013	----
	Hyla versicolor	Wever, 1985	----	Wever, 1985
	Hyla walkeri	----	Duellman, 1970b	----
	Hyla wrightorum	Dood, 2013	Dood, 2013	----
	Hyloscirtus alytolylax	----	Duellman, 1972a	----
	Hyloscirtus armatus	----	Duellman *et al.*, 1997	----
	Hyloscirtus colymba	----	----	Duellman, 1970b
	Hyloscirtus larinopygion	----	Rivera-Correa & Faivovich, 2013	----
	Hyloscirtus lindae	----	----	Coloma *et al.*, 2012
	Hyloscirtus pacha	----	----	Coloma *et al.*, 2012
	Hyloscirtus palmeri	Savage, 2002	Savage, 2002	----
	Hyloscirtus pantostictus	----	----	Coloma *et al.*, 2012
	Hyloscirtus phyllognathus	----	Duellman, 1972a	----
	Hyloscirtus psarolaimus	----	----	Coloma *et al.*, 2012
	Hyloscirtus ptychodactylus	----	----	Coloma *et al.*, 2012
	Hyloscirtus simmonsi	----	Duellman, 1989	----
	Hyloscirtus staufferorum	----	----	Coloma *et al.*, 2012
	Hyloscirtus tapichalaca	----	Kizirian *et al.*, 2003	----
	Hyloscirtus tigrinus	----	Mueses-Cisneros & Anganoy-Criollo, 2008	----

Hylidae (cont.)	*Hypsiboas albomarginatus*	Lutz, 1973	Lutz, 1973	Parker, 1881
	Hypsiboas albopunctatus	----	Lutz, 1973	----
	Hypsiboas andinus	----	Barrio, 1965	----
	Hypsiboas balzani	----	Duellman *et al.*, 1997	----
	Hypsiboas bischoffi	Lutz, 1973	Lutz, 1973	----
	Hypsiboas boans	Lima *et al.*, 2006	Boulenger, 1882; Lima *et al.*, 2006	----
	Hypsiboas caingua	----	Carrizo, 1990	----
	Hypsiboas calcaratus		Caminer & Ron, 2014	
	Hypsiboas cinerascens	Lima *et al.*, 2006	Lima *et al.*, 2006	----
	Hypsiboas cordobae	----	Barrio, 1965	----
	Hypsiboas crepitans	----	Lutz, 1973	----
	Hypsiboas dentei	----	Bokermann, 1967b	----
	Hypsiboas ericae	----	Caramaschi & Cruz, 2000	----
	Hypsiboas faber	Lutz, 1973	Boulenger, 1882	----
	Hypsiboas fasciatus	----	Boulenger, 1882	----
	Hypsiboas geographicus	Boulenger, 1882	Boulenger, 1882	----
	Hypsiboas guentheri	----	Langone, 1997	----
	Hypsiboas heilprini	----	Trueb & Tyler, 1974	----
	Hypsiboas joaquini	----	Garcia *et al.*, 2003	----
	Hypsiboas lanciformis	Lutz, 1973; Lima *et al.*, 2006	Boulenger, 1882; Lutz, 1973; Lima *et al.*, 2006	----
	Hypsiboas latistriatus	----	Caramaschi & Cruz, 2004	----
	Hypsiboas leptolineatus	----	Braun & Braun, 1977	----
	Hypsiboas lundii	----	Bokermann & Sazima, 1978	----
	Hypsiboas marginatus	----	Garcia *et al.*, 2001	----
	Hypsiboas marianitae	----	Duellman *et al.*, 1997	----
	Hypsiboas melanopleura	----	Duellman *et al.*, 1997	----
	Hypsiboas microderma	----	Pyburn, 1977	----

Hylidae (cont.)	*Hypsiboas multifasciatus*	----	de Sá, 1996	----
	Hypsiboas nympha	----	Faivovich *et al.*, 2006	----
	Hypsiboas ornatissimus	Kok & Kalamandeen, 2008	Kok & Kalamandeen, 2008	----
	Hypsiboas pardalis	----	Lutz, 1973	----
	Hypsiboas pellucens	----	Duellman, 1971	----
	Hypsiboas picturatus	MOP pers. obs.	MOP pers. obs.	----
	Hypsiboas polytaenius	Lutz, 1973	Boulenger, 1882; Lutz, 1973	----
	Hypsiboas prasinus	----	Lutz, 1973	----
	Hypsiboas pulchellus	DB pers. obs.; Lutz, 1973	DB pers. obs.; Boulenger, 1882; Lutz, 1973	----
	Hypsiboas punctatus	Duellman, 2005	Duellman, 2005	----
	Hypsiboas raniceps	Lutz, 1973	Lutz, 1973	----
	Hypsiboas riojanus	Lutz, 1973	Lutz, 1973	----
	Hypsiboas roraima	----	Duellman & Hoogmoed, 1992	----
	Hypsiboas rosenbergi	----	----	Duellman, 1970b
	Hypsiboas rufitelus	----	----	Duellman, 1970b
	Hypsiboas semiguttatus	García *et al.*, 2007	García *et al.*, 2007	----
	Hypsiboas semilineatus	----	Lutz, 1973	----
	Hypsiboas sibleszi	----	Hoogmoed, 1979	----
	Isthmohyla pseudopuma	Savage, 2002	Savage, 2002	----
	Isthmohyla rivularis	Savage, 2002	Savage, 2002	----
	Isthmohyla tica	----	Boulenger, 1882	Duellman, 1970b
	Itapothyla langsdorfii	Lutz, 1973	Boulenger, 1882; Lutz, 1973	----
	Litoria adelaidensis	Barker *et al.*, 1995	Boulenger, 1882; Barker *et al.*, 1995	----
	Litoria alboguttata	----	Anstis, 2013	----
	Litoria amboinensis	----	Menzies, 2006	----
	Litoria andiirrmalin	----	Anstis, 2013	----
	Litoria angiana	----	----	Tyler & Davies, 1979

Hylidae (cont.)	*Litoria arfakiana*	----	Boulenger, 1882	----
	Litoria aurea	Barker *et al.*, 1995	Barker *et al.*, 1995	----
	Litoria australis	Barker *et al.*, 1995	Barker *et al.*, 1995	Lynch, 1971
	Litoria barringtonensis	----	Anstis, 2013	----
	Litoria bicolor	Barker *et al.*, 1995	Boulenger, 1882; Barker *et al.*, 1995	----
	Litoria booroolongensis	----	Anstis, 2013	----
	Litoria brevipes	Barker *et al.*, 1995	Boulenger, 1882; Barker *et al.*, 1995	Boulenger, 1882
	Litoria burrowsi	----	Anstis, 2013	----
	Litoria caerulea	Barker *et al.*, 1995	Boulenger, 1882; Barker *et al.*, 1995	Tyler & Davies, 1978
	Litoria cavernicola	Barker *et al.*, 1995	Barker *et al.*, 1995	----
	Litoria chloris	----	Anstis, 2013	----
	Litoria citropa	Barker *et al.*, 1995	Barker *et al.*, 1995; Anstis, 2013	----
	Litoria congenita	----	Menzies, 2006	----
	Litoria coplandi	----	Anstis, 2013	----
	Litoria cryptotis	----	Anstis, 2013	----
	Litoria cultripes	Barker *et al.*, 1995	Barker *et al.*, 1995	Lynch, 1971
	Litoria cyclorhyncha	----	Anstis, 2013	----
	Litoria dahlii	Barker *et al.*, 1995	Barker *et al.*, 1995	Lynch, 1971
	Litoria darlingtoni	----	Menzies, 2006	----
	Litoria daviesae	----	Anstis, 2013	----
	Litoria dayi	Barker *et al.*, 1995	Barker *et al.*, 1995; Anstis, 2013	----
	Litoria dentata	Barker *et al.*, 1995	Barker *et al.*, 1995	----
	Litoria electrica	Barker *et al.*, 1995	Barker *et al.*, 1995	----
	Litoria eucnemis	----	Anstis, 2013	----
	Litoria ewingii	Barker *et al.*, 1995	Boulenger, 1882	Parker, 1881

Hylidae (cont.)	*Litoria fallax*	Barker *et al.*, 1995	Barker *et al.*, 1995	----
	Litoria freycineti	Barker *et al.*, 1995	Barker *et al.*, 1995	----
	Litoria genimaculata	Barker *et al.*, 1995	Barker *et al.*, 1995	----
	Litoria gilleni	----	Anstis, 2013	----
	Litoria gracilenta	----	Boulenger, 1882	----
	Litoria havina	----	Menzies, 2006	----
	Litoria impura	----	Menzies, 2006	----
	Litoria inermis	----	Davies *et al.*, 1983	----
	Litoria jervisiensis	----	Anstis, 2013	----
	Litoria jungguy	----	Anstis, 2013	----
	Litoria kumae	----	Menzies, 2006	----
	Litoria latopalmata	----	----	Tyler & Davies, 1978
	Litoria lesueurii	Barker *et al.*, 1995	----	Tyler & Davies, 1978
	Litoria longipes	----	Anstis, 2013	----
	Litoria longirostris	Barker *et al.*, 1995	Barker *et al.*, 1995	----
	Litoria maculosa	Barker *et al.*, 1995	Barker *et al.*, 1995	----
	Litoria maini	----	Anstis, 2013	----
	Litoria manya	----	Anstis, 2013	----
	Litoria meiriana	----	Anstis, 2013	----
	Litoria microbelos	Barker *et al.*, 1995	Barker *et al.*, 1995	----
	Litoria micromembrana	----	----	Tyler & Davies, 1979
	Litoria modica	----	----	Tyler & Davies, 1978
	Litoria moorei	Barker *et al.*, 1995	Barker *et al.*, 1995	Tyler & Davies, 1978
	Litoria nannotis	Barker *et al.*, 1995	----	----
	Litoria nasuta	----	Anstis, 2013	----
	Litoria nigrofrenata	Barker *et al.*, 1995	Boulenger, 1882	----
	Litoria novaehollandiae	----	Anstis, 2013	----
	Litoria nudidigita	----	Anstis, 2013	----

Hylidae (cont.)	*Litoria nyakalensis*	Barker *et al.*, 1995	Barker *et al.*, 1995	----
	Litoria pallida	----	Davies *et al.*, 1983	----
	Litoria paraewingi	----	Anstis, 2013	----
	Litoria pearsoniana	Barker *et al.*, 1995	Barker *et al.*, 1995	----
	Litoria peronii	Barker *et al.*, 1995	Boulenger, 1882	----
	Litoria personata	----	Anstis, 2013	----
	Litoria phyllochroa	Barker *et al.*, 1995	Boulenger, 1882; Barker *et al.*, 1995	Parker, 1881
	Litoria platycephala	Barker *et al.*, 1995	Barker *et al.*, 1995	Lynch, 1971
	Litoria pronimia	----	Menzies, 2006	----
	Litoria raniformis	----	Anstis, 2013	----
	Litoria revelata	----	Anstis, 2013	----
	Litoria rheocola	----	Anstis, 2013	----
	Litoria rothi	Barker *et al.*, 1995	Barker *et al.*, 1995	----
	Litoria rubella	----	Anstis, 2013	----
	Litoria splendida	----	Anstis, 2013	----
	Litoria subglandulosa	Barker *et al.*, 1995	Barker *et al.*, 1995	----
	Litoria tornieri	----	Davies *et al.*, 1983	----
	Litoria tyleri	----	Anstis, 2013	----
	Litoria vagitus	----	Anstis, 2013	----
	Litoria verreauxii	Barker *et al.*, 1995	----	Tyler & Davies, 1978
	Litoria verrucosa	----	Anstis, 2013	----
	Litoria watjulumensis	----	Anstis, 2013	----
	Litoria wilcoxii	----	Anstis, 2013	----
	Litoria xanthomera	Barker *et al.*, 1995	Barker *et al.*, 1995; Anstis, 2013	----
	Lysapsus limellum	MOP pers. obs.	MOP pers. obs.; Boulenger, 1882	----
	Megastomatohyla mixe	----	Duellman, 1970b	----

Hylidae (cont.)	*Myersiohyla imparquesi*	Ayarzagüena & Señaris, 1993	Ayarzagüena & Señaris, 1993	----
	Myersiohyla kanaima	----	Duellman & Hoogmoed, 1992	----
	Nyctimantis rugiceps	----	Duellman & Trueb, 1976	----
	Nyctimystes brevipalmatus	Barker *et al.*, 1995	Barker *et al.*, 1995	----
	Nyctimystes foricula	----	----	Tyler & Davies, 1979
	Nyctimystes infrafrenatus	Barker *et al.*, 1995	Boulenger, 1882; Barker *et al.*, 1995	Tyler & Davies, 1978
	Nyctimystes kubori	----	----	Tyler & Davies, 1979
	Nyctimystes narinosus	----	----	Tyler & Davies, 1979
	Nyctimystes papua	----	----	Tyler & Davies, 1979
	Nyctimystes pulcher	----	----	Tyler & Davies, 1979
	Nyctymistes zweifeli	----	----	Tyler & Davies, 1979
	Osteocephalus alboguttatus	----	Boulenger, 1882	----
	Osteocephalus buckleyi	Jungfer *et al.*, 2013; Lima *et al.*, 2006	Jungfer *et al.*, 2013; Lima *et al.*, 2006	----
	Osteocephalus cabrerai	----	Jungfer, 2010	----
	Osteocephalus deridens	----	Jungfer *et al.*, 2000	----
	Osteocephalus fuscifascies	----	Jungfer *et al.*, 2000	----
	Osteocephalus leprieurii	Kok & Kalamandeen, 2008	Boulenger, 1882; Kok & Kalamandeen, 2008	Trueb & Duellman, 1971
	Osteocephalus mutabor	Kok & Kalamandeen, 2008	Kok & Kalamandeen, 2008	----
	Osteocephalus planiceps	----	Jungfer *et al.*, 2000	----
	Osteocephalus oophagus	Jungfer *et al.*, 2013; Lima *et al.*, 2006	Jungfer *et al.*, 2013; Lima *et al.*, 2006	----
	Osteocephalus taurinus	----	----	Trueb, 1970b
	Osteocephalus verruciger	----	Trueb & Duellman, 1971	----
	Osteocephalus yasuni	----	Jungfer *et al.*, 2013	----
	Osteopilus brunneus	----	Trueb & Tyler, 1974	----
	Osteopilus crucialis	----	Boulenger, 1882	----
	Osteopilus dominicensis	----	Trueb & Tyler, 1974	----

Hylidae (cont.)	*Osteopilus marianae*	----	Trueb & Tyler, 1974	----
	Osteopilus pulchrilineatus	----	Trueb & Tyler, 1974	----
	Osteopilus septentrionalis	Wever, 1985; Diaz & Cadiz, 2008	Diaz & Cadiz, 2008	Wever, 1985
	Osteopilus vastus	----	Boulenger, 1882	----
	Osteopilus wilderi	----	Trueb & Tyler, 1974	----
	Phasmahyla cochranae	----	Bokermann, 1966	----
	Phasmahyla cruzi	----	Carvalho-e-Silva *et al.*, 2009	----
	Phasmahyla exilis	----	Cruz, 1980	----
	Phasmahyla guttata	----	----	Cruz, 1990
	Phasmahyla jandaia	----	Bokermann & Sazima, 1978	----
	Phrynomedusa marginata	----	Izecksohn & Cruz, 1976	----
	Phyllodytes auratus	----	Kenny, 1969	----
	Phyllodytes luteolus	----	----	KAV pers. comm.
	Phyllomedusa atelopoides	----	Duellman, 2005	Sheil & Alamillo, 2005
	Phyllomedusa ayeaye	----	Lutz, 1966	----
	Phyllomedusa azurea	MOP pers. obs.	MOP pers. obs.	MOP pers. obs.
	Phyllomedusa bahiana	----	Lutz & Lutz, 1939	----
	Phyllomedusa baltea	----	Duellman & Toft, 1979	----
	Phyllomedusa bicolor	Lima *et al.*, 2006; Kok & Kalamandeen, 2008	Boulenger, 1882; Lima *et al.*, 2006; Kok & Kalamandeen, 2008	Parker, 1881
	Phyllomedusa boliviana	----	Cei, 1980	----
	Phyllomedusa burmeisteri	----	Izecksohn & Carvalho-e-Silva, 2001	----
	Phyllomedusa camba	Duellman, 2005	Duellman, 2005	----
	Phyllomedusa centralis	----	Bokermann, 1965	----
	Phyllomedusa duellmani	----	Cannatella, 1982	----
	Phyllomedusa hypochondrialis	----	Caramaschi, 2006	----

Hylidae (cont.)	*Phyllomedusa iheringii*	----	Langone, 1994	----
	Phyllomedusa megacephala	----	Caramaschi, 2006	----
	Phyllomedusa neildi	----	Barrio-Amorós, 2006	----
	Phyllomedusa nordestina	----	Caramaschi, 2006	----
	Phyllomedusa oreades	----	Brandão, 2002	----
	Phyllomedusa palliata	Boulenger, 1882	Boulenger, 1882	----
	Phyllomedusa perinesos	----	Duellman, 1973	----
	Phyllomedusa rohdei	----	Izecksohn & Carvalho-e-Silva, 2001	----
	Phyllomedusa sauvagii	MOP pers. obs.	MOP pers. obs.	MOP pers. obs.
	Phyllomedusa tarsius	Lima *et al.*, 2006	Boulenger, 1882; Lima *et al.*, 2006	----
	Phyllomedusa tetraploidea	----	Pombal & Haddad, 1992	----
	Phyllomedusa tomopterna	Lima *et al.*, 2006	Lima *et al.*, 2006	----
	Phyllomedusa trinitatis	----	Funkhouser, 1957	----
	Phyllomedusa vaillanti	----	Funkhouser, 1957	----
	Plectrohyla ameibothalame	----	Canseco-Márquez *et al.*, 2002	----
	Plectrohyla arborescandens	----	Duellman, 2001	----
	Plectrohyla bistincta	----	Boulenger, 1882	----
	Plectrohyla calthula	----	Duellman, 2001	----
	Plectrohyla chrysopleura	----	Duellman, 2001	----
	Plectrohyla cyclada	----	Duellman, 2001	----
	Plectrohyla glandulosa	----	Duellman, 1970b	----
	Plectrohyla guatemalensis	----	Boulenger, 1882	----
	Plectrohyla mutadai	----	Duellman, 1970b	----
	Plectrohyla pentheter	----	Duellman, 1970b	----
	Plectrohyla siopela	----	Duellman, 1970b	Duellman, 1970b
	Pseudacris brachypona	Dood, 2013	Dood, 2013	----
	Pseudacris brimleyi	Dood, 2013	Dood, 2013	----

Hylidae (cont.)	*Pseudacris cadaverina*	----	Duellman, 1970b	----
	Pseudacris clarkii	----	----	Duellman, 1970b
	Pseudacris crucifer	Dood, 2013	Dood, 2013	----
	Pseudacris feriarum	Dood, 2013	Dood, 2013	----
	Pseudacris fouquetti	----	Elliot *et al.*, 2009	----
	Pseudacris illinoensis	Dood, 2013	Dood, 2013	----
	Pseudacris kalmi	----	Elliot *et al.*, 2009	----
	Pseudacris maculata	----	Elliot *et al.*, 2009	----
	Pseudacris nigrita	----	Boulenger, 1882	----
	Pseudacris ocularis	----	Boulenger, 1882	Gaudin, 1974
	Pseudacris ornata	----	Boulenger, 1882	Gaudin, 1974
	Pseudacris regilla	----	Boulenger, 1882	Gaudin, 1974
	Pseudacris streckeri	Wever, 1985	----	Wever, 1985
	Pseudacris triseriata	----	Boulenger, 1882	----
	Pseudis bolbodactyla	----	Caramaschi & Cruz, 1998	----
	Pseudis cardosoi	----	Kwet, 2000	----
	Pseudis fusca	----	Caramaschi & Cruz, 1998	----
	Pseudis minuta	MOP pers. obs.	MOP pers. obs.; Boulenger, 1882	Parker, 1881
	Pseudis paradoxa	Duellman, 2005	Boulenger, 1882; Duellman, 2005	Parker, 1881
	Pseudis tocantins	----	Caramaschi & Cruz, 1998	----
	Ptychohyla dendrophasma	----	Duellman, 2001	----
	Ptychohyla euthysanota	----	Duellman, 1970b	----
	Ptychohyla hypomykter	----	McCranie & Wilson, 1993	----
	Ptychohyla leonhardschultzei	----	Duellman, 1970b	----
	Ptychohyla spinipollex	----	----	Duellman, 1970b
	Ptychohyla zophodes	----	Duellman, 2001	----
	Scarthyla goinorum	Duellman, 2005	Duellman, 2005	KAV pers. comm.

Hylidae (cont.)	*Scinax acuminatus*	DB pers. obs.	DB pers. obs.; Boulenger, 1882	----
	Scinax berthae	Barrio, 1962	Barrio, 1962	----
	Scinax boesemani	Lima *et al.*, 2006; Kok & Kalamandeen, 2008	Lima *et al.*, 2006; Kok & Kalamandeen, 2008	----
	Scinax boulengeri	Wever, 1985	----	Wever, 1985
	Scinax catharinae	----	JF pers. obs.	JF pers. obs.
	Scinax crospedospilus	----	Heyer *et al.*, 1990	----
	Scinax cruentommus	----	JF pers. obs.	JF pers. obs.
	Scinax elaeochrous	----	Boulenger, 1882	Duellman, 1970b
	Scinax faivovichi	Brasileiro *et al.*, 2007	Brasileiro *et al.*, 2007	----
	Scinax fuscovarius	DB pers. obs.; Lutz, 1973	DB pers. obs.; Lutz, 1973	----
	Scinax garbei	Lima *et al.*, 2006	JF pers. obs.; Lima *et al.*, 2006	JF pers. obs.
	Scinax nasicus	----	Boulenger, 1882	----
	Scinax nebulosus	----	JF pers. obs.	JF pers. obs.
	Scinax perpusillus	Lutz, 1973	JF pers. obs.; Lutz, 1973	JF pers. obs.
	Scinax rostratus	----	Boulenger, 1882	Duellman, 1970b
	Scinax ruber	----	JF pers. obs.	JF pers. obs.
	Scinax squalirostris	----	JF pers. obs.	JF pers. obs.
	Scinax staufferi	----	Boulenger, 1882	----
	Scinax uruguayus	Giraudo *et al.*, 2005	Giraudo *et al.*, 2005	----
	Smilisca baudinii	Savage, 2002	Boulenger, 1882; Savage, 2002	Trueb, 1970a
	Smilisca cyanosticta	----	Duellman, 1970b	----
	Smilisca fodiens	----	----	Trueb, 1970a
	Smilisca phaeota	Wever, 1985; Savage, 2002	Savage, 2002	Wever, 1985
	Smilisca puma	----	Duellman, 1970b	----
	Smilisca sila	----	Duellman, 1970b	----
	Smilisca sordida	----	Boulenger, 1882	Duellman, 1970b

Hylidae (cont.)	*Sphaenorhynchus lacteus*	Duellman, 2005	Duellman, 2005	KAV pers. comm.
	Tepuhyla talbergae	Kok & Kalamandeen, 2008	Kok & Kalamandeen, 2008	----
	Tepuihyla aecii	----	Ayarzagüena *et al.*, 1992	----
	Tepuihyla exophthalma	Smith & Noonan (2001)	Smith & Noonan (2001)	Smith & Noonan (2001)
	Tepuihyla edelcae	----	Ayarzagüena *et al.*, 1992	----
	Tepuihyla rodriguezi	----	Ayarzagüena *et al.*, 1992	----
	Tlalocohyla godmani	----	Duellman, 1970b	----
	Tlalocohyla loquax	Savage, 2002	Savage, 2002	Duellman, 1970b
	Tlalocohyla picta	----	Duellman, 1970b	----
	Tlalocohyla smithtii	----	Duellman, 1970b	----
	Trachycephalus hadroceps	----	Duellman & Hoogmoed, 1992	----
	Trachycephalus jordani	----	----	Trueb, 1970b
	Trachycephalus mesophaeus	Boulenger, 1882	Boulenger, 1882	----
	Trachycephalus nigromaculatus	----	----	Trueb, 1970b
	Trachycephalus resinifictrix	Lima *et al.*, 2006	Lima *et al.*, 2006	----
	Trachycephalus typhonius	Lutz, 1973	Lutz, 1973	Trueb, 1970b, Duellman, 1970b
	Triprion petasatus	----	Duellman, 1970b	Duellman, 1970b
	Xenohyla truncata	Lutz, 1973	Lutz, 1973	KAV pers. comm.
Hylodidae	*Crossodactylus schmidti*	MOP pers. obs.	MOP pers. obs.	MOP pers. obs.
	Hylodes ornatus	----	Bokermann, 1967c	----
	Megaelosia goeldii	----	----	Lynch, 1971
Hyperoliidae	*Acanthixalus spinosus*	Drewes, 1984	Amiet, 2012	----
	Afrixalus dorsalis	----	Drewes, 1984	----
	Afrixalus fornasini	du Preez & Carruthers, 2009	du Preez & Carruthers, 2009	Laurent, 1944
	Afrixalus laevis	----	----	Laurent, 1944
	Alexteroon obstetricans	----	Amiet, 2012	----

Hyperoliidae (cont.)	*Cryptothylax gresshoffi*	----	Drewes, 1984	Laurent & Combaz, 1950; Liem, 1970
	Heterixalus alboguttatus	Glaw & Vences, 2007	Glaw & Vences, 2007	----
	Heterixalus betsileo	Glaw & Vences, 2007	----	Laurent, 1944
	Heterixalus boettgeri	Glaw & Vences, 2007	Glaw & Vences, 2007	----
	Heterixalus carbonei	Vences *et al.*, 2000	Vences *et al.*, 2000	----
	Heterixalus madagascariensis	Glaw & Vences, 2007	Vences *et al.*, 2002; Glaw & Vences, 2007	Laurent, 1944
	Heterixalus punctatus	----	Vences *et al.*, 2002	----
	Heterixalus tricolor	----	Glaw & Vences, 2007	----
	Hyperolius benguellensis	du Preez & Carruthers, 2009	----	Laurent, 1944
	Hyperolius castaneus	----	Laurent, 1944	Laurent, 1944
	Hyperolius chlorosteus	----	Boulenger, 1915	----
	Hyperolius concolor	----	Amiet, 2012	----
	Hyperolius guttulatus	----	Boulenger, 1882	----
	Hyperolius nasutus	Drewes, 1984; du Preez & Carruthers, 2009; Harper *et al.*, 2010	Harper *et al.*, 2010	Laurent, 1944
	Hyperolius ocellatus	----	Laurent, 1944	Laurent, 1944
	Hyperolius phantasticus	----	Drewes, 1984	Laurent, 1944
	Hyperolius puncticulatus	----	Schiotz, 1975	----
	Hyperolius pusillus	Drewes, 1984; du Preez & Carruthers, 2009	----	Laurent, 1944
	Hyperolius tuberilinguis	du Preez & Carruthers, 2009	du Preez & Carruthers, 2009	----
	Hyperolius viridiflavus	Wever, 1985	Wever, 1985	Wever, 1985; Laurent, 1944
	Kassina maculata	du Preez & Carruthers, 2009	Drewes, 1984; du Preez & Carruthers, 2009	----
	Kassina senegalensis	Scott, 2005; du Preez & Carruthers, 2009	Scott, 2005; Boulenger, 1882; Drewes, 1984; du Preez & Carruthers, 2009	Scott, 2005

Hyperoliidae (cont.)	*Morerella cyanophthalma*	Rödel *et al.*, 2009	Rödel *et al.*, 2009	Rödel *et al.*, 2009
	Opisthotylax immaculatus	Drewes, 1984	----	Drewes, 1984
	Phlyctimantis leonardi	----	Drewes, 1984	----
	Phlyctimantis verrucosus	Spawls *et al.*, 2006	Spawls *et al.*, 2006	Laurent, 1941b
	Semnodactylus wealii	du Preez & Carruthers, 2009	Drewes, 1984; Boulenger, 1882	----
	Tachycnemis seychellensis	----	Drewes, 1984; Boulenger, 1882	----
Leiopelmatidae	*Leiopelma archeyi*	----	----	Stephenson, 1951
	Leiopelma hochstetteri	Wever, 1985	Wever, 1985	Wever, 1985
Leptodactylidae	*Adenomera andreae*	Lima *et al.*, 2006	Lima *et al.*, 2006	----
	Adenomera heyeri	Boistel *et al.*, 2006	Boistel *et al.*, 2006	Boistel *et al.*, 2006
	Adenomera hylaedactyla	Lima *et al.*, 2006	Lima *et al.*, 2006	Lynch, 1971
	Edalorhina perezi	Beirne & Witworth, 2011	Boulenger, 1882; Beirne & Witworth, 2011	Lynch, 1971
	Engystomops coloradum	Nacimento *et al.*, 2005	----	----
	Engystomops guayaco	Nacimento *et al.*, 2005	----	----
	Engystomops montubio	Nacimento *et al.*, 2005	----	----
	Engystomops petersi	Nacimento *et al.*, 2005	DB pers. obs.	Lynch, 1971
	Engystomops pustulatus	DB pers. obs.	DB pers. obs.	Lynch, 1971
	Engystomops pustulosus	Nacimento *et al.*, 2005	----	Lynch, 1971
	Engystomops randi	Nacimento *et al.*, 2005	----	----
	Leptodactylus albilabris	----	Ponssa, 2008.	Lynch, 1971; Ponssa, 2008
	Leptodactylus bolivianus	----	----	Lynch, 1971
	Leptodactylus bufonius	DB pers. obs.	DB pers. obs.; Ponssa, 2008	Lynch, 1971; Ponssa, 2008
	Leptodactylus chaquensis	DB pers. obs.	DB pers. obs.	Lynch, 1971
	Leptodactylus diedrus	de Sá *et al.*, 2014	de Sá *et al.*, 2014	----
	Leptodactylus discodactylus	de Sá *et al.*, 2014	de Sá *et al.*, 2014	----
	Leptodactylus didymus	de Sá *et al.*, 2014	de Sá *et al.*, 2014	----

Leptodactylidae (cont.)	*Leptodactylus elenae*	de Sá *et al.*, 2014	de Sá *et al.*, 2014	----
	Leptodactylus fallax	de Sá *et al.*, 2014	de Sá *et al.*, 2014	----
	Leptodactylus fuscus	Lima *et al.*, 2006	Ponssa, 2008; Lima *et al.*, 2006	Parker, 1881; Ponssa, 2008
	Leptodactylus gracilis	Boulenger, 1882	Boulenger, 1882; Ponssa, 2008	Lynch, 1971; Ponssa, 2008
	Leptodactylus griseigularis	de Sá *et al.*, 2014	de Sá *et al.*, 2014	----
	Leptodactylus knudseni	Lima *et al.*, 2006	Lima *et al.*, 2006	----
	Leptodactylus labyrinthicus	de Sá *et al.*, 2014	de Sá *et al.*, 2014	----
	Leptodactylus latrans	DB pers. obs.	DB pers. obs.	Parker, 1881
	Leptodactylus leptodactyloides	de Sá *et al.*, 2014	de Sá *et al.*, 2014	----
	Leptodactylus longirostris	de Sá *et al.*, 2014	de Sá *et al.*, 2014	----
	Leptodactylus melanonotus	de Sá *et al.*, 2014	de Sá *et al.*, 2014	Lynch, 1971
	Leptodactylus myersi	de Sá *et al.*, 2014	de Sá *et al.*, 2014	----
	Leptodactylus mystaceus	Lima *et al.*, 2006	Ponssa, 2008; Lima *et al.*, 2006	Lynch, 1971; Ponssa, 2008
	Leptodactylus mystacinus	MOP pers. obs.	MOP pers. obs.; Ponssa, 2008	Lynch, 1971; Ponssa, 2008
	Leptodactylus notoaktites	de Sá *et al.*, 2014	de Sá *et al.*, 2014	----
	Leptodactylus pentadactylus	Lima *et al.*, 2006	Boulenger, 1882; Lima *et al.*, 2006	Lynch, 1971
	Leptodactylus plaumanni	de Sá *et al.*, 2014	de Sá *et al.*, 2014	----
	Leptodactylus podicipinus	DB pers. obs.	DB pers. obs.	Lynch, 1971
	Leptodactylus rhodonotus	Duellman, 2005	Duellman, 2005	----
	Leptodactylus rhodomystax	de Sá *et al.*, 2014	de Sá *et al.*, 2014	----
	Leptodactylus riveroi	Lima *et al.*, 2006	Lima *et al.*, 2006	----
	Leptodactylus silvanimbus	de Sá *et al.*, 2014	de Sá *et al.*, 2014	----
	Leptodactylus spixi	de Sá *et al.*, 2014	de Sá *et al.*, 2014	----
	Leptodactylus stenodema	Lima *et al.*, 2006	Lima *et al.*, 2006	----
	Leptodactylus syphax	DB pers. obs.	DB pers. obs.	Lynch, 1971
	Leptodactylus validus	de Sá *et al.*, 2014	de Sá *et al.*, 2014	----

Leptodactylidae (cont.)	*Leptodactylus vastus*	de Sá *et al.*, 2014	de Sá *et al.*, 2014	----
	Leptodactylus wagneri	de Sá *et al.*, 2014	de Sá *et al.*, 2014	Lynch, 1971
	Lithodytes lineatus	Lima *et al.*, 2006	Lima *et al.*, 2006	Lynch, 1971
	Paratelmatobius cardosoi	----	----	Fouquet *et al.*, 2013
	Paratelmatobius poecilogaster	----	----	BLB pers. obs.; Fouquet *et al.*, 2013
	Physalaemus albonotatus	DB pers. obs.	DB pers. obs.	Lynch, 1971
	Physalaemus biligonigerus	Boulenger, 1882	DB pers. obs.	DB pers. obs.; Lynch, 1971
	Physalaemus centralis	DB pers. obs.	DB pers. obs.	Lynch, 1971
	Physalaemus cuvieri	DB pers. obs.	DB pers. obs.	Lynch, 1971
	Physalaemus gracilis	----	Boulenger, 1882	Lynch, 1971
	Physalaemus nattereri	DB pers. obs.	DB pers. obs.	Lynch, 1971
	Physalaemus signifer	----	----	Lynch, 1971
	Pleurodema brachyops	Wever, 1985	Wever, 1985	Lynch, 1971; Wever, 1985
	Pleurodema cinereum	DB pers. obs.	DB pers. obs.; Boulenger, 1882	Lynch, 1971
	Pleurodema diplolister	----	Boulenger, 1882	Lynch, 1971
	Pleurodema kriegi	----	MOP pers. obs.	MOP pers. obs.
	Pleurodema marmoratum	DB pers. obs.	DB pers. obs.	----
	Pleurodema tucumanum	----	MOP pers. obs.	MOP pers. obs.
	Pseudopaludicola falcipes	DB pers. obs.; Boulenger, 1882	DB pers. obs.	Lynch, 1971
	Pseudopaludicola mystacalis	DB pers. obs.	DB pers. obs.	Lynch, 1971
	Scythrophrys sawayae	Lynch, 1971		Verdade, 2005
Limnodynastidae	*Adelotus brevis*	Boulenger, 1882	----	Lynch, 1971
	Heleioporus australiacus	Barker *et al.*, 1995	Barker *et al.*, 1995	Lynch, 1971
	Lechriodus fletcheri	Barker *et al.*, 1995	Barker *et al.*, 1995	Lynch, 1971
	Lechriodus melanopyga	Boulenger, 1882	Boulenger, 1882	----
	Limnodynastes depressus	Barker *et al.*, 1995	Barker *et al.*, 1995	----
	Limnodynastes dorsalis	Barker *et al.*, 1995	----	Lynch, 1971

Limnodynastidae (cont.)	*Limnodynastes dumerilii*	Barker *et al.*, 1995	Barker *et al.*, 1995	----
	Limnodynastes fletcheri	Barker *et al.*, 1995	----	Lynch, 1971
	Limnodynastes lignarius	Barker *et al.*, 1995	Barker *et al.*, 1995	----
	Limnodynastes peronii	Barker *et al.*, 1995	Boulenger, 1882	Lynch, 1971
	Limnodynastes salmini	Barker *et al.*, 1995	Boulenger, 1882; Barker *et al.*, 1995	Boulenger, 1882
	Limnodynastes tasmaniensis	Boulenger, 1882; Barker *et al.*, 1995	----	Lynch, 1971; Parker, 1881
	Neobatrachus pelobatoides	Anstis, 2013	Anstis, 2013	----
	Neobatrachus pictus	Barker *et al.*, 1995	----	Lynch, 1971
	Neobatrachus sudelli	Barker *et al.*, 1995	----	Lynch, 1971
	Notaden bennettii	Barker *et al.*, 1995	----	Lynch, 1971
	Philoria sphagnicolus	Barker *et al.*, 1995	----	Lynch, 1971
	Platyplectrum ornatum	Barker *et al.*, 1995	Boulenger, 1882; Barker *et al.*, 1995	Boulenger, 1882
Mantellidae	*Aglyptodactylus madagascariensis*	Glaw & Vences, 2007	Boulenger, 1882; Glaw & Vences, 2007	----
	Blommersia blommersae	Glaw & Vences, 2007	Glaw & Vences, 2007	----
	Blommersia domerguei	Glaw & Vences, 2007	Glaw & Vences, 2007	----
	Blommersia grandisonae	Glaw & Vences, 2007	Glaw & Vences, 2007	----
	Blommersia madinika	Glaw & Vences, 2007	Glaw & Vences, 2007	----
	Blommersia sarotra	Glaw & Vences, 2007	Glaw & Vences, 2007	----
	Boehmantis microtympanum	Glaw & Vences, 2007	Glaw & Vences, 2007	----
	Boophis ankaratra	Glaw & Vences, 2007	Glaw & Vences, 2007	----
	Boophis axelmeyeri	Glaw & Vences, 2007	Glaw & Vences, 2007	----
	Boophis bottae	Glaw & Vences, 2007	Glaw & Vences, 2007	----
	Boophis englaenderi	Glaw & Vences, 2007	Glaw & Vences, 2007	----
	Boophis goudotii	Boulenger, 1882	Boulenger, 1882	Laurent, 1943
	Boophis idae	Glaw & Vences, 2007	Glaw & Vences, 2007	----
	Boophis madagascariensis	----	Boulenger, 1882	----

Mantellidae (cont.)	*Boophis mandraka*	Glaw & Vences, 2007	Glaw & Vences, 2007	----
	Boophis marojezensis	Glaw & Vences, 2007	Glaw & Vences, 2007	----
	Boophis pauliani	Glaw & Vences, 2007	Glaw & Vences, 2007	----
	Boophis phyrrus	Glaw & Vences, 2007	Glaw & Vences, 2007	----
	Boophis picturatus	Glaw & Vences, 2007	Glaw & Vences, 2007	----
	Boophis rufioculis	Glaw & Vences, 2007	Glaw & Vences, 2007	----
	Boophis sambirano	----	Vences & Glaw, 2005	----
	Boophis sibilans	Glaw & Vences, 2007	Glaw & Vences, 2007	----
	Boophis tephraeomystax	Glaw & Vences, 2007	Glaw & Vences, 2007; Boulenger, 1882	----
	Boophis vittatus	Glaw & Vences, 2007	Glaw & Vences, 2007	----
	Boophis williamsi	Glaw & Vences, 2007	Guibe, 1974; Glaw & Vences, 2007	----
	Gephyromantis asper	Glaw & Vences, 2007	Glaw & Vences, 2007	----
	Gephyromantis azurrae	Glaw & Vences, 2007	Glaw & Vences, 2007	----
	Gephyromantis blanci	----	Glaw & Vences, 2007	----
	Gephyromantis boulengeri	Glaw & Vences, 2007	Glaw & Vences, 2007	----
	Gephyromantis cornutus	Glaw & Vences, 2007	Glaw & Vences, 2007	----
	Gephyromantis corvus	Glaw & Vences, 2007	Glaw & Vences, 2007	----
	Gephyromantis eiselti	Glaw & Vences, 2007	Glaw & Vences, 2007	----
	Gephyromantis granulatus	----	Boulenger, 1882	----
	Gephyromantis horridus	Glaw & Vences, 2007	Vences *et al.*, 2002	----
	Gephyromantis klemmeri	Glaw & Vences, 2007	Guibe, 1974; Glaw & Vences, 2007	----
	Gephyromantis luecocephalus	Glaw & Vences, 2007	Glaw & Vences, 2007	----
	Gephyromantis leucomaculatus	Glaw & Vences, 2007	Glaw & Vences, 2007	----
	Gephyromantis luteus	Glaw & Vences, 2007	Glaw & Vences, 2007	----
	Gephyromantis malagasius	Glaw & Vences, 2007	Glaw & Vences, 2007	----

Mantellidae (cont.)	*Gephyromantis plicifer*	Glaw & Vences, 2007	Glaw & Vences, 2007	----
	Gephyromantis pseudoasper	Glaw & Vences, 2007	Guibe, 1974; Glaw & Vences, 2007	----
	Gephyromantis redimitus	Glaw & Vences, 2007	Glaw & Vences, 2007	----
	Gephyromantis rivicola	Glaw & Vences, 2007	Glaw & Vences, 2007	----
	Gephyromantis salegy	Glaw & Vences, 2007	Glaw & Vences, 2007	----
	Gephyromantis sculpturatus	Glaw & Vences, 2007	Glaw & Vences, 2007	----
	Gephyromantis striatus	Glaw & Vences, 2007	Vences *et al*., 2002	----
	Gephyromantis ventrimaculatus	Vences *et al*., 2002; Glaw & Vences, 2007	Vences *et al*., 2002; Glaw & Vences, 2007	----
	Gephyromantis zavona	Glaw & Vences, 2007	Glaw & Vences, 2007	----
	Guibemantis albolineatus	Glaw & Vences, 2007	Glaw & Vences, 2007	----
	Guibemantis bicalcaratus	Glaw & Vences, 2007	Glaw & Vences, 2007	----
	Guibemantis liber	Glaw & Vences, 2007	Glaw & Vences, 2007	----
	Guibemantis pulcher	----	Boulenger, 1882	----
	Guibemantis punctatus	Glaw & Vences, 2007	Glaw & Vences, 2007	----
	Guibemantis tornieri	Glaw & Vences, 2007	Glaw & Vences, 2007	----
	Laliostoma labrosum	Glaw & Vences, 2007	Glaw & Vences, 2007	----
	Mantella aurantiaca	Scott, 2005	Vences *et al*., 2002	Scott, 2005
	Mantella baroni	Glaw & Vences, 2007	Glaw & Vences, 2007	----
	Mantella betsileo	----	Boulenger, 1882	----
	Mantella bernhardi	Glaw & Vences, 2007	Glaw & Vences, 2007	----
	Mantella crocea	----	Vences *et al*., 2002	----
	Mantella ebenaui	----	Boulenger, 1882	----
	Mantella expectata	Glaw & Vences, 2007	Glaw & Vences, 2007	----
	Mantella laevigata	----	Vences *et al*., 2002	----
	Mantella madagascariensis	----	Boulenger, 1882	----
	Mantella nigricans	----	Glaw & Vences, 2007	----
	Mantella viridis	Glaw & Vences, 2007	Glaw & Vences, 2007	----

Mantellidae (cont.)	*Mantidactylus aerumnalis*	Glaw & Vences, 2007	Glaw & Vences, 2007	----
	Mantidactylus ambreensus	Glaw & Vences, 2007	Glaw & Vences, 2007	----
	Mantidactylus argenteus	Glaw & Vences, 2007	Glaw & Vences, 2007	----
	Mantidactylus betsileanus	Glaw & Vences, 2007	Glaw & Vences, 2007	----
	Mantidactylus biporus	Glaw & Vences, 2007	Glaw & Vences, 2007	----
	Mantidactylus charlotteae	Glaw & Vences, 2007	Glaw & Vences, 2007	----
	Mantidactylus curtus	Glaw & Vences, 2007	Glaw & Vences, 2007	----
	Mantidactylus femoralis	Scott, 2005; Glaw & Vences, 2007	Glaw & Vences, 2007	Scott, 2005
	Mantidactylus lugubris	----	Boulenger, 1882	----
	Mantidactylus majori	Glaw & Vences, 2007	Glaw & Vences, 2007	----
	Mantidactylus mocquardi	Glaw & Vences, 2007	Glaw & Vences, 2007	----
	Mantidactylus opiparis	Glaw & Vences, 2007	Glaw & Vences, 2007	----
	Mantidactylus ulcerosus	Glaw & Vences, 2007	Glaw & Vences, 2007	----
	Spinomantis aglavei	----	Vences *et al.*, 2002	----
	Spinomantis guibei	----	Guibe, 1974	----
	Tsingymantis antitra	Glaw & Vences, 2007	Glaw & Vences, 2007	----
Megophryidae	*Brachytarsophrys feae*	Boulenger, 1887	----	----
	Leptobrachium banae	----	Lathrop *et al.*, 1998a	----
	Leptobrachium chapaense	Delorme *et al.*, 2006	Delorme *et al.*, 2006	----
	Leptobrachium echinatum	Delorme *et al.*, 2006	Delorme *et al.*, 2006	----
	Leptobrachium hasseltii	Delorme *et al.*, 2006	Delorme *et al.*, 2006; Cannatella & Trueb, 1988	Cannatella & Trueb, 1988
	Leptobrachium hendricksoni	Delorme *et al.*, 2006	Delorme *et al.*, 2006	----
	Leptobrachium mouhoti	----	Stuart *et al.*, 2006	----
	Leptobrachium montanum	Delorme *et al.*, 2006	Delorme *et al.*, 2006	----
	Leptobrachium nigrops	Delorme *et al.*, 2006	Delorme *et al.*, 2006	----
	Leptobrachium pullum	Delorme *et al.*, 2006	Delorme *et al.*, 2006	----
	Leptobrachium smithi	Delorme *et al.*, 2006	Delorme *et al.*, 2006	----

Megophryidae (cont.)	*Leptolalax arayai*	Lathrop *et al.*, 1998b	Lathrop *et al.*, 1998b	----
	Leptolalax bourreti	Ohler *et al.*, 2011	Ohler *et al.*, 2011	----
	Leptolalax oshanensis	----	Liu, 1950	-----
	Leptolalax pelodytoides	Lathrop *et al.*, 1998b	Lathrop *et al.*, 1998b	----
	Leptolalax pictus	Inger & Stuebing, 1997	Inger & Stuebing, 1997	----
	Leptolalax pluvialis	Ohler *et al.*, 2011	Ohler *et al.*, 2011	----
	Leptolalax ventripunctatus	Ohler *et al.*, 2011	Ohler *et al.*, 2011	----
	Megophrys baluensis	----	Wang *et al.*, 2012	----
	Megophrys longipes	----	Wang *et al.*, 2012	----
	Megophrys major	----	Wang *et al.*, 2012	----
	Megophrys minor	----	Wang *et al.*, 2012	----
	Megophrys nasuta	Wever, 1985	Wever, 1985; Delorme *et al.*, 2006	Wever, 1985
	Megophrys parva	----	Wang *et al.*, 2012; Boulenger, 1882	----
	Megophrys shapingensis	----	Wang *et al.*, 2012	----
	Megophrys spinata	----	Wang *et al.*, 2012	----
	Ophryophryne hansi	----	Stuart *et al.*, 2006	----
	Ophryophryne microstoma	----	Wang *et al.*, 2012; Delorme *et al.*, 2006	----
	Oreolalax chuanbeiensis	Wu *et al.*, 1993	----	Wu *et al.*, 1993
	Oreolalax jiandongensis	Wei *et al.*, 2009	----	----
	Oreolalax liangbeiensis	Wei *et al.*, 2009	Wei *et al.*, 2009	Wei *et al.*, 2009
	Oreolalax lichuanensis	Wei *et al.*, 2009	Wei *et al.*, 2009; Wu *et al.*, 1993	Wei *et al.*, 2009
	Oreolalax major	Wu *et al.*, 1993; Wei *et al.*, 2009	Wu *et al.*, 1993; Wei *et al.*, 2009	Wu *et al.*, 1993; Wei *et al.*, 2009
	Oreolalax multipunctatus	Wei *et al.*, 2009	Wei *et al.*, 2009	Wei *et al.*, 2009
	Oreolalax nanjiangensis	Wei *et al.*, 2009	Wei *et al.*, 2009	Wei *et al.*, 2009
	Oreolalax omeimontis	Wei *et al.*, 2009	Wei *et al.*, 2009	Wei *et al.*, 2009

Megophryidae (cont.)	*Oreolalax pingii*	Delorme *et al.*, 2006; Wei *et al.*, 2009	Delorme *et al.*, 2006; Wei *et al.*, 2009	Wei *et al.*, 2009
	Oreolalax popei	Delorme *et al.*, 2006; Wei *et al.*, 2009	Delorme *et al.*, 2006; Wei *et al.*, 2009	Wei *et al.*, 2009
	Oreolalax rhodostigmatus	Wu *et al.*, 1993; Delorme *et al.*, 2006; Wei *et al.*, 2009	Wu *et al.*, 1993; Delorme *et al.*, 2006; Wei *et al.*, 2009	Wu *et al.*, 1993; Wei *et al.*, 2009
	Oreolalax rugosus	Wu *et al.*, 1993; Wei *et al.*, 2009	Wu *et al.*, 1993; Wei *et al.*, 2009	Wu *et al.*, 1993; Wei *et al.*, 2009
	Oreolalax schmidti	Delorme *et al.*, 2006; Wei *et al.*, 2009	Delorme *et al.*, 2006; Wei *et al.*, 2009	Wei *et al.*, 2009
	Oreolalax xiangchengensis	Wei *et al.*, 2009	----	----
	Scutiger boulengeri	----	----	Fu *et al.*, 1997
	Scutiger chintingensis	----	----	Fu *et al.*, 1997
	Scutiger mammatus	----	Günther, 1896	Fu *et al.*, 1997
	Scutiger muliensis	----	----	Fu *et al.*, 1997
	Scutiger tuberculatus	----	----	Fu *et al.*, 1997
Micrixalidae	*Micrixalus fuscus*	----	Boulenger, 1882; Biju *et al.*, 2014a	Biju *et al.*, 2014a
	Micrixalus kottigeharensis	----	Rao, 1937; Biju *et al.*, 2014a	----
	Micrixalus saxicola	----	Biju *et al.*, 2014a; Chandramouli & Dutta, 2015	Chandramouli & Dutta, 2015
Microhylidae	*Anodonthyla boulengerii*	Glaw & Vences, 2007	Glaw & Vences, 2007; Parker, 1934	Parker, 1934
	Anodonthyla montana	Glaw & Vences, 2007	Glaw & Vences, 2007; Parker, 1934	Parker, 1934
	Anodonthyla moramora	Glaw & Vences, 2007	Glaw & Vences, 2007	----
	Anodonthyla rouxae	Glaw & Vences, 2007	Guibe, 1974; Glaw & Vences, 2007	----
	Apantophryne pansa	----	Parker, 1934	Parker, 1934
	Asterophrys turpicola	Zweifel, 1972	Zweifel, 1972	----
	Austrochaperina palmipes	Zweifel, 1956a	Zweifel, 1956a	Zweifel, 1956a

Microhylidae (cont.)	*Barygenys flavigularis*	Zweifel, 1972	Zweifel, 1972	----
	Calluela guttulata	Boulenger, 1882	Parker, 1934	Parker, 1934
	Calluela minuta	----	Parker, 1934	Parker, 1934
	Calluela yunnanensis	Zhao & Adler, 1993	Parker, 1934	Parker, 1934
	Callulops robustus	Zweifel, 1972	Parker, 1934; Zweifel, 1972	Parker, 1934
	Chaperina fusca	Inger, 1966	Inger, 1966; Parker, 1934	Parker, 1934
	Chiasmocleis albopunctata	----	Parker, 1934	Parker, 1934
	Chiasmocleis hudsoni	Lima *et al.*, 2006	Lima *et al.*, 2006	----
	Choerophryne rostellifer	----	Parker, 1934	Parker, 1934
	Cophixalus ornatus	----	----	Zweifel, 1985
	Cophyla berara	----	Glaw & Vences, 2007	----
	Cophyla phyllodactyla	Glaw & Vences, 2007	Boulenger, 1882; Glaw & Vences, 2007; Parker, 1934	Parker, 1934
	Copiula derongo	Zweifel, 2000	Zweifel, 2000	----
	Copiula guttata	----	----	Zweifel, 2000
	Ctenophryne aequatorialis	MT pers. obs.	MT pers. obs.	MT pers. obs.; Trueb *et al.*, 2011
	Ctenophryne geayi	MT pers. obs.; Parker, 1934	MT pers. obs.; Parker, 1934	MT pers. obs.; Parker, 1934
	Dasypops schirchi	MT pers. obs.	MT pers. obs.; Parker, 1934	MT pers. obs.; Parker, 1934
	Dermatonotus muelleri	DB & MT pers. obs.	MT pers. obs.	MT pers. obs.; Trueb *et al.*, 2011
	Dyscophus antongilii	Nussbaum & Wu, 2007	Boulenger, 1882; Nussbaum & Wu, 2007; Parker, 1934	Nussbaum & Wu, 2007; Parker, 1934
	Dyscophus guineti	MT pers. obs.; Glaw & Vences, 2007	MT pers. obs.; Glaw & Vences, 2007	MT pers. obs.; Parker, 1934
	Dyscophus insularis	Boulenger, 1882; Glaw & Vences, 2007	Glaw & Vences, 2007; Parker, 1934	Parker, 1934
	Elachistocleis bicolor	MT pers. obs.; Boulenger, 1882	MT pers. obs.	MOP & MT pers. obs.; Parker, 1934
	Gastrophryne carolinensis	MT pers. obs.; Wever, 1985	MT pers. obs.; Parker, 1934; Wever, 1985	MT pers. obs.; Parker, 1934; Wever, 1985

Microhylidae (cont.)	*Gastrophryne elegans*	----	Parker, 1934	Parker, 1934
	Genyophryne thomsoni	----	Parker, 1934	Parker, 1934
	Glyphoglossus molossus	Boulenger, 1882	Parker, 1934	Parker, 1934
	Hamptophryne boliviana	MT pers. obs.	MT pers. obs.; de Sa & Trueb, 1991	MT pers. obs.; Parker, 1934; de Sa & Trueb, 1991
	Hoplophryne rogersi	Parker, 1934	Parker, 1934	Parker, 1934
	Hoplophryne uluguruensis	Parker, 1934	Parker, 1934	Parker, 1934
	Hylophorbus picoides	Kraus, 2013	Kraus, 2013	----
	Hylophorbus rufescens	Zweifel, 1972	Zweifel, 1972	Parker, 1934
	Hypopachus variolosus	MT pers. obs.; Wever, 1985; Boulenger, 1882	MT pers. obs.; Wever, 1985	MT pers. obs.; Parker, 1934; Wever, 1985
	Kalophrynus baluensis	----	Inger & Stuebing, 1997	----
	Kalophrynus interlineatus	----	Parker, 1934	Parker, 1934
	Kalophrynus intermedius	----	Inger, 1966	----
	Kalophrynus pleurostigma	Zhao & Adler, 1993	Boulenger, 1882; Parker, 1934; Zhao & Adler, 1993	Parker, 1934
	Kaloula borealis	----	Parker, 1934	Parker, 1934
	Kaloula conjuncta	----	Parker, 1934	Parker, 1934
	Kaloula mediolineata	----	Parker, 1934	Parker, 1934
	Kaloula picta	----	Parker, 1934	Parker, 1934
	Kaloula pulchra	MT pers. obs.; Wever, 1985	MT pers. obs.; Wever, 1985	MT pers. obs.; Parker, 1934; Wever, 1985
	Liophryne rhododactyla	----	Parker, 1934	Parker, 1934
	Liophryne schlaginhaufeni	----	Parker, 1934	Parker, 1934
	Mantophryne lateralis	----	Zweifel, 1972	----
	Melanobatrachus indicus	Parker, 1934; Boulenger, 1882	Parker, 1934	Parker, 1934
	Metamagnusia slateri	----	Günther, 2009	----
	Metaphrynella pollicaris	----	Parker, 1934	Parker, 1934
	Metaphrynella sundana	----	Inger, 1966; Parker, 1934	Parker, 1934
	Microhyla achatina	----	Parker, 1934	Parker, 1934

Microhylidae (cont.)	*Microhyla annectens*	Inger, 1966	Parker, 1934	Parker, 1934
	Microhyla berdmorei	Parker, 1934	Inger, 1966; Parker, 1934	Parker, 1934 present; Vorobieva & Smirnov, 1987 absent
	Microhyla borneensis	Parker, 1934	Parker, 1934	Parker, 1934
	Microhyla butleri	Parker, 1934	Parker, 1934 present; Vorobieva & Smirnov, 1987 absent	Parker, 1934 present; Vorobieva & Smirnov, 1987 absent
	Microhyla heymonsi	----	Parker, 1934 present; Vorobieva & Smirnov, 1987 absent	Parker, 1934 present; Vorobieva & Smirnov, 1987
	Microhyla okinavensis	Parker, 1934	Parker, 1934	Parker, 1934
	Microhyla ornata	----	Smirnov, 1991	Vorobieva & Smirnov, 1987; Smirnov, 1991
	Microhyla palmipes	Parker, 1934	Parker, 1934	Parker, 1934
	Microhyla pulchra	Parker, 1934	Smirnov, 1991	Parker, 1934; Vorobieva & Smirnov, 1987; Smirnov, 1991
	Microhyla superciliaris	Parker, 1934	Parker, 1934	Parker, 1934
	Micryletta inornata	----	Parker, 1934	Parker, 1934
	Oreophryne monticola	----	Parker, 1934	Parker, 1934
	Oreophryne sibilans	Günther, 2003	Günther, 2003	----
	Oreophryne unicolor	Günther, 2003	Günther, 2003	----
	Otophryne pyburni	Boulenger, 1900a	Boulenger, 1900a	----
	Oxydactyla crassa	----	----	Zweifel, 2000
	Paradoxophyla palmata	Guibe, 1974	Guibe, 1974	----
	Phrynella pulchra	----	Parker, 1934	Parker, 1934
	Phrynomantis bifasciatus	Scott, 2005 Boulenger, 1882	Scott, 2005; Harper *et al.*, 2010	Scott, 2005
	Phrynomantis microps	MT pers. obs.	MT pers. obs.	MT pers. obs.
	Platypelis grandis	----	Parker, 1934	Parker, 1934
	Platypelis milloti	Glaw & Vences, 2007	Glaw & Vences, 2007	----

Microhylidae (cont.)	*Platypelis pollicaris*	Glaw & Vences, 2007	Glaw & Vences, 2007; Parker, 1934	Parker, 1934
	Platypelis tuberifera	----	Parker, 1934	Parker, 1934
	Plethodonthyla bipunctata	----	Guibe, 1974	Guibe, 1974
	Plethodonthyla guentheri	----	Glaw & Vences, 2007	----
	Plethodonthyla inguinalis	Glaw & Vences, 2007	Glaw & Vences, 2007; Parker, 1934	Parker, 1934
	Plethodonthyla mihanika	Glaw & Vences, 2007	Glaw & Vences, 2007	----
	Plethodonthyla notosticta	Glaw & Vences, 2007	Boulenger, 1882; Glaw & Vences, 2007; Parker, 1934	Parker, 1934
	Plethodonthyla ocellata	Glaw & Vences, 2007	Glaw & Vences, 2007; Parker, 1934	Parker, 1934
	Plethodonthyla tuberata	----	Parker, 1934	Parker, 1934
	Pseudocallulops eurydactylus	Günther, 2009	Günther, 2009; Zweifel, 1972	----
	Ramanella montana	----	Parker, 1934	Parker, 1934
	Ramanella obscura	----	Parker, 1934	Parker, 1934
	Ramanella variegata	----	Parker, 1934	Parker, 1934
	Rhombophryne alluaudi	Glaw & Vences, 2007	Glaw & Vences, 2007	----
	Rhombophryne coronata	Vences & Glaw, 2003	Vences & Glaw, 2003	----
	Rhombophryne coudreaui	Glaw & Vences, 2007	Glaw & Vences, 2007	----
	Rhombophryne grandis	----	Guibe, 1974	----
	Rhombophryne coudreaui	----	Glaw & Vences, 2007	----
	Rhombophryne psologlossa	----	Parker, 1934	Parker, 1934
	Rhombophryne serratopalpebrosa	Scherz *et al.*, 2014	Scherz *et al.*, 2014	----
	Rhombophryne testudo	Glaw & Vences, 2007	Glaw & Vences, 2007; Parker, 1934	Parker, 1934
	Rhombophryne tetradactyla	Glaw & Vences, 2007	Glaw & Vences, 2007	----
	Rhombophryne tridactyla	Glaw & Vences, 2007	Glaw & Vences, 2007	----
	Scaphiophryne calcarata	Nussbaum & Wu, 2007	Nussbaum & Wu, 2007	Nussbaum & Wu, 2007

Microhylidae (cont.)	*Scaphiophryne marmorata*	MT pers. obs.; Glaw & Vences, 2007	MT pers. obs.; Glaw & Vences, 2007	MT pers. obs.
	Scaphiophryne menabensis	Glaw & Vences, 2007	Glaw & Vences, 2007	----
	Sphenophryne cornuta	----	Boulenger, 1882; Parker, 1934	Parker, 1934
	Synapturanus mirandaribeiroi	MT pers. obs.	MT pers. obs.; Nelson & Lescure, 1975	MT pers. obs.
	Uperodon systoma	Boulenger, 1882	Parker, 1934	Parker, 1934
	Xenorhina bouwensi	----	Parker, 1934	Parker, 1934
	Xenorhina obesa	Zweifel, 1960	Zweifel, 1960	----
	Xenorhina oxycephala	----	Menzies, 2006	----
Myobatrachidae	*Assa darlingtoni*	Barker *et al.*, 1995	Tyler, 1972	Tyler, 1972
	Crinia deserticola	----	----	Davies, 1984; Davies, 1989
	Crinia parinsignifera	----	----	Davies, 1984; Davies, 1989
	Crinia riparia	----	----	Blake, 1973; Davies, 1984; Davies, 1989
	Crinia signifera	Boulenger, 1882	----	Lynch, 1971; Davies, 1984; Davies, 1989
	Crinia tinnula	----	----	Davies, 1984; Davies, 1989
	Geocrinia victoriana	MOP pers. obs.	MOP pers. obs.	MOP pers. obs.; Gollmann, 1981
	Metacrinia nichollsi	----	----	Lynch, 1971
	Mixophyes carbinensis	Anstis, 2013	Anstis, 2013	----
	Mixophyes coggeri	Anstis, 2013	Anstis, 2013	----
	Mixophyes fasciolatus	MOP pers. obs.; Barker *et al.*, 1995	MOP pers. obs.; Boulenger, 1882; Barker *et al.*, 1995	MOP pers. obs.; Lynch, 1971
	Mixophyes schevilli	Nussbaum & Wu, 2007; Barker *et al.*, 1995	Nussbaum & Wu, 2007; Barker *et al.*, 1995	Nussbaum & Wu, 2007
	Myobatrachus gouldii	----	----	Lynch, 1971
	Pseudophryne bibronii	Barker *et al.*, 1995	Barker *et al.*, 1995	Lynch, 1971; Heyer & Liem, 1976
	Pseudophryne coriacea	Barker *et al.*, 1995	Barker *et al.*, 1995	Heyer & Liem, 1976

Myobatrachidae (cont.)	*Rheobatrachus silus*	Barker *et al*., 1995	----	Davies & Burton, 1982
	Spicospina flammocaerulea	Roberts *et al*., 1997	Roberts *et al*., 1997	Roberts *et al*., 1997
	Taudactylus acutirostris	Barker *et al*., 1995	----	Lynch, 1971; Davies, 1984; Perry, 2004
	Uperoleia aspera	Barker *et al*., 1995	----	Tyler *et al*., 1981
	Uperoleia borealis	----	----	Davies, 1989
	Uperoleia crassa	----	----	Davies, 1989
	Uperoleia fusca	----	----	Davies, 1989
	Uperoleia glandulosa	----	----	Davies, 1989
	Uperoleia inundata	----	----	Davies, 1989
	Uperoleia laevigata	Barker *et al*., 1995	----	Davies, 1989; Perry, 2004
	Uperoleia lithomoda	----	----	Davies, 1989
	Uperoleia littlejohni	----	----	Davies, 1989
	Uperoleia micromeles	----	----	Davies, 1989
	Uperoleia minima	----	----	Davies, 1989
	Uperoleia mjobergii	----	----	Tyler *et al*., 1981; Davies, 1989
	Uperoleia rugosa	Barker *et al*., 1995	----	Lynch, 1971; Davies, 1989; Perry, 2004
	Uperoleia russelli	Barker *et al*., 1995	----	Lynch, 1971 Davies, 1989
	Uperoleia talpa	----	----	Davies, 1989
	Uperoleia trachyderma	----	----	Davies, 1989
	Uperoleia tyleri	----	----	Davies, 1989
Nasikabatrachidae	*Nasikabatrachus sahyadrensis*	Biju & Bossuyt, 2003	----	Senevirathne *et al*. 2016
Nyctibatrachidae	*Nyctibatrachus beddomii*	----	Inger *et al*., 1984	----
	Nyctibatrachus deccanensis	Boulenger, 1882	----	----
	Nyctibatrachus kempholeyensis	----	Rao, 1937	----
	Nyctibatrachus major	Boulenger, 1882	----	----

Nyctibatrachidae (cont.)	*Nyctibatrachus minimus*	----	Biju *et al.*, 2007	----
	Nyctibatrachus minor	----	Inger *et al.*, 1984	----
	Nyctibatrachus sylvaticus	----	Rao, 1937; Chandramouli & Dutta, 2015	Chandramouli & Dutta, 2015
Odontophrynidae	*Macrogenioglottus alipioi*	DB pers. obs.	DB pers. obs.	Reig, 1972
	Odontophrynus achalensis	MOP pers. obs.	MOP pers. obs.	MOP pers. obs.; Lynch, 1971
	Odontophrynus americanus	DB pers. obs.	DB pers. obs.	Lynch, 1971
	Odontophrynus carvalhoi	DB pers. obs.	DB pers. obs.	Lynch, 1971
	Odontophrynus cultripes	DB pers. obs.	DB pers. obs.	Lynch, 1971
	Proceratophrys appendiculata	Dias *et al.*, 2013	Boulenger, 1882; Dias *et al.*, 2013	Dias *et al.*, 2013; Lynch, 1971
	Proceratophrys avelinoi	DB pers. obs.	BLB pers. obs.	BLB pers. obs.
	Proceratophrys boiei	DB pers. obs.	Boulenger, 1882	Lynch, 1971
	Proceratophrys concavitympanum	----	Giaretta *et al.*, 2000	----
	Proceratophrys cristiceps	DB pers. obs.	DB pers. obs.	Lynch, 1971
	Proceratophrys laticeps	----	----	Izecksohn *et al.*, 2005
	Proceratophrys melanopogon	----	----	Izecksohn *et al.*, 2005
Pelodytidae	*Pelobates cultripes*	----	----	Cannatella & Trueb, 1988
	Pelobates fuscus	Laurent, 1986	----	Cannatella & Trueb, 1988
	Pelobates syriacus	----	----	Cannatella & Trueb, 1988
	Pelobates varaldii	----	----	Maglia, 1998
	Pelodytes caucasicus	----	----	Sanchiz *et al.*, 2002
	Pelodytes ibericus	----	----	Sanchiz *et al.*, 2002
	Pelodytes punctatus	----	----	Cannatella & Trueb, 1988; Sanchiz *et al.*, 2002
Petropedetidae	*Arthroleptides martiensseni*	Scott, 2005	Scott, 2005	Scott, 2005
	Arthroleptides yakusini	Harper *et al.*, 2010	Harper *et al.*, 2010	----
	Aubria subsigillata	Scott, 2005	Boulenger, 1882; Scott, 2005	Scott, 2005

Petropedetidae (cont.)	*Petropedetes cameronensis*	Scott, 2005	Scott, 2005	Scott, 2005
	Petropedetes newtoni	Scott, 2005	Scott, 2005	Liu, 1936; Scott, 2005
	Petropedetes parkeri	Scott, 2005	Scott, 2005	Scott, 2005
Phrynobatrachidae	*Phrynobatrachus acridoides*	Scott, 2005; du Preez & Carruthers, 2009; Spawls *et al.*, 2006	Scott, 2005; du Preez & Carruthers, 2009; Spawls *et al.*, 2006	Scott, 2005
	Phrynobatrachus africanus	Scott, 2005	Scott, 2005	Scott, 2005
	Phrynobatrachus calcaratus	Boulenger, 1882	----	----
	Phrynobatrachus cricogaster	Scott, 2005	Scott, 2005	Scott, 2005
	Phrynobatrachus dispar	----	Boulenger, 1882	----
	Phrynobatrachus krefftii	Scott, 2005	Scott, 2005	Scott, 2005
	Phrynobatrachus natalensis	Scott, 2005; du Preez & Carruthers, 2009	Scott, 2005; du Preez & Carruthers, 2009	Scott, 2005
	Phrynobatrachus sandersoni	Scott, 2005	Scott, 2005	Scott, 2005
Pipidae	*Hymenochyrus boettgeri*	----	Rabb & Rabb, 1963	Trueb & Cannatella, 1982
	Pipa carvalhoi	----	----	Trueb & Cannatella, 1982
	Pipa parva	----	Cannatella & Trueb, 1988	Cannatella & Trueb, 1988
	Pipa pipa	Wever, 1985	Wever, 1985	Wever, 1985; Trueb *et al.*, 2000; Trueb & Cannatella, 1982
	Xenopus borealis	Wever, 1985	Wever, 1985; Cannatella & Trueb, 1988	Wever, 1985
	Xenopus epitropicalis	----	Cannatella & Trueb, 1988	Cannatella & Trueb, 1988
	Xenopus fraseri	----	Evans *et al.*, 2015	Evans *et al.*, 2015
	Xenopus laevis	Wever, 1985	Wever, 1985; Cannatella & Trueb, 1988	Trueb & Cannatella, 1982
	Xenopus muelleri	----	Cannatella & Trueb, 1988	Cannatella & Trueb, 1988
	Xenopus tropicalis	Cannatella & Trueb, 1988	Cannatella & Trueb, 1988	Cannatella & Trueb, 1988

Ptychadenidae	*Hildebrandtia ornata*	Scott, 2005; du Preez & Carruthers, 2009	Scott, 2005; Boulenger, 1882; du Preez & Carruthers, 2009	----
	Ptychadena aequiplicata	----	Boulenger, 1900c	----
	Ptychadena anchietae	Scott, 2005; du Preez & Carruthers, 2009	Scott, 2005; du Preez & Carruthers, 2009	Scott, 2005
	Ptychadena mascareniensis	Scott, 2005; du Preez & Carruthers, 2009	Scott, 2005; du Preez & Carruthers, 2009	Scott, 2005
	Ptychadena oxyrhynchus	du Preez & Carruthers, 2009	Boulenger, 1882; du Preez & Carruthers, 2009	----
	Ptychadena porosissima	du Preez & Carruthers, 2009	du Preez & Carruthers, 2009	----
	Ptychadena taenioscelis	Harper *et al.*, 2010	Harper *et al.*, 2010	----
Pyxicephalidae	*Amietia angolensis*	Scott, 2005; du Preez & Carruthers, 2009; Spawls *et al.*, 2006	Scott, 2005; du Preez & Carruthers, 2009; Spawls *et al.*, 2006	Scott, 2005
	Amietia fuscigula	Scott, 2005	Scott, 2005; Boulenger, 1882	Scott, 2005
	Anhydrophryne rattrayi	Scott, 2005	Scott, 2005	Scott, 2005
	Arthroleptella landdrosia	Scott, 2005; du Preez & Carruthers, 2009	Scott, 2005; du Preez & Carruthers, 2009	Scott, 2005
	Arthroleptella lightfooti	----	-----	Laurent, 1941a
	Cacosternum boettgeri	Scott, 2005; Nussbaum & Wu, 2007; du Preez & Carruthers, 2009	Scott, 2005; Nussbaum & Wu, 2007; du Preez & Carruthers, 2009	Scott, 2005; Nussbaum & Wu, 2007
	Cacosternum capense	Scott, 2005; du Preez & Carruthers, 2009	Scott, 2005	Scott, 2005
	Cacosternum nanum	Scott, 2005	Scott, 2005	Scott, 2005
	Microbatrachella capensis	Scott, 2005	Scott, 2005	Scott, 2005
	Natalobatrachus bonebergi	Scott, 2005; du Preez & Carruthers, 2009	Scott, 2005; du Preez & Carruthers, 2009	Laurent, 1941a; Scott, 2005
	Poyntonia paludicola	Scott, 2005; du Preez & Carruthers, 2009	Scott, 2005	Scott, 2005

Pyxicephalidae (cont.)	*Pyxicephalus adspersus*	Scott, 2005; du Preez & Carruthers, 2009	Scott, 2005; Haas, 1999; Boulenger, 1882; du Preez & Carruthers, 2009	Scott, 2005; Haas, 1999
	Pyxicephalus edulis	Scott, 2005; du Preez & Carruthers, 2009	Scott, 2005; du Preez & Carruthers, 2009	Scott, 2005
	Strongylopus fasciatus	du Preez & Carruthers, 2009	Boulenger, 1882; du Preez & Carruthers, 2009	----
	Strongylopus grayii	Scott, 2005	Boulenger, 1882; Scott, 2005	du Troit, 1933; Scott, 2005
	Tomopterna delalandii	du Preez & Carruthers, 2009; Boulenger, 1882	Boulenger, 1882	----
	Tomopterna marmorata	Scott, 2005	Scott, 2005	Scott, 2005
	Tomopterna natalensis	du Preez & Carruthers, 2009	Boulenger, 1882	----
	Tomopterna tandyi	Scott, 2005	Scott, 2005	Scott, 2005
	Tomopterna tuberculosa	du Preez & Carruthers, 2009	Boulenger, 1882	----
Ranidae	*Abavorana luctuosa*	Inger & Stuebing, 1997	Inger, 1966; Inger & Stuebing, 1997	----
	Amnirana albolabris	Scott, 2005; Spawls *et al.*, 2006	Boulenger, 1882; Scott, 2005; Spawls *et al.*, 2006	Scott, 2005
	Amnirana galamensis	Scott, 2005; du Preez & Carruthers, 2009; Spawls *et al.*, 2006	Boulenger, 1882; Scott, 2005; du Preez & Carruthers, 2009; Spawls *et al.*, 2006	Scott, 2005
	Amolops chunganensis	----	Yang, 1991	----
	Amolops daorum	Bain *et al.*, 2003	Bain *et al.*, 2003	----
	Amolops granulosus	----	Yang, 1991	----
	Amolops hainanensis	----	Yang, 1991	----
	Amolops hongkongensis	----	Yang, 1991	----
	Amolops jinjiangensis	----	Yang, 1991	----
	Amolops larutensis	----	Yang, 1991	----
	Amolops loloensis	----	Yang, 1991	----
	Amolops montzorum	----	Yang, 1991	----
	Amolops ricketti	Scott, 2005	Inger *et al.*, 1999; Scott, 2005	Scott, 2005

Ranidae (cont.)	*Amolops spinapectoralis*	Inger *et al.*, 1999	Inger *et al.*, 1999	----
	Amolops viridimaculatus	Zhao & Adler, 1993	Zhao & Adler, 1993	----
	Babina adenopleura	----	Liu, 1936	----
	Babina okinavana	----	Boettger, 1895	----
	Babina pleuraden	----	Liu, 1950	----
	Chalcorana chalconota	Inger & Stuebing, 1997	Boulenger, 1882; Inger & Stuebing, 1997	----
	Chalcorana eschatia	----	Inger *et al.*, 2009	----
	Chalcorana megalonesa	----	Inger *et al.*, 2009	----
	Chalcorana parvaccola	----	Inger *et al.*, 2009	----
	Chalcorana raniceps	----	Inger *et al.*, 2009	----
	Clinotarsus alticola	----	Boulenger, 1882	----
	Clinotarsus curtipes	----	Boulenger, 1882	----
	Glandirana rugosa	----	Boulenger, 1882	----
	Glandirana tientaiensis	Zhao & Adler, 1993	Zhao & Adler, 1993	----
	Huia cavitympanum	Inger, 1966	Inger, 1966	----
	Huia masonii	----	Iskandar, 1998	----
	Hydrophylax gracilis	Parker, 1881	Parker, 1881	Parker, 1881
	Hydrophylax malabaricus	Biju *et al.*, 2014b	Boulenger, 1882; Biju *et al.*, 2014b	----
	Hylarana erythrea	Teynie *et al.*, 2010	Inger, 1966; Boulenger, 1882	----
	Hylarana macrodactyla	----	Liu, 1936; Boulenger, 1882	----
	Hylarana taipehensis	Zhao & Adler, 1993	Inger *et al.*, 1999; Zhao & Adler, 1993	Liu, 1936
	Indosylvirana aurantiaca	Biju *et al.*, 2014b	Biju *et al.*, 2014b	----
	Indosylvirana milleti	----	Inger *et al.*, 1999	----
	Lithobates areolatus	Elliot *et al.*, 2009	Elliot *et al.*, 2009	----
	Lithobates berlandieri	----	McCranie & Wilson, 2002	----
	Lithobates blairi	Elliot *et al.*, 2009	Elliot *et al.*, 2009	----

Ranidae (cont.)	*Lithobates bwana*	Hillis & de Sá, 1988	Hillis & de Sá, 1988	----
	Lithobates capito	----	Boulenger, 1882	----
	Lithobates catesbeianus	Wever, 1985	Wever, 1985; Boulenger, 1882	Wever, 1985
	Lithobates chiricahuensis	Elliot *et al.*, 2009	Elliot *et al.*, 2009	----
	Lithobates clamitans	Wever, 1985	Wever, 1985; Boulenger, 1882	Wever, 1985
	Lithobates fisheri	Dood, 2013	Dood, 2013	----
	Lithobates forreri	Savage, 2002	Savage, 2002	----
	Lithobates grylio	Elliot *et al.*, 2009	Elliot *et al.*, 2009	----
	Lithobates heckscheri	Elliot *et al.*, 2009	Elliot *et al.*, 2009	----
	Lithobates juliani	Hillis & de Sá, 1988	Hillis & de Sá, 1988	----
	Lithobates macroglossa	----	Boulenger, 1882; Inger, 1966	----
	Lithobates maculatus	Hillis & de Sá, 1988	Hillis & de Sá, 1988	----
	Lithobates montezumae	----	Boulenger, 1882	----
	Lithobates okaloosae	Elliot *et al.*, 2009	Elliot *et al.*, 2009	----
	Lithobates onca	Elliot *et al.*, 2009	Elliot *et al.*, 2009	----
	Lithobates palmipes	Kok & Kalamandeen, 2008	Kok & Kalamandeen, 2008	----
	Lithobates palustris	Elliot *et al.*, 2009	Elliot *et al.*, 2009	----
	Lithobates pipiens	Scott, 2005	Scott, 2005	Scott, 2005
	Lithobates septentrionalis	Elliot *et al.*, 2009	Elliot *et al.*, 2009	----
	Lithobates sevosus	Dood, 2013	Dood, 2013	----
	Lithobates sierramadrensis	Hillis & de Sá, 1988	Hillis & de Sá, 1988	----
	Lithobates sphenocephalus	Elliot *et al.*, 2009	Elliot *et al.*, 2009	----
	Lithobates sylvaticus	----	Boulenger, 1882	----
	Lithobates tarahumarae	Elliot *et al.*, 2009	Elliot *et al.*, 2009	----
	Lithobates taylori	Savage, 2002	Savage, 2002	----
	Lithobates vaillanti	Savage, 2002	Savage, 2002	----
	Lithobates vibicarius	Savage, 2002	Savage, 2002	----
	Lithobates virgatipes	Wever, 1985	Wever, 1985	Wever, 1985

Ranidae (cont.)	*Lithobates warszewitschii*	Jaslow *et al.*, 1988; Savage, 2002	Jaslow *et al.*, 1988; Boulenger, 1882; Savage, 2002	Jaslow *et al.*, 1988
	Lithobates yavapaiensis	Elliot *et al.*, 2009	Elliot *et al.*, 2009	----
	Meristogenys jerboa	----	Inger, 1966; Boulenger, 1882	----
	Meristogenys kinabaluensis	Inger & Stuebing, 1997	Inger & Stuebing, 1997	----
	Meristogenys phaeomerus	Inger & Stuebing, 1997	Yang, 1991	----
	Meristogenys poecilus	----	Yang, 1991	----
	Meristogenys whiteheadi	----	Inger & Stuebing, 2009	----
	Odorrana andersonii	----	Liu, 1936; Boulenger, 1882	----
	Odorrana bacboensis	Bain *et al.*, 2003	Bain *et al.*, 2003	----
	Odorrana chloronota	Bain *et al.*, 2003	Inger, 1966; Boulenger, 1882	Parker, 1881
	Odorrana grahami	----	Bain *et al.*, 2003	----
	Odorrana hosii	----	Bain *et al.*, 2003	----
	Odorrana ishikawae	----	Stejneger, 1901	----
	Odorrana jingdongensis	----	Bain *et al.*, 2003	----
	Odorrana junlianensis	----	Bain *et al.*, 2003	----
	Odorrana khalam	Stuart *et al.*, 2005	Stuart *et al.*, 2005	----
	Odorrana livida	Bain *et al.*, 2003	Bain *et al.*, 2003	----
	Odorrona margaretae	Zhao & Adler, 1993	Liu, 1950; Zhao & Adler, 1993	----
	Odorrana morafkai	----	Bain *et al.*, 2003	----
	Odorrana schmackeri	Zhao & Adler, 1993	Zhao & Adler, 1993	----
	Odorrana swinhoana	Zhao & Adler, 1993	Zhao & Adler, 1993	----
	Odorrana tiannanensis	Zhao & Adler, 1993	Zhao & Adler, 1993	----
	Odorrana tormota	Gridi-Papp *et al.*, 2008	Gridi-Papp *et al.*, 2008	Gridi-Papp *et al.*, 2008
	Papurana arfaki	----	Boulenger, 1882	----
	Papurana daemeli	Barker *et al.*, 1995	Barker *et al.*, 1995	----
	Pelophylax cerigensis	Beerli *et al.*, 1994	Beerli *et al.*, 1994	----
	Pelophylax cretensis	Beerli *et al.*, 1994	Beerli *et al.*, 1994	----
	Pelophylax epeiroticus	----	Schneider *et al.*, 1984	----

Ranidae (cont.)	*Pelophylax esculentus*	Boulenger, 1882	Ecker, 1889	Ecker, 1889
	Pelophylax fukienensis	Zhao & Adler, 1993	Zhao & Adler, 1993	----
	Pelophylax nigromaculatus	Zhao & Adler, 1993	Liu, 1950; Zhao & Adler, 1993	----
	Pelophylax plancyi	Zhao & Adler, 1993	Zhao & Adler, 1993; Boulenger, 1882	Liu, 1936
	Pelophylax ridibundus	Wever, 1985	Wever, 1985	Wever, 1985
	Pelophylax saharicus	Schleich *et al*., 1996	Schleich *et al*., 1996	----
	Pulchrana baramica	----	Iskandar, 1998; Inger, 1966	----
	Pulchrana glandulosa	----	Inger, 1966; Boulenger, 1882	----
	Pulchrana signata	----	Inger, 1966; Boulenger, 1882	----
	Rana arvalis	Boulenger, 1882	Boulenger, 1882	----
	Rana aurora	Elliot *et al*., 2009	Elliot *et al*., 2009	----
	Rana boylii	Elliot *et al*., 2009	Elliot *et al*., 2009	----
	Rana cascadae	Elliot *et al*., 2009	Elliot *et al*., 2009	----
	Rana chaochiaoensis	----	Liu, 1950	----
	Rana chensinensis	----	Liu, 1950	----
	Rana dalmatina	----	Boulenger, 1882	----
	Rana hanluica	----	Shen et *al*., 2007	----
	Rana iberica	----	Boulenger, 1882	----
	Rana japonica	----	Liu, 1936; Boulenger, 1882	----
	Rana johnsi	----	Smith, 1921	----
	Rana latastei	----	Boulenger, 1882	----
	Rana longicrus	Zhao & Adler, 1993	Zhao & Adler, 1993	----
	Rana luteiventris	Elliot *et al*., 2009	Elliot *et al*., 2009	----
	Rana macrocnemis	----	Picariello *et al*., 1999	----
	Rana muscosa	Elliot *et al*., 2009	Elliot *et al*., 2009	----
	Rana pirica	Matsui, 1991	Matsui, 1991	----
	Rana pretiosa	----	Boulenger, 1882	----
	Rana sauteri	Zhao & Adler, 1993	Zhao & Adler, 1993	----

Ranidae (cont.)	*Rana shuchinae*	----	Liu, 1950	----
	Rana tagoi	----	Ryuzaki *et al*., 2014	----
	Rana temporaria	----	Boulenger, 1882	----
	Sanguina sanguinea	Inger, 1954	Inger, 1954	Inger, 1954
	Staurois latopalmatus	----	Inger & Stuebing, 1997	----
	Staurois natator	Scott, 2005	Scott, 2005 Boulenger, 1882	Scott, 2005
	Staurois tuberilinguis	----	Inger, 1966	----
	Sylvirana guentheri	Zhao & Adler, 1993	Zhao & Adler, 1993; Boulenger, 1882	----
	Sylvirana maosonensis	----	Inger *et al*., 1999	----
	Sylvirana nigrovittata	----	Smith, 1921	----
Ranixalidae	*Indirana beddomi*	----	Inger *et al*., 1984; Boulenger, 1882	----
	Indirana brachytarsus	----	Inger *et al*., 1984	----
	Indirana diplosticta	----	Inger *et al*., 1984	----
	Indirana semipalmata	----	Inger *et al*., 1984; Chandramouli & Dutta, 2015	Chandramouli & Dutta, 2015
Rhacophoridae	*Buergeria buergeri*	----	Boulenger, 1882	----
	Buergeria japonica	Zhao & Adler, 1993	Zhao & Adler, 1993	----
	Buergeria robusta	Zhao & Adler, 1993	Zhao & Adler, 1993	----
	Chiromantis doriae	----	Zhao & Adler, 1993	----
	Chiromantis petersii	----	Boulenger, 1882	Laurent, 1941b
	Chiromantis rufescens	----	Boulenger, 1882	Laurent, 1941b
	Chiromantis vittatus	----	Orlov *et al*., 2012	----
	Chiromantis xerampelina	du Preez & Carruthers, 2009; Scott, 2005	du Preez & Carruthers, 2009; Scott, 2005	du Preez & Carruthers, 2009; Scott, 2005
	Feihyla kajau	----	Inger & Stuebing, 1997	----
	Feihyla palpebralis	----	Inger *et al*., 2012	----
	Ghatixalus variabilis	----	----	Laurent, 1943
	Gracixalus gracilipes	----	Rowley *et al*., 2011	----

Rhacophoridae (cont.)	*Gracixalus supercornutus*	----	Rowley *et al.*, 2011	----
	Kurixalus appendiculatus	----	Inger, 1966; Boulenger, 1882	----
	Kurixalus banaensis	----	Orlov *et al.*, 2012	----
	Kurixalus eiffingeri	Zhao & Adler, 1993	Zhao & Adler, 1993	----
	Kurixalus idiooticus	Wu *et al.*, 2016	Wu *et al.*, 2016	
	Kurixalus odontotarsus	----	Orlov *et al.*, 2012	----
	Liuixalus romeri	Zhao & Adler, 1993	Zhao & Adler, 1993	----
	Nyctixalus pictus	Inger & Stuebing, 1997	Inger & Stuebing, 1997	----
	Philautus acutirostris	Boulenger, 1882	----	----
	Philautus acutus	Dring, 1987	Dring, 1987	----
	Philautus aurantium	----	Inger & Stuebing, 1997	----
	Philautus aurifasciatus	----	Inger, 1966; Boulenger, 1882	Inger, 1966
	Philautus bunitus	Inger & Stuebing, 1997	Inger & Stuebing, 1997	----
	Philautus hosii	----	Inger, 1966	----
	Philautus petersi	Inger & Stuebing, 1997	Inger & Stuebing, 1997	----
	Philautus surdus	Scott, 2005; Boulenger, 1882	Scott, 2005	Scott, 2005
	Philautus umbra	----	Dring, 1987	----
	Polypedates colletti	----	Inger, 1966	----
	Polypedates leucomystax	----	Inger, 1966	Laurent, 1943
	Polypedates macrotis	----	Inger, 1966	Liem, 1970
	Polypedates maculatus	Wever, 1985	Wever, 1985; Boulenger, 1882	Wever, 1985
	Polypedates otilophus	----	Inger, 1966	Liem, 1970
	Pseudophilautus alto	----	Manamendra-Arachchi & Pethiyagoda, 2005	----
	Pseudophilautus amboli	Biju & Bossuyt, 2009	Biju & Bossuyt, 2009	
	Pseudophilautus cavirostris	----	Boulenger, 1882; Manamendra-Arachchi & Pethiyagoda, 2005	----
	Pseudophilautus decoris	Wickramasinghe *et al.*, 2013	Wickramasinghe *et al.*, 2013	----

Rhacophoridae (cont.)	*Pseudophilautus femoralis*	----	Günther, 1864	----
	Pseudophilautus folicola	----	Wickramasinghe *et al.*, 2013	----
	Pseudophilautus hoffmanni	Wickramasinghe *et al.*, 2013	Wickramasinghe *et al.*, 2013	----
	Pseudophilautus jayarami	Biju & Bossuyt, 2009	Biju & Bossuyt, 2009	----
	Pseudophilautus kani	Biju & Bossuyt, 2009	Biju & Bossuyt, 2009	----
	Pseudophilautus leucorhinus	----	Boulenger, 1882	----
	Pseudophilautus lunatus	----	Manamendra-Arachchi & Pethiyagoda, 2005	----
	Pseudophilautus mooreorum	----	Meegaskumbara & Manamendra-Arachchi, 2005	----
	Pseudophilautus microtympanum	----	Manamendra-Arachchi & Pethiyagoda, 2005	----
	Pseudophilautus ocularis	----	Manamendra-Arachchi & Pethiyagoda, 2005	----
	Pseudophilautus papillosus	----	Manamendra-Arachchi & Pethiyagoda, 2005	----
	Pseudophilautus pleurotaenia	----	Manamendra-Arachchi & Pethiyagoda, 2005	----
	Pseudophilautus popularis	----	Manamendra-Arachchi & Pethiyagoda, 2005	----
	Pseudophilautus reticulatus	----	Boulenger, 1882	----
	Pseudophilautus sarasinorum	----	Manamendra-Arachchi & Pethiyagoda, 2005	----
	Pseudophilautus schmarda	----	Boulenger, 1882	----
	Pseudophilautus tanu	Wickramasinghe *et al.*, 2013	Wickramasinghe *et al.*, 2013	----
	Pseudophilautus zorro	----	Wickramasinghe *et al.*, 2013	----
	Raorchestes anili	----	Seshadri *et al.*, 2012	----
	Raorchestes beddomii	----	Boulenger, 1882	----
	Raorchestes charius	----	Rao, 1937	----
	Raorchestes chlorosoma	----	Seshadri *et al.*, 2012	----
	Raorchestes chotta	Seshadri *et al.*, 2012	----	----

Rhacophoridae (cont.)	*Raorchestes chromasynchysi*	----	Seshadri *et al*., 2012	----
	Raorchestes coonoorensis	----	Seshadri *et al*., 2012	----
	Raorchestes dubois	----	Seshadri *et al*., 2012	----
	Raorchestes glandulosus	Boulenger, 1882	Seshadri *et al*., 2012	Laurent, 1943
	Raorchestes graminirupes	----	Seshadri *et al*., 2012	----
	Raorchestes griet	----	Seshadri *et al*., 2012	----
	Raorchestes luteolus	----	Kuramoto & Joshy, 2003	----
	Raorchestes marki	----	Seshadri *et al*., 2012	----
	Raorchestes munnarensis	----	Seshadri *et al*., 2012	----
	Raorchestes nerostagona	----	Seshadri *et al*., 2012	----
	Raorchestes ponmudi	----	Seshadri *et al*., 2012	----
	Raorchestes sushili	----	Seshadri *et al*., 2012	----
	Raorchestes tinniens	----	Boulenger, 1882; Rao, 1937; Seshadri *et al*., 2012	----
	Raorchestes tuberohumerus	----	Kuramoto & Joshy, 2003	----
	Rhacophorus annamensis	----	Stuart *et al*., 2006	----
	Rhacophorus angulirostris	Inger & Stuebing, 1997	Inger & Stuebing, 1997	----
	Racophorus calcaneus	----	Stuart *et al*., 2006	----
	Rhacophorus chenfui	Zhao & Adler, 1993	Liu, 1950; Zhao & Adler, 1993	----
	Rhacophorus dennysi	----	Boulenger, 1882	Liem, 1970
	Rhacophorus dugritei	Zhao & Adler, 1993	Liu, 1950; Boulenger, 1882	----
	Rhacophorus dulitensis	----	Inger, 1966	----
	Rhacophorus fasciatus	----	Inger, 1966	----
	Rhacophorus gauni	----	Inger, 1966	----
	Rhacophorus harrisoni	Inger & Stuebing, 1997	Inger & Stuebing, 1997	----
	Rhacophorus maximus	----	Günther, 1858	----
	Rhacophorus minimus	Rao *et al*., 2006	Rao *et al*., 2006	----
	Rhacophorus nigropalmatus	Wever, 1985; Inger, 1966	Wever, 1985	Wever, 1985
	Rhacophorus omeimontis	Zhao & Adler, 1993	Liu, 1950; Zhao & Adler, 1993	----

Rhacophoridae (cont.)	*Rhacophorus orlovi*	Ziegler & Köhler, 2001	Ziegler & Köhler, 2001	----
	Rhacophorus pardalis	Inger & Stuebing, 1997	Inger & Stuebing, 1997	----
	Rhacophorus reindwartii	Zhao & Adler, 1993	Inger *et al.*, 1999; Zhao & Adler, 1993	----
	Rhacophorus rhodopus	Zhao & Adler, 1993	Zhao & Adler, 1993	----
	Rhacophorus rufipes	Inger & Stuebing, 1997	Inger & Stuebing, 1997	----
	Rhacophorus schlegelii	----	Boulenger, 1882	Laurent, 1943
	Taruga eques	----	Boulenger, 1882; Meegaskumbura *et al.*, 2010	Meegaskumbura *et al.*, 2010
	Taruga fastigo	----	Meegaskumbura *et al.*, 2010	----
	Taruga longinasus	----	Meegaskumbura *et al.*, 2010	----
	Theloderma asperum	Zhao & Adler, 1993	Zhao & Adler, 1993	----
	Theloderma corticale	----	Zhao & Adler, 1993	----
	Theloderma moloch	-----	Annandale, 1912	----
	Theloderma stellatum	----	Zhao & Adler, 1993	----
Rhinodermatidae	*Insuetophrynus acarpicus*	Barrio, 1970	Barrio, 1970	Lynch, 1978; Barrio, 1970
	Rhinoderma darwinii	Nussbaum & Wu, 2007	Nussbaum & Wu, 2007; Boulenger, 1882	MOP pers. obs.; Laurent, 1942; Lynch, 1971; Nussbaum & Wu, 2007
Rhinophrynidae	*Rhinophrynus dorsalis*	Wever, 1985	Wever, 1985	Lynch, 1973; Trueb & Cannatella, 1982; Wever, 1985
Scaphiopodidae	*Scaphiopus couchii*	Wever, 1985; Nussbaum & Wu, 2007	Wever, 1985; Nussbaum & Wu, 2007	Wever, 1985; Nussbaum & Wu, 2007
	Scaphiopus holbrookii	Laurent, 1986;	Laurent, 1986	Laurent, 1986
	Scaphiopus hurteri	Wever, 1985	Wever, 1985	Wever, 1985
	Spea bombifrons	Wever, 1985	Wever, 1985	Wiens, 1989; Wever, 1985
	Spea hammondi	Wever, 1985; Boulenger, 1882	Wever, 1985	Wever, 1985
	Spea intermontana	----	Hall & Larsen, 1998	Hall & Larsen, 1998
	Spea multiplicata	----	----	Maglia, 1998

Sooglossidae	*Sechellophryne gardineri*	Boistel *et al*., 2013; Nussbaum & Wu, 2007	Nussbaum & Wu, 2007	Nussbaum & Wu, 2007; Boistel *et al*., 2013
	Sooglosus sechellensis	Scott, 2005; Nussbaum & Wu, 2007	Scott, 2005; Nussbaum & Wu, 2007	Scott, 2005; Nussbaum & Wu, 2007
	Sooglosus thomasseti	Nussbaum & Wu, 2007	Nussbaum & Wu, 2007	Nussbaum & Wu, 2007
Telmatobiidae	*Telmatobius bolivianus*	Lavilla & Ergueta Sandoval, 1999	Parker, 1940; Lavilla & Ergueta Sandoval, 1999	Lavilla & Ergueta Sandoval, 1999
	Telmatobius culeus	JSB pers. obs.	JSB pers. obs.; Jaslow *et al*., 1988	JSB pers. obs.; Jaslow *et al*., 1988
	Telmatobius gigas	de la Riva, 2002a	----	----
	Telmatobius hintoni	JSB pers. obs.	JSB pers. obs.	JSB pers. obs.
	Telmatobius huayra	Lavilla & Ergueta Sandoval, 1995	Lavilla & Ergueta Sandoval, 1995	Lavilla & Ergueta Sandoval, 1995
	Telmatobius marmoratus	JSB pers. obs.	JSB pers. obs.	JSB pers. obs.; Lynch, 1971; Nussbaum & Wu, 2007
	Telmatobius niger	Trueb, 1979	Trueb, 1979	Trueb, 1979
	Telmatobius sibiricus	JSB pers. obs;.de la Riva & Harvey, 2003	JSB pers. obs.	JSB pers. obs.
	Telmatobius simonsi	JSB pers. obs;.de la Riva & Harvey, 2003	JSB pers. obs;.de la Riva & Harvey, 2003	JSB pers. obs;.de la Riva & Harvey, 2003
	Telmatobius truebae	----	----	Wiens, 1993
	Telmatobius vellardi	Trueb, 1979	Trueb, 1979	Trueb, 1979
	Telmatobius verrucosus	JSB pers. obs.	JSB pers. obs.	----
	Telmatobius vilamensis	Formas *et al*., 2003	Formas *et al*., 2003	Formas *et al*., 2003
	Telmatobius yuracare	de la Riva & Harvey, 2003	JSB pers. obs.	JSB pers. obs.
	Telmatobius zapahuirensis	Veloso *et al*., 1982	Veloso *et al*., 1982	----

Anura not included in Pyron (2014)				
**Family**	**Species**	**Tympanic membrane**	**Tympanic annulus**	**Columella**
Alsodidae	*Eupsophus altor*	----	----	Nuñez *et al.*, 2012
Arthroleptidae	*Arthroleptis phrynoides*	Liu, 1950	Liu, 1950	----
	Cardioglossa escalaerae	Scott, 2005	Scott, 2005	Scott, 2005
	Letodactylodon ventrimarmoratus	Scott, 2005	Scott, 2005	Scott, 2005
	Leptopelis mossambicus	Scott, 2005	Scott, 2005	Scott, 2005
Batrachylidae	*Atelognathus nitoi*	----	----	Lynch, 1978
	Atelognathus praebasalticus	----	----	Lynch, 1978
	Atelognathus reverberii	----	----	Lynch, 1978
	Atelognathus solitarius	----	----	Lynch, 1978
	Chaltenobatrachus grandisonae	Basso *et al.*, 2011	Basso *et al.*, 2011	Basso *et al.*, 2011
Brachycephalidae	*Ischnocnema nigriventris*	----	----	Lynch, 1971
Callyptocephalellidae	*Telmatobufo australis*	Formas *et al.*, 2001	Formas *et al.*, 2001	Formas *et al.*, 2001
Ceratobatrachidae	*Alcalus mariae*	----	Fuiten, 2012	Fuiten, 2012
	Cornufer bufoniformis	Scott, 2005	Scott, 2005	Scott, 2005
Ceratophryidae	*Ceratophrys aurita*	----	----	Lynch, 1971
	Ceratophrys calcarata	----	----	Lynch, 1971
	Ceratophrys cranwelli	MOP pers. obs.	MOP pers. obs.	MOP pers. obs.
Craugastoridae	*Bryophryne abramalagae*	Lehr & Catenazzi, 2010	Lehr & Catenazzi, 2010	----
	Bryophryne bakersfield	Chaparro *et al.*, 2015	Chaparro *et al.*, 2015	----
	Bryophryne bustamantei	Chaparro *et al.*, 2007	Chaparro *et al.*, 2007	----
	Bryophryne flammiventris	Lehr & Catenazzi, 2010	Lehr & Catenazzi, 2010	Lehr & Catenazzi, 2010
	Bryophryne gymnotis	Lehr & Catenazzi, 2010	Lehr & Catenazzi, 2010	Lehr & Catenazzi, 2010
	Bryophryne hanssaueri	Lehr & Catenazzi, 2009	Lehr & Catenazzi, 2009	----
	Bryophryne nubilosus	Lehr & Catenazzi, 2008	Lehr & Catenazzi, 2008	----
	Bryophryne zonalis	Lehr & Catenazzi, 2009	Lehr & Catenazzi, 2009	----

Craugastoridae (cont.)	*Euparkerella cochranae*	----	----	Hedges *et al.*, 2008
	Euparkerella cryptica	----	----	Hedges *et al.*, 2008
	Euparkerella robusta	----	----	Hedges *et al.*, 2008
	Euparkerella tridactyla	----	----	Hedges *et al.*, 2008
	Hypodactylus nigrovittatus	----	----	Lynch, 1971
Cycloramphidae	*Cycloramphus asper*	Verdade, 2005	----	Lynch, 1971
	Cycloramphus dubius	----	----	Lynch, 1971
	Cycloramphus granulosus	----	----	Lynch, 1971
	Cycloramphus ohausi	Verdade, 2005	----	Lynch, 1971
	Cycloramphus stejnegeri	Verdade, 2005	----	Lynch, 1971
	Thoropa lutzi	----	----	Lynch, 1971
	Thoropa petropolitana	----	----	Lynch, 1971
Eleutherodactylidae	*Eleutherodactylus albolabris*	----	----	Lynch, 1971
	Eleutherodactylus grandis	----	----	Lynch, 1971
	Eleutherodactylus guttilatus	----	----	Lynch, 1971
	Eleutherodactylus karlschmidti	----	----	Lynch, 1971
	Eleutherodactylus leprus	----	----	Lynch, 1971
	Eleutherodactylus pallidus	----	----	Lynch, 1971
	Eleutherodactylus rubrimaculatus	----	----	Lynch, 1971
	Phrynopus montium	----	----	Lynch, 1971
	Pristimantis festae	----	----	Lynch, 1971
	Pristimantis ornatissimus	----	----	Lynch, 1971
	Pristimantis variabilis	----	----	Lynch, 1971
Hemiphractidae	*Cryptobatrachus conditus*	----	----	Duellman, 2015
	Cryptobatrachus fuhrmanni	----	----	Duellman & Hoogmoed, 1984
	Fritziana fissilis	----	----	Folly *et al.*, 2014
	Fritziana goeldii	----	----	Folly *et al.*, 2014
	Fritziana ohausi	----	----	Folly *et al.*, 2014
	Fritziana ulei	----	----	Folly *et al.*, 2014

Hylidae	*Duellmanohyla uranochroa*	----	Boulenger, 1882	Duellman, 1970a
	Hyla hallowellii	----	----	Gaudin, 1974
	Hyloscirtus bogotensis	Lynch & Renjifo, 2001	Lynch & Renjifo, 2001	----
Hylodidae	*Crossodactylus dispar*	----	----	Lynch, 1971
	Crossodactylus grandis	----	----	Lynch, 1971
	Crossodactylus gaudichaudii	----	----	Lynch, 1971
	Hylodes asper	----	----	Lynch, 1971
	Hylodes dactylocinus	Pavan *et al.*, 2001	Pavan *et al.*, 2001	----
	Hylodes glaber	----	----	Lynch, 1971
	Hylodes lateristrigatus	----	----	Lynch, 1971
	Hylodes magalhaesi	----	----	Lynch, 1971
	Hylodes nasus	----	----	Lynch, 1971
	Hylodes perplicatus	----	Miranda Ribero, 1926	----
	Hylodes phyllodes	Heyer & Cocroft, 1986	Heyer & Cocroft, 1986	----
Hyperoliidae	*Callixalus pictus*	----	Drewes, 1984	Drewes, 1984
	Hyperolius horstockii	Boulenger, 1882; du Preez & Carruthers, 2009	----	----
	Kassinula wittei	----	Drewes, 1984	Drewes, 1984
Leptodactylidae	*Crossodactylodes bokermanni*	Barata *et al.*, 2013	----	Gomes, 1988
	Crossodactylodes izecksohni	Barata *et al.*, 2013	----	Gomes, 1988
	Crossodactylodes pintoi	Barata *et al.*, 2013	----	Lynch, 1971
	Crossodactylodes septentrionalis	Teixeira *et al.*, 2013	Teixeira *et al.*, 2013	Teixeira *et al.*, 2013
	Hydrolaetare schmidti	Cochran & Goin, 1959	Cochran & Goin, 1959	Lynch, 1971
	Leptodactylus latinasus	DB pers. obs.	DB pers. obs.; Ponssa, 2008	Lynch, 1971; Ponssa, 2008
	Leptodactylus macrosternum	----	----	Lynch, 1971
	Leptodactylus poecilochilus	----	Boulenger, 1882	Lynch, 1971
	Leptodactylus pustulatus	----	----	Lynch, 1971
	Paratelmatobius lutzii	Barata *et al.*, 2013	----	Lynch, 1971

Leptodactylidae (cont.)	*Paratelmatobius mantiqueira*	----	Pombal & Haddad, 1999	Pombal & Haddad, 1999
	Paratelmatobius yepiranga	Garcia *et al.*, 2009	Garcia *et al.*, 2009	Garcia *et al.*, 2009
	Physalaemus ephippifer	DB pers. obs.	DB pers. obs.	Lynch, 1971
	Physalaemus maculiventris	Heyer *et al.*, 1990	----	Lynch, 1971
	Physalaemus nanus	----	----	Lynch, 1971
	Pseudopaludicola boliviana	DB pers. obs.	DB pers. obs.	Lynch, 1971
	Pseudopaludicola saltica	DB pers. obs.	DB pers. obs.	Lynch, 1971
	Pseudopaludicola pusilla	----	----	Lynch, 1971
	Rupirana cardosoi	----	----	Heyer, 1999
Limnodynastidae	*Heleioporus albopunctatus*	----	----	Lynch, 1971
	Heleioporus eyrei	----	----	Lynch, 1971
	Notaden nichollsi	Nussbaum & Wu, 2007	Nussbaum & Wu, 2007	Lynch, 1971; Nussbaum & Wu, 2007
	Philoria frosti	----	----	Lynch, 1971
Mantellidae	*Boophis rhodoscelis*	----	----	Laurent, 1943
	Tsingymantis antitra	Glaw & Vences, 2007	Glaw & Vences, 2007	----
Megophryidae	*Leptobrachella mjobergi*	Inger & Stuebing, 1997	Inger & Stuebing, 1997	----
Microhylidae	*Adelastes hylonomus*	----	Zweiffel, 1986	Zweiffel, 1986
	Arcovomer passarelii	MT pers. obs.	MT pers. obs.	MT pers. obs.
	Callulops glandulosus	Zweifel, 1972	Zweifel, 1972	Zweifel, 1972
	Chiasmocleis avilapiresae	MT pers. obs.	MT pers. obs.	MT pers. obs.
	Chiasmocleis capixaba	MT pers. obs.	MT pers. obs.	MT pers. obs.
	Chiasmocleis carvalhoi	MT pers. obs.	MT pers. obs.	MT pers. obs.
	Chiasmocleis leucosticta	MT pers. obs.	MT pers. obs.	MT pers. obs.
	Chiasmocleis schubarti	MT pers. obs.	MT pers. obs.	MT pers. obs.
	Cophixalus saxatilis	----	----	Zweifel, 1985
	Ctenophryne aterrima	MT pers. obs.	MT pers. obs.	MT pers. obs.
	Myersiella microps	MT pers. obs.	MT pers. obs.	MT pers. obs.
	Otophryne pyburni	----	----	Parker, 1934

Microhylidae (cont.)	*Otophryne steyermarki*	Kok & Kalamandeen, 2008	Kok & Kalamandeen, 2008	----
	Oxydactyla coggeri	----	----	Zweiffel, 2000
	Stereocyclops histrio	MT pers. obs.	MT pers. obs.	MT pers. obs.
	Stereocyclops incrassatus	MT pers. obs.	MT pers. obs.	MT pers. obs.
	Stereocyclops parkeri	MT pers. obs.	MT pers. obs.	MT pers. obs.
	Uperodon mormorata	----	Chandramouli & Dutta, 2015	Chandramouli & Dutta, 2015
Myobatrachidae	*Arenophryne rotunda*	----	----	Davies, 1984
	Crinia bilingua	----	----	Davies, 1984
	Crinia georgiana	----	Heyer & Liem, 1976	Heyer & Liem, 1976
	Crinia glauerti	----	----	Davies, 1984
	Crinia insignifera	----	----	Davies, 1984
	Crinia tasmaniensis	----	----	Davies, 1984
	Crinia subinsignifera	----	----	Davies, 1984
	Pseudophryne australis	----	----	Heyer & Liem, 1976
	Pseudophryne corroboree	----	----	Lynch, 1971; Heyer & Liem, 1976,
	Pseudophryne dendyi	----	----	Heyer & Liem, 1976
	Pseudophryne douglasi	----	----	Heyer & Liem, 1976
	Pseudophryne guentheri	----	----	Heyer & Liem, 1976,
	Pseudophryne major	----	----	Heyer & Liem, 1976
	Pseudophryne occidentalis	----	----	Heyer & Liem, 1976
	Pseudophryne semimarmorata	Loftus-Hill, 1973	Loftus-Hill, 1973	Loftus-Hill, 1973; Heyer & Liem, 1976
	Taudactylus diurnus	Nussbaum & Wu, 2007	Nussbaum & Wu, 2007	Nussbaum & Wu, 2007; Perry, 2004
Odontobatrachidae	*Odontobatrachus natator*	Scott, 2005	Scott, 2005; Barej *et al.*, 2014	Scott, 2005; Barej *et al.*, 2014
Phrynobatrachidae	*Phrynobatrachus plicatus*	Scott, 2005	Scott, 2005	Scott, 2005

Pyxicephalidae	*Anhydrophryne hewitti*	Scott, 2005	Scott, 2005	Scott, 2005
	Arthroleptella lightfooti	Scott, 2005	Scott, 2005	Scott, 2005
	Ericabatrachus baleensis	----	Scott, 2005	Scott, 2005
	Nothophryne broadleyi	Scott, 2005	Scott, 2005	Scott, 2005
Ranidae	*Hydrophylax gracilis*	Biju *et al.*, 2014b	Biju *et al.*, 2014b	----
	Indosylvirana temporalis	Biju *et al.*, 2014b	Biju *et al.*, 2014b	----
	Indosylvirana flavescens	----	Chandramouli & Dutta, 2015	Chandramouli & Dutta, 2015
Ranixalidae	*Indirana temporalis*	----	Boulenger, 1882	Boulenger, 1882
Rhacophoridae	*Pseudophilautus silus*	----	----	Kernet *et al.*, 2007
	Theloderma pictum	----	----	Liem, 1970
Telmatobiidae	*Telmatobius brachydactylus*	----	----	Lynch, 1978
	Telmatobius laticeps	----	----	Lynch, 1971
	Telmatobius macrostomus	----	----	Lynch, 1971
Incertae sedis (Brachycephaloidea)	*Atopophrynus syntomopus*	----	----	Myers & Ford, 1986
	Geobatrachus walkeri	Ardila-Robayo, 1979	Ardila-Robayo, 1979	Ardila-Robayo, 1979

1.5—References

Acosta-Galvis A. R., Rueda-Almonacid, J. V., Velásquez-Alvarez, Á. A., Sánchez-Pacheco, S. J. & Peña Prieto, J. A. (2006). Descubrimiento de una nueva especie de *Atelopus* (Bufonidae) para Colombia: ¿Una luz de esperanza o el ocaso de los sapos arlequines? *Revista de la Academia Colombiana de Ciencias Exactas, Físicas y Naturales* **30**, 279–290.

Aguayo-Vedia C.R. & Harvey, M.B. (2001). Dos nuevas especies de *Phrynopus* (Anura: Leptodactylidae) de los bosques nublados de Bolivia. *Revista de Biología Tropical* **49**, 333–345.

Alves A. C. R., Ribeiro, L. F., Haddad, C. F. B. & Reis, S. F. d. (2006). Two new species of *Brachycephalus* (Anura: Brachycephalidae) from the Atlantic Forest in Paraná state, Southern Brazil. *Herpetologica* **62**, 221–233.

Alves A. C. R., Sawaya, R. J., dos Reis, S. F. & Haddad, C. F. B. (2009). New species of Brachycephalus (Anura: Brachycephalidae) from the Atlantic Rain Forest in São Paulo state, southeastern Brazil. *Journal of Herpetology* **43**, 212–219.

Amiet J.-L. (1980). Revision du genre *Leptodactylodon* Andersson (Amphibia, Anura, Astylosterninae). *Annales de la Faculté des Sciences du Yaoundé* **27**, 69–224.

Amiet J.-L. (2012). *Les rainettes de Cameroun (Amphibiens anoures)*. La Nef des Livres, Nyons.

Anderson J. (1893). On a new species of *Zamenis* and a new species of *Bufo* from Egypt. *Annals and Magazine of Natural History* **Series 6**, 439–440.

Andersson L. G. (1903). Neue Batrachier aus Kamerun, von den Herren Dr. Y. Sjostedt und Dr. R. Jungner gesammelt (vorlaufige Mitteilung). *Verhandlungen des Zoologisch-Botanischen Vereins in Wien* **53**, 141–145.

Andren C. & Nilson, G. (1979). A new species of toad (Amphibia, Anura, Bufonidae) from the Kavir Desert, Iran. *Journal of Herpetology* **13**, 93–100.

Annandale N. (1912). Zoological results of the Abor Expedition, 1911–1912. I. *Amphibia. Records of the Indian Museum* **8**, 7–36.

Anstis M. (2013). *Tadpoles and frogs of Australia*. (New Holland Publishers).

Arch V. S., Richards-Zawaki, C. L. & Feng, A. S. (2011). Acoustic communication in the Kihansi spray toad (*Nectophrynoides asperginis*): insights from a captive population. *Journal of Herpetology* **45**, 45–49.

Ardila-Robayo M. C. & Ruiz-Carranza, P. M. (1998). Una nueva especie de *Atelopus* A.M.C. Dumeril & Bibron 1841 (Amphibia: Bufonidae) de la Cordillera Central colombiana. *Revista de la Academia Colombiana de Ciencias Exactas, Físicas y Naturales* **22**, 281–285.

Ardila-Robayo M. C. (1979). Status sistemático del género *Geobatrachus* Ruthven 1915 (Amphibia: Anura). *Caldasia* 12, 383–495.

Ardila-Robayo M. C. (1999). Una nueva especie de *Atelopus* A.M.C. Dumeril & Bibron 1841 (Amphibia: Anura: Bufonidae) de la Cordillera Oriental colombiana. *Revista de la Academia Colombiana de Ciencias Exactas, Físicas y Naturales* **23**, 139–142.

Ardila-Robayo M. C., Osorno-Muñoz, M. & Ruiz-Carranza, P. M. (2002). Una nueva especie del género *Atelopus* A.M.C. Dumeril & Bibron 1841 (Amphibia: Bufonidae) de la Cordillera Oriental colombiana. *Revista de la Academia Colombiana de Ciencias Exactas, Físicas y Naturales* **26**, 133–139.

Ávila R. W., Carvalho, V. T., Gordo, M., Kawashita-Ribeiro, R. A. & Morais, D. H. (2012). A new species of *Amazophrynella* (Anura: Bufonidae) from southern Amazonia. *Zootaxa* **3484**, 65–74.

Ávila R. W., Pansonato, A. & Strüssmann, C. (2010). A new species of the *Rhinella margaritifera* group (Anura: Bufonidae) from Brazilian Pantanal. *Zootaxa* **2339**, 57–68.

Avilán P. & Hoyos, J. (2006). Osteology of *Eleutherodactylus bogotensis* (Amphibia, Anura, Leptodactylidae) from the Parque Nacional Natural Chingaza (Cundinamarca, Colombia). *Caldasia* **28**, 89–109.

Ayarzagüena J. & Señaris J. C. (1997) Dos nuevas especies de *Cochranella* (Anura; Centrolenidae) para Venezuela. *Publicaciones de la Asociación de Amigos de Doñana* **8**, 1–16.

Ayarzagüena J. & Señaris, J. C. (1993). Dos nuevas especies de *Hyla* (Anura; Hylidae) para las Cumbres Tepuyanas del Estado Amazonas, Venezuela. *Memoria de la Sociedad de Ciencias Naturales La Salle* **53**, 127–146.

Ayarzagüena J., Señaris, J. C. & Gorzula, S. (1992). El grupo *Osteocephalus rodriguezi* de las tierras altas de la guayana venezolana: descripcion de cinco nuevas especies. *Memoria Sociedad de Ciencias Naturales La Salle* **52**, 133-142.

Badenhorst C. E. (1945). Die Skedelmorfologie van die Neotropiese Anure *Atelopus moreirae* de Mirando-Ribeiro. *Annals of the University of Stellenbosch* **23**, 1–19.

Baha El Din S. M. (1993). A new species of toad (Anura: Bufonidae) from Egypt. *The Journal of the Herpetological Association of Africa* **42**, 24–27.

Bain R. H., Lathrop A., Murphy R. W., Orlov N. L. & Cuc H. T. (2003) Cryptic species of a cascade frog from Southeast Asia: taxonomic revisions and descriptions of six new species. *American Museum Novitates* **3417**, 1-60.

Baldauf R. J. (1955). Contributions to the cranial morphology of *Bufo w. woodhousei* Girad. *The Texas Journal of Science* **7**, 275–311.

Baldauf R. J. (1959). Morphological criteria and their use in showing bufonid phylogeny. *Journal of morphology* **104**, 527–560.

Baldissera F. A., Jr., Caramaschi, U. & Haddad, C. F. B. (2004). Review of the *Bufo crucifer* species group, with descriptions of two new related species (Amphibia, Anura, Bufonidae). *Arquivos do Museu Nacional do Rio de Janeiro* **62**, 255–282.

Baldo D. & Basso, N. G. (2004). New species of *Melanophryniscus* Gallardo, 1961 (Anura: Bufonidae), with comments on the species of the genus reported for Misiones, Northeastern Argentina. *Journal of Herpetology* **38**, 393–403.

Baldo D., Borteiro, C., Kolenc, F., Rosset, S., Prigioni, C. & Martínez Debat, C. (2012). The taxonomic status of *Melanophryniscus orejasmirandai* Prigioni & Langone, 1987 "1986" (Anura: Bufonidae). *Zootaxa* **3235**, 45–61.

Barata I. M., Santos, M., Leite, F. & Garcia, P. (2013). A new species of *Crossodactylodes* (Anura: Leptodactylidae) from Minas Gerais, Brazil: first record of genus within the Espinhaco Mountain Range. *Zootaxa* **3731**, 552–560.

Barbour T. (1910). Notes on the Herpetology of Jamaica. *Bulletin of the Museum of Comparative Zoology* **52**, 273–301.

Barbour T. (1914). A contribution to the zoogeography of the West Indies, with special reference to Amphibians and Reptiles. *Memoirs of the Museum of Comparative Zoology* **44**, 205–359.

Barbour T. & Shreve, B. (1937). Novitates cubanae. *Bulletin of the Museum of Comparative Zoology* **80**, 377–387.

Barej M. F., Schmitz, A., Günther, R., Loader, S. P., Mahlow, K. & Rödel, M.-O. (2014). The first endemic West African vertebrate family–a new anuran family highlighting the uniqueness of the Upper Guinean biodiversity hotspot. *Frontiers in zoology* **11**, 8[1–10].

Barej M. F., Schmitz, A., Menegon, M., Hillers, A., Hinkel, H., Böhme, W. & Rödel, M.-O. (2011). Dusted off—the African *Amietophrynus superciliaris*-species complex of giant toads. *Zootaxa* **2772**, 1–32.

Barker J., Grigg, G. & Tyler, M. (1995). *A field guide to Australian frogs*. Surrey Beatty & Sons, Chipping Norton, Australia.

Barrera-Rodríguez M. (2000) Estudio Anatómico de cuatro especies de ranitas de cristal del género *Hyalinobatrachium* Ruíz and Lynch 1991 grupo *fleischmanni* (Amphibia: Anura: Centrolenidae). *Revista de la Academia Colombiana de Ciencias Exactas, Físicas y Naturales* **23**, 245–60.

Barrera G. S. & Rodríguez J. P. (2004) Status of three species of toads in North-western Mexico. Sonoran Herpetologist 17, 74–76.

Barrio A. (1962). Los Hylidae de Punta Lara, provincia de Buenos Aires. *Physis* **23**, 129–142.

Barrio A. (1965). La subespecies de *Hyla pulchella* Duméril y Bibron (Anura, Hylidae). *Physis* **25**, 115-128.

Barrio A. (1970). *Insuetophrynus acarpicus,* un nuevo leptodactylido firmisternio sudamericano (Amphibia, Anura). *Physis* **30**, 331–341.

Barrio-Amoros C. L. (2006) A new species of *Phyllomedusa* (Anura: Hylidae: Phyllomedusinae) from northwestern Venezuela. *Zootaxa* **1309**, 55–68.

Basso N. G., Ubeda, C. A., Bunge, M. M. & Martinazzo, L. B. (2011). A new genus of neobatrachian frog from southern Patagonian forests, Argentina and Chile. *Zootaxa* **3002**, 31–44.

Beerli P., Hotz H., Tunner H. G., Heppich S. & Uzzell T. (1994) Two new water frog species from the Aegean islands Crete and Karpathos (Amphibia, Salientia, Ranidae). *Notulae naturae* **470**, 1–9.

Beirne C. & Whitworth. (2011). *Frogs of the Yachana reserve*. Global Vision International.

Beukema W., De Pous, P., Donaire-Barroso, D., Bogaerts, S., Garcia-Porta, J., Escoriza, D., Arribas, O. J., El Mouden, E. H. & Carranza, S. (2013). Review of the systematics, distribution, biogeography and natural history of Moroccan amphibians. Zootaxa **3661**, 1–60.

Biju S. & Bossuyt F. (2009) Systematics and phylogeny of *Philautus* Gistel, 1848 (Anura, Rhacophoridae) in the Western Ghats of India, with descriptions of 12 new species. *Zoological Journal of the Linnean Society* **155**, 374–444.

Biju S. D. & Bossuyt, F. (2003). New frog family from India reveals an ancient biogeographical link with the Seychelles. *Nature* **425**, 711–714.

Biju S. D., Garg, S., Gururaja, K. V., Shouche, Y. & Walujkar, S. A. (2014a) DNA barcoding reveals unprecedented diversity in Dancing Frogs of India (Micrixalidae, *Micrixalus*): a taxonomic revision with description of 14 new species. *Ceylon Journal of Science* **43**, 37–123.

Biju S. D., Garg, S., Mahony, S., Wijayathilaka, N., Senevirathne, G. & Meegaskumbura, M. (2014b). DNA barcoding, phylogeny and systematics of Golden-backed frogs (*Hylarana*, Ranidae) of the Western Ghats-Sri Lanka biodiversity hotspot, with the description of seven new species. Contributions to Zoology **83**, 269–335.

Biju S. D., van Bocxlaer, I., Giri, V. B., Loader, S. P. & Bossuyt, F. (2009). Two new endemic genera and a new species of toad (Anura: Bufonidae) from the Western Ghats of India. *BMC Research Notes* **2**.

Biju S. D., van Bocxlaer, I., Giri, V. B., Roelants, K., Nagaraju, J. & Bossuyt, F. (2007). A new rightfrog, *Nyctibatrachus minimus* sp. nov.(Anura: Nyctibatrachidae): the smallest frog from India. *Current Science* **93**, 854–859.

Blackburn D. C. (2008). A new species of *Cardioglossa* (Amphibia: Anura: Arthroleptidae) endemic to Mount Manengouba in the Republic of Cameroon, with an analysis of morphological diversity in the genus. *Zoological Journal of the Linnean Society* **154**, 611–630.

Blake A. J. D. (1973). Taxonomy and relationships of myobatrachine frogs (Leptodactylidae): a numerical approach. *Australian Journal of Zoology* **21**, 119–149.

Boettger O. (1880). Neue Krötenvarietät von den Balearen. *Zoologischer Anzeiger* **3**, 642–643.

Boettger O. (1895). Neue Frösche und Schlangen von den Liukiu-Inseln. *Zoologischer Anzeiger* **18**, 266–270

Bogert C. M. & Senanayake, R. (1966). A new species of toad (*Bufo*) indigenous to southern Ceylon. *American Museum Novitates* **2269**, 1–18.

Boistel R. & Amiet, J.-L. (2001). Une nouvelle espèce de *Wolterstorffina* (Amphibia, Anura, Bufonidae) de l'étage afro-subalpin du Mont Okou (Cameroun). *Alytes* **18**, 127–140.

Boistel R. & de Massary, J.-C. (1999). Les amphibiens vénéneux de la famille des dendrobatidés. *Le Courrier de la nature* **176**, 34–37.

Boistel R., Aubin, T., Cloetens, P., Langer, M., Gillet, B., Josset, P., Pollet, N. & Herrel, A. (2011). Whispering to the deaf: communication by a frog without external vocal sac or tympanum in noisy environments. *PloS one* **6**, e22080.

Boistel R., Aubin, T., Cloetens, P., Peyrin, F., Scotti, T., Herzog, P., Gerlach, J., Pollet, N. & Aubry, J.-F. (2013). How minute sooglossid frogs hear without a middle ear. *Proceedings of the National Academy of Sciences* **110**, 15360–15364.

Boistel, R., de Massary, J.-C. & Angulo, A. (2006) Description of a new species of the genus *Adenomera* (Amphibia, Anura, Leptodactylidae) from French Guiana. *Acta Herpetologica* **1**, 1-14.

Bokermann W. C. A. (1964a). Dos nuevas especies de *Hyla* de Minas Gerais y notas sobre *Hyla alvarengai* Bok. (Amphibia, Salientia, Hylidae). *Neotropica* **10**, 67-76.

Bokermann W. C. A. (1964b). Notes on treefrogs of the *Hyla marmorata* group with description of a new species (Amphibia, Hylidae). *Senckenbergiana Biologica* **45**, 243–254.

Bokermann W. C. A. (1965) Tres novos batraquios da regiao central de Mato Grosso, Brasil. *Revista Brasileira de Biologia* **25**, 257–264.

Bokermann W. C. A. (1966). A new *Phyllomedusa* from southeastern Brazil. *Herpetologica* **22**, 293–297.

Bokermann W. C. A. (1967a). *Hyla astartea*, nova especie da Serra do Mar em Sao Paulo (Amphibia, Hylidae). *Revista Brasileira de Biologia* **27**, 157–158.

Bokermann W. C. A. (1967B). Nova especie de *Hyla* do Amapa (Amphibia, Hylidae). *Revista Brasileira de Biologia* **27**, 109-112.

Bokermann W. C. A. (1967c). Una nueva especie do *Elosia* de Itatiaia, Brasil (Amphibia, Leptodactylidae). *Neotropica* **13**, 135–138.

Bokermann W. C. A. (1975). Três espécies novas de *Eleutherodactylus* do sudeste da Bahia, Brasil (Anura, Leptodactylidae). *Revista Brasileira de Biología* **34**, 11–18.

Bokermann W. C. A. & Sazima, I. (1978). Anfíbios da Serra do Cipó, Minas Gerais, Brasil. 4: Descrição de *Phyllomedusa jandaia* sp. n. (Anura, Hylidae). *Revista Brasileira de Biologia* **38**, 927-930.

Bornschein M. R., Firkowski, C. R., Baldo, D., Ribeiro, L. F., Belmonte-Lopes, R., Corrêa, L., Morato, S. A. A. & Pie, M. R. (2015). Three new species of phytotelm-breeding *Melanophryniscus* from the Atlantic Rainforest of southern Brazil (Anura: Bufonidae). *Plos One* **10**, e0142791.

Boulenger E.G. (1915). On two new tree'frogs from Sierra Leone, recently living in the Society´s gardens. *Proc. Zool. Soc. London* **1915**, 243.

Boulenger G. A. (1882). *Catalogue of the Batrachia Salientia s. Ecaudata in the Collection of the British Museum. Second Edition*. Taylor and Francis, London.

Boulenger G. A. (1887). Description of a new frog of the genus *Megalophrys*. *Annali del Museo Civico di Storia Naturale di Genova, Ser. 2* **4**, 512–513.

Boulenger G. A. (1888). On some Reptiles and Batrachians from Iguarasse, Pernambuco. *Journal of Natural History* **2**, 40–43.

Boulenger G. A. (1890). *Fauna of British India, including Ceylon and Burma*, London.

Boulenger G. A. (1895a). An account of the reptiles and batrachians collected by Dr. A. Donaldson Smith in western Somaliland and the Galla country. In *Proceedings of the Zoological Society of London*, vol. 63, pp. 530–540.

Boulenger G. A. (1895b). Description of a new batrachian (*Oreophryne quelchii*) discovered by Messrs. J. J. Quelch and F. McConnell on the Summit of Mount Roraima. *Annals and Magazine of Natural History Ser. 6* **15**, 521–522.

Boulenger G. A. (1897). *The Tailless Batrachians of Europe. Parts I & II.* Ray Society, London.

Boulenger G. A. (1898). Fourth report on additions to the Batrachian collection in the Natural-History Museum. *Proceedings of the Zoological Society of London* **1898**, 473–482, 2 plates.

Boulenger G. A. (1899). Descriptions of new batrachians in the collection of the British Museum (Natural History). *Annals and Magazine of Natural History* **ser. 7**, **3**, 273–277, 2 plates.

Boulenger G. A. (1900a). Batrachians. In E. R. Lankester, Report on a collection made by Messrs. F. V. McConnell and J. J. Quelch at Mount Roraima in British Guiana. **8**, 55–56.

Boulenger G. A. (1900b). Descriptions of new batrachians and reptiles collected by Mr. PO Simons in Peru. *Journal of Natural History* **6**, 181–186.

Boulenger G. A. (1900c). A list of the batrachians and reptiles of the Gaboon (French Congo), with descriptions of new genera and species. *Proceedings of the Zoological Society of London* **1900**, 433–456.

Boulenger G. A. (1902). Descriptions of new batrachians and reptiles from the Andes of Peru and Bolivia. *Journal of Natural History* **10**, 394–402.

Boulenger G. A. (1903a). Batraciens de la Guinée espagnole. *Memorias de la Real Sociedad Española de Historia Natural* **1**, 61–64.

Boulenger G. A. (1903b). Descriptions of new batrachians in the British Museum. *Annals and Magazine of Natural History* **ser. 7**, **12**, 552–557.

Boulenger G. A. (1913). On the presence of two closely allied species of toads of the genus *Nectophryne* in Cameroon. *Annals and Magazine of Natural History, Ser. 8* **12**, 70–72.

Boulenger G. A. (1919). Descriptions of three new batrachians from the Goro Hills, Assam. *Records of the Indian Museum* **16**, 207–208.

Boulenger G. A. (1920) A monograph of the South Asian, Papuan, Melanesian and Australian frogs of the genus *Rana*. *Records of the Indian Museum* **20**, 1–226.

Brandão R. A. (2002). A new species of *Phyllomedusa* Wagler, 1830 (Anura: Hylidae) from central Brazil. *Journal of Herpetology* **36**, 571–578.

Brandão R. A., Maciel, N. M. & Sebben, A. (2007). A new species of *Chaunus* from Central Brazil (Anura; Bufonidae). *Journal of Herpetology* **41**, 309–316.

Brasileiro C. A., Oyamaguchi H. M. & Haddad C. F. (2007) A new island species of Scinax (Anura; Hylidae) from southeastern Brazil. *Journal of Herpetology* **41**, 271-5.

Braun P. C. & Braun, C. A. S. (1977). Nova espécie de *Hyla* do estado do Rio Grande do Sul, Brasil (Anura, Hylidae). *Revista Brasileira de Biologia* **37**, 853–857.

Bravo-Valencia L. & Rivera-Correa, M. (2011). A new species of harlequin frog (Bufonidae: *Atelopus*) with an unusual behavior from Andes of Colombia. *Zootaxa* **3045**, 57–67.

Brocchi M. P. (1879). Sur divers batraciens anoures de l'Amérique Centrale. *Société Philomathique de Paris* **(7)3**, 19–24.

Brocchi P. (1877). Sur quelques batraciens raniformes et bufoniformes de l'Amérique Centrale. *Société Philomathique de Paris* **7**, 175–197.

Brown J. L., Twomey E., Amezquita A., Souza M. B., Caldwell J. P., Loetters S., von May R., Melo-Sampaio P. R., Mejia-Vargas D., Perez-Pena P., Pepper M., Poelman E. H., Sanckez-Rodriguez M. & Summers K. (2011) A taxonomic revision of the Neotropical poison frog genus *Ranitomeya* (Amphibia: Dendrobatidae). *Zootaxa* **3083**, 1–120.

Caldwell J. P. (1991). A new species of toad in the genus *Bufo* from Pará, Brazil, with an unusual breeding site. *Papéis Avulsos de Zoologia* **37**, 389–400.

Caldwell J.P. (2005). A new Amazonian species of *Cryptophyllobates* (Anura: Dendrobatidae). *Herpetologica* **61**, 449–461.

Caminer M. A. & Ron, S. R. (2014) Systematics of treefrogs of the *Hypsiboas calcaratus* and *Hypsiboas fasciatus* species complex (Anura, Hylidae) with the description of four new species. *ZooKeys* **370**, 1–68.

Camp C. L. (1915). *Batrachoseps major* and *Bufo cognatus californicus*, new Amphibia from southern California. *University of California Publications in Zoology* **12**, 327–334.

Camp C. L. (1916). Description of *Bufo canorus*, a New toad from the Yosemite National Park. *University of California Publications in Zoology* **17**, 59–62.

Campbell J. A. & Savage, J. M. (2000). Taxonomic reconsideration of Middle American frogs of the *Eleutherodactylus rugulosus* group (Anura: Leptodactylidae): a reconnaissance of subtle nuances among frogs. *Herpetological Monographs* **14**, 186–292.

Cannatella D. C. (1980) Two new species of *Centrolenella* from Bolivia (Anura: Centrolenidae). *Proceedings of the Biological Society of Washington* **93**, 714–24.

Cannatella D. C. (1981). A New *Atelopus* from Ecuador and Colombia. *Journal of Herpetology* **15**, 133–138.

Cannatella D. C. (1982). Leaf–frogs of the *Phyllomedusa perinesos* group (Anura: Hylidae). Copeia **1982**, 501–513.

Cannatella D. C. (1984). Two new species of the leptodactylid frog genus *Phrynopus*, with comments on the phylogeny of the genus. *Occasional Papers of the Museum of Natural History, The University of Kansas* **113**, 1–16.

Cannatella D. C. (1986). A new genus of bufonid (Anura) from South America, and phylogenetic relationships of the neotropical genera. *Herpetologica* **42**, 197–205.

Cannatella D. C. & Trueb, L. (1988). Evolution of pipoid frogs: Intergeneric relationships of the aquatic frog family Pipidae (Anura). *Zoological Journal of the Linnean Society* **94**, 1–38.

Canseco-Márquez L., Mendelson, J. R., Gutiérrez–Mayén, G. (2002). A new species of *Hyla* (Anura: Hylidae) from the Mixteca Alta, Oaxaca, Mexico. *Herpetologica* **58**, 260–269.

Caramaschi U. (2006). Redefinição do grupo de *Phyllomedusa hypochondrialis*, com redescrição de *P. megacephala* (Miranda–Ribeiro, 1926), revalidação de *P. azurea* Cope, 1862 e descrição de uma nova espécie (Amphibia, Anura, Hylidae). *Arquivos do Museu Nacional, Rio de Janeiro* **64**, 159–179.

Caramaschi U. (2012). New species of beaked toad, *Rhinella* (Anura: Bufonidae), from the State of Bahia, Brazil. *Zoologia (Curitiba)* **29**, 343–348.

Caramaschi U. & Cruz, C. A. G. (2004). Duas novas espécies de *Hyla* do grupo de *H. polytaenia* Cope, 1870 do sudeste do Brasil (Amphibia, Anura, Hylidae). *Arquivos do Museu Nacional. Rio de Janeiro* **62**, 247–254.

Caramaschi U. & Cruz, C. A. G. (1998). Notas taxonômicas sobre *Pseudis fusca* Garman e *P. bolboactyla* A. Lutz, com a descrição de uma nova especie correlata (Anura, Pseudidae). *Revista Brasileira de Biologia* **15**, 929–944.

Caramaschi U. & Cruz, C. A. G. (2000). Duas espécies novas de *Hyla* Laurenti, 1768 do estado de Goiás, Brasil (Amphibia, Anura, Hylidae). *Boletim do Museu Nacional, Nova Série, Zoologia* **422**, 1–12.

Caramaschi U. & Cruz, C. A. G. (2002). Taxonomic status of *Atelopus pachyrhynus* Miranda-Ribeiro, 1920, redescription of *Melanophryniscus tumifrons* (Boulenger, 1905), and description of two new species of *Melanophryniscus* from the state of Santa Catarina, Brazil (Amphibia, Anura, Bufonidae). *Arquivos do Museu Nacional do Rio de Janeiro* **60**, 303–314.

Caramaschi U. & Cruz, C. A. G. (2011). A new, possibly threatened species of *Melanophryniscus* Gallardo, 1961 from the state of Mina Gerais, Southeastern Brazil (Amphibia, Anura, Bufonidae). *Boletim do Museu Nacional do Rio de Janeiro* **528**, 1–9.

Caramaschi U. & Kisteumacher, G. (1989). A new species of *Eleutherodactylus* (Anura: Leptodactylidae) from Minas Gerais, southeastern Brazil. *Herpetologica* **44**, 423–426.

Caramaschi U. & Niemeyer, H. (2003). Nova espécie do complexo de *Bufo margaritifer* (Laurenti, 1768) do estado do Mato Grosso do Sul, Brasil (Amphibia, Anura, Bufonidae). *Boletim do Museu Nacional do Rio de Janeiro* **501**, 1–16.

Caramaschi U. & Pombal, J. P. (2006). A new species of *Rhinella* Fitzinger, 1826 from the Atlantic Rain Forest, eastern Brazil (Amphibia, Anura, Bufonidae). *Papéis Avulsos de Zoologia* **46**, 251–259.

Carrizo G. R. (1990). Sobre los hilidos de Misiones, con la descripción de una nueva especie, *Hyla caingua* n.sp. (Anura, Hylidae). *Cuadernos de Herpetologia* **6**, 32–39.

Carrizo G. R. (1992). Cuatro especies nuevas de anuros (Bufonidae: *Bufo* e Hylidae: *Hyla*) del norte de la Argentina. *Cuadernos de herpetología* **7**, 14–23.

Carvalho-e-Silva A. M. P. T. & Carvalho-e-Silva, S. P. (2006). New species of the *Hyla albofrenata* group, from the states of Rio de Janeiro and São Paulo, Brazil (Anura, Hylidae). *Journal of Herpetology* **38**, 73–81.

Carvalho-e-Silva A. M. P. T., Mongin, M. M., Izecksohn, E. & Carvalhoe-Silva, S. P. (2010). A new species of *Dendrophryniscus* Jiménez–de–la–Espada from the Parque Nacional da Serra dos Órgãos, Teresópolis, State of Rio de Janeiro, Brazil (Amphibia, Anura, Bufonidae). *Zootaxa* **2632**, 46–52.

Carvalho-e-Silva A. M. P. T., Ramos Da Silva, G. & Carvalho-e-Silva, S. P. (2009). A new species of *Phasmahyla* Cruz, 1990 from the Atlantic Forest in the state of Rio de Janeiro, Brazil (Amphibia, Hylidae, Phyllomedusinae). *Zootaxa* **2120**, 15–26.

Castanho L. M. & Haddad, C. F. (2000). New species of *Eleutherodactylus* (Amphibia: Leptodactylidae) from Guaraqueçaba, Atlantic Forest of Brazil. *Copeia* **2000**, 777–781.

Castroviejo-Fisher S., Vila, C., Ayarzagueena, J., Blanc, M. & Ernst, R. (2011). Species diversity of *Hyalinobatrachium* glassfrogs (Amphibia: Centrolenidae) from the Guiana Shield, with the description of two new species. *Zootaxa* **3132**, 1–55.

Castroviejo-Fisher, S., Señaris, J. C., Ayarzagüena, J. & Vilà, C. (2008) Resurrection of *Hyalinobatrachium orocostale* and notes on the *Hyalinobatrachium orientale* species complex (Anura: Centrolenidae). *Herpetologica* **64**, 472–484.

Catenazzi A., Lehr, E. & von May, R. (2013). The amphibians and reptiles of Manu National Park and its buffer zone, Amazon basin and eastern slopes of the Andes, Peru. *Biota Neotropica* **13**, 269–283.

Cei J. M. (1972). Segregación corologica y procesos de especiación por aislamiento en anfibios de la Pampa de Achala, Córdoba. *Acta Zoológica Lilloana* **29**, 233–246.

Cei J. M. (1980). Amphibians of Argentina. *Monitore Zoologico Italiano* **2**, 1–609.

Céspedez J. A. (2008). Una nueva especie de *Melanophryniscus* Gallardo, 1961 de Argentina (Amphibia: Anura: Bufonidae). *FACENA* **24**, 35–48.

Céspedez J. A. & Alvarez, B. B. (2000). Una nueva especie de *Melanophryniscus* (Anura: Bufonidae) del grupo *stelzneri* de Corrientes, Argentina. *FACENA* **15**, 57–67.

Céspedez J. A. & Motte, M. (2007). Una nueva especie de *Melanophryniscus* Gallardo, 1961 de Paraguay (Amphibia: Anura: Bufonidae). *FACENA* **23**, 31–42.

Chabanaud P. (1919). Description d’une espèce nouvelle de batracien du Sénégal. *Bulletin du Museum National d'Histoire Naturelle. Paris* **25**, 454–455.

Chan K. O., Wodd Jr, P., Anuar, S., Muin, M. A., Quah, E. S., Sumaril, A. & Grismer, L. L. (2014). A new species of upland stream toad of the genus *Ansonia* Stoliczka, 1870 (Anura: Bufonidae) from northeastern Peninsular Malaysia. *Zootaxa* **3764**, 427–440.

Chandramouli S. R. & Dutta, S. K. (2015) Comparative osteology of anuran genera in the Western Ghats, Peninsular India. *Alytes* **32**, 67–81.

Chandramouli S. R., Vasudevan, K., Harikrishnan, S., Dutta, S. K., Janani, S. J., Sharma, R., Das, I. & Aggarwal, R. K. (2016). A new genus and species of arboreal toad with phytotelmonous larvae, from the Andaman Islands, India (Lissamphibia, Anura, Bufonidae). *ZooKeys* **555**, 57–90.

Channing A. & Stanley, W. T. (2002). A new tree toad from the Ukaguru Mountains, Tanzania. *African Journal of Herpetology* **51**, 121–128.

Channing A. & Vences, M. (1999). The advertisement call, breeding biology, description of the tadpole and taxonomic status of *Bufo dombensis*, a little-known dwarf toad from southern Africa. *South African Journal of Zoology* **34**, 74–79.

Chaparro J. C., Padial, J. M., Gutiérrez, R. C. & De la Riva, I. (2015). A new species of Andean frog of the genus *Bryophryne* from southern Peru (Anura: Craugastoridae) and its phylogenetic position, with notes on the diversity of the genus. *Zootaxa* **3994**, 94–108.

Chaparro J. C., Pramuk, J. B. & Gluesenkamp, A. G. (2007). A new species of arboreal *Rhinella* (Anura: Bufonidae) from cloud forest of southeastern Peru. *Herpetologica* **63**, 203–212.

Cisneros-Heredia D. F. & Gluesenkamp, A. G. (2010). A new andean toad of the genus *Osornophryne* (Amphibia: Anura: Bufonidae) from northwestern Ecuador, with taxonomic remarks on the genus. *Avances* **2**, B64–B73.

Cisneros-Heredia D. F. & McDiarmid, R. W. (2007). Revision of the characters of Centrolenidae (Amphibia: Anura: Athesphatanura), with comments on its taxonomy and the description of new taxa of glassfrogs. *Zootaxa* **1572**, 82 pp.

Cisneros-Heredia, D. F. & Morales-Mite, M. (2008) A new species of glassfrog from elfin forests of the Cordillera del Cóndor, southeastern Ecuador. *Herpetozoa* **21**, 49–56.

Clarke B. T. (1983). A morphological re-examination of the frog genus *Nannophrys* (Anura: Ranidae) with comments on its biology, distribution and relationships. *Zoological Journal of the Linnean Society* **79**, 377–398.

Clarke B. T. (1989). Real vs apparent distributions of dwarf amphibians: *Bufo lindneri* Mertens, 1955-a case in point. *Amphibia-Reptilia* **10**, 297–303.

Cochran D. M. (1923). A new species of *Eleutherodactylus* from the Dominican Republic. *Proceedings of the Biological Society of Washington* **36**, 93–94.

Cochran D. M. (1932). A new frog, *Eleutherodactylus wetmorei*, from the Republic of Haiti. *Proceedings of the Biological Society of Washington* **45**, 191–194.

Cochran D. M. (1935). New reptiles and amphibians collected in Haiti by PJ Darlington. *Proceedings of the Boston Society of Natural History* **40**, 367–376.

Cochran D. M. (1938). A new species of frog from Haiti. *Proceedings of the Biological Society of Washington* **51**, 93–94.

Cochran D. M. (1941). The herpetology of Hispaniola. *Bulletin of the United States National Museum*, 1–398.

Cochran D. M. (1955). Frogs of southeastern Brazil. *Bulletin of the United States National Museum* **206**, 1–423.

Cochran D. M. & Goin, C. J. (1959). A new frog of the genus *Limnomedusa* from Colombia. *Copeia* **1959**, 208–210.

Cochran D. M. & Goin, C. J. (1970). Frogs of Colombia. *Bulletin of the United States National Museum* **288**, 1–655.

Coloma L.A. (1995). Ecuadorian frogs of the genus *Colostethus* (Anura: Dendrobatidae). *University of Kansas Museum of Natural History, Miscellaneous Publications* **87**, 1–72

Coloma L. A. (1997). Morphology, systematics and phylogenetic relationships among frogs of the genus *Atelopus* (Anura:Bufonidae), University of Kansas.

Coloma L. A. (2002). Two new species of *Atelopus* (Anura: Bufonidae) from Ecuador. *Herpetologica* **58**, 229–252.

Coloma L. A., Carvajal-Endara, S., Dueñas, J. F., Paredes-Recalde, A., Morales-Mite, M., Almeida-Reinoso, D., Tapia, E. E., Hutter, C. R., Toral, E. & Guayasamin, J. M. (2012). Molecular phylogenetics of stream treefrogs of the *Hyloscirtus larinopygion* group (Anura: Hylidae), and description of two new species from Ecuador. *Zootaxa* **3364**, 1–78.

Coloma L. A., Duellman, W. E., Almendariz, A., Ron, S. R., Terán-Valdez, A. & Guayasamin, J. M. (2010). Five new (extinct?) species of *Atelopus* (Anura: Bufonidae) from Andean Colombia, Ecuador, and Peru. *Zootaxa* **2574**, 1–54.

Coloma L. A., Lötters, S. & Salas, A. W. (2000). Taxonomy of the *Atelopus ignescens* complex (Anura: Bufonidae): Designation of a neotype of *Atelopus ignescens* and recognition of *Atelopus exiguus*. *Herpetologica* **56**, 303–324.

Coloma L. A., Lötters, S., Duellman, W. E. & Miranda-Leiva, A. (2007). A taxonomic revision of *Atelopus pachydermus*, and description of two new (extinct?) species of *Atelopus* from Ecuador (Anura: Bufonidae). *Zootaxa* **1557**, 1–32.

Cope E. D. (1862a). Catalogues of the reptiles obtained during the explorations of the Parana, Paraguay, Vermejo and Uraguay Rivers, by Capt. Thos. J. Page, USN; and of those procured by Lieut. N. Michler, US Top. Eng., Commander of the expedition conducting the survey of the Atrato River. *Proceedings of the Academy of Natural Sciences of Philadelphia* **14**, 346–359.

Cope E. D. (1862b). On some new and little known American Anura. *Proceedings of the Academy of Natural Sciences of Philadelphia* **14**, 151-594.

Cope E. D. (1867). On the Reptilia and Batrachia of the Sonoran Province of the Nearctic Region. *Proceedings of the Academy of Natural Sciences of Philadelphia* **18**, 300–314.

Cope E. D. (1887). Synopsis of the Batrachia and Reptilia obtained by H. H. Smith, in the Province of Mato Grosso, Brazil. *Proceedings of the American Philosophical Society* **24**, 44–60.

Cope E. D. (1889). The Batrachia of North America. *Bulletin of the United States National Museum*, 1–525.

Crombie R. I. (1977). A new species of frog of the genus *Eleutherodactylus* (Amphibia: Leptodactylidae) from the Cockpit Country of Jamaica. *Proceedings of the Biological Society of Washington* **90**, 194–204.

Crombie R. I. (1986). Another New Forest-dwelling Frog (Leptodactylidae, *Eleutherodactylus*) from the Cockpit Country of Jamaica. *Transactions of the San Diego Society of Natural History* **21**, 145–153.

Cruz C. A. G. (1980). Descrição de uma nova espécies de Phyllomedusinae do estado do Espírito Santo, Brasil (Amphibia, Anura, Hylidae). *Revista Brasilera de Biologia* **40**, 683–687.

Cruz C. A. G. (1990). Sobre as relações intergenéricas de Phyllomedusinae da Floresta Atlântica (Amphibia, Anura, Hylidae). *Revista Brasileira de Biologia* **50**, 709–726.

Cruz C. A. G. & Fusinatto, L. A. (2008). A new species of *Dendrophryniscus*, Jiménez de la Espada, 1871 (Amphibia, Anura, Bufonidae) from the atlantic rain forest of Rio Grande do Sul, Brazil. *South American Journal of Herpetology* **3**, 22–26.

Cruz C. A. G. & Peixoto, O. L. (1985 "1984"). Especies verdes de *Hyla*: O complexo "*albosignata*" (Amphibia, Anura, Hylidae). *Arquivos de Universidade Federal Rural do Rio de Janeiro* **7**, 31–47.

Cruz C. A. G. & Peixoto, O. L. (1987 "1985"). Espécies verdes de *Hyla*: O complexo "*albofrenata*" (Amphibia, Anura, Hylidae). *Arquivos de Universidade Federal Rural do Rio de Janeiro* **8**, 59–70.

da Silva H. R., Campos, L. A. & Sebben, A. (2007). The auditory region of *Brachycephalus* and its bearing on the monophyly of the genus (Anura: Brachycephalidae). *Zootaxa* **1422**, 59–68.

Dahanukar N., Padhye, A. D., Salelkar, G. P. & Ghate, H. V. (2004). Aktueller Beleg für die Malabar-Baumkröte, *Pedostibes tuberculosus* Günther, 1876, in Indien. *Sauria. Berlin* **26**, 17–20.

Das A., Chetia, M., Dutta, S. K. & Sengupta, S. (2013). A new species of *Duttaphrynus* (Anura: Bufonidae) from Northeast India. *Zootaxa* **3646**, 336–348.

Das I. (2008). Two new species of *Pelophryne* (Anura: Bufonidae) from Gunung Murud, Sarawak (Northwestern Borneo). *Raffles Bulletin of Zoology* **56**, 435–443.

Davies M. (1984). Osteology of the Myobatrachine Frog *Arenophryne rotunda* Tyler (Anura: Leptodactylidae) and Comparisons with other Myobatrachine Genera. *Australian Journal of Zoology* **32**, 789–802.

Davies M. (1989). Ontogeny of bone and the role of heterochrony in the myobatrachine genera *Uperoleia*, *Crinia*, and *Pseudophryne* (Anura: Leptodactylidae: Myobatrachinae). *Journal of Morphology* **200**, 269–300.

Davies M. & Burton, T. C. (1982). Osteology and myology of the gastric brooding frog *Rheobatrachus silus* Liem (Anura: Leptodactylidae). *Australian Journal of Zoology* **30**, 501–521.

Davies M., Martin, A. A. & Watson, G. F. (1983). Redefinition of the *Litoria latopalmata* species group (Anura: Hylidae). *Trans. R. Soc. S. Aust.* **107**, 87-108.

Davis D. D. (1935). A new generic and family position for *Bufo borbonica*. *Zoological Series of Field Museum of Natural History* **20**, 87–92.

de la Riva I. (2002a). Rediscovery and taxonomic status of *Telmatobius marmoratus gigas* Vellard, 1969 "1968" (Anura: Leptodactylidae). *Herpetologica* **58**, 220–228.

de la Riva I. (2002b). Taxonomy and distribution of the south american toad *Bufo poeppigii* Tschudi, 1845 (Amphibia, Anura, Bufonidae). *Graellsia* **58**, 49–57.

de la Riva I. & Harvey, M. (2003). A new species of *Telmatobius* from Bolivia and a redescription of *T. simonsi* Parker, 1940 (Amphibia: Anura: Leptodactylidae). *Herpetologica* **59**, 127–142.

de la Riva I. & Lynch, J. D. (1997). New species of *Eleutherodactylus* from Bolivia (Amphibia: Leptodactylidae). *Copeia* **1997**, 151–157.

de la Riva I., Castroviejo-Fisher, S., Chaparro, J. C., Boistel, R. & Padial, J. M. (2011). A new species of *Atelopus* (Anura: Bufonidae) from the Amazonian slopes of the Andes in south-eastern Peru. *Salamandra* **47**, 161–168.

de la Riva I., Ríos, J. N. & Aparicio, J. (2005). A new species of *Bufo* (Anura: Bufonidae) from the Andes of Bolivia. *Herpetologica* **61**, 280–286.

de Sá R. O. (1996). *Hyla multifasciata*. *Catalogue of American Amphibians and Reptiles* **624**, 1–4.

de Sa R. O. & Trueb, L. (1991). Osteology, skeletal development, and chondrocranial structure of *Hamptophryne boliviana* (Anura: Microhylidae). *Journal of morphology* **209**, 311–330.

de Sá R. O., Grant T., Camargo A., Heyer, W. R., Ponssa, M. L. & Stanley, E. (2014). Systematics of the neotropical genus *Leptodactylus* Fitzinger, 1826 (Anura: Leptodactylidae): phylogeny, the relevance of non-molecular evidence, and species accounts. *South American Journal of Herpetology* **9**, s1–s128.

Delorme M., Dubois, A., Grosjean, S. & Ohler, A. (2006). Une nouvelle ergotaxinomie des Megophryidae (Amphibia, Anura). *Alytes* **24**, 6–21.

di Bernardo M., Maneyro, R. & Grillo, H. (2006). New species of *Melanophryniscus* (Anura: Bufonidae) from Rio Grande do Sul, Southern Brazil. *Journal of Herpetology* **40**, 261–266.

Dias P. H. S., Amaro, R. C., Carvalho-E-Silva, A. M. P. T. & Rodrigues, M. T. (2013). Two new species of *Proceratophrys* Miranda-Ribeiro, 1920 (Anura; Odontophrynidae) from the Atlantic forest, with taxonomic remarks on the genus. *Zootaxa* **3682**, 277–304.

Diaz L. M. & Cádiz, A. (2008). Guía taxonómica de los anfibios de Cuba. *Abc Taxa* **4**, 1–294.

Diaz L. M., Cadiz, A. & Hedges, S. B. (2003). A new grass frog from pine forests of Western Cuba, and description of acoustic and pattern variation in *Eleutherodactylus varleyi* (Amphibia: Leptodactylidae). *Caribbean Journal of Science* **39**, 176–188.

Díaz L. M., Cádiz, A. & Navarro, N. (2005). A new ground dwelling frog of the genus *Eleutherodactylus* (Anura: Leptodactylidae) from eastern Cuba, and a reconsideration of the *E. dimidiatus* group. *Caribbean Journal of Science* **41**, 307–318.

Díaz L. M., Estrada, A. R. & Hedges, S. B. (2001). A new riparial frog of the genus *Eleutherodactylus* (Anura: Leptodactylidae) from Eastern Cuba. *Caribbean Journal of Science* **37**, 63–71.

Díaz, L. M. & Fong, A. (2001). A new mottled frog of the genus *Eleutherodactylus* (Anura: Leptodactylidae) from eastern Cuba. *Solenodon*: *Revista Cubana de Taxonomía Zoológica* **1**, 76–84.

Diego-Aransay A. & Gorzula, S. (1990). Una nueva especies de *Oreophrynella* (Anura: Bufonidae) de la Guayan Venezolana. *Memoria de la Sociedad de Ciencias Naturales La Salle* **47**, 233–238.

Dodd K.C. (2013). *Frogs of the United States and Canada*. Johns Hopkins University Press. 2 Vols. 980 pp.

Drewes R. C. (1984). A phylogenetic analysis of the Hyperoliidae (Anura): Treefrogs of Africa, Madagascar, and the Seychelles islands. *Occasional Papers of the California Academy of Sciences* **139**, 70.

Dring J. C. M. (1987). Bornean treefrogs of the genus *Philautus* (Rhacophoridae). *Amphibia-Reptilia* **8**, 19–47.

du Preez L. & Carruthers, V. (2009). *A complete guide to the frogs of Southern Africa*. Struik Nature, Cape Town.

Du Toit C. (1933) Some aspects of the cranial morphology of *Rana grayi* Smith. *Proceedings of the Zoological Society of London* **103**, 715–734.

Dubois A. (1975). Un nouveau sous-genre (*Paa*) et trois nouvelles espèces du genre *Rana*. Remarques sur la phylogénies des Ranidés (Amphibiens, Anoures). *Bulletin du Muséum National d'Histoire Naturelle* **324**, 1093–1115.

Dubois A. & Ohler, A. (2005). Taxonomic notes on the Asian frogs of the tribe Paini (Ranidae, Dicroglossinae): 1. Morphology and synonymy of *Chaparana aenea* (Smith, 1922), with proposal of a new statistical method for testing homogeneity of small samples. *Journal of Natural History* **39**, 1759–1778.

Duellman W. E. (1963). A new species of tree frog, genus *Phyllomedusa*, from Costa Rica. *Revista de Biología Tropical* **11**, 1–23.

Duellman W. E. (1970a). The Genera of Phyllomedusine Frogs (Anura: Hylidae). *University of Kansas Publications, Museum of Natural History* **18**, 1–10.

Duellman W. E. (1970b). Hylid frogs of Middle America. *Monographs of the Museum of Natural History, University of Kansas* **1-2**, 1–753.

Duellman W. E. (1971). The identities of some Ecuadorian hylid frogs. *Herpetologica* **27**, 212–227.

Duellman W. E. (1972a). A review of the neotropical frogs of the *Hyla bogotensis* group. *Occasional Papers of the Museum of Natural History, University of Kansas* **11**, 1–31.

Duellman W. E. (1972b). The systematic status and life history of *Hyla rhodopepla* Günther. *Herpetologica* **28**, 369–375.

Duellman W. E. (1973). Descriptions of new hylid frogs from Colombia and Ecuador. *Herpetologica* **29**, 219–227.

Duellman W. E. (1974). A reassesement of the taxonomic status of some neotropical hylid frogs. *Occasional Papers of the Museum of Natural History, University of Kansas* **27**, 1–27.

Duellman W. E. (1978). Three new species of *Eleutherodactylus* from Amazonian Peru (Amphibia: Anura: Leptodactylidae). *Herpetologica* **34**, 264–270.

Duellman W. E. (1989). New species of hylid frogs from the Andes of Colombia and Venezuela. *Occasional Papers of the Museum of Natural History, The University of Kansas* **131**, 1–12.

Duellman W. E. (1990). A new species of leptodactylid frog, genus *Ischnocnema*, from Peru. *Occasional papers of the Museum of Natural History, The University of Kansas* **138**, 1–7.

Duellman W. E. (2001). *Hylid Frogs of Middle America*. Society for the Study of Amphibians and Reptiles, Ithaca.

Duellman W. E. (2005). *Cusco Amazonico. The lives of amphibians and reptiles in an Amazonian rainforest*. Cornell University Press, Ithaca.

Duellman W. E. (2015). *Marsupial Frogs:* Gastrotheca *and allied genera*. Johns Hopkins University Press, Baltimore, Maryland.

Duellman W. E. & Burrowes, P. A. (1989) New species of frogs, *Centrolenella*, from the Pacific versant of Ecuador and southern Colombia. *Occasional Papers of the Museum of Natural History, University of Kansas* **132**, 1–14.

Duellman W. E. & Crump, M. (1974). Speciation in frogs of the *Hyla parviceps* group in the upper amazon basin. *Occasional Papers of the Museum of Natural History, University of Kansas* **23**, 1–40.

Duellman W. E. & Hedges, S. B. (2005). Eleutherodactyline frogs (Anura: Leptodactylidae) from the Cordillera Yanchaga in central Peru. *Copeia* **2005**, 526–538.

Duellman W. E. & Hoogmoed, M. S. (1984). The taxonomy and phylogenetic relationships of the hylid frog genus *Stefania*. *Miscellaneous Publications of the Museum of Natural History, University of Kansas* **75**, 1–39.

Duellman W. E. & Hoogmoed, M. S. (1992). Some hylid frogs from the Guiana highlands, northeastern south America: new species, distributional records, and a generic reallocation. *Occasional Papers of the Museum of Natural History, University of Kansas* **147**, 1–21.

Duellman W. E. & Lehr, E. (2009). *Terrestrial-breeding frogs Strabomantidae in Peru*. Natur und Tier Verlag, Münster, Germany.

Duellman W. E. & Ochoa, O. (1991). A new species of *Bufo* (Anura: Bufonidae) from the Andes of Southern Peru. *Copeia* **1991**, 137–141.

Duellman W. E. & Pramuk, J. B. (1999). Frogs of the genus *Eleutherodactylus* (Anura: Leptodactylidae) in the Andes of northern Peru. *Scientific papers. Natural History Museum, the University of Kansas*, 1–78.

Duellman W. E. & Schulte, R. (1992). Description of a new species of *Bufo* from northern Peru with comments on phenetic groups of South American toads (Anura: Bufonidae). *Copeia* **1992**, 162–172.

Duellman W. E. & Toft, C. A. (1979). Anurans from the Serranía de Sira, Amazonian Perú: Taxonomy and biogeography. *Herpetologica* **35**, 60–70.

Duellman W. E. & Trueb, L. (1967). Two new species of tree frogs (genus *Phyllomedusa*) from Panamá. *Copeia* **1967**, 125–131.

Duellman W. E. & Trueb, L. (1976). The systematic status and relationships of the hylid frog *Nyctimantis rugiceps* Boulenger. *Occasional Papers of the Museum of Natural History, University of Kansas* **58**, 1–14.

Duellman W. E. & Trueb, L. (1989). Two new treefrogs of the *Hyla parviceps* group from the amazon basin in southestern Peru. *Herpetologica* **45**, 1–10.

Duellman W. E. & Wild, E. R. (1993). Anuran amphibians from the Cordillera de Huancabamba, northern Peru: Systematics, ecology, and biogeography. *Occasional Papers of the Museum of Natural History, The University of Kansas*, 1–53.

Duellman W. E., de La Riva, I. & Wild, E. R. (1997). Frogs of the *Hyla armata* and *Hyla pulchella* groups in the Andes of South America, with definitions and analyses of phylogenetic relationships of Andean groups of *Hyla*. *Scientific Papers of the Natural History Museum, The University of Kansas* **3**, 1–41.

Duellman W. E., Jungfer, K.-H. & Blackburn, D. C. (2011). The phylogenetic relationship of geographically separated "*Flectonotus*"(Anura: Hemiphractidae), as revealed by molecular, behavioral, and morphological data. *Phyllomedusa: Journal of Herpetology* **10**, 15–29.

Duellman, W. E. (1981) Three new species of centrolenid frogs from the Pacific versant of Ecuador and Colombia. **88**, 1–9.

Dunn E. R. (1925). New frogs from Cuba. *Occasional Papers of the Boston Society of Natural History* **5**, 163–166.

Dunn E. R. (1926). Additional frogs from Cuba. *Occasional Papers of the Boston Society of Natural History* **5**, 209–215.

Ecke H. (1934). Anatomische und histologische Untersuchungen am Labyrinth der Erdkröte (*Bufo vulgaris* Laur.). *Zeitschrift für Morphologie und Ökologie der Tiere* **29**, 79–113.

Ecker A. (1889). *The Anatomy of the Frog*. Asher, Amsterdam.

Elliot L., Gerhardt, C. & Davidson, C. (2009). *The frogs and Toads of North America. A comprehensive guide to their identification, behavior, and calls*. Houghton Mifflin Harcourt.

Emerson S. B. & Berrigan, D. (1993). Systematics of Southeast Asian ranids: Multiple originas of voicelessness in the subgenus *Limnonectes* (Fitzinger). *Herpetologica* **49**, 22–31.

Estrada A. R. & Alonso, R. (1997). Nueva especie del grupo *limbatus* (Leptodactylidae: *Eleutherodactylus*) de la región oriental de Cuba. *Caribbean Journal of Science* **33**, 41–44.

Estrada A. R. & Hedges, S. B. (1991). Nueva especie de *Eleutherodactylus* (Anura: Leptodactylidae) de la region oriental de Cuba. *Caribbean Journal of Science* **27**, 139–145.

Estrada A. R. & Hedges, S. B. (1996). A new frog of the *Eleutherodactylus* from eastern Cuba (Anura: Leptodactylidae). *Herpetologica* **52**, 435–439.

Estrada A. R. & Hedges, S. B. (1997a). A new species of frog from the Meseta de Cabo Cruz, eastern Cuba (Leptodactylidae, *Eleutherodactylus*). *Caribbean Journal of Science* **33**, 227–232.

Estrada A. R. & Hedges, S. B. (1997b). A new species of frog from the Sierra Maestra, Cuba (Leptodactylidae), *Eleutherodactylus*). *Journal of Herpetology* **31**, 364–368.

Estrada A. R. & Hedges, S. B. (1997c). Nueva especie de *Eleutherodactylus* (Anura: Leptodactylidae) del Macizo Sagua-Baracoa, Cuba. *Caribbean Journal of Science* **33**, 222–226.

Estrada A. R., Díaz, L. M. & Rodríguez, A. (1998). Nueva especie de *Eleutherodactylus* (Anura: Leptodactylidae) del litoral norte de La Habana, Cuba. *Revista Española de Herpetología* **11**, 19–29.

Evans B. J., Carter, T. F., Greenbaum, E., Gvoždík, V., Kelley, D. B., McLaughlin, P. J., Pauwels, O. S. G., Portik, D. M., Stanley, E. L. & Tinsley, R.C. (2015). Genetics, Morphology, Advertisement Calls, and Historical Records Distinguish Six New Polyploid Species of African Clawed Frog (*Xenopus*, Pipidae) from West and Central Africa. *PloS One* **10**, e0142823.

Fabrezi M. & Langone, J. A. (2000). Los caracteres morfológicos del controvertido Neobatrachia arborícola *Allophryne ruthveni* Gaige, 1926. *Cuadernos de herpetología* **14**, 47–59.

Faivovich J., Moravec, J., Cisneros–Heredia, D. F. & Köhler, J. (2006). A new species of the *Hypsiboas benitezi* group from the western amazon basin (Amphibia: Anura: Hylidae). *Herpetologica* **62**, 96–108.

Fei L., Ye, C.-Y. & Jiang, J.-P. (2003). A new bufonid genus *Parapelophryne* from China (Amphibia, Anura). *Acta Zootaxonomica Sinica* **28**, 762–766.

Feldkamp L.A., Davis L.C., Kress J.W. (1984). Practical cone-beam algorithm. Journal of the Optical Society of America A 1(6), 612–619.

Fenolio D. B., Mendelson, J. R. & Lamar, W. W. (2012). A new diagnosis and description of variation among adult *Rhinella ceratophrys* (Boulenger) (Amphibia: Bufonidae), with notes on ecology and distribution. *South American Journal of Herpetology* **7**, 9–15.

Fernando P., Dayawansa, N. & Siriwardhane, M. (1994). *Bufo kotagamai*, a new toad (Bufonidae) from Sri Lanka. *Journal of South Asian Natural History* **1**, 119–124.

Firschein I. L. (1950). A new toad from Mexico with a redefinition of the *cristatus* group. *Copeia* **1950**, 81–87.

Flores G. & Vigle, G. O. (1994). A new species of *Eleutherodactylus* (Anura: Leptodactylidae) from the lowland rainforests of Amazonian Ecuador, with notes on the *Eleutherodactylus frater* assembly. *Journal of Herpetology* **28**, 416–424.

Folly M., Hepp, F., Carvalho-e-Silva, S. P. & Duellman, W. E. (2014). Taxonomic status and redescription of *Flectonotus ulei* (Anura: Hemiphractidae), with a key for the species of *Fritziana*. *Zoologia (Curitiba)* **31**, 393–399.

Ford L. S. & Savage, J. M. (1984). A new frog of the genus *Eleutherodactylus* (Leptodactylidae) from Guatemala. *Occasional Papers of the Museum of Natural History, The University of Kansas* **110**, 1–9.

Formas J. R. (1989) A new species of *Eupsophus* (Amphibia, Anura, Leptodactylidae) from Southern Chile. *Proceedings of the Biological Society of Washington* **102**, 568–576.

Formas J. R., Benavides, E. & Cuevas, C. (2003). A new species of *Telmatobius* (Anura: Leptodactylidae) from río Vilama, northern Chile, and the redescription of *T. halli* Noble. *Herpetologica* **59**, 253–270.

Formas J. R., Cuevas, C. C. & Brieva, L. M. (2002). A new species of *Alsodes* (Anura: Leptodactylidae) from Cerro Mirador, Cordillera Pelada, southern Chile. *Proceedings of the Biological Society of Washington* **115**, 708–719.

Formas J. R., Nuñez, J. & Cuevas, C. (2008). Identidad de la rana austral chilena *Eupsophus coppingeri* (Amphibia, Anura, Neobatrachia): evidencias morfológicas, cromosómicas y moleculares. *Revista Chilena de Historia Natural* **81**, 3–20.

Formas J. R., Núñez, J. J. & Brieva, L. M. (2001). Osteología, taxonomía y relaciones filogenéticas de las ranas del género *Telmatobufo* (Leptodactylidae). *Revista Chilena de Historia Natural* **74**, 365–387.

Fouquet A., Blotto, B. L., Maronna, M. M., Verdade, V. K., Juncá, F. A., de Sá, R. & Rodrigues, M. T. (2013). Unexpected phylogenetic positions of the genera *Rupirana* and *Crossodactylodes* reveal insights into the biogeography and reproductive evolution of leptodactylid frogs. *Molecular Phylogenetics and Evolution* **67**, 445–457.

Fouquet A., Gaucher, P., Blanc, M. & Velez-Rodriguez, C. M. (2007). Description of two new species of *Rhinella* (Anura: Bufonidae) from the lowlands of the Guiana shield. *Zootaxa* **1663**, 17–32.

Frost D. R. (1986). A new *Colostethus* (Anura: Dendrobatidae) from Ecuador. *Proceedings of the Biological Society of Washington* **99**, 214–217.

Fu J., LathrOP; A. & Murphy, R. W. (1997). Phylogeny of genus *Scutiger* (Amphibia: Megophryidae): A re-evaluation. *Asiatic Herpetological Research* **7**, 32–37.

Fuiten A. M. (2012). Skeletal Variation in Melanesian Forest Frogs (Anura: Ceratobatrachidae), University of Kansas.

Funkhouser A. (1957). A review of the neotropical tree frogs of the genus *Phyllomedusa*. *Occasional Papers of the Natural History Museum of Stanford University* **5**, 1–90.

Gabadage D. E., de Silva, A., Botejue, W. M. S., Bahir, M. M., Surasinghe, T. D., Madawala, M. B., Amarasinghe, A. A. T. & Karunarathna, D. M. S. S. (2014). On the discovery of second living population of *Adenomus kandianus* (Günther, 1872) from Sri Lanka: with the bioecology, and detailed redescription to the species. *Herpetotropicos* **10**, 37–49.

Gallardo J. M. (1961). Three new toads from South America: *Bufo manicoriensis*, *Bufo spinulosus altiperuvianus* and *Bufo quechua*. *Breviora* **141**, 1–8.

Gallardo J. M. (1962a). Caracterización de *Bufo rubropunctatus* Guichenot y su presencia en Argentina. *Neotropica* **8**, 28–30.

Gallardo J. M. (1962b). A propósito de *Bufo variegatus* (Günther), sapo del Bosque Húmedo Antartándico, y las otras especies de *Bufo* neotropicales. *Physis* **23**, 93–102.

Gallardo J. M. (1965). Especiación en tres *Bufo* neotropicales (Amphibia, Anura). *Papéis Avulsos do Departamento de Zoologia* **17**, 57–75.

Gallardo J. M. (1967). *Bufo gnustae* sp. nov. del grupo de *B. ockendeni* Boulenger, hallado en la provincia de Jujuy, Argentina. *Neotropica* **13**, 54–56.

Garcia P. C. A., Berneck, B. V. M. & Costa, C. O. R. (2009). A new species of *Paratelmatobius* (Amphibia, Anura, Leptodactylidae) from Atlantic Rain Forest of southeastern Brazil. *South American Journal of Herpetology* **4**, 217–224.

Garcia P. C. A., Faivovich, J. & Haddad, C. F. B. (2007). Redescription of *Hypsiboas semiguttatus*, with the description of a new species of the *Hypsiboas pulchellus* group. *Copeia* **2007**, 933–951.

Garcia P. C. A., Vinciprova, G. & Haddad, C. F. B. (2001). Vocalização, girino, distribução geografica e novos comentarios sobre *Hyla marginata* Boulenger, 1887 (Anura, Hylidae, Hylinae). *Boletim do Museu Nacional do Rio de Janeiro* **460**, 19 pp.

Garcia P. C. A., Vinciprova, G. & Haddad, C. F. B. (2003). The taxonomic status of *Hyla pulchella joaquini* B. Lutz, 1968 (Anura: Hylidae). *Herpetologica* **59**, 350–363.

García-R J. C. & Lynch, J. D. (2006). A new species of frog (genus *Eleutherodactylus*) from a cloud forest in Western Colombia. *Zootaxa* **1171**, 39–45.

Gaudin A. J. (1974). An osteological analysis of Holarctic tree frogs, family Hylidae. *Journal of Herpetology* **8**, 141–152.

Gaudin A. J. (1978). The sequence of cranial ossification in the California toad, *Bufo boreas* (Amphibia, Anura, Bufonidae). *Journal of Herpetology*, 309–318.

Giaretta A. A. & Sawaya, R. J. (1998). Second species of *Psyllophryne* (Anura: Brachycephalidae). *Copeia* **1998**, 985–987.

Giaretta A. A., Bernarde, P. S. & Kokubum, M. N. C. (2000) A new species of *Proceratophrys* (Anura: Leptodactylidae) from the Amazon rain forest. *Journal of Herpetology* **34**, 173–178.

Girard C. F. (1854). A list of north american bufonids with diagnoses of new species. *Proceedings of the National Academy of Sciences* **7**, 68–88.

Giraudo A. R., Krauczuk, E. R. & Baldo, D. (2005). *Hyla uruguaya* Schmidt, 1944, un nuevo anfibio para la herpetofauna de Argentina. *Cuadernos de Herpetología* **18**, 61–66.

Glaw F. & Vences, M. (2007). *A field guide to the amphibians and reptiles of Madagascar*. Vences & Glaw, Köln, Germany.

Gluesenkamp A. G. & Guayasamin, J. M. (2008). A new species of *Osornophryne* (Anura: Bufonidae) from the Andean highlands of northern Ecuador. *Zootaxa* **1828**, 18–28.

Gluesenkamp A. G. (1995). A new species of *Osornophryne* (Anura: Bufonidae) from Volcan Sumaco, Ecuador with notes on other members of the genus. *Herpetologica* **51**, 268–279.

Goin C. J. (1955). Description of a new subspecies of the frog *Eleutherodactylus ricordi* from the Bahama Islands. *American Museum Novitates* **1708**, 1–7.

Gollmann G. (1991). Osteological variation in *Geocrinia laevis*, *Geocrinia victoriana*, and their hybrid populations (Amphibia, Anura, Myobatrachinae). *Journal of Zoological Systematics and Evolutionary Research* **29**, 289–303.

Gomes N. (1988). Osteologia comparada de duas espécies do genero *Crossodactylodes* Cochran, 1938 (Amphibia, Anura, Leptodactylidae). *Memorias do Instituto Butantan* **50**, 51–61.

Grandison A. G. C. (1972). The status and relationships of some East African earless toads (Anura, Bufonidae) with a description of a new species. *Zoologische Mededelingen* **47**, 30–48.

Grandison A. G. C. (1978). The occurrence of *Nectophrynoides* (Anura Bufonidae) in Ethiopia. A new concept of the genus with a description of a new species. *Monitore Zoologico Italiano* **6**, 119–172.

Grandison A. G. C. (1980). A new genus of toad (Anura: Bufonidae) from the Republic of South Africa with remarks on its relationships. *Bulletin of the British Museum of Natural History* **39**, 293–298.

Grandison A. G. C. (1981). Morphology and phylogenetic position of the west african *Didynamipus sjoestedti* Andersson, 1903 (Anura Bufonidae). *Monitore Zoologico Italiano* **15**, 187–215.

Grant T. & Bolívar-G, W. (2014). A new species of semiarboreal toad with a salamander-like ear (Anura: Bufonidae: *Rhinella*). *Herpetologica* **70**, 198–210.

Grant T. (2000). Una nueva especie de *Rhamphophryne* (Anura: Bufonidae) de la cordillera central de Colombia. *Revista de la Academia Colombiana de Ciencias Exactas, Físicas y Naturales* **23**, 287–292.

Gray P. & Cannatella, D. C. (1985). A new species of *Atelopus* (Anura, Bufonidae) from the Andes of northern Perú. *Copeia* **1985**, 910–917.

Graybeal A. & Cannatella, D. C. (1995). A new taxon of Bufonidae from Peru, with descriptions of two new species and a review of the phylogenetic status of supraspecific bufonid taxa. *Herpetologica* **51**, 105–131.

Greding E. J., Jr. (1972). An unusually large toad (Anura: Bufonidae) from the lower southeastern slope Volvan Turrialba, with a key to the *Bufo* of Costa Rica. *Caribbean Journal of Science* **12**, 91–94.

Gridi-Papp M., Feng, A. S., Shen, J.-X., Yu, Z.-L., Rosowski, J. J. & Narins, P. M. (2008). Active control of ultrasonic hearing in frogs. *Proceedings of the National Academy of Sciences* **105**, 11014–11019.

Griffiths I. (1954). On the "otic element" in Amphibia Salientia. *Proceedings of the Zoological Society of London* **124**, 35–54.

Grismer L. L. (2006). A new species of *Ansonia* Stoliczka, 1870 (Anura: Bufonidae) from a lowland rainforest in southern peninsular Malaysia. *Herpetologica* **62**, 466–475.

Grismer L. L. (2007). A new species of *Ingerophrynus* (Anura: Bufonidae) from a lowland rain forest in Southern Peninsular Malaysia. *Journal of Herpetology* **41**, 225–230.

Guayasamin J. M. & Trueb, L. (2007). A new species of glassfrog (Anura: Centrolenidae) from the lowlands of northwestern Ecuador, with comments on centrolenid osteology. *Zootaxa* **1447**, 27–45.

Guayasamin J. M., Bustamante M. R., Almeida-Reinoso D. & Funk W. C. (2006) Glass frogs (Centrolenidae) of Yanayacu Biological Station, Ecuador, with the description of a new species and comments on centrolenid systematics. *Zoological Journal of the Linnean Society* **147**, 489–513.

Guayasamin J. M., Castroviejo-Fisher S., Trueb L., Ayarzagüena J., Rada M. & Vilà C. (2009) Phylogenetic systematics of Glassfrogs (Amphibia: Centrolenidae) and their sister taxon *Allophryne ruthveni*. *Zootaxa* **2100**, 1–97.

Guibé J. (1974). Batraciens nouveaux de Madagascar. *Bulletin du Muséum National d'Histoire Naturelle. Serie 3, Zoologie* **171**, 1069–1192.

Günther A. C. L. G. (1858). *Catalogue of the Batrachia Salientia in the Collection of the British Museum*. British Museum, London.

Günther A. C. L. G. (1864). The Reptiles of British India. London: Ray Society by R. Hardwicke.

Günther A. C. L. G. (1872). Descriptions of some ceylonese reptiles and batrachians. *Journal of Natural History* **9**, 85–88.

Günther A. C. L. G. (1875). Third report on collections of Indian reptiles obtained by the British Museum. *Proceedings of the Zoological Society of London* **1875**, 567–577.

Günther A. C. L. G. (1896). Report on the collections of reptiles, batrachians and fishes made by Messrs Potanin and Berezowski in the Chinese provinces kansu and Sze-Chuan. *Annuaire du Musée Zoologique de l'Academie Impériale des Sciences de St. Pétersbourg* **1**, 199–219.

Günther R. (2003). Three new species of the genus *Oreophryne* from western Papua, Indonesia. *Spixiana* **26**, 175–191.

Günther R. (2009). *Metamagnusia* and *Pseudocallulops*, two new genera of microhylid frogs from New Guinea (Amphibia, Anura, Microhylidae). *Zoosystematics and Evolution* **85**, 171–187.

Haas A. (1995). Cranial features of dendrobatid larvae (Anura: Anura: Dendrobatidae). *Journal of morphology* **224**, 241–264.

Haas A. (1999). Larval and metamorphic skeletal development in the fast-developing frog *Pyxicephalus adspersus* (Anura, Ranidae). *Zoomorphology* **119**, 23–35.

Haddad C. F. B., Alves, A. C. R., Clemente-Carvalho, R. B. G. & dos Reis, S. F. (2010). A new species of *Brachycephalus* from the Atlantic rain forest in São Paulo state, southeastern Brazil (Amphibia: Anura: Brachycephalidae). *Copeia* **2010**, 410–420.

Haddad C. F. B., Toledo, L. F., Prado, C., Loebmann, D., Gasparini, J. & Sazima, I. (2013). *Guia dos Anfibios da Mata Atlãntica*. Anolis Books, Brazil.

Hall J. A. & Larsen, J. H. (1998). Postembryonic ontogeny of the spadefoot toad, *Scaphiopus intermontanus* (Anura: Pelobatidae): Skeletal morphology. *Journal of morphology* **238**, 179–244.

Harper E. B., Measey, G. J., Patrick, D. A., Menegon, M. & Vonesh, J. R. (2010). *Field guide to the amphibians of the Eastern Arc Mountains and coastal forests of Tanzania and Kenya*. Camerapix Publishers International, Nairobi, Kenya.

Harvey M. B. & Keck, M. B. (1995). A new species of *Ischnocnema* (Anura: Leptodactylidae) from high elevations in the Andes of central Bolivia. *Herpetologica*, **51**, 56–66.

Harvey M. B. & Sheehy, C. M. (2005). A new species of *Ischnocnema* (Anura: Leptodactylidae) from La Paz, Bolivia. *Herpetologica* **61**, 268–275.

Harvey M. B. & Smith, E. N. (1993). A new aquatic *Bufo* (Anura: Bufonidae) from cloud forests in the Serranía de Siberia, Bolivia. *Proceedings of the Biological Society of Washington* **106**, 442–449.

Harvey M. B. & Smith, E. N. (1994). A new species of *Bufo* (Anura: Bufonidae) from cloud forest in Bolivia. *Herpetologica* **50**, 32–38.

Hedges S. B. & Thomas, R. (1987). A new burrowing frog from Hispaniola with comments on the *inoptatus* group of the genus *Eleutherodactylus* (Anura: Leptodactylidae). *Herpetologica* **43**, 269–279.

Hedges S. B. & Thomas, R. (1992a). A new marsh-dwelling species of *Eleutherodactylus* from Haiti (Anura: Leptodactylidae). *Journal of Herpetology* **26**, 191–195.

Hedges S. B. & Thomas, R. (1992b). Two new species of *Eleutherodactylus* from remnant cloud forest in Haiti (Anura: Leptodactylidae). *Herpetologica* **48**, 351-358.

Hedges S. B. (1988). A new diminutive frog from Hispaniola (Leptodactylidae: *Eleutherodactylus*). *Copeia* **1988**, 636–641.

Hedges S. B. (1992). A reconsideration of two montane species of *Eleutherodactylus* in Hispaniola (Anura: Leptodactylidae). *Caribbean Journal of Science* **28**, 11–16.

Hedges S. B., Duellman, W. E. & Heinicke, M. P. (2008). New World direct-developing frogs (Anura: Terrarana): Molecular phylogeny, classification, biogeography, and conservation. *Zootaxa* **1737**, 1–182.

Hedges S. B., Estrada, A. R. & Thomas, R. (1992). Three new species of *Eleutherodactylus* from eastern Cuba, with notes on vocalizations of other species (Anura: Leptodactylidae). *Herpetological Monographs* **6**, 68–83.

Hedges S. B., Thomas, R. & Franz, R. (1987). A new species of *Eleutherodactylus* (Anura, Leptodactylidae) from the Massif de la Hotte, Haiti. *Copeia* **1987**, 943–949.

Heinicke M. P., Duellman, W. E., Trueb, L., Means, D. B., MacCulloch, R. D. & Hedges, S. B. (2009). A new frog family (Anura: Terrarana) from South America and an expanded direct-developing clade revealed by molecular phylogeny. *Zootaxa* **2211**, 1–35.

Hertwig S., Min, P., Haas, A. & Das, I. (2014). Dressed in black. A Nnw *Ansonia* Stoliczka, 1870 (Lissamphibia: Anura: Bufonidae) from Gunung Murud, Sarawak, east Malaysia (Borneo). *Zootaxa* **3814**, 419–431.

Hetherington T. E. & Lindquist, E. D. (1999). Lung-based hearing in an “earless” anuran amphibian. *Journal of Comparative Physiology A* **184**, 395–401.

Hewitt J. (1926a). 11. Some new or little-known reptiles and batrachians from South Africa. *Annals of the South African Museum* **20**, 413–431.

Hewitt J. (1926b). 14. Some new or little-known reptiles and batrachians from South Africa. *Annals of the South African Museum* **20**, 473–491.

Heyer W. R. (1969). Studies on the genus *Leptodactylus* (Amphibia, Leptodactylidae) III. A redefinition of the genus *Leptodactylus* and a description of a new genus of leptodactylid frogs. **155**, 1–14.

Heyer W. R. (1977). Taxonomic notes on frogs from the Madeira and Purus rivers, Brasil. *Papeis Avulsos de Zoologia. São Paulo* **31**, 141–162.

Heyer W. R. (1984). Variation, systematics, and zoogeography of *Eleutherodactylus guentheri* and closely related species (Amphibia: Anura: Leptodactylidae). *Smithsonian Contributions to Zoology* **402**, 1–42.

Heyer W. R. (1985). New species of frogs from Boraceia, São Paulo, Brazil. *Proceedings of the Biological Society of Washington* **98**, 657–671.

Heyer W. R. (1999). A new genus and species of frog from Bahia, Brazil (Amphibia: Anura: Letodactylidae) with comments on the zoogeography of the Brazilian campos rupestres. *Proceedings of the Biological Society of Washington* **112**, 19–39.

Heyer W. R. & Cocroft, R. B. (1986) Descriptions of two new species of *Hylodes* from the Atlantic Forests of Brazil (Amphibia: Leptodactylidae). *Proceedings of the Biological Society of Washington* **99**, 100–109.

Heyer W. R. & Liem, D. S. (1976). Analysis of the intergeneric relationships of the Australian frog family Myobatrachidae. *Smithsonian Contributions to Zoology* **233**, 1–29.

Heyer W. R., Rand, A. S., Cruz, C. A. G. d., Peixoto, O. L. & Nelson, C. E. (1990). Frogs of Boracéia. *Arquivos de Zoologia* **31**, 231–410.

Hillis D. M. & de Sa, R. (1988). Phylogeny and taxonomy of the *Rana palmipes* Group (Salientia: Ranidae). *Herpetological Monographs* **2**, 1-26.

Hoogmoed M. S. (1972). On a new species of toad from Southern Morocco. *Zoologische Mededelingen* **47**, 49–64.

Hoogmoed M. S. (1979). Resurrection of *Hyla ornatissima* Noble (Amphibia, Hylidae) and remarks on related species of green tree frogs from the Guiana area. Notes on the herpetofauna of Surinam VI. *Zoologische Verhandelingen* **172**, 1–46.

Hoogmoed M. S. (1985). A new genus of toads (Amphibia: Anura: Bufonidae) from the pacific slopes of the Andes in northern Ecuador and southern Colombia, with the description of two new species. *Zoologische Mededelingen* **59**, 251–274.

Hoogmoed M. S. (1987). New *Osornophryne* (Amphibia: Anura: Bufonidae) from the Atlantic Versant of the Andes of Ecuador. *Zoologische Mededelingen* **61**, 209–242.

Hoogmoed M. S. (1989). On the identity of some toads of the genus *Bufo* from Ecuador, with additional remarks on *Andinophryne colomai* Hoogmoed, 1985 (Amphibia: Anura; Bufonidae). *Zoologische Verhandelingen* **250**, 1–32.

Hoogmoed M. S. & Lescure, J. (1984). A new genus and two new species of minute leptodactylid frogs from northern South America, with comments upon *Phyzelaphryne* (Amphibia: Anura: Leptodactylidae). *Zoologische Mededelingen* **58**, 85–115.

Hoogmoed M. S., Borges, D. & Cascon, P. (1994). Three new species of the genus *Adelophryne* (Amphibia: Anura: Leptodactylidae) from northeastern Brazil, with remarks on the other species of the genus. *Zoologische Mededelingen* **68**, 271–300.

Hoogmoed M. S., Lynch, J. D. & Lescure, J. (1977). A new species of *Eleutherodactylus* from Guiana (Leptodactylidae, Anura). *Zoologische Mededelingen* **51**, 33–41.

Hoyos J. M., Medina, P. & Schoch, P. (2015). Osteology of *Atelopus muisca* (Anura, Bufonidae) from Colombia. *Zootaxa* **3905**, 119–130.

Ibáñez D R. & Crawford, A. J. (2004). A new species of *Eleutherodactylus* (Anura: Leptodactylidae) from the Darién Province, Panama. *Journal of Herpetology* **38**, 240–243.

Ibañez D. R., Jaramillo, C. A. & Solís, F. A. (1995). Una especie nueva de *Atelopus* (Amphibia: Bufonidae) de Panama. *Caribbean Journal of Science* **31**, 57–64.

Ibañez R., Jaramillo, C. A. & Arosemena, F. A. (1994). A new species of *Eleutherodactylus* (Anura: Leptodactylidae) from Panamá. *Amphibia-Reptilia* **15**, 337–341.

Ibarra-Vidal, H., Ortiz, J. C. & Torres-Perez, F. (2004) *Eupsophus septentrionalis* n. sp., nueva especie de Leptodactylidae (Amphibia) de Chile Central. *Boletín de la Sociedad de Biología de Concepción* **75**, 91–102.

Inger R. F. (1960). A review of the oriental toads of the genus *Ansonia* Stoliczka. *Fieldiana Zoology* **39**, 473–503.

Inger R. F. (1964). Two new species of frogs from Borneo. *Fieldiana Zoology* **44**, 151–159.

Inger R. F. (1966). The systematics and zoogeography of the Amphibia of Borneo. *Fieldiana Zoology* **53**, 1–402.

Inger R. F. & Menzies, J. I. (1961). A new species of toad (*Bufo*) from Sierra Leone. *Fieldiana Zoology* **39**, 589–595.

Inger R. F. & Stuebing R. B. (1997) *A field guide to the frogs of Borneo*. Natural History Publications.

Inger R. F. & Stuebing, R. B. (2009). New species and new records of bornean frogs (Amphibia: Anura). *Raffles Bulletin of Zoology* **57**, 527–535.

Inger R. F., Orlov, N. & Darevsky, I. (1999). Frogs of Vietnam: A report on new collections. *Fieldiana, Zoology* **92**, 1–46.

Inger R. F., Shaffer, H. B., Koshy, M. & Bakde, R. (1984). A report on a collection of amphibians and reptiles from the Ponmudi, Kerala, South India. *Journal of the Bombay Natural History Society* **81**, 406–427.

Inger R. F., Stuart B. L. & Iskandar D. T. (2009) Systematics of a widespread southeast Asian frog, *Rana chalconota* (Amphibia: Anura: Ranidae). *Zoological Journal of the Linnean Society* **155**, 123–147

Inger R., Boeadi & Taufik A. (1996) New species of ranid frogs (Amphibia: Anura) from central Kalimantan, Borneo. *Raffles Bulletin of Zoology* **44**, 363–369.

Iskandar D. T. (1998). *The amphibians of Java and Bali*. Research and Development Centr for Biology - LIPI.

Iskandar D. T. & Mumpuni. (2004). A new toad of the genus *Ansonia* (Amphibia, Anura, Bufonidae) from Sumatra, Indonesia. *Hamadryad* **28**, 59–65.

Iskandar D. T., Bickford, D. P. & Arifin, U. (2011). A new *Ingerana* (Anura, Dicroglossidae) with no external tympanum from Borneo, Indonesia. *Raffles Bulletin of Zoology* **59**, 213–218.

Izecksohn E. (1968). Nova espécie de "*Dendrophryniscus*" do Estado do Rio de Janeiro (Amphibia, Salientia). *Revista Brasileira de Biologia* **28**, 357–362.

Izecksohn E. (1976). O status sistemático de *Phryniscus proboscideus* Boulenger (Amphibia, Anura, Bufonidae). *Revista Brasileira de Biología* **36**, 341–345.

Izecksohn E. (1993a). Nova especie de *Dendrophryniscus* da regiao amazonica (Amphibia, Anura, Bufonidae). *Revista Brasileira de Biologia* **10**, 407–412.

Izecksohn E. (1993b). Tres novas especies de *Dendrophryniscus* Jiménez de la Espada das regioes sudeste e sul do Brasil (Amphibia, Anura, Bufonidae). *Revista Brasileira de Biologia* **10**, 473–488.

Izecksohn E. & Carvalho-e-Silva, S. P. (2001). *Anfíbios do Município do Rio de Janeiro*. Editora UFRJ, Rio de janeiro, Brasil.

Izecksohn E. & Cruz, C. A. G. (1976). Nove espécie de Phyllomedusinae do estado do Espírito Santo, Brasil (Amphibia, Anura, Hylidae). *Revista Brasileira de Biologia* **36**, 257–261.

Izecksohn E., Carvalho-e-Silva, S. P. & Deiss, I. (2005) The osteocranium of *Proceratophrys boiei* (Wied-Neuwied), *P. appendiculata* (Günther), *P. melanopogon* (Miranda-Ribeiro), and *P. laticeps* Izecksohn & Peixoto,(Anura, Leptodactylidae). *Revista Brasileira de Zoologia* **22**, 225–229.

Jaslow A. P. & Lombard, R. E. (1996). Hearing in the neotropical frog, *Atelopus chiriquiensis*. *Copeia* **1996**, 428–432.

Jaslow A. P., Hetherington, T. E. & Lombard, R. E. (1988). Structure and function of the amphibian middle ear. In *The evolution of the amphibian auditory system*, pp. 69–91. Wiley, New York.

Javari M. & Torki, F. (2009). Notes on morphology, ecology, behavior and systematics of *Bufo luristanicus* Schmidt, 1952. *Herpetozoa* **21**, 171–178.

Jiménes de la Espada M. (1875). *Vertebrados del viaje al Pacífico verificado de 1862 a 1865 por una comisión de naturalistas enviada por el gobierno españo. Batracios*. Imprenta de Miguel Ginesta, Madrid.

Jungfer K-H. (2010). The taxonomic status of some spiny–backed treefrogs, genus *Osteocephalus* (Amphibia: Anura: Hylidae). *Zootaxa* **2407**, 28–50.

Jungfer K.-H., Faivovich, J., Padial, J. M., Castroviejo-Fisher, S., Lyra, M. M., Berneck, B. V. M., Iglesias, P. P., Kok, P. J. R., MacCulloch, R. D., Rodrigues, M. T., Verdade, V. K., Torres Gastello, C. P., Chaparro, J. C., Valdujo, P. H., Reichle, S., Moravec, J., Gvozdık, V., Gagliardi-Urrutia, G., Ernst, R., De la Riva, I., Means, D. B., Lima, A. P., Señaris, J. C., Wheeler, W. C. & Haddad, C. F. B. (2013). Systematics of spiny-backed treefrogs (Hylidae: *Osteocephalus*): an Amazonian puzzle. *Zoologica Scripta* **42**, 351–380.

Jungfer K.-H., Ron, S., Seipp, R. & Almendariz, A. (2000). Two new species of hylid frogs, genus *Osteocephalus*, from Amazonian Ecuador. *Amphibia–Reptilia* **21**, 327–340.

Kaiser H., Green, D. M. & Schmid, M. (1994). Systematics and biogeography of Eastern Caribbean frogs (Leptodactylidae: *Eleutherodactylus*), with the description of a new species from Dominica. *Canadian Journal of Zoology* **72**, 2217–2237.

Kaiser H., Hardy, J. D. & Green, D. M. (1994). Taxonomic status of Caribbean and South American frogs currently ascribed to *Eleutherodactylus urichi* (Anura: Leptodactylidae). *Copeia* **1994**, 780–796.

Kattan G. (1986). Nueva especie de rana (*Atelopus*) de los Farallones de Cali, Cordillera Occidental de Colombia. *Caldasia* **14**, 651–657.

Keith R. (1968). A new species of *Bufo* from Africa, with comments on the toads of the *Bufo regularis* complex. *American Museum Novitates* **2345**, 1–22.

Kenny J. S. (1969). The Amphibia of Trinidad. *Studies of the Fauna of Curaçao and other Caribbean Islands* **29**, 1–78.

Kerney R., Meegaskumbura, M., Manamendra-Arachchi, K. & Hanken, J. (2007). Cranial ontogeny in *Philautus silus* (Anura: Ranidae: Rhacophorinae) reveals few similarities with other direct-developing anurans. *Journal of morphology* **268**, 715–725.

Kizirian D., Coloma, L. A. & Paredes–Recalde, A. (2003). A new treefrog (Hylidae: *Hyla*) from southern Ecuador and a description of its antipredator behavior. *Herpetologica* **59**, 339–349.

Klappenbach M. A. (1968). Notas Herpetológicas, IV. El género *Melanophryniscus* (Amphibia, Salientia) en el Uruguay, con descripcion de dos nuevas especies. *Comunicaciones Zoológicas del Museo de Historia Natural de Montevideo* **9**, 377–379.

Köhler J. (2000a). Amphibian diversity in Bolivia: a study with special reference to montane forest regions. *Bonner Zoologische Monographien* **48**, 1–243.

Köhler J. (2000b). New species of *Eleutherodactylus* (Anura: Leptodactylidae) from cloud forest of Boliva. *Copeia* **2000**, 516–520.

Köhler J. & Jungfer, K.-H. (1995). Eine neue Art und ein Erstnachweis von Fröschen der Gattung Eleutherodactylus aus Bolivien. *Salamandra* **31**, 149–156.

Köhler J. & Lötters, S. (1999). New species of the *Eleutherodactylus unistrigatus* group (Amphibia: Anura: Leptodactylidae) from montane rain forest of Bolivia. *Copeia* **1999**, 422–427.

Kok P. J. R. (2009). A new species of *Oreophrynella* (Anura: Bufonidae) from the Pantepui region of Guyana, with notes on *O. macconnelli* Boulenger, 1900. *Zootaxa* **2071**, 35–49.

Kok P. J. R. & Kalamandeen, M. (2008). Introduction to the taxonomy of the amphibians of Kaieteur National Park, Guyana. *Abc Taxa* **5**, 1–278.

Kok P. J. R., MacCulloch R. D., Gaucher P., Poelman E. H., Bourne G. R., Lathrop A. & Lenglet G. L. (2006) A new species of *Colostethus* (Anura, Dendrobatidae) from French Guiana with a redescription of Colostethus beebei (Noble, 1923) from its type locality. *Phyllomedusa: Journal of Herpetology* **5**, 43–66.

Kou Z. (1984). Preliminary reports on the herpetofauna of Shuitang and Zhelong districts of the eastern slope of Mt. Ailao, with description of a new species. *Acta Herpetol. Sinica* **3**, 39–45.

Kraus F. (2013). A new species of *Hylophorbus* (Anura: Microhylidae) from Papua New Guinea. *Current Herpetology* **32**, 102–111.

Kuramoto M., Joshy, S. H., Kurabayashi, A. & Sumida, M. (2007). The genus *Fejervarya* (Anura: Ranidae) in central Western Ghats, India, with descriptions of four new cryptic species. *Current Herpetology* **26**, 81–105.

Kuramoto, M. & Joshy, S. H. (2003) Two new species of the genus *Philautus* (Anura: Rhacophoridae) from the Western Ghats, southwestern India. *Current Herpetology* **22**, 51–60.

Kwet A. (2000). The genus *Pseudis* (Anura: Pseudidae) in Rio Grande do Sul, southern Brazil, with description of a new species. *Amphibia–Reptilia* **21**, 39–55.

La Marca E. & Mijares-Urrutia, A. (1996). Taxonomy and geographic distribution of a northwestem Venezuelan toad (Anura, Bufonidae, *Bufo sternosignatus*). *Alytes* **14**, 101–114.

La Marca E. (1983). A new frog of the genus *Atelopus* (Anura: Bufonidae) from a Venezuelan cloud forest. *Contributions in Biology and Geology, Milwaukee Public Museum* **54**, 1–12.

La Marca E. (1996 "1994"). Descripción de una nueva especie de *Atelopus* (Amphibia: Anura: Bufonidae) de la selva andina nublada de Venezuela. *Memoria de la Sociedad de Ciencias Naturales de La Salle* **54**, 101–108.

La Marca E., García-Pérez, J. E. & Renjifo, J. M. (1990 "1989"). Una nueva especie de *Atelopus* (Amphibia: Anura: Bufonidae) del Páramo de Tamá, Estado Apure, Venezuela. *Caldasia* **16**, 97–104.

Langone J. A. (1994). Ranas y sapos del Uruguay (Reconocimiento y aspectos biologicos). *Museo Damaso Antonio Larrañaga. Serie Divulgación* n. 5. 123 pp.

Langone J. A. (1997). Caracterización de *Hyla guentheri* Boulenger, 1886 (Amphibia, Anura, Hylidae). *Cuadernos de Herpetologia* **11**, 13–20.

Langone J. A., Segalla, M. V., Bornschein, M. & de Sa, R. O. (2008). A new reproductive mode in the genus *Melanophryniscus* Gallardo, 1961 (Anura: Bufonidae) with description of a new species from the state of Paraná, Brazil. *South American Journal of Herpetology* **3**, 1–9.

Lanza B., Nascetti, G., Capula, M. & Bullini, L. (1984). Genetic relationships among West Mediterranean *Discoglossus* with the description of a new species (Amphibia Salientia Discoglossidae). *Monitore Zoologico Italiano* **18**, 133–152.

Largen M. J. & Drewes, R. C. (1989). A new genus and species of brevicipitine frog (Amphibia Anura Microhylidae) from high altitude in the mountains of Ethiopia. *Tropical Zoology* **2**, 13–30.

Lathrop A. & MacCulloch, R. D. (2007). A new species of *Oreophrynella* (Anura: Bufonidae) from Mount Ayanganna, Guyana. *Herpetologica* **63**, 87–93.

Lathrop A., Murphy R. W., Orlov N. L. & Ho C. T. (1998) Two new species of *Leptobrachium* (Anura: Megophryidae) from the Central Highlands of Vietnam with a redescription of *Leptobrachium chapaense*. *Russian Journal of Herpetology* **5**, 51–60.

Lathrop A., Murphy, R. W., Orlov, N. & Ho, C. T. (1998). Two new species of *Leptolalax* (Anura: Megophryidae) from northern Vietnam. *Amphibia-Reptilia* **19**, 253–267.

Laurent R. F. & Combaz, J. (1950). Sur l'attribution générique de certains Batraciens appartenant à la sous-famille des Hyperoliinae. *Revue de Zoologie et de Botanique Africaines* **43**, 269–280.

Laurent R. F. (1940). Contribution à l'ostéologie et à la systématique des ranides africains - Première note. *Revue de Zoologie et de Botanique Africaines* **34**, 74–97.

Laurent R. F. (1941a). Contribution à l'ostéologie et à la systématique des Ranides africains - Deuxieme note - IV.- Phrynobatrachinae. *Revue de Zoologie et de Botanique Africaines* **34**, 192–235.

Laurent R. F. (1941b). Contribution à l'osteologie et a la systematique des Rhacophorides africains - Premiere note. *Revue de Zoologie et de Botanique Africaines* **35**, 85–110.

Laurent R. F. (1942). Note sur les procoellens firmisternes (Batrachia Anura). *Bulletin du Musée Royal d'Histoire Naturelle de Belgique* **18**, 1–20.

Laurent R. F. (1943a). Contribution a l'Etude des genres *Megalixalus* et *Hyperolius*. *Bulletin du Musée Royal d'Histoire Naturelle de Belgique* **29**, 1–20.

Laurent R. F. (1943b). Sur l'Ostéologie de deux ranides exotiques. *Bulletin du Musée Royal d'Histoire Naturelle de Belgique* **19**, 1–4.

Laurent R. F. (1944). Contribution a l'osteologie systematique des rhacophorides africains. *Revue de Zoologie et de Botanique Africaines* **38**, 110–138.

Laurent R. F. (1986). Sous classe de Lissamphibiens: Lissamphibia Systématique. In *Traité de Zoologie: Anatomie, Systématique, Biologie, Tome XIV*, vol. Fascicle 1B -Batrachiens (ed. P.-P. Grassé and M. Delsol), pp. 594–797. Masson, Paris.

Laurent R. F., Lavilla, E. O. & Teran, E. M. (1986). Contribucion al conocimiento del genero *Gastrotheca* Fitzinger (Amphibia: Anura: Hylidae) en Argentina. *Acta Zoológica Lilloana* **38**, 171–210.

Lavilla E. O. & Ergueta Sandoval, P. (1995). Una nueva especie de *Telmatobius* (Anura: Leptodactylidae) del sudoeste de Bolivia. *Ecología en Bolivia* **24**, 91–101.

Lavilla E. O. & Ergueta Sandoval, P. (1999). A new Bolivian species of the genus *Telmatobius* (Anura: Leptodactylidae) with a humeral spine. *Amphibia-Reptilia* **20**, 55–64.

Lavilla E. O., Caramaschi, U., Langone, J. A., Pombal, J. P. & de Sa, R. O. (2013). The identity of *Rana margaritifera* Laurenti, 1768 (Anura, Bufonidae). *Zootaxa* **3646**, 251–264.

Leão A. T. & Cochran, D. M. (1952). Revalidation and re-description of *Bufo ocellatus* Günther, 1858 (Anura: Bufonidae). *Memorias do Instituto Butantan* **24**, 271–280.

Lehr E. (2006). Taxonomic status of some species of peruvian *Phrynopus* (Anura: Leptodactylidae), with the description of a new species from the Andes of southern Peru. *Herpetologica* **62**, 331–347.

Lehr E. & Catenazzi, A. (2008). A new species of *Bryophryne* (Anura: Strabomantidae) from southern Peru. *Zootaxa* **1784**, 1–10.

Lehr E. & Catenazzi, A. (2009). Three new species of *Bryophryne* (Anura: Strabomantidae) from the region of Cusco, Peru. *South American Journal of Herpetology* **4**, 125–138.

Lehr E. & Catenazzi, A. (2010). Two new species of *Bryophryne* (Anura: Strabomantidae) from high elevations in southern Peru (Region of Cusco). *Herpetologica* **66**, 308–319.

Lehr E., Köhler, G., Aguilar, C. & Ponce, E. (2001). New Species of *Bufo* (Anura: Bufonidae) from Central Peru. *Copeia* **2001**, 216–223.

Lehr E., Lötters, S. & Lundberg, M. (2008). A new species of *Atelopus* (Anura: Bufonidae) from the Cordillera Oriental of Central Peru. *Herpetologica* **64**, 368–378.

Lehr E., Pramuk, J. B. & Lundberg, M. (2005). A new species of *Bufo* (Anura: Bufonidae) from Andean Peru. *Herpetologica* **61**, 308–318.

Lehr E., Pramuk, J. B., Hedges, S. B. & Córdova, J. H. (2007). A new species of arboreal *Rhinella* (Anura: Bufonidae) from Yanachaga-Chemillén National Park in central Peru. *Zootaxa* **1662**, 1–14.

Lescure J. & Marty, C. (2000). *Atlas des amphibiens de Guyane*. Publications Scientifiques du M.N.H.N., Paris.

Liem S. S. (1970) The morphology, systematics, and evolution of the Old World treefrogs (Rhacophoridae and Hyperoliidae). *Fieldiana, Zoology* **57**, 1–145.

Lima A. P., Caldwell, J. P. & Strussmann, C. (2009). Redescription of *Allobates brunneus* (Cope) 1887 (Anura: Aromobatidae: Allobatinae), with a description of the tadpole, call, and reproductive behavior. *Zootaxa* **1988**, 1–16.

Lima A. P., Magnusson, W. E., Menin, M., Erdtmann, L. K., Rodrigues, D. J., Keller, C. & Hödl, W. (2006). *Guide to the frogs of Reserva Adolpho Ducke, Central Amazonia*. Áttema Design Editorial, Manaus.

Lima A. P., Menin, M. & Araújo, M. C. (2007). A new species of *Rhinella* (Anura: Bufonidae) from Brazilian Amazon. *Zootaxa* **1663**, 1–15.

Lindquist E. D. & Hetherington, T. E. (1996). Field studies on visual and acoustic signaling in the "earless" panamanian golden frog, *Atelopus zeteki*. *Journal of Herpetology* **30**, 347–354.

Litvinchuk S. N., Borkin, L. J., Skorinov, D. V. & Rosanov, J. M. (2008). A new species of common toads from the Talysh Mountains, south-eastern Caucasus: genome size, allozyme, and morphological evidences. *Russian Journal of Herpetology* **15**, 19–43.

Liu C.-C. (1936). Secondary sex characters of Chinese frogs and toads. *Field Museum of Natural History* **22**, 115–156.

Liu C.-C. (1950). Amphibians of Western China. *Fieldiana: Zoology Memoirs* **2**, 1–400 + 10 plates.

Liu C.-C., Hu, S.-Q., Fei, L. & Huang, C.-C. (1973). On collections of amphibians from Hainan Island. *Acta Zoologica Sinica* **19**, 385–404.

Loader S. P., Poynton, J. C., Davenport, T. R. B. & Rödel, M.-O. (2009). Re-description of the type series of *Nectophrynoides viviparus* (Bufonidae), with a taxonomic reassessment. *Zootaxa* **2304**, 41–50.

Loftus-Hills J. J. (1973). Neural mechanisms underlying acoustic behaviour of the frog *Pseudophryne semimarmorata* (Anura: Leptodactylidae). *Animal Behaviour* **21**, 781–787.

Lötters S. & de la Riva, I. (1998). Redescription of *Atelopus tricolor* Boulenger from southeastern Peru and adjacent Bolivia, with comments on related forms. *Journal of Herpetology* **32**, 481-488.

Lötters S. & Henzl, M. J. (2000). A new species of *Atelopus* (Anura: Bufonidae) from the Serranía de Sira, Amazonian Peru. *Journal of Herpetology* **34**, 169–173.

Lötters S. & Köhler, J. (2000). A new toad of the *Bufo typhonius* complex. *Spixiana* **23**, 293–303.

Lötters S. (2003). On the systematics of the harlequin frogs (Amphibia: Bufonidae: *Atelopus*) from Amazonia. III: A new, remarkably dimorphic species from the Cordillera Azul, Peru. *Salamandra* **39**, 169–180.

Lötters S., Böhme, W. & Günther, R. (1998). Notes on the type material of the neotropical harlequin frogs *Atelopus varius* (Lichtenstein & Martens, 1856) and *Atelopus cruciger* (Lichtenstein & Martens, 1856) deposited in the Museum fur Naturkunde of Berlin (Anura, Bufonidae). *Mitteilungen aus dem Zoologischen Museum in Berlin* **74**, 173–184.

Lötters S., Haas, W., Schick, S. & Böhme, W. (2002a). On the systematics of the harlequin frogs (Amphibia: Bufonidae: *Atelopus*) from Amazonia. I: description of a new species from the Cordillera Azul, Peru. *Salamandra* **38**, 95–104.

Lötters S., Haas, W., Schick, S. & Böhme, W. (2002b). On the systematics of the harlequin frogs (Amphibia: Bufonidae: *Atelopus*) from Amazonia. II: Redescription of *Atelopus pulcher* (Boulenger, 1882) from the eastern Andean versant in Peru. *Salamandra* **38**, 165–184.

Lötters S., La Marca, E., Vences, M. & Lannoo, M. J. (2004). Redescriptions of two toad species of the genus *Atelopus* from coastal Venezuela. *Copeia* **2004**, 222–234.

Lötters S., Schulte, R. & Duellman, W. E. (2005 "2004"). A new and critically endangered species of *Atelopus* from the Andes of northern Peru (Anura: Bufonidae). *Revista Española de Herpetología* **17**, 101–109.

Lötters S., van der Meijden, A., Coloma, L. A., Boistel, R., Cloetens, P., Ernst, R., Lehr, E. & Veith, M. (2011). Assessing the molecular phylogeny of a near extinct group of vertebrates: the neotropical harlequin frogs (Bufonidae; *Atelopus*). *Systematic and Biodiversity* **2011**, 1.

Loveridge A. (1932). Eight new toads of the genus *Bufo* from east and central Africa. *Occasional Papers of the Boston Society of Natural History* **8**, 43–54.

Loveridge A. (1953). Zoological results of a fifth expedition to East Africa. IV. Amphibians from Nyasaland and Tete. *Bulletin of the Museum of Comparative Zoology* **110**, 325–406.

Lu S.-Q. & Yang, D.-T. (1995). A study on morphological similarity between the genera *Nanorana* and *Altirana* (Amphibia, Anura, Ranidae). *Asiatic Herpetological Research* **6**, 69–72.

Lutz A. & Lutz, B. (1939). I. Notes on the genus *Phyllomedusa* Wagler. A) Observations on small Phyllomedusae without vomerine teeth or conspicuous parotids found in the region of Rio de Janeiro. B) *Phyllomedusa bahiana* Lutz. *Annaes da Academia Brasileira de Sciencias* **11**, 219–263.

Lutz B. (1958). Anfíbios novos e raros das serras costeiras do Brasil. *Memorias do Instituto Butantan* **56**, 373–399.

Lutz B. (1966). *Pithecopus ayeaye*, a new Brazilian hylid with vertical pupils and grasping feet. *Copeia* **1966**, 236–240.

Lutz B. (1973). *Brazilian Species of Hyla*. University of Texas Press, Austin.

Lynch J. D. (1965a). A new species of *Eleutherodactylus* from Guadeloupe, West Indies. *Breviora* **220**, 1–7.

Lynch J. D. (1965b). A review of the eleutherodactylid frog genus *Microbatrachylus* (Leptodactylidae). *Natural History Miscellanea* **182**, 1–12.

Lynch J. D. (1968). Two new frogs of the genus *Eleutherodactylus* from eastern Ecuador (Amphibia: Leptodactylidae). *Journal of Herpetology* **2**, 129–135.

Lynch J. D. (1969). Identity of two Andean *Eleutherodactylus* with the description of a new species (Amphibia: Leptodactylidae). *Journal of Herpetology* **3**, 135–143.

Lynch J. D. (1971). Evolutionary relationships, osteology, and zoogeography of leptodactyloid frogs. *Miscellaneous Publications of the Museum of Natural History, University of Kansas* **53**, 1–238.

Lynch J. D. (1972). Generic partitioning of the South American leptodactylid frog genus *Eupsophus* Fitzinger, 1843 (sensu lato). *Bulletin of the Southern California Academy of Sciences* **71**, 2–11.

Lynch J. D. (1973). A new narrow-toed frog from Andean Ecuador (Leptodactylidae: *Eleutherodactylus*). *Copeia* **1973**, 222–225.

Lynch J. D. (1974a). New species of frogs (Leptodactylidae: *Eleutherodactylus*) from the Amazonian lowlands of Ecuador. *Occasional Papers of the Museum of Natural History, University of Kansas* **31**, 1–22.

Lynch J. D. (1974b). A new species of leptodactylid frog (*Ischnocnema*) from the Cordillera del Condor in Ecuador. *Journal of Herpetology* **8**, 85–87.

Lynch J. D. (1975a). The identity of the frog *Eleutherodactylus conspicillatus* (Günther), with descriptions of two related species from northwestern South America (Amphibia, Leptodactylidae). *Contributions in Science. Natural History Museum of Los Angeles County* **272**, 1–19.

Lynch J. D. (1975b). A review of the andean leptodactylid frog genus *Phrynopus*. *Occasional Papers of the Museum of Natural History, The University of Kansas* **35**, 1–51.

Lynch J. D. (1975c). A review of the broadheaded eleutherodactyline frogs of South America (Leptodactylidae). *Occasional Papers of the Museum of Natural History, University of Kansas* **38**, 1–46.

Lynch J. D. (1976a). New species of frogs (Leptodactylidae, *Eleutherodactylus*) from the Pacific Versant of Ecuador. *Occasional Papers of the Museum of Natural History, University of Kansas* **55**, 1–33.

Lynch J. D. (1976b). Two new species of frogs of the genus *Euparkerella* (Amphibia: Leptodactylidae) from Ecuador and Peru. *Herpetologica* **32**, 48–53.

Lynch J. D. (1978). A re-assessment of the telmatobiine leptodactylid frogs of Patagonia. *Occasional Papers of the Museum of Natural History, University of Kansas* **72**, 1–57.

Lynch J. D. (1979a). The identity of *Eleutherodactylus vertebralis* (Boulenger) with the description of a new species from Colombia and Ecuador (Amphibia: Leptodactylidae). *Journal of Herpetology* **13**, 411–418.

Lynch J. D. (1979b). Leptodactylid frogs of the genus *Eleutherodactylus* from the Andes of southern Ecuador. *Miscellaneous Publication. Museum of Natural History, University of Kansas* **66**, 1–62.

Lynch J. D. (1979c). A new frog species of the *Eleutherodactylus fitzingeri* group from the Pacific Andean versant in Ecuador. *Herpetologica* **35**, 228–233.

Lynch J. D. (1980a). Systematic status and distribution of some poorly known frogs of the genus *Eleutherodactylus* from the Chocoan lowlands of South America. *Herpetologica* **36**, 175–189.

Lynch J. D. (1980b). A taxonomic and distributional synopsis of the Amazonian frogs of the genus *Eleutherodactylus*. American Museum novitates; no. 2696. *American Museum Novitates* **2696**, 1–24.

Lynch J. D. (1980c). Two new species of earless frogs allied to *Eleutherodactylus surdus* (Leptodactylidae) from the Pacific slopes of the Ecuadorian Andes. *Proceedings of the Biological Society of Washington* **93**, 327–338.

Lynch J. D. (1981a). Leptodactylid frogs of the genus *Eleutherodactylus* in the Andes of northern Ecuador and adjacent Colombia. *Miscellaneous Publication. Museum of Natural History, University of Kansas* **72**, 1–46.

Lynch J. D. (1981b). Two new species of *Eleutherodactylus* from western Colombia (Amphibia: Anura: Leptodactylidae). *Occasional Papers of the Museum of Zoology, University of Michigan* **697**, 1–12.

Lynch J. D. (1984). New frogs (Leptodactylidae: *Eleutherodactylus*) from cloud forest of the northern Cordillera Oriental, Colombia. *Contributions in Biology and Geology. Milwaukee Public Museum* **60**, 1–19.

Lynch J. D. (1985). A new species of *Eleutherodactylus* from western Panama (Amphibia: Leptodactylidae). *Herpetologica* **41**, 443–447.

Lynch J. D. (1986). New species of minute leptodactylid frogs from the Andes of Ecuador and Peru. *Journal of Herpetology* **20**, 423–431.

Lynch J. D. (1989). Intrageneric relationships of mainland *Eleutherodactylus* (Leptodactylidae). I. A review of the frogs assigned to the *Eleutherodactylus discoidalis* species group. Contributions in Biology and Geology. *Milwaukee Public Museum* **79**, 1–25.

Lynch J. D. (1993). A new harlequin frog from the Cordillera Oriental of Colombia (Anura, Bufonidae, *Atelopus*). *Alytes* **11**, 77–87.

Lynch J. D. (1994). A new species of high-altitude frog (*Eleutherodactylus*: Leptodactylidae) from the cordillera oriental of Colombia. *Revista de la Academia Colombiana de Ciencias Exactas, Fisicas y Naturales* **19**, 195–203.

Lynch J. D. (1996). New frogs of the genus *Eleutherodactylus* (Family Leptodactylidae) from the San Antonio region of the Colombian Cordillera Occidental. *Revista de la Academia Colombiana de Ciencias Exactas, Físicas y Naturales* **20**, 331–345.

Lynch J. D. (1998). New species of *Eleutherodactylus* from the Cordillera Occidental of western Colombia with a synopsis of the distributions of species in western Colombia. *Revista de la Academia Colombiana de Ciencias Exactas, Físicas y Naturales* **22**, 117–148.

Lynch J. D. (2000). The relationships of an ensemble of Guatemalan and Mexican frogs (*Eleutherodactylus*: Leptodactylidae: Amphibia). *Revista de la Academia Colombiana de Ciencias Exactas, Físicas y Naturales* **24**, 129–156.

Lynch J. D. & Ardila-Robayo, M. C. (1999). The *Eleutherodactylus* of the *taeniatus* complex in western Colombia: taxonomy and distribution. *Revista de la Academia Colombiana de Ciencias Exactas, Físicas y Naturales* **23**, 615–624.

Lynch J. D. & Burrowes, P. A. (1990). The frogs of the genus *Eleutherodactylus* (Family Leptodactylidae) at the La Planada Reserve in southwestern Colombia with descriptions of eight new species. *Occasional Papers of the Museum of Natural History, University of Kansas* **136**, 1–31.

Lynch J. D. & Duellman W. E. (1973) A review of the centrolenid frogs of Ecuador, with descriptions of new species. *Occasional Papers of the Museum of Natural History, University of Kansas* **16**, 1–66.

Lynch J. D. & Duellman, W. E. (1980). The *Eleutherodactylus* of the Amazonian slopes of the ecuadorian Andes (Anura: Leptodactylidae). *Miscellaneous Publication. Museum of Natural History, University of Kansas* **69**, 1–86.

Lynch J. D. & Duellman, W. E. (1997). Frogs of the genus *Eleutherodactylus* in western Ecuador. *University of Kansas Natural History Museum, Special Publication* **23**, 1–236.

Lynch J. D. & Hoogmoed, M. S. (1977). Two new species of *Eleutherodactylus* (Amphibia, Leptodactylidae) from northeastern South America. *Proceedings of the Biological Society of Washington* **90**, 424–439.

Lynch J. D. & McDiarmid, R. W. (1987). Two new species of *Eleutherodactylus* (Amphibia: Anura: Leptodactylidae) from Bolivia. *Proceedings of the Biological Society of Washington* **100**, 337–346.

Lynch J. D. & Myers, C. W. (1983). Frogs of the *fitzingeri* group of *Eleutherodactylus* in eastern Panama and Chocoan South America (Leptodactylidae). *Bulletin of the American Museum of Natural History* **175**, 481–572.

Lynch J. D. & Renjifo, J. M. (1990). Two new toads (Bufonidae: *Rhamphophryne*) from the northern Andes of Colombia. *Journal of Herpetology* **24**, 364–371.

Lynch J. D. & Renjifo, J. M. (2001). *Guía de anfibios y reptiles de Bogotá y sus alrededores*. Alcaldía Mayor de Bogotá. Departamento Técnico Administrativo del Medio Ambiente (DAMA), Bogotá, Colombia.

Lynch J. D. & Rueda, J. V. (1999). New species of frogs from low and moderate elevations from the Caldas transect of the easter flank of the cordillera Central. *Revista de la Academia Colombiana de Ciencias Exactas, Físicas y Naturales* **23**, 307–314.

Lynch J. D. & Rueda-Almonacid, J. V. (1998). New frogs of the genus *Eleutherodactylus* from the eastern flank of the northern cordillera central of Colombia. *Revista de la Academia Colombiana de Ciencias Exactas, Físicas y Naturales* **85**, 561–570.

Lynch J. D. & Ruiz-Carranza, P. M. (1981). A new species of toad (Anura: Bufonidae) from the Cordillera Occidental in southern Colombia. *Lozana* **33**, 1–7.

Lynch J. D. & Ruiz-Carranza, P. M. (1983). New frogs of the genus *Eleutherodactylus* from the andes of Southern Colombia. *Transactions of the Kansas Academy of Science* **86**, 99–112.

Lynch J. D. & Smith, H. M. (1966). A new toad from western Mexico. *The Southwest Naturalist* **11**, 19–23.

Lynn W. G. (1954). Description of a new frog of the genus *Eleutherodactylus* from Jamaica, BWI. *B.W.I. Journal of the Washington Academy of Sciences* **44**, 400–402.

MacCulloch R. D., LathrOP; A., Kok, P. J. R., Minter, L. R., Khan, S. Z. & Barrio-Amóros, C. L. (2008). A new species of *Adelophryne* (Anura: Eleutherodactylidae) from Guyana, with additional data on *A. gutturosa*. *Zootaxa* **1884**, 36–50.

Maciel N. M., Brandao, R. A., Campos, L. A. & Sebben, A. (2007). A large new species of *Rhinella* (Anura: Bufonidae) from Cerrado of Brazil. *Zootaxa* **1627**, 23–39.

Maglia A. M. (1998) Phylogenetic relationships of extant pelobatoid frogs (Anura: Pelobatoidea). Scientific Papers, Natural History Museum, University of Kansas 10, 1–19.

Maglia A. M., Pugener, L. A. & Mueller, J. M. (2007). Skeletal morphology and postmetamorphic ontogeny of *Acris crepitans* (Anura: Hylidae): a case of miniaturization in frogs. *Journal of morphology* **268**, 194–223.

Manamendra-Arachchi K. & Pethiyagoda R. (2005) The Sri Lankan shrub-frogs of the genus *Philautus* Gistel, 1848 (Ranidae: Rhacophorinae), with description of 27 new species. *Raffles Bulletin of Zoology* **12**, 163–303.

Manamendra-Arachchi K. & Pethiyagoda, R. (1998). A synopsis of the Sri Lankan Bufonidae (Amphibia: Anura), with description of two new species. *Journal of South Asian Natural History* **3**, 213–246.

Maneyro R., Arrieta, D. & de Sa, R. O. (2004). A new toad (Anura: Bufonidae) from Uruguay. *Journal of Herpetology* **38**, 161–165.

Maneyro R., Naya, D. E. & Baldo, D. (2008). A new species of *Melanophryniscus* (Anura, Bufonidae) from Uruguay. *Iheringia, Serie Zool.* **98**, 189–192.

Mathew R. & Sen, N. (2009). *Studies on little known amphibian species of North East India*. Records of the Zoological Survey of India: Occasional Paper, 293, Kolkata, India.

Matsui M. (1976). A new toad from Japan. *Contributions from the Biological Laboratory, Kyoto University* **25**, 1–10.

Matsui M. (1980). The status and relationships of the korean toad, *Bufo stejnegeri* Schmidt. *Herpetologica* **36**, 37–41.

Matsui M. (1984). Morphometric variation analyses and revision of the Japanese toads (genus *Bufo*, Bufonidae). *Contributions from the Biological Laboratory, Kyoto University* **26**, 209–428.

Matsui M. (1991). Original description of the brown frog from Hokkaido, Japan (genus Rana). *Japanese Journal of Herpetology* **14**, 63–78.

Matsui M., Nabhitabhata, J. & Panha, S. (1998). A new *Ansonia* from northern Thailand (Anura: Bufonidae). *Herpetologica* **54**, 448–454.

Matsui M., Nishikawa, K., Yeo, S. T. & Eto, K. (2012). Notes on a rare Bornean bufonid *Ansonia latidisca* Inger, 1966, with special reference to its phylogenetic position. *Current Herpetology* **31**, 87–96.

Matsui M., Yambun, P. & Sudin, A. (2007). Taxonomic relationships of *Ansonia anotis* Inger, Tan, and Yambun, 2001 and *Pedostibes maculatus* (Mocquard, 1890), with a description of a new genus (Amphibia, Bufonidae). *Zoological Science* **24**, 1159–66.

McCranie J. R. & Wilson, L. D. (1993). Taxonomic changes associated with the names *Hyla spinipollex* Schmidt and *Ptychohyla merazi* Wilson and McCranie (Anura, Hylidae). *The Southwestern Naturalist* **38**, 100–104.

McCranie J. R. & Wilson, L. D. (2000). A new species of high-crested toad of the *Bufo valliceps* group from North-Central Honduras. *Journal of Herpetology* **34**, 21–31.

McCranie J. R. & Wilson, L. D. (2002). *The amphibians of Honduras*. Society for the Study of Amphibians and Reptiles, Ithaca, NY.

McCranie J. R., Wilson, L. D. & William, K. L. (1989). A new genus and species of toad (Anura: Bufonidae) with a extraordinary stream-adapted tadpole from northern Honduras. *Occasional Papers of the Museum of Natural History, The University of Kansas* **129**, 1–18.

McDiarmid R. W. (1971). Comparative morphology and evolution of frogs of the genera *Atelopus*, *Dendrophryniscus*, *Melanophryniscus*, and *Oreophrynella*. *Bulletin of Los Angeles County Museum of Natural History* **12**, 1–66.

McDiarmid R. W. (1973). A new species of *Atelopus* (Anura, Bufonidae) from northeastern South America. *Los Angeles County Museum Contributions in Science* **240**, 1–12.

Meade-Waldo G. (1908). Description of a new species of toad from Sumatra. *Proceedings of the Zoological Society of London* **78**, 786–788.

Meegaskumbura M., Meegaskumbura S., Bowatte G., Manamendra-Arachchi K., Pethiyagoda R., Hanken J. & Schneider C. (2011) *Taruga* (Anura: Rhacophoridae), a new genus of foam-nesting tree frogs endemic to Sri Lanka. *Ceylon Journal of Science (Biological Sciences)* **39**, 75–94.

Meegaskumbura M., Senevirathne, G., Wijayathilaka, N., Jayawardena, B., Bandara, C., Manamendra-Arachchi, K. & Pethiyagoda, R. (2015). The Sri Lankan torrent toads (Bufonidae: Adenominae: *Adenomus*): species boundaries assessed using multiple criteria. *Zootaxa* **3911**, 245–261.

Melin D. (1941). Contribution to the knowledge of the Amphibia of South America. *Göteborgs Kungl. Vetenskaps-och Vitterhets-samhälles. Handlingar. Serien B, Matematiska och Naturvetenskapliga Skrifter* **1**, 1–71.

Mendelson J. R. (1994). A new species of toad (Anura: Bufonidae) from the lowlands of eastern Guatemala. *Occasional Papers of the Museum of Natural History, University of Kansas* **166**, 1–21.

Mendelson J. R. (1997a). A new species of toad (Anura: Bufonidae) from Oxaca, Mexico with comments on the status of *Bufo cavifrons* and *Bufo cristatus*. *Herpetologica* **53**, 268–286.

Mendelson J. R. (1997b). A new species of toad (Anura: Bufonidae) from the Pacific Highlands of Guatemala and Southern Mexico, with comments on the status of *Bufo valliceps macrocristatus*. *Herpetologica* **53**, 14–30.

Mendelson J. R. (1997c). Systematics of the *Bufo valliceps* group (Anura: Bufonidae) of Middle America, The University of Kansas.

Mendelson J. R. (2001). A review of the guatemalan toad *Bufo ibarrai* (Anura: Bufonidae), with distributional and taxonomic notes on *Bufo valliceps* and *Bufo coccifer*. In *Mesoamerican Herpetology: Systematics, Natural History, and Conservation* (ed. J. D. Johnson, R. G. Webb and O. Flores-Villela), pp. 10–19. The University of Texas at El Paso, El Paso, Texas.

Mendelson J. R. & Campbell, J. A. (1994). Two new species of the *Hyla sumichrasti* group (Amphibia: Anura: Hylidae) from Mexico. *Proccedings of the Biological Society of Washington* **107**, 397–409.

Mendelson J. R. & Campbell, J. A. (1999). The taxonomic status of populations referred to *Hyla chaneque* in southern Mexico, with the description of a new treefrog from Oaxaca. *Journal of Herpetology* **33**, 80–86.

Mendelson J. R. & Mulcahy, D. G. (2010). A new species of toad (Bufonidae: *Incilius*) from central Panama. *Zootaxa* **2396**, 61–68.

Mendelson J. R., da Silva, H. R. & Maglia, A. M. (2000). Phylogenetic relationships among marsupial frog genera (Anura: Hylidae: Hemiphractinae) based on evidence from morphology and natural history. *Zoological Journal of the Linnean Society* **128**, 125–148.

Mendelson J. R., Mulcahy, D. G., Snell, S., Acevedo, M. E. & Campbell, J. A. (2012). A new golden toad (Bufonidae: *Incilius*) from northwestern Guatemala and Chiapas, Mexico. *Journal of Herpetology* **46**, 473–479.

Mendelson J. R., Mulcahy, D. G., Williams, T. S. & Sites, J. W. (2011). A phylogeny and evolutionary natural history of mesoamerican toads (Anura: Bufonidae: *Incilius*) based on morphology, life history, and molecular data. *Zootaxa* **3138**, 1–34.

Mendelson J. R., Williams, B. L., Sheil, C. A. & Mulcahy, D. G. (2005). Systematics of the *Bufo coccifer* complex (Anura: Bufonidae) of Mesoamerica. *Scientific Papers of the Natural History Museum of the University of Kansas* **38**, 1–27.

Mendes C. V. M., Ruas, D. S., Lourenço-de-Moraes, R., Rödder, D. & Solé, M. (2012). The advertisement call of *Gastrotheca fissipes* Boulenger, 1888 (Anura, Hemiphractidae) with comments on its distribution. *Zootaxa* **3312**, 62–64.

Menegon M., Saldivio, S. & Loader, S. P. (2004). Five new species of *Nectophrynoides* Noble 1926 (Amphibia Anura Bufonidae) from the Eastern Arc Mountains, Tanzania. *Tropical Zoology* **17**, 97–121.

Menegon M., Saldivio, S., Ngalason, W. & Loader, S. P. (2007). A new dwarf forest toad (Amphibia: Bufonidae: *Nectophrynoides*) from the Ukaguru Mountains, Tanzania. *Zootaxa* **1541**, 31–40.

Menzies J. I. (2006). *The Frogs of New Guinea and the Solomon Islands*. Moscow: Pensoft.

Meriggio V., Veloso, A., Young, S. & Núñez, H. (2004). *Atelognathus jeinimenensis* n. sp. de Leptodactylidae para el sur de Chile. *Boletín del Museo Nacional de Historia Natural* **53**, 99–123.

Mijares-Urrutia A. & Arends, A. (2001). A new toad of the *Bufo margaritifer* complex (Amphibia: Bufonidae) from Northwestern Venezuela. *Herpetologica* **57**, 523–531.

Milto K. D. & Barabanov, A. V. (2011). An annotated catalogue of the amphibian types in the collection of the Zoological Institute, Russian Academy of Sciences, St. Petersburg. *Russian Journal of Herpetology* **18**, 137–153.

Miranda Ribeiro A. (1920). Algumas considerações sobre *Holoaden lüderwaldti* e generos correlatos. *Revista do Museu Paulista* **12**, 1–4.

Miranda-Ribeiro A. (1926). Notas para servirem ao estudo do Gymnobatrachios (Anura) Brasileiros. *Archivos do Museu Nacional do Rio de Janeiro* **27**, 1–227, 22 plates.

Miyata K. (1980). A new species of *Atelopus* (Anura: Bufonidae) from the cloud forests of northwestern Ecuador. *Breviora* **458**, 1–10.

Moravec J., Aparicio, J. & Kohler, J. (2006). A new species of tree frog, genus *Dendropsophus* (Anura: Hylidae), from the Amazon of northern Bolivia. *Zootaxa* **1327**, 23–40.

Moravec J., Lehr, E., Cusi, J. C., Córdova, J. H. & Gvoždík, V. (2014). A new species of the *Rhinella margaritifera* species group (Anura, Bufonidae) from the montane forest of the Selva Central, Peru. *ZooKeys* **371**, 35–56.

Moreno L. V. & Rivalta, V. (2007). Especie nueva de sapo del género *Bufo* (Anura: Bufonidae) de la Península de Zapata, Cuba. *Solenodon* **6**, 60–69.

Mueses Cisneros J. J. (2009). *Rhaebo haematiticus* (Cope 1862): Un complejo de especies, con redescripción de *Rhaebo hypomelas* (Boulenger 1913) y descripción de una nueva especie. *Herpetotropicos* **5**, 29–47.

Mueses-Cisneros J. J. (2007). A new species of *Rhaebo* (Anura: Bufonidae) from the Cordillera Occidental of Colombia. *Zootaxa* **1662**, 53–59.

Mueses-Cisneros J. J., Cisneros-Heredia, D. F. & McDiarmid, R. W. (2012). A new Amazonian species of *Rhaebo* (Anura: Bufonidae) with comments on *Rhaebo glaberrimus* (Günther, 1869) and *Rhaebo guttatus* (Schneider, 1799). *Zootaxa* **3447**, 22–40.

Mueses-Cisneros J. J., Yánez-Muñoz, M. H. & Guayasamin, J. M. (2010). Una nueva especie de sapo del género *Osornophryne* (Anura: Bufonidae) de las estribaciones amazónicas de los Andes de Ecuador. *Papeis Avulsos de Zoologia* **50**, 269–279.

Mueses-Cisneros J.J. & Anganoy-Criollo, M.A. (2008) Una nueva especie del grupo *Hyloscirtus larinopygion* (Amphibia: Anura: Hylidae) del Suroccidente de Colombia. *Papéis Avulsos de Zoología* **48**, 129–138.

Myers C. W. & Ford, L. S. (1986). On *Atopophrynus*, a recently described frog wrongly assigned to the Dendrobatidae. *American Museum Novitates* **2843**, 1–15.

Myers C. W. (1982). Spotted poison frogs: Descriptions of three new *Dendrobates* from western Amazonia, and resurrection of a lost species from "Chiriqui". *American Museum Novitates* **2721**, 1–23.

Myers C. W., Paolillo, A. O. & Daly, J. W. (1991). Discovery of a defensively malodorous and nocturnal frog in the family Dendrobatidae: Phylogenetic significance of a new genus and species from the Venezuelan Andes. *American Museum Novitates* **3002**, 1–33.

Myers C. W., Rodríguez L. O. & Icochea J. (1998) *Epipedobates simulans*, a new cryptic species of poison frog from southeastern Peru, with notes on *E. macero* and *E. petersi* (Dendrobatidae). *American Museum Novitates* **3238**, 1–20.

Myers G. S. & Carvalho, A. L. (1945). Notes on some new or little known brazilian amphibians, with an examination of the history of the Plata salamender, *Ensatina platensis*. *Boletim do Museu Nacional do Rio de Janeiro* **35**, 1–24.

Myers G. S. & Funkhouser, J. W. (1951). A new giant toad from Southwestern Colombia. *Zoologica* **36**, 279–282.

Narvaes P. & Rodrigues, M. T. (2009). Taxonomic revision of *Rhinella granulosa* species group (Amphibia, Anura, Bufonidae), with a description of a new species. *Arquivos de Zoologia* **40**, 1–73.

Nascimento, L. B., Caramaschi, U. & Cruz, C. A. G. (2005) Taxonomic review of the species groups of the genus *Physalaemus* Fitzinger, 1826 with revalidation of the genera *Engystomops* Jiménez-de-la-Espada, 1872 and *Eupemphix* Steindachner, 1863 (Amphibia, Anura, Leptodactylidae). *Arquivos do Museu Nacional* **63**, 297–320.

Nelson C. E. & Lescure, J. (1975). The taxonomy and distribution of *Myersiella* and *Synapturanus* (Anura: Microhylidae). *Herpetologica* **31**, 389–397.

Noble G. K. (1920). Two new batrachians from Colombia. *Bulletin of the American Museum of Natural History* **42**, 441–446.

Noble G. K. (1921). Five new species of salientia from South America. *American Museum Novitates*, **21**, 1–7.

Noble G. K. (1923). Six new batrachians from the Dominican Republic. *American Museum Novitates* **61**, 1–6.

Noble G. K. & Hassler, W. G. (1933). Two new species of frogs, five new species and a new race of lizards from the Dominican Republic. *American Museum Novitates* **652**, 1–17.

Nuñez J. J., Rabanal, F. E. & Formas, J. R. (2012). Description of a new species of *Eupsophus* (Amphibia: Neobatrachia) from the Valdivian Coastal range, Southern Chile: an integrative taxonomic approach. *Zootaxa* **3305**, 53–68.

Nussbaum R. A. & Wu, S.-H. (2007). Morphological assessments and phylogenetic relationships of the seychellean frogs of the family Sooglossidae (Amphibia: Anura). *Zoological Studies* **46**, 322–335.

Ohler A. & Dubois, A. (2006). Phylogenetic relationships and generic taxonomy of the tribe Paini (Amphibia, Anura, Ranidae, Dicroglossinae), with diagnoses of two new genera. *Zoosystema* **28**, 769–784.

Ohler A., Wollenberg, K. C., Grosjean, S., Hendrix, R., Vences, M., Ziegler, T. & Dubois, A. (2011). Sorting out Lalos: description of new species and additional taxonomic data on megophryid frogs from northern Indochina (genus *Leptolalax*, Megophryidae, Anura). *Zootaxa* **3147**, 1–83.

Onadeko A. B., Roedel, M.-O., Liedtke, H. C. & Barej, M. F. (2014). The rediscovery of Perret's toad, *Amietophrynus perreti* (Schiøtz, 1963) after more than 40 years, with comments on the species' phylogenetic placement and conservation status. *Zoosystematics and Evolution* **90**, 113–119.

O'Neill E. M. & Mendelson, J. R. (2004). Taxonomy of Costa Rican toads referred to *Bufo melanochlorus* Cope, with the description of a new species. *Journal of Herpetology* **38**, 487–494.

Orlov N. L., Poyarkov N. A., Vassilieva A. B., Ananjeva N. B., Nguyen T. T., Nguyen N. S. & Geissler P. (2012) Taxonomic notes on rhacophorid frogs (Rhacophorinae: Rhacophoridae: Anura) of southern part of Annamite Mountains (Truong Son, Vietnam), with description of three new species. *Russian Journal of Herpetology* **19**, 23–64.

Ortiz J. C., Ibarra-Vidal, H. & Formas, J. R. A new species of *Eupsophus* (Anura: Leptodactylidae) from Contulmo, Nahuelbuta range, southern Chile. *Proceedings of the Biological Society of Washington* **102**, 1031–1035 (1989).

Osorno-Muñoz M., Ardila-Robayo, M. C. & Ruiz-Carranza, P. M. (2001). Tres nuevas especies del género *Atelopus* A.M.C. Dumeril & Bibron 1841 (Amphibia: Bufonidae) de las partes altas de la cordillera oriental colombiana. *Caldasia* **23**, 509–522.

Ospina-Sarria J. J., Méndez-Narváez, J., Burbano-Yandi, C. E. & Bolívar-García, W. (2011). A new species of *Pristimantis* (Amphibia: Craugastoridae) with cranial crests from the Colombian Andes. *Zootaxa* **3111**, 37–48.

Ouboter P. E. & Jairam, R. (2012). *Amphibian of Suriname*. Brill, Leiden.

Padial J. M. & de la Riva, I. (2005). Rediscovery, redescription, and advertisement call of *Eleutherodactylus heterodactylus* (Miranda Ribeiro, 1937) (Anura: Leptodactylidae), and notes on other *Eleutherodactylus*. *Journal of Herpetology* **39**, 372–379.

Padial J. M. & De la Riva, I. (2009). Integrative taxonomy reveals cryptic Amazonian species of *Pristimantis* (Anura: Strabomantidae). *Zoological Journal of the Linnean Society* **155**, 97–122.

Padial J. M., Castroviejo-Fisher, S., Köhler, J., Domic, E. & De la Riva, I. (2007). Systematics of the *Eleutherodactylus fraudator* species group (Anura: Brachycephalidae). *Herpetological Monographs* **21**, 213–240.

Padial J. M., Chaparro, J. C. & de la Riva, I. (2007). A new species of the *Eleutherodactylus discoidalis* group (Anura: Brachycephalidae) from cloud forests of Peru. *Herpetologica* **63**, 114–122.

Padial J. M., Chaparro, J. C., Köhler, J. & de la Riva, I. (2009). Rediscovery, resurrection and redescription of *Rhinella leptoscelis* (Boulenger, 1912) (Anura: Bufonidae). *Zootaxa* **2115**, 56–64.

Padial J. M., Gonzáles, L. & de la Riva, I. (2005). A new species of the *Eleutherodactylus discoidalis* group (Anura: Leptodactylidae) from andean humid montane forest of Bolivia. *Herpetologica* **61**, 318–325.

Padial J. M., Reichle, S. & de la Riva, I. (2005). New species of *Ischnocnema* (Anura: Leptodactylidae) from the Andes of Bolivia. *Journal of Herpetology* **39**, 186–191.

Padial J. M., Reichle, S., McDiarmid, R. W. & de la Riva, I. (2006). A new species of arboreal toad (Anura: Bufonidae: *Chaunus*) from Madidi National Park, Bolivia. *Zootaxa* **1278**, 56–64.

Páez-Moscoso D. J., Guayasamin, J. M. & Yánez-Muñoz, M. (2011). A new species of Andean toad (Bufonidae, *Osornophryne*) discovered using molecular and morphological data, with a taxonomic key for the genus. *ZooKeys* **108**, 73–97.

Parker H. W. (1934). *A monograph of the frogs of the family Microhylidae*. Trustees of the British Museum, London.

Parker H. W. (1938). The vertical distribution of some reptiles and Amphibians in Southern Ecuador. *Journal of Natural History* **2**, 438–450.

Parker H. W. (1940). Undescribed anatomical structures and new species of reptiles and amphibians. *Journal of Natural History* **5**, 257–274.

Parker W. K. (1881). On the structure and development of the skull in the Batrachia. Part III. *Philosophical Transactions of the Royal Society of London* **172**, 1–266.

Pavan D., Narvaes, P. & Rodrigues, M. (2001) A new species of leptodactylid frog from the Atlantic Forests of southeastern Brazil with notes on the status and on the speciation of the *Hylodes* species groups. *Papéis Avulsos de Zoologia* **41**, 407–425.

Peloso P. L. V., Faivovich, J., Grant, T., Gasparini, J. L. & Haddad, C. F. B. (2012). An extraordinary new species of *Melanophryniscus* (Anura, Bufonidae) from southeastern Brazil. *American Museum Novitates* **3762**, 1–31.

Penna M., Contreras, S. & Veloso, A. (1983). Acoustical repertoires and morphological differences in the ear of two *Alsodes* species (Amphibia: Leptodactylidae). *Canadian Journal of Zoology* **61**, 2369–2376.

Pereyra M. O., Cardozo, D. E., Baldo, J. & Baldo, D. (2014). Description and phylogenetic position of a new species of *Oreobates* (Anura: Craugastoridae) from Northwestern Argentina. *Herpetologica* **70**, 211–227.

Perret J. L. & Amiet, J. L. (1971). Remarques sur les *Bufo* (Amphibiens Anoures) du Cameroun. *Annales de la Faculte des Sciences du Cameroun* **5**, 47–55.

Perret J. L. (1972). Les especes des genres *Wolterstorffina* et *Nectophrynoides* d'Afrique (Amphibia Bufonidae). *Annales de la Faculte des Sciences du Cameroun* **11**, 93–119.

Perret J.-L. (1977). Une nouvelle espèce de crapaud africain: "*Bufo danielae*" de Côte d'Ivoire. *Revue Suisse de Zoologie* **84**, 237–245.

Perry C. R. (2004). Phylogenetic analysis of the australian genus *Pseudophryne* (Myobatrachidae) using morphological characters, East Tennessee State University.

Peters J. A. (1973). The frog genus *Atelopus* in Ecuador (Anura: Bufonidae). *Smithsonian Contributions to Zoology* **145**, 1–49.

Peters W. C. H. (1882). Neue Batrachier (*Amblystoma Krausei, Nyctibatrachus sinensis, Bufo buchneri*). *Sitzungsberichte der Gesellschaft Naturforschender Freunde zu Berlin* **1882**, 145–148.

Pfeffer G. (1893). Ostafrikanische Reptilien und Amphibien, gesammelt von Herrn Dr. F. Stuhlmann im Jahre 1888 und 1889. *Jahrbuch der Hamburgischen Wissenschaftlichen Anstalten* **10**, 69–105.

Picariello O., Feliciello I., Scillitani G., Cataudo A., Maresca I. & Chinali G. (1999) Evidenze morfologiche e molecolari dell'identità tassonomica di *Rana macrocnemis, R. camerani* e *R. holtzi* (Anura: Ranidae). *Riv. Idrobiol.* **38**, 167–82.

Pillai R. S. & Pattabiraman, R. (1981). A new species of torrent toad (Genus: *Ansonia*) from Silent Valley, S. India. *Proceedings of the Indian Acad Sci* **90**, 203–208.

Pillai R. S. & Yadzani, G. M. (1973). *Bufoides*, a new genus for the rock-toad, *Ansonia meghalayana* Yadzani and Chanda, with notes on its ecology and breeding habits. *Journal of Zoology Soc. India* **25**, 65–70.

Pombal J. P. (2001). A new species of *Brachycephalus* (Anura: Brachycephalidae) from Atlantic Rain Forest of southeastern Brazil. *Amphibia-Reptilia* **22**, 179–185.

Pimenta B. V. S. & Caramaschi, U. (2007). New species of toad, genus *Frostius* Cannatella, 1986, from the Atlantic Rain Forest of Bahia, Brazil (Amphibia, Anura, Bufonidae). *Zootaxa* **1508**, 61–68.

Pombal J. P. & Gasparini, J. L. (2006). A new *Brachycephalus* (Anura: Brachycephalidae) from the Atlantic Rainforest of Espírito Santo, Southeastern Brazil. *South American Journal of Herpetology* **1**, 87–93.

Pombal J. P. & Haddad, C. F. B. (1992). Espécies de *Phyllomedusa* do grupo *burmeisteri* do Brasil oriental, com descrição de uma espécie nova (Amphibia: Hylidae). *Revista Brasileira de Biologia* **52**, 217–229.

Pombal J. P. & Haddad, C. F. B. (1999). Frogs of the genus *Paratelmatobius* (Anura: Leptodactylidae) with descriptions of two new species. *Copeia* **1999**, 1014–1026.

Ponssa M. L. (2008). Cladistic analysis and osteological descriptions of the frog species in the *Leptodactylus fuscus* species group (Anura, Leptodactylidae). *Journal of Zoological Systematics and Evolutionary Research* **46**, 249–266.

Poynton J. C. (1964). The Amphibia of South Africa. *Annals of the Natal Museum* **17**, 1–334.

Poynton J. C. (1977). A new *Bufo* and associated Amphibia from southern Tanzania. *Annals of the Natal Museum* **23**, 37–41.

Poynton J. C. (1991). Amphibians of Southeastern Tanzania, with special reference to *Stephopaedes* and *Mertensophryne* (Bufonidae). *Bulletin of the Museum of Comparative Zoology* **152**, 451–473.

Poynton J. C. & Broadley, D. G. (1987). Amphibia Zambesiaca 3. Rhacophoridae and Hyperoliidae. *Annals of the Natal Museum* **28**, 161–229.

Poynton J. C. & Broadley, D. G. (1988). Amphibia Zambesiaca 4. Bufonidae. *Annals of the Natal Museum* **29**, 447–490.

Poynton J. C. & Clarke, B. T. (1999). Two new species of *Stephopaedes* (Anura: Bufonidae) from Tanzania, with a review of the genus. *African Journal of Herpetology* **48**, 1–14.

Poynton J. C., Menegon, M. & Saldivio, S. (2005). *Bufo uzunguensis* of southern Tanzania (Amphibia: Anura): a history of confusion. *African Journal of Herpetology* **54**, 159–170.

Pramuk J. B. (2002). Combined evidence and cladistic relationships of West Indian toads (Anura: Bufonidae). *Herpetological Monographs* **16**, 121–151.

Pramuk J. B. (2006). Phylogeny of South American *Bufo* (Anura: Bufonidae) inferred from combined evidence. *Zoological Journal of the Linnean Society* **146**, 407–452.

Pramuk J. B. & Kadivar, F. (2003). A new species of *Bufo* (Anura: Bufonidae) from southern Ecuador. *Herpetologica* **59**, 270–283.

Pramuk J. B. & Lehr, E. (2005). Taxonomic status of *Atelophryniscus chrysophorus* McCranie, Wilson, and Williams, 1989 (Anura: Bufonidae) inferred from phylogeny. *Journal of Herpetology* **39**, 610–618.

Prigioni C. M. & Langone, J. A. (2000). Una nueva especie de *Melanophryniscus* Gallardo, 1961, de Argentina y Paraguay (Amphibia, Anura, Bufonidae). *Comunicaciones Zoológicas del Museo de Historia Natural de Montevideo* **12**, 1–11.

Pyburn W. F. & Lynch, J. D. (1981). Two little-known species of *Eleutherodactylus* (Amphibia: Leptodactylidae) from the Sierra de la Macarena, Colombia. *Proceedings of the Biological Society of Washington* **94**, 404–412.

Pyburn W. F. (1977). A new hylid frog (Amphibia, Anura, Hylidae) from the Vaupes river of Colombia with comments on related species. *Journal of Herpetology* **11**, 405–410.

Quah S. H. E., Grismer, L. L., Muin, M. A. & Anuar, S., M. S. (2011). Re-discovery and Re-description of *Ansonia penangensis* Stoliczka, 1870 (Anura: Bufonidae) from Penang Island, Malaysia. *Zootaxa* **2807**, 57–64.

Quezada C. L. C., Ayerza, M. S., Jara-Arancio, P., Lobos, G., Soto, E. & Méndez, M. A. (2008). Amphibia, Anura, Bufonidae, *Rhinella atacamensis*: Altitudinal distribution extension, new records and geographic distribution map. *Check List* **4**, 478–484.

Rabb G. B. & Rabb, M. S. (1963). On the behavior and breeding biology of the African pipid frog: *Hymenochirus boettgeri*. *Zeitschrift für Tierpsychologie* **20**, 215–241.

Ramaswami L. S. (1936). The morphology of the bufonid head. *Proceedings of the Zoological Society of London* **4**, 1157–1169.

Ramaswami L. S. (1944). The chondrocranium of two torrent-dwelling anuran tadpoles. *Journal of morphology* **74**, 347–373.

Rao C. R. N. (1937) On some new forms of Batrachia from S. India. Proceedings of the Indian Academy of Sciences. Section B **6**, 387–427.

Rao D.-Q., Wilkinson J. A. & Liu H.-N. (2006) A new species of *Rhacophorus* (Anura: Rhacophoridae) from Guangxi Province, china. *Zootaxa* **1258**, 17–31.

Recoder R. S., Teixeira, J., M., Cassimiro, J., Camacho, A. & Trefaut Rodrigues, M. (2010). A new species of *Dendrophryniscus* (Amphibia, Anura, Bufonidae) from the Atlantic Rainforest of southern Bahia, Brazil. *Zootaxa* **2642**, 36–44.

Reichle S., Lötters, S. & de la Riva, I. (2001). A new species of the *discoidalis* group of *Eleutherodactylus* (Anura, Leptodactylidae) from inner-andean dry valleys of Bolivia. *Journal of Herpetology* **35**, 21–26.

Reig O. A. (1972). *Macrogenioglottus* and the South American bufonid toads. In *Evolution in the genus Bufo* (ed. W. F. Blair), pp. 14–36. University of Texa Press, Austin & London.

Ribeiro L. F., Alves, A. C. R., Haddad, C. F. B. & Reis, S. F. (2005). Two new species of *Brachycephalus* Günther, 1858 from the state of Paraná, southern Brazil (Amphibia, Anura, Brachycephalidae). *Boletim do Museu Nacional* **519**, 10–18.

Rivera-Correa M. & Faivovich, J. (2013). A new species of *Hyloscirtus* (Anura: Hylidae) from Colombia with comments on *Hyloscirtus larinopygion* (Duellman, 1973). *Herpetologica* **69**, 298–313

Rivero J. A. (1959). Two new species of *Eleutherodactylus* from Puerto Rico. *Breviora* **103**, 1–6.

Rivero J. A. (1961). Salientia of Venezuela. *Bulletin of the Museum of Comparative Zoology* **126**, 1–207.

Rivero J. A. (1963). *Eleutherodactylus hedricki,* a new species of frog from Puerto Rico (Salientia, Leptodactylidae). *Breviora* **185**, 1–7.

Rivero J. A. (1968a). A new species of *Eleutherodactylus* (Amphibia, Salientia) from the Guyana Region, Edo. Bolivar, Venezuela. *Breviora*, 1–11.

Rivero, J. A. (1968b) Los centrolénidos de Venezuela. *Memoria de la Sociedad de Ciencias Naturales La Salle* **28**, 301–334.

Rivero J. A. & Castaño, C. J. (1990). A new and peculiar species of *Rhamphophryne* (Amphibia: Bufonidae) from Antioquia, Colombia. *Journal of Herpetology* **24**, 1–5.

Rivero J. A. & Granados Díaz, H. (1993). Nueva especie de *Atelopus* (Amphibia Bufonidae) del Departamento del Cauca, Colombia. *Caribbean Journal of Science* **29**, 12–17.

Rivero J. A. & Serna, M. A. (1989). Una nueva especie de *Atelopus* (Amphibia, Bufonidae) de Colombia. *Caribbean Journal of Science* **25**, 36–40.

Roberto I. J., Brito, L. & Thomé, M. T. C. (2014). A new species of *Rhinella* (Anura: Bufonidae) from northeastern Brazil. *South American Journal of Herpetology* **9**, 190–199.

Roberts J. D. (1997). Call evolution in *Neobatrachus* (Anura: Myobatrachidae): Speculations on tetraploid origins. *Copeia* **1997 (4)**, 791–801.

Rödel M.-O., Schmitz, A., Pauwels, O. S. G. & Böhme, W. (2004). Revision of the genus *Werneria* Poche, 1903, including the descriptions of two new species from Cameroon and Gabon (Amphibia: Anura: Bufonidae). *Zootaxa* **720**, 1–28.

Rödel M.-O. & Ernst, R. (2000). *Bufo taiensis* n. sp., eine neue Kröte aus dem Taï-Nationalpark, Elfenbeinküste. *Herpetofauna* **22**, 9–16.

Rödel M.-O., Kosuch, J., Grafe, T. U., Boistel, R., Assemian, N. E., Kouamé, N. G., Tohé, B., Gourène, G., Perret, J.-L., Henle, K., Tafforeau, P., Pollet, N. & Veith, M. (2009). A new tree-frog genus and species from Ivory Coast, West Africa (Amphibia: Anura: Hyperoliidae). *Zootaxa* **2044**, 23–45.

Rojas R. R., Carvalho, V., Ávila, R., Farias, I., Gordo, M. & Hrbek, T. (2015). Two new species of *Amazophrynella* (Amphibia: Anura: Bufonidae) from Loreto, Peru. *Zootaxa* **3946**, 79–103.

Rojas R. R., Carvalho, V., Gordo, M., Ávila, R., Farias, I. & Hrbek, T. (2014). A new species of *Amazophrynella* (Anura: Bufonidae) from the southwestern part of the Brazilian Guiana Shield. *Zootaxa* **3753**, 79–95.

Rojas R. R., Chaparro, J. C., Carvalho, V. T., Avila, R. W., Farias, I. P., Hrbek, T. & Gordo, M. (2016). Uncovering the diversity in the *Amazophrynella minuta* complex: integrative taxonomy reveals a new species of *Amazophrynella* (Anura, Bufonidae) from southern Peru. *ZooKeys* **563**, 43–71.

Ron S. R., Mueses-Cisneros, J. J., Gutiérrez-Cárdenas, P. D. A., Rojas-Rivera, A., Lynch, R. L., Rocha, C. F. D. & Galarza, G. (2015). Systematics of the endangered toad genus *Andinophryne* (Anura: Bufonidae): phylogenetic position and synonymy under the genus *Rhaebo*. *Zootaxa* **3947**, 347–366.

Rowley J. J. L., Dau, Q. V., Nguyen, T. T., Cao, T. T. & Nguyen, S. V. (2011). A new species of *Gracixalus* (Anura: Rhacophoridae) with a hyperextended vocal repertoire from Vietnam. *Zootaxa* **3125**, 22–38.

Rueda-Almonacid J. V. & Hoyos, J. M. (1992 "1991"). *Atelopus muisca*, nueva especie de anfibio (Anura: Bufonidae) para el Parque Nacional Natural Chingaza, Colombia. *Trianea* **4**, 471–480.

Rueda-Almonacid J. V. (1994) Estudio anatómico y relaciones sistemáticas de *Centrolene geckoideum* (Salientia: Anura: Centrolenidae). *Trianea* **5**, 133–87.

Ruibal R. (1959). *Bufo gundlachi*, a new species of cuban toad. *Breviora* **105**, 1–14.

Ruiz-Carranza P. M. & Hernandez-Camacho, J. I. (1976). *Osornophryne*, género nuevo de anfibios bufónidos de Colombia y Ecuador. *Caldasia* **11**, 93–148.

Ruiz-Carranza P. M. & Lynch J. D. (1991) Ranas Centrolenidae de Colombia IV. Nuevas especies de *Cochranella* del grupo ocellata de la Cordillera Oriental. *Lozania (Acta Zoologica Colombiana)* **60**, 1–16.

Ruiz-Carranza P. M. & Lynch J. D. (1995) Ranas Centrolenidae de Colombia VII. Redescripción de *Centrolene andinum* (Rivero 1968). *Lozania* **64**, 1–12.

Ruiz-Carranza P. M. & Lynch J. D. (1996) Ranas Centrolenidae de Colombia IX. Dos nuevas especies del suroeste de Colombia. *Lozania* **68**, 1–11.

Ruiz-Carranza P. M. & Lynch J. D. (1998) Ranas Centrolenidae de Colombia XI. Nuevas especies de ranas cristal del genero *Hyalinobatrachium*. *Revista de la Academia Colombiana de Ciencias Exactas, Físicas y Naturales* **22**, 571–86.

Ruíz-Carranza P. M. & Osorno-Muñoz, M. (1994). Tres nuevas especies de *Atelopus* AMC Dumeril & Bibron 1841 (Amphibia: Bufonidae) de la Cordillera Central de Colombia. *Revista de la Academia Colombiana de Ciencias* **19**, 165–179.

Ruíz-Carranza P. M., Ardila-Robayo, M. C. & Hernández-Camacho, J. I. (1994). Tres nuevas especies de *Atelopus* AMC Duméril, Bibron 1841 (Amphibia: Bufonidae) de la Sierra Nevada de Santa Marta. *Revista de la Academia Colombiana de Ciencias* **19**, 153–163.

Ruthven A. G. (1916). Description of a new species of *Atelopus* from the Santa Marta mountains, Colombia. *Occasional Papers of the Museum of Zoology, University of Michigan* **28**, 1–3.

Ryuzaki M., Hasegawa, Y. & Kuramoto, M. (2014) A new brown frog of the genus *Rana* from Japan (Anura: Ranidae) revealed by cytological and bioacoustic studies. *Alytes* **30**, 49–58.

Sanabria E., Quiroga, L., Arias, F. & Cortez, R. (2010). A new species of *Rhinella* (Anura: Bufonidae) from Ischigualasto Provincial Park, San Juan, Argentina. *Zootaxa* **2396**, 50–60.

Sanchiz B., Tejedo, M. & Sánchez-Herráiz, M. J. (2002). Diferenciación osteológica entre los *Pelodytes* ibéricos (Anura, Pelodytidae). *Graellsia* **58**, 35–68.

Sandberger L., Hillers, A., Doumbia, J., Loua, N.-S., Brede, C. & Rödel, M.-O. (2010). Rediscovery of the Liberian Nimba toad, *Nimbaphrynoides liberiensis* (Xavier, 1978) (Amphibia: Anura: Bufonidae), and reassessment of its taxonomic status. *Zootaxa* **2355**.

Sanders O. (1953). A new species of toad, with discussion of morphology of the bufonid skull. *Herpetologica* **9**, 25–47.

Santos S., Ibáñez, R. & Ron, S. (2015). Systematics of the *Rhinella margaritifera* complex (Anura, Bufonidae) from western Ecuador and Panama with insights in the biogeography of *Rhinella alata*. *ZooKeys* **501**, 109–145.

Santos-Barrera G. & Villela, O. F. (2011). A new species of toad of the genus *Incilius* from the Sierra Madre Occidental of Chihuahua, Mexico (Anura: Bufonidae). *Journal of Herpetology* **45**, 211–215.

Santos-Barrera G. (2014). Geographic variation in *Incilius occidentalis* (Anura: Bufonidae), an endemic toad from Mexico, with a redescription of the species and delimitation of the type locality. *Revista Mexicana de Biodiversidad* **85**, 414–428.

Savage J. M. (1969). Clarification of the status of the toad, *Bufo veraguensis* O. Schmidt, 1857. *Copeia* **1969**, 178–179.

Savage J. M. (1972). The systematic status of *Bufo simus* O. Schmidt with description of a new toad from western Panama. *Journal of Herpetology* **6**, 25–33.

Savage J. M. (1975). Systematics and distribution of the Mexican and Central American stream frogs related to *Eleutherodactylus rugulosus*. *Copeia* **1975**, 254–306.

Savage J. M. (1981). The systematic status of Central American frogs confused with *Eleutherodactylus cruentus*. *Proceedings of the Biological Society of Washington* **94**, 413–420.

Savage J. M. (1987). Systematics and distribution of the Mexican and Central American rainfrogs of the *Eleutherodactylus gollmeri* group (Amphibia: Leptodactylidae). *Fieldiana. Zoology* **33**, 1–57.

Savage J. M. (2002). *The Amphibians and Reptiles of Costa Rica*. University of Chicago Press, Chicago.

Savage J. M. & Bolaños, F. (2009). An enigmatic frog of the genus *Atelopus* (Family Bufonidae) from Parque Nacional Chirripó, Cordillera de Talamanca, Costa Rica. *Revista de Biologia Tropical* **57**, 381–386.

Savage J. M. & DeWeese, J. E. (1981 "1980"). The status of the Central American leptodactylid frogs *Eleutherodactylus melanostictus* (Cope) and *Eleutherodactylus platyrhynchus* (Günther). *Proceedings of the Biological Society of Washington* **93**, 928–942.

Savage J. M. & Heyer, W. R. (1967). Variation and distribution in the tree–frog genus *Phyllomedusa* in Costa Rica, central America. *Beitr. neotrop. Fauna* **5**, 111–131.

Savage J. M. & Kluge, A. G. (1961). Rediscovery of the strange Costa Rica toad, *Crepidius epioticus* Cope. *Revista de Biologia Tropical* **9**, 39–51.

Savage J. M. & Myers, C. W. (2002). Frogs of the *Eleutherodactylus biporcatus* group (Leptodactylidae) of central america and northern south america, including rediscovered, resurrected, and new taxa. *American Museum Novitates* **3357**, 1–48.

Savage J. M., Hollingsworth, B. D., Lips, K. R. & Jaslow, A. P. (2004). A new species of rainfrog (genus *Eleutherodactylus*) from the Serranía de Tabasará, west-central Panama and reanalysis of the *fitzingeri* group. *Herpetologica* **60**, 519–529.

Savage J. M., Ugarte, C. A. & Donnelly, M. A. (2013). A new species of earless toad (Bufonidae: *Incilius*) from western Panama. *Copeia* **2013**, 8-12.

Scherz M. D., Ruthensteiner, B., Vences, M. & Glaw, F. (2014). A new microhylid frog, genus *Rhombophryne*, from northeastern Madagascar, and a re-description of *R. serratopalpebrosa* using micro-computed tomography. *Zootaxa* **3860**, 547–560.

Schiotz A. (1975). *The treefrogs of eastern Africa. Steenstrupia*. 232 pp.

Schleich H. H., Kästle, W. & Kabisch, K. (1996). *Amphibians and reptiles of North Africa*. Koeltz Scientific Publisher, Koenigstein, Germany.

Schmidt K. P. (1920). Contributions to the herpetology of Porto Rico. *Annals of the New York Academy of Sciences* **28**, 167–200.

Schmidt K. P. (1927). A new treefrog from Porto Rico. *American Museum Novitates*, 1–3.

Schmidt K. P. & Inger, R. F. (1959). Exploration du Parc National de l'Upemba: Mission G.F. de Witte: Amphibians. *Institut des Parcs Nationaux du Congo Belge* **56**, 1–264, 9 plates, 1 foldout map.

Schneider H., Sofianidou, T. S., & Kyriakopoulou-Sklavounou, P. (1984). *Bioacoustic and morphometric studies in water frogs (genus Rana) of Lake Ioannina in Greece, and description of a new species (Anura, Amphibia). Zeitschrift für Zoologische Systematik und Evolutionsforschung* **22**, 349–366.

Schoonees D. A. (1930). Die skedelmorphologie van *Bufo angusticeps* (Smith). *South African Journal of Science* **27**, 456–469.

Schwartz A. (1957). A new species of *Eleutherodactylus* (Amphibia: Leptodactylidae) from Cuba. *Proceedings of the Biological Society of Washington* **70**, 209–212.

Schwartz A. (1958a). Another new large *Eleuthrodactylus* (Amphibia: Leptodactylidae) from western Cuba. *Proceedings of the Biological Society of Washington* **71**, 37–42.

Schwartz A. (1958b). Four new frogs of the genus *Eleutherodactylus* (Leptodactylidae) from Cuba. *American Museum Novitates* **1873**, 1–20.

Schwartz A. (1959). A new species of toad, *Bufo cataulaciceps*, from the Isla de Pinos and western Cuba. *Proceedings of the Biological Society of Washington* **72**, 109–120.

Schwartz A. (1960). The large toads of Cuba. *Proceedings of the Biological Society of Washington* **73**, 45–56.

Schwartz A. (1964). Three new species of frogs (Leptodactylidae, *Eleuthrodactylus*) from Hispaniola. *Breviora*, 1–15.

Schwartz A. (1965 "1964"). Two new species of *Eleutherodactylus* from the eastern Cordillera Central of the "República Dominica". *Caribbean Journal of Science* **4**, 473–484.

Schwartz A. (1965). Variation and natural history of *Eleutherodactylus ruthae* on Hispaniola. *Bulletin of the Museum of Comparative Zoology* **132**, 479–508.

Schwartz A. (1966). The relationships of four small Hispaniolan *Eleutherodactylus* (Leptodactylidae). *Bulletin of the Museum of Comparative Zoology* **133**, 369–399.

Schwartz A. (1972). The native toads (Anura, Bufonidae) of Hispaniola. *Journal of Herpetology* **6**, 217–231.

Schwartz A. (1973). Six new species of *Eleutherodactylus* (Anura, Leptodactylidae) from Hispaniola. *Journal of Herpetology* **7**, 249–273.

Schwartz A. (1976a). Two new species of Hispaniolan *Eleutherodactylus* (Leptodactylidae). *Herpetologica* **32**, 163–171.

Schwartz A. (1976b). Variation and relationships of some Hispaniolan frogs (Leptodactylidae, *Eleutherodactylus*) of the *ricordi* group. *Bulletin of the Florida Museum of Natural History. Biological Sciences* **21**, 1–46.

Schwartz A. (1979). A new species of *Eleutherodactylus* (Amphibia, Anura, Leptodactylidae) from northwestern Haiti, Hispaniola. *Journal of Herpetology* **13**, 199–202.

Scott E. (2005). A phylogeny of ranid frogs (Anura: Ranoidea: Ranidae), based on a simultaneous analysis of morphological and molecular data. *Cladistics* **21**, 507–574.

Sedra S. N. (1959). The ontogenesis of the sound conducting apparatus of the Egyptian toad, *Bufo regularis* Reuss, with a review of this apparatus in Salientia. *Journal of morphology* **104**, 359–375.

Senevirathne G., Thomas A., Kerney R., Hanken J., Biju S.D., Meegaskumbura M. (2016). From clinging to digging: the postembryonic skeletal ontogeny of the indian purple frog, *Nasikabatrachus sahyadrensis* (Anura: Nasikabatrachidae). *PloS One* 11(3), e0151114.

Señaris C., Lampo, M., Rojas-Runjaic, F. J.M. & Barrio-Amorós, C. L. (2014). *Guía ilustrada de los anfibios del Parque Nacional Canaima, Venezuela*.

Señaris J. C. (1995 "1993"). Una nueva especie de *Oreophrynella* (Anura; Bufonidae) de la cima del Auyan-tepui, Estado Bolivar, Venezuela. *Memoria Sociedad de Ciencias Naturales La Salle* **53**, 177–183.

Señaris J. C., Ayarzagüena, J. & Gorzula, S. (1994). Los sapos de la familia Bufonidae (Amphibia: Anura) de las tierras altas de la Guayana Venezolana: Descripción de un nuevo género y tres especies. *Publicaciones de la Asociacion de Amigos de Doñana* **3**, 1–37.

Señaris J. C., do Nascimiento, C. & Villarreal, O. (2005). A new species of the genus *Oreophrynella* (Anura; Bufonidae) from the Guiana Highlands. *Papeis Avulsos de Zoologia* **45**, 61–67.

Seshadri K. S., Gururaja, K. V. & Aravind, N. A. (2012). A new species of *Raorchestes* (Amphibia: Anura: Rhacophoridae) from mid-elevation evergreen forests of the southern Western Ghats, India. *Zootaxa* **3410**, 19–34.

Shah K. B. & Gruber, U. (1994). *Bufo microtympanum* Boulenger, 1882, a bufonid toad new for Nepal. *Spixiana* **17**, 57–61.

Sheil C. A. & Alamillo, H. (2005). Osteology and skeletal development of *Phyllomedusa vaillanti* (Anura: Hylidae: Phyllomedusinae) and a comparison of this arboreal species with a terrestrial member of the genus. *Journal of morphology* **265**, 343–368.

Shen Y.-H., Jiang, J.-P. & Yang, D.-D. (2007). *A new species of the genus Rana—Rana hanluica sp. nov. from Hunan Province, China (Anura: Ranidae). Acta Zoologica Sinica* **53**, 481–488.

Smirnov S. V. (1991). The anuran middle ear: developmental heterochronies and adult morphology diversification. *Belgian Journal of Zoology* **121**, 99–110.

Smith E. N. & Noonan, B. P. (2001). A new species of *Osteocephalus* (Anura: Hylidae) from Guyana. *Revista de Biología Tropical. San José* **49**, 347–357.

Smith H. M. (1939). Mexican herpetological novelties. *Proceedings of the Biological Society of Washington* **52**, 187–196.

Smith H. M., Chiszar, D., Collins, J. T. & van Breukelen, F. (1998). The taxonomic status of the Wyoming toad, *Bufo baxteri* Porter. *Contemporary Herpetology* **22**, [1–5].

Smith M. A. (1921) New or Little-known Reptiles and Batrachians from Southern Annam (Indo-China). *Proceedings of the Zoological Society of London* **91**, 423–440.

Spawls S., Howell, K. M. & Drewes, R. C. (2006). *Pocket guide to the Reptiles and Amphibians of East Africa*. A&C Black Publishers Ltd.

Stejneger L. (1901). Diagnoses of eight new batrachians and reptiles from the Riu Kiu Archipelago, Japan. *Proceedings of the Biological Society of Washington* **14**, 189–191.

Stephenson E. M. (1951). The anatomy of the head of the New Zealand frog, *Leiopelma*. *Transactions of the Zoological Society of London* **27**, 255–305.

Stevaux M. N. (2002). A new species of *Bufo* Laurenti (Anura, Bufonidae) from northeastern Brazil. *Revista Brasileira de Zoología* **19**, 235–242.

Stöck M., Gunther, R. & Bohme, W. (2001). Progress towards a taxonomic revision of the Asian *Bufo viridis* group: Current status of nominal taxa and unsolved problems (Amphibia: Anura: Bufonidae). *Zoologische Abhandlungen* **51**, 253–319.

Stöck M., Schmid, M., Steinlein, C. & Grosse, W.-R. (1999). Mosaicism in somatic triploid specimens of the *Bufo viridis* complex in the Karakoram with examination of calls, morphology and taxonomic conclusions. *Italian Journal of Zoology* **66**, 215–232.

Stöck M., Sicilia, A., Belfiore, N. M., Buckley, D., Lo Brutto, S., Lo Valvo, M. & Arculeo, M. (2008). Post-Messinian evolutionary relationships across the Sicilian channel: mitochondrial and nuclear markers link a new green toad from Sicility to African relatives. *BMC Evolutionary Biology* **8**, 1–19.

Streicher J. W., Meik, J. M., Smith, E. N. & Campbell, J. A. (2011). Low levels of genetic diversity among morphologically distinct populations of an enigmatic montane frog from Mexico (*Craugastor uno*: Craugastoridae). *Amphibia-Reptilia* **32**, 125–131.

Stuart B. L., Orlov, N. L. & Chan-ard, T. (2005). A new cascade frog (Amphibia: Ranidae) from Laos and Vietnam. *Raffles Bulletin of Zoology* **53**, 125–131.

Tandy M. & Keith, R. (1972). *Bufo* of Africa. In *Evolution in the genus Bufo* (ed. W. F. Blair), pp. 119–170. University of Texas Press, Austin.

Tandy M., Bogart, J. P., Largen, M. J. & Feener, D. J. (1982). A tetraploid species of *Bufo* (Anura Bufonidae) from Ethiopia. *Monitore Zoologico Italiano Supplemento* **17**, 1–79.

Tandy M., Tandy, J., Keith, R. & MacKay, A. D. (1976). A new species of *Bufo* (Anura: Bufonidae) from Africa's dry savannas. *Pearce-Sellards Series. Texas Memorial Museum. Austin* **24**, 1–20.

Targino M. & Carvalho-e-Silva, S. P. d. (2008). Redescription of *Ischnocnema holti* (Amphibia, Anura). *Revista Brasileira de Zoologia* **25**, 716–723.

Taylor E. H. (1936). Notes on the Herpetological fauna of the Mexican state of Sinaloa. *University of Kansas Science Bulletin* **24**, 505–537.

Taylor E. H. (1937 "1936"). New species of Amphibia from Mexico. *Transactions of the Kansas Academy of Science* **39**, 349–363.

Taylor E. H. (1940a). A new frog from the Tarahumara Mountains of Mexico. *Copeia* **1940**, 250–253.

Taylor E. H. (1940b). A new *Syrrhophus* from Guerrero, Mexico. *Proceedings of the Biological Society of Washington* **53**, 95–98.

Taylor E. H. (1942). New tailless amphibia from Mexico. *University of Kansas Science Bulletin* **28**, 67–89.

Taylor E. H. (1943). Herpetological novelties from Mexico. *University of Kansas Science Bulletin* **29**, 343–361.

Taylor E. H. (1951). The rediscovery of the toad *Bufo simus* Schmidt. *Copeia* **1951**, 134–137, 1 pl.

Taylor E. H. (1962). The Amphibian Fauna of Thailand. *University of Kansas Science Bulletin* **43**, 265–599.

Teixeira M., Recoder, R. S., Amaro, R. C., Damasceno, R. P., Cassimiro, J. & Rodrigues, M. T. (2013). A new *Crossodactylodes* Cochran, 1938 (Anura: Leptodactylidae: Paratelmatobiinae) from the highlands of the Atlantic Forests of southern Bahia, Brazil. *Zootaxa* **3702**, 459–472.

Teynié A., David, P. & Ohler, A. (2010). Note on a collection of amphibians and reptiles from western Sumatra (Indonesia), with the description of a new species of the genus *Bufo*. *Zootaxa* **2416**, 1–43.

Tihen J. A. (1960). Two new genera of African bufonids, with remarks on the phylogeny of related genera. *Copeia* **1960**, 225–233.

Tolley K. A., de Villiers, A. L., Cherry, M. I. & Measey, G. J. (2010). Isolation and high genetic diversity in dwarf mountain toads (*Capensibufo*) from South Africa. *Biological Journal of the Linnean Society* **100**, 822–834.

Trueb L. (1970a). Cranial Osteology of the Hylid Frog, *Smilisca baudini*. *University of Kansas Publications, Museum of Natural History* **18**, 11–35.

Trueb L. (1970b). Evolutionary relationships of casque-headed tree frogs with co-ossified skulls (Family Hylidae). *University of Kansas Publications, Museum of Natural History* **18**, 547–716.

Trueb L. (1970c). The generic status of *Hyla siemersi* Mertens. *Herpetologica* **26**, 254–267.

Trueb L. (1971). Phylogenetic relationships of certain neotropical toads with the description of a new genus (Anura: Bufonidae). *Bulletin of Los Angeles County Museum of Natural History* **216**, 1–40.

Trueb L. (1973). Bones, frogs, and evolution. In *Evolutionary biology of the anurans: Contemporary research on major problems* (ed. J. L. Vial), pp. 65–132. University of Missouri Press, Columbia.

Trueb L. (1974). Systematic relationships of neotropical horned frogs, genus *Hemiphractus* (Anura: Hylidae). *Occasional Papers of the Museum of Natural History, University of Kansas* **29**, 1-60.

Trueb L. (1979). Leptodactylid frogs of the genus *Telmatobius* in Ecuador with the description of a new species. *Copeia* **1979**, 714-733.

Trueb L. & Cannatella, D. C. (1982). The cranial osteology and hyolaryngeal apparatus of *Rhinophrynus dorsalis* (Anura: Rhinophrynidae) with comparisons to recent pipid frogs. *Journal of morphology* **171**, 11–40.

Trueb L. & Duellman, W. E. (1971). A synopsis of neotropical hylid frogs, genus *Osteocephalus. Occasional Papers of the Museum of Natural History, The University of Kansas* **1**, 1–47.

Trueb L. & Duellman, W. E. (1971). A synopsis of neotropical hylid frogs, genus *Osteocephalus*. *Occasional Papers of the Museum of Natural History, The University of Kansas* **1**, 1–47.

Trueb L. & Tyler, M. J. (1974). Systematics and evolution of the greater Antillean hylid frogs. *Occasional Papers of the Museum of Natural History, University of Kansas* **24**, 1–60.

Trueb L., Diaz, R. & Blackburn, D. C. (2011). Osteology and chondrocranial morphology of *Gastrophryne carolinensis* (Anura: Microhylidae), with a review of the osteological diversity of New World microhylids. *Phyllomedusa* **10**, 99–135.

Trueb L., Pugener, L. A. & Maglia, A. M. (2000). Ontogeny of the bizarre: an osteological description of *Pipa pipa* (Anura: pipidae), with an account of skeletal development in the species. *Journal of Morphology* **243**, 75–104.

Tyler M. J. & Davies, M. (1978). Species-groups within the australopapuan hylid frog genus *Litoria* Tschudi. *Australian Journal of Zoology* **Suppl. 63**, 1–47.

Tyler M. J. & Davies, M. (1979). Redefinition and evolutionary origin of the australopapuan hylid frog genus *Nyctimystes* Setjneger. *Australian Journal of Zoology* **27**, 755–772.

Tyler M. J. (1972). A new genus for the Australian leptodactylid frog *Crinia darlingtoni*. *Zoologische Mededelingen* **47**, 193–201.

Tyler M. J., Davis, M. & Martin, A. A. (1981). Australian frogs of the leptodactylid genus *Uperoleia* Gray. *Australian Journal of Zoology* **29**, 1–64.

Urra F. A. (2013). Síntesis del conocimiento actual sobre los sapos *Rhinella atacamensis*, *R. arunco* y *R. spinulosa*. *La Chiricoca* **3**, 4–15.

Vaira M. & Ferrari, L. (2008). A new species of *Oreobates* (Anura: Strabomantidae) from the Andes of northern Argentina. *Zootaxa* **1908**, 41–40.

Valdés de la Osa A. & Ruiz, F. (1980). Consideraciones sistemáticas sobre *Bufo longinasus* (Anura: Bufonidae) y descripción de una nueva subespecie. *Poeyana* **206**, 1–33.

van Kampen P. N. (1923). *The amphibia of the Indo-Australian archipelago*. Brill, Leiden.

Vaughan A. & Mendelson, J. R. (2007). Taxonomy and ecology of the Central American toads of the genus *Crepidophryne* (Anura: Bufonidae). *Copeia* **2007**, 304–314.

Vaz-Silva W., Maciel, N. M., Bastos, R. P. & Pombal Jr, J. P. (2015). Revealing two new species of the *Rhinella margaritifera* species group (Anura, Bufonidae): an enigmatic taxonomic group of Neotropical toads. *Herpetologica* **71**, 212–222.

Vaz-Silva W., Valdujo, P. H. & Pombal, J. P. (2012). New species of the *Rhinella crucifer* group (Anura, Bufonidae) from the Brazilian Cerrado. *Zootaxa* **3265**, 57–65.

Veith M., Kosuch J., Ohler A. & Dubois A. (2001) Systematics of *Fejervarya limnocharis* (Gravenhorst, 1829) (Amphibia, Anura, Ranidae) and related species. 2. Morphological and molecular variation in frogs from the Greater Sunda Islands (Sumatra, Java, Borneo) with the definition of two species. *Alytes* **19**, 5–28.

Vélez-Rodriguez C. M. (2005). Osteology of *Bufo sternosignatus* Gunther, 1858 (Anura: Bufonidae) with comments on phylogenetic implications. *Journal of Herpetology* **39**, 299–303.

Vélez-R. C. M. & Ruiz-C., P. M. (2002). A new species of *Bufo* (Anura: Bufonidae) from Colombia. *Herpetologica* **58**, 453–462.

Vélez-Rodriguez C. M. & Ruiz-Carranza, P. M. (1997). Una nueva especie de *Atelopus* (Amphibia: Anura: Bufonidae) de la Cordillera Central, Colombia. *Revista de la Academia Colombiana de Ciencias Exactas, Físicas y Naturales* **21**, 555–563.

Vellard J. (1959). Estudios sobre batracios andinos V. El género *Bufo*. *Memorias del Museo de Historia Natural "Javier Prado"* **8**, 1–48.

Veloso A., Díaz, N., Iturra, P. & Penna, M. (1981). Descripción de una nueva especie de telmatobino del género *Alsodes* (Amphibia, Leptodactylidae) de la Cordillera de Nahuelbuta (sur de Chile). *Medio Ambiente* **5**, 72–77.

Veloso A., Sallaberry-Ayerza, M., Navarro, J., Iturra-Constant, P., Valencia, J., Penna, M. & Díaz, N. F. (1982). Contribución sistemática al conocimiento de la herpetofauna del extremo norte de Chile. In *El Ambiente Natural y Los Poblaciones Humanas de los Andes del Norte Grande de Chile (Arica, Lat 18° 28′ S). Volume 1. La Vegetación y los Vertebrados Inferiores de los Pisos Altitudinalis entre Arica y el Lago Chungara* (ed. A. Veloso A. & E. Bustos), pp. 135–265. Man and Biosphere (MAB-6, El Hombre y los Ecosistemas de Montaña), UNESCO, Montevideo, Uruguay.

Vences M. & Glaw, F. (2003). New microhylid frog (*Plethodontohyla*) with a supraocular crest from Madagascar. *Copeia* **2003**, 789–793.

Vences M. & Glaw, F. (2005). A new cryptic frog of the genus *Boophis* from the northwestern rainforests of Madagascar. *African Journal of Herpetology* **54**, 77–84.

Vences M., Glaw, F. Jesu, R. & Schimmenti, G. (2000). A new species of *Heterixalus* (Amphibia: Hyperoliidae) from western Madagascar. *African Zoology* **35**, 269–276.

Vences M., Glaw, F., Andreone, F., Jesu, R. & Schimmenti, G. (2002). Systematic revision of the enigmatic Malagasy broad-headed frogs (*Laurentomantis* Dubois, 1980), and their phylogenetic position within the endemic mantellid radiation of Madagascar. *Contributions to Zoology* **70**, 191–212.

Venegas P. J. & Barrio, J. (2005). A new species of harlequin frog (Anura: Bufonidae: *Atelopus*) from the northern Cordillera Central, Peru. *Revista Española de Herpetología* **29**, 103–112.

Venegas P. J., Catenazzi, A., Siu-Ting, K. & Carrillo, J. (2008). Two new harlequin frogs (Anura: *Atelopus*) from the Andes of northern Peru. *Salamandra* **44**, 163–176.

Verdade V. K. (2005). Relações filogenéticas entre as espécies dos gêneros *Cycloramphus* Tschudi 1838 e *Zachaenus* Cope 1866 (Anura, Leptodactylidae), Universidade de São Paulo.

Vigle G. O. & Goberdhan-Vigle, D. C. I. (1990). A new species of small colorful *Hyla* from the lowland rainforest of amazonian Ecuador. *Herpetologica* **46**, 467–473.

Vorobyeva E. & Smirnov, S. (1987). Characteristic features in the formation of anuran sound-conducting systems. *Journal of morphology* **192**, 1–11.

Vrcibradic D., Almeida-Gomes, A., van Sluys, M. & Rocha, C. F. D. (2008). Amphibia, Anura, *Hylodes charadranaetes, Ischnocnema octavioi,* and *Euparkerella cochranae*: Distribution extension. *Check List* **4**, 103–106.

Wang Y.-Y., Zhang, T.-D., Zhao, J., Sung, Y.-H., Yang, J.-H., Pang, H. & Zhang, Z. (2012). Description of a new species of the genus *Xenophrys* Günther, 1864 (Amphibia: Anura: Megophryidae) from Mount Jinggang, China, based on molecular and morphological data. *Zootaxa* **3546**, 53–67.

Wassersug R.J. (1976) A procedure for differential staining of cartilage and bone in whole formalin-fixed vertebrates. Stain Technology 51(2), 131–134.

Wei G., Wang, B., Xu, N., Li, Z. & Jiang, J. (2009). Morphological evolution from aquatic to terrestrial in the genus *Oreolalax* (Amphibia, Anura, Megophryidae). *Progress in Natural Science* **19**, 1403–1408.

Wever E. G. (1985). *The amphibian ear*. Princeton University Press, Princeton.

Wickramasinghe L. J. M., Vidanapathirana, D. R., Rajeev, M. D. G., Ariyarathne, S. C., Chanaka, A. W. A., Priyantha, L. L. D., Bandara, I. N. & Wickramasinghe, N. (2013). Eight new species of *Pseudophilautus* (Amphibia: Anura: Rhacophoridae) from Sripada World Heritage Site (Peak Wilderness), a local amphibian hotspot in Sri Lanka. *Journal of Threatened Taxa* **5**, 3789–3920.

Wiens J. J. (1989). Ontogeny of the skeleton of *Spea bombifrons* (Anura: Pelobatidae). *Journal of morphology* **202**, 29–51.

Wiens J. J. (1993). Systematics of the leptodactylid frog genus *Telmatobius* in the Andes of northern Peru. *Occasional Papers of the Museum of Natural History, University of Kansas* **161**, 1–76.

Wild E. R. (1999). Description of the chondrocranium and osteogenesis of the Chacoan burrowing frog, *Chacophrys pierotti* (Anura: Leptodactylidae). *Journal of morphology* **242**, 229–246.

Wilkinson J. A., Sellas, A. B. & Vindum, J. V. (2012). A new species of *Ansonia* (Anura: Bufonidae) from northern Tanintharyi Division, Myanmar. *Zootaxa* **3163**, 54–68.

Wogan G. O. U., Win, H., Thin, T., Lwin, K. S., Shein, A. K., Kyi, S. W. & Tun, H. (2003). A new species of *Bufo* (Anura: Bufonidae) from Myanmar (Burma), and redescription of the little-known species *Bufo stuarti* Smith 1929. *Proceedings of the California Academy of Science* **54**, 141–153.

Wood P. L., Grismer, L. L., Ahmad, N. & Senawi, J. (2008). Two new species of torrent-dwelling toads *Ansonia* Stoliczka, 1870 (Anura: Bufonidae) from Peninsular Malaysia. *Herpetologica* **64**, 321–340.

Wright A. H. & Wright, A. A. (1949). *Handbook of frogs and toads of the United States and Canada*. Comstock, Ithaca, New York.

Wu G.-F., Zhao, E.-M., Inger, R. F. & Shaffer, H. B. (1993). A new frog of the genus *Oreolalax* (Pelobatidae) from Sichuan, China. *Journal of Herpetology* **27**, 410-413.

Wu S.-P., Huang, C.-C., Tsai, C.-L., Lin, T.-E., Jhang, J.-J. & Wu, S.-H. (2016) Systematic revision of the Taiwanese genus *Kurixalus* members with a description of two new endemic species (Anura, Rhacophoridae). *ZooKeys* **557**, 121-153.

Yamazaki Y., Kouketsu, S., Fukuda, T., Araki, Y. & Nambu, H. (2008). Natural hybridization and directional introgression of two species of Japanese toads *Bufo japonicus formosus* and *Bufo torrenticola* (Anura: Bufonidae) resulting from changes in their spawning habitat. *Journal of Herpetology* **42**, 427–436.

Yánez-Muñoz M. H., Altamirano-Benavides, M., Cisneros-Heredia, D. F. & Gluesenkamp, A. G. (2011). Nueva especie de sapo andino del género *Osornophryne* (Amphibia: Bufonidae) del norte de Ecuador, con notas sobre la diversidad del género en Colombia. *Avances en Ciencias e Ingenierías* **2**, 46–53.

Yang D.-T. (1991). Phylogenetic systematics of the *Amolops* group of ranid frogs of southeastern Asia and the Greater Sunda Islands. *Fieldiana Zoology* **63**, 1–42.

Yang D.-T., Liu, W.-Z. & Rao, D.-Q. (1996). A new toad genus of Bufonidae *Torrentophryne* from the transhimalaya mountain of Yunnan of China with its biology. *Zoological Research/Dngwùxué yánji. Kunming* **17**, 353–359.

Zhao E.-M. & Adler, K. A. (1993). *Herpetology of China*. Society for the Study of Amphibians and Reptiles, Oxford, Ohio: Contributions to Herpetology 10.

Ziegler T. & Köhler J. (2001) *Rhacophorus orlovi* sp. n., ein neuer Ruderfrosch aus Vietnam (Amphibia: Anura: Rhacophoridae). *Sauria* **23**, 37–46.

Zug G. R. (2013). *Reptiles and Amphibians of the Pacific Islands*. University of California Press, Berkeley.

Zweifel R. G. (1956a). Results of the Archbold Expeditions. No. 72. Microhylid frogs from New Guinea, with descriptions of new species. *American Museum Novitates* **1766**, 1–49.

Zweifel R. G. (1956b). Survey of the frogs of the *augusti* group, genus *Eleutherodactylus*. *American Museum Novitates* **1813**, 1–35.

Zweifel R. G. (1960). A new microhylid frog from the Adelbert Mountains of New Guinea. *American Museum Novitates* **2012**, 1–7.

Zweifel R. G. (1972). Results of the Archbold expeditions. No. 97. A revision of the frogs of the subfamily Asterophryinae family Microhylidae. *Bulletin of the American Museum of Natural History* **148**, 411–546.

Zweifel R. G. (1985). Australian frogs of the family Microhylidae. *Bulletin of the American Museum of Natural History* **182**, 265–388.

Zweifel R. G. (1986). A new genus and species of microhylid frog from the Cerro de la Neblina region of Venezuela and a discussion of relationships among new world microhylid genera. *American Museum Novitates* **2863**, 1–24.

Zweifel R. G. (2000). Partition of the australopapuan microhylid frog genus *Sphenophryne* with descriptions of new species. *Bulletin of the American Museum of Natural History* **253**, 1–130.

Section S2. Ancestral character state reconstructions and detailed description of the occurrence of the columella in non-Bufonidae anuran families

S2.1—Parsimony ancestral state reconstructions of columella condition for outgroups following the phylogenetic hypothesis of Pyron (2014).

Note that the plesiomorphic condition in Anura is presence of columella (see Discussion section for details).

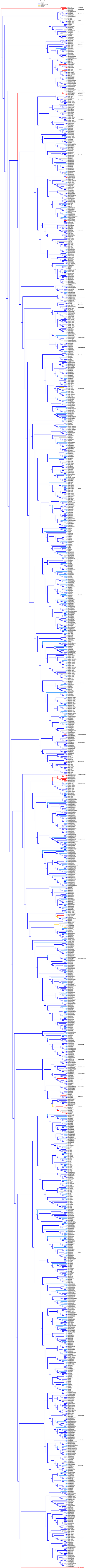

Columella
Absent
Present
Presence inferred
Ambiguous
No data
Ascaphidae
Leiopelmatidae
Bombinatoridae
Alytidae
Rhinophrynidae
Pipidae
Scaphiopodidae
Pelodytidae
Pelobatidae
Megophryidae
Heleophrynidae
Nasikabatrachidae
Sooglossidae
Hemisotidae
Brevicipitidae

S2.2—Additional information on clades or species of Anura in which tympanic middle ear structures were completely lost (as evidenced by the lack of columella).

Clades in which the lack of columella is a putative synapomorphy. Numbers given in brackets are scored species/total species in the genus

Ascaphus [1/2] (Ascaphidae) + *Leiopelma* [2/4] (Leiopelmatidae): As was noted in the Discussion section, the lack of tympanic middle ear structures is a synapomorphy of the most basal extant anuran clade (see Section S2.1).

Atelognathus [6/7] + *Chaltenobatrachus* [1/1] (Batrachylidae): The only two species included by Pyron (2014) lack a columella, as well as *A. nitoi, A. praebasalticus, A. reverberii,* and *A. solitarius* (see Section S1). *Chaltenobatrachus grandisonae* was not included by Pyron (2014) but Basso *et al.* (2011) recovered *Atelognathus* and *Chaltenobatrachus* as highly supported sister genera.

Brachycephalus [8/29] (Brachycephalidae): The eight scored species that were included by Pyron (2014) lack a columella. The remaining four sampled species have unknown character states.

Hoplophryne [2/2] (Microhylidae): Both species of this genus lack a columella.

Pseudophryne [10/14] (Myobatrachidae): The only two species included by Pyron (2014) lack a columella. In addition eight species not included in that hypothesis lack this structure (see Section S1), and the condition is unknown in the remaining four species.

Telmatobufo [3/4] (Calyptocephalellidae): The only two species included by Pyron (2014) lack a columella, as well as *T. australis* (see Section S1). The character state in the remaining species (*T. ignotus*) is unknown.

Species in which the lack of a columella is autapomorphic according to the phylogenetic hypothesis of Pyron (2014)

Balebreviceps hilmani (Brevicipitidae): Monotypic genus.

Melanobatrachus indicus (Microhylidae): Monotypic genus.

Rhinophrynus dorsalis (Rhinophrynidae): Monotypic family.

Pristimantis duellmani, P. simonsii, and *P. surdus* (Craugastoridae): The lack of a columella in these species represents three independent autapomorphies within *Pristimantis*.

Species where a more exhaustive phylogenetic or/and osteological data are necessary to better understand the evolution of tympanic middle ear

Alsodes (Alsodidae): The plesiomorphic condition in this genus is recovered as ambiguous. The revision of the sequences employed in the phylogenetic analysis of Pyron (2014) revealed some

chimeric terminals (B. L. Blotto, obs. pers.). Despite having considered more terminals for *Alsodes*, the phylogenetic hypothesis of Blotto *et al*. (2013) differs in the position of only a few terminals, and optimization of the columella results in the same ambiguities as Pyron's hypothesis. Nevertheless, a rather complex pattern of evolution for the occurrence of columella is evident (see Section S2.1). Thus, the coding of columella condition in additional species of *Alsodes* may help to solve the ambiguities.

Atopophrynus syntomopus (Brachycephaloidea): This species lacks a columella. Unfortunately, its phylogenetic relationships within Brachycephaloidea are uncertain (see Padial *et al*., 2014), so it is not possible to interpret the significance of this condition in a phylogenetic context.

Bombina (Bombinatoridae): As was noted by Ramaswami (1942) and Wever (1985) the pattern of morphological and developmental variation of the columella in this genus is complex. In some species this element was reported to be a cartilaginous structure connected to the hyoid laterally (see Wever, 1985) or as a small independent cartilage that was interpreted as a remnant of the columella (Stadtmüller, 1931). Based on the presence or absence of these cartilages, we score the columella as present in *B. maxima*, *B. orientalis*, *B. pachypus,* and *B. variegata* and absent in *B. bombina*.

Crinia riparia (Myobatrachidae): This species is deeply nested in *Crinia* (see Appendix S2.1). The inclusion of the remaining species not sampled by Pyron (2014) in a phylogenetic analysis, as well as additional description of the columella in this genus, will help to understand if this condition represents an apomorphy of this species or of a subclade of *Crinia*.

Euparkerella + *Holoaden* and *Bryophryne* (Craugastoridae): all five species of *Euparkerella*, the two scored species of *Holoaden*, and the only species of *Bryophryne* included by Pyron (2014) lack a columella. The phylogenetic relationships among these genera result in an ambiguity for columella condition within Craugastoridae (see Section S2.1). As *Bryophryne* includes eight additional species, some of which have a columella (see Section S1), a more inclusive phylogeny of this clade, and new morphological data will help to understand the pattern of tympanic middle ear evolution in Craugastoridae.

Hemisus (Hemisotidae): The only included species of this genus lacks a columella. The study of the remaining species will help to understand if this condition represents an autapomorphy of this species or a synapomorphy of *Hemisus* (or a subclade).

Microhyla (Microhylidae): Parker (1934) indicates that all the species of *Microhyla* he studied have a complete middle ear except for lacking an external tympanic membrane. However, Vorobyeva and Smirnov (1987), who specifically studied this character system, found *Microhyla butleri* and *M. heymonsi* to lack a columella. Subsequently, Smirnov (1991) states that *M. ornata* possesses a tympanic middle ear whereas *M. heymonsi* lacks it. He also described *M. pulchra* as

having a poorly differentiated plectral apparatus in newly metamorphosed individuals. So, it is clear that the columella is both present and absent in *Microhyla*, and more studies are necessary to understand the taxonomic distribution of the different elements of the tympanic middle ear in this genus.

Nanorana (Dicroglossidae): There is an ambiguity in a subclade of this genus (see Section S2.1) for which it is not possible to define the number of transformations in the columella in this genus.

Oreolalax (Megophryidae): Only one species of this genus was included by Pyron (2014), and it lacks a columella; however, the occurrence of a columella varies among species of this genus, so the inclusion of at least some of the remaining 17 species in phylogenetic analyses is necessary to determine if this condition represents an apomorphy of this species or of a subclade of *Oreolalax*.

Phrynopus juninensis and related species (Craugastoridae): The closely related species of *P. juninensis* (e.g. *P. bracki*, *P. tautzorum*) lack a tympanic membrane and annulus, but the condition for the columella is unknown (see Section S1). Additional studies are necessary to understand the pattern of evolution of the tympanic middle ear.

Scutiger (Megophryidae): Two independent losses occurred within this genus (see Section S2.1), although only six of the 20 species were included by Pyron (2014) and the columella condition is known for only five of those species (see Section S1).

Telmatobius (Telmatobiidae): Two independent secondary losses and two cases of intraspecific polymorphism occur in this genus (see Section S2.1). Only 17 of the 63 species of the genus were included by Pyron (2014), and columella condition was scored for 15 of those (see Section S1). Although the columella was described as absent in *T. yuracare* (de la Riva, 1994), it was scored as present because the examination of the same specimens used for the original osteological description (MNCN 16645-46) reveal the presence of an extremely small columella. In *Telmatobius*, the columella shows marked differences in shape and size, even intraspecifically and within the same individual (Wiens, 1993).

Additional species with absence of tympanic middle ear not included in Pyron (2014)

Arenophryne rotunda (Myobatrachidae): This species lacks a columella and was recovered as sister of taxon of *Myobatrachus* by Read *et al*. (2001). In the context of this phylogenetic relationship, this condition represents an independent loss in Anura. The condition in the other species of this genus is unknown.

Crossodactylodes and *Paratelmatobius* (Leptodactylidae): *Crossodactylodes septentrionalis* and two additional unnamed species were included in the phylogenetic analysis of Fouquet *et al.* (2013), and the absence of columella was proposed as a synapomorphy of this genus. Four analyzed species

lack a columella (see Section S1), whereas this condition is unknown in the remaining species. *Paratelmatobius lutzi* is the only known species of *Paratelmatobius* lacking a columella, whereas it is present in *P. cardosoi*, *P. mantiqueira*, *P. poecilogaster*, and *P. yepiranga*, and the condition is unknown in *P. gaigeae* (see Section S1). If *P. lutzi* is recovered nested in *Paratelmatobius* the lack of columella in this species represent another independent loss in Anura. Alternatively, if this species is the sister taxon of all other species of the genus, this can result in an ambiguity for the columella condition within Paratelmatobiinae, according to the relationships proposed by Fouquet *et al*. (2013).

S2.3—References

Basso N. G., Ubeda, C. A., Bunge, M. M. & Martinazzo, L. B. (2011). A new genus of neobatrachian frog from southern Patagonian forests, Argentina and Chile. *Zootaxa* **3002,** 31–44.

Biju S. D. & Bossuyt, F. (2003). New frog family from India reveals an ancient biogeographical link with the Seychelles. *Nature* **425,** 711–714.

Blotto B. L., Nunez, J. J., Basso, N. G., Ubeda, C. A., Wheeler, W. C. & Faivovich, J. (2013). Phylogenetic relationships of a Patagonian frog radiation, the *Alsodes* + *Eupsophus* clade (Anura: Alsodidae), with comments on the supposed paraphyly of *Eupsophus*. *Cladistics* **29,** 113–131.

de la Riva I. (1994). A new aquatic frog of the genus *Telmatobius* (Anura: Leptodactylidae) from Bolivian cloud forests. *Herpetologica* **50,** 38–45.

Dutta S. K., Vasudevan, K., Chaitra, M., Shanker, K. & Aggarwal, R. K. (2004). Jurassic frogs and the evolution of amphibian endemism in the Western Ghats. *Current Science* **86,** 211–216

Fouquet A., Blotto, B. L., Maronna, M. M., Verdade, V. K., Juncá, F. A., de Sá, R. & Rodrigues, M. T. (2013). Unexpected phylogenetic positions of the genera *Rupirana* and *Crossodactylodes* reveal insights into the biogeography and reproductive evolution of leptodactylid frogs. *Molecular Phylogenetics and Evolution* **67,** 445–457.

Padial J. M., Grant, T. & Frost, D. R. (2014). Molecular systematics of terraranas (Anura: Brachycephaloidea) with an assessment of the effects of alignment and optimality criteria. *Zootaxa* **3825,** 1–132.

Parker H. W. (1934). *A monograph of the frogs of the family Microhylidae*. Trustees of the British Museum, London.

Pyron R. A. (2014). Biogeographic analysis reveals ancient continental vicariance and recent oceanic dispersal in amphibians. *Systematic Biology* **63,** 779–797

Ramaswami L. S. (1942). The discoglossid skull. *Proceedings of the Indian Academy of Sciences. Section B* **16,** 10–2

Read K., Keogh, J. S., Scott, I. A. W., Roberts, J. D. & Doughty, P. (2001). Molecular phylogeny of the australian frog genera *Crinia, Geocrinia*, and allied taxa (Anura, Myobatrachidae). *Molecular Phylogenetics and Evolution* **21,** 294–308.

van der Meijden A., Boistel, R., Gerlach, J., Ohler, A., Vences, M. & Meyer, A. (2007). Molecular phylogenetic evidence for paraphyly of the genus *Sooglossus*, with the description of a new genus of Seychellean frogs. *Biological Journal of the Linnean Society* **91,** 347–359.

Vorobyeva E. & Smirnov, S. (1987). Characteristic features in the formation of anuran sound-conducting systems. *Journal of morphology* **192,** 1–11.

Wiens J. J. (1993). Systematics of the leptodactylid frog genus *Telmatobius* in the Andes of northern Peru. *Occasional Papers of the Museum of Natural History, University of Kansas* **161,** 1–76.

Section S3. Results of ancestral reconstructions for Bufonidae

S3.1—Partial phylogenetic tree of Pyron (2014) showing ancestral reconstructions for: (a) tympanic membrane with parsimony, (b) tympanic annulus with parsimony, (c) columella considering equal rates transitions with maximum likelihood, (d) columella considering all rates differ transitions with maximum likelihood, (e) columella considering equal rates transitions with stochastic character mapping, (f) columella considering equal rates transitions + "eared" ancestor with stochastic character mapping, (g) columella considering Dollo's law (no regains) with stochastic character mapping.

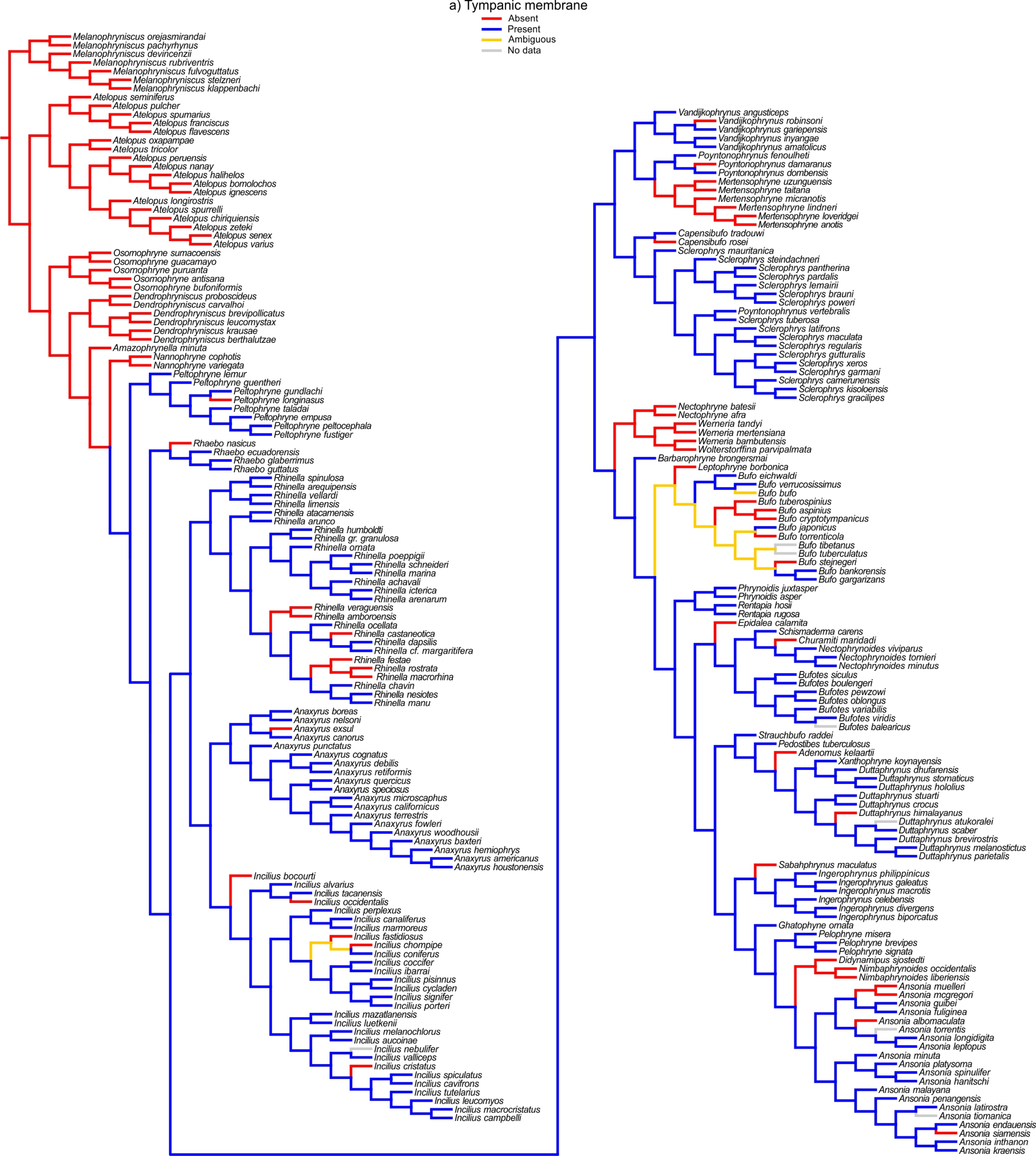
a) Tympanic membrane
Absent
Present
Ambiguous
No data
Melanophryniscus orejasmirandai
Melanophryniscus pachyrhynus
Melanophryniscus devincenzii
Melanophryniscus rubriventris
Melanophryniscus fulvoguttatus
Melanophryniscus stelzneri
Melanophryniscus klappenbachi
Atelopus seminiferus
Atelopus pulcher
Atelopus spumarius
Atelopus franciscus
Atelopus flavescens
Atelopus oxapampae
Atelopus tricolor
Atelopus peruensis
Atelopus nanay
Atelopus halihelos
Atelopus bomolochos
Atelopus ignescens
Atelopus longirostris
Atelopus spurrelli
Atelopus chiriquiensis
Atelopus zeteki
Atelopus senex
Atelopus varius
Osornophryne sumacoensis
Osornophryne guacamayo
Osornophryne puruanta
Osornophryne antisana
Osornophryne bufoniformis
Dendrophryniscus proboscideus
Dendrophryniscus carvalhoi
Dendrophryniscus brevipollicatus
Dendrophryniscus leucomystax
Dendrophryniscus krausae
Dendrophryniscus berthalutzae
Amazophrynella minuta
Nannophryne cophotis
Nannophryne variegata
Peltophryne lemur
Peltophryne guentheri
Peltophryne gundlachi
Peltophryne longinasus
Peltophryne taladai
Peltophryne empusa
Peltophryne peltocephala
Peltophryne fustiger
Rhaebo nasicus
Rhaebo ecuadorensis
Rhaebo glaberrimus
Rhaebo guttatus
Rhinella spinulosa
Rhinella arequipensis
Rhinella vellardi
Rhinella limensis
Rhinella atacamensis
Rhinella arunco
Rhinella humboldti
Rhinella gr. granulosa
Rhinella ornata
Rhinella poeppigii
Rhinella schneideri
Rhinella marina
Rhinella achavali
Rhinella icterica
Rhinella arenarum
Rhinella veraguensis
Rhinella amboroensis
Rhinella ocellata
Rhinella castaneotica
Rhinella dapsilis
Rhinella cf. margaritifera
Rhinella festae
Rhinella rostrata
Rhinella macrorhina
Rhinella chavin
Rhinella nesiotes
Rhinella manu
Anaxyrus boreas
Anaxyrus nelsoni
Anaxyrus exsul
Anaxyrus canorus
Anaxyrus punctatus
Anaxyrus cognatus
Anaxyrus debilis
Anaxyrus retiformis
Anaxyrus quercicus
Anaxyrus speciosus
Anaxyrus microscaphus
Anaxyrus californicus
Anaxyrus terrestris
Anaxyrus fowleri
Anaxyrus woodhousii
Anaxyrus baxteri
Anaxyrus hemiophrys
Anaxyrus americanus
Anaxyrus houstonensis
Incilius bocourti
Incilius alvarius
Incilius tacanensis
Incilius occidentalis
Incilius perplexus
Incilius canaliferus
Incilius marmoreus
Incilius fastidiosus
Incilius chompipe
Incilius coniferus
Incilius coccifer
Incilius ibarrai
Incilius pisinnus
Incilius cycladen
Incilius signifer
Incilius porteri
Incilius mazatlanensis
Incilius luetkenii
Incilius melanochlorus
Incilius aucoinae
Incilius nebulifer
Incilius valliceps
Incilius cristatus
Incilius spiculatus
Incilius cavifrons
Incilius tutelarius
Incilius leucomyos
Incilius macrocristatus
Incilius campbelli
Vandijkophrynus angusticeps
Vandijkophrynus robinsoni
Vandijkophrynus gariepensis
Vandijkophrynus inyangae
Vandijkophrynus amatolicus
Poyntonophrynus fenoulheti
Poyntonophrynus damaranus
Poyntonophrynus dombensis
Mertensophryne uzunguensis
Mertensophryne taitana
Mertensophryne micranotis
Mertensophryne lindneri
Mertensophryne loveridgei
Mertensophryne anotis
Capensibufo tradouwi
Capensibufo rosei
Sclerophrys mauritanica
Sclerophrys steindachneri
Sclerophrys pantherina
Sclerophrys pardalis
Sclerophrys lemairii
Sclerophrys brauni
Sclerophrys poweri
Poyntonophrynus vertebralis
Sclerophrys tuberosa
Sclerophrys latifrons
Sclerophrys maculata
Sclerophrys regularis
Sclerophrys gutturalis
Sclerophrys xeros
Sclerophrys garmani
Sclerophrys camerunensis
Sclerophrys kisoloensis
Sclerophrys gracilipes
Nectophryne batesii
Nectophryne afra
Werneria tandyi
Werneria mertensiana
Werneria bambutensis
Wolterstorffina parvipalmata
Barbarophryne brongersmai
Leptophryne borbonica
Bufo eichwaldi
Bufo verrucosissimus
Bufo bufo
Bufo tuberospinius
Bufo aspinius
Bufo cryptotympanicus
Bufo japonicus
Bufo torrenticola
Bufo tibetanus
Bufo tuberculatus
Bufo stejnegeri
Bufo bankorensis
Bufo gargarizans
Phrynoidis juxtasper
Phrynoidis asper
Rentapia hosii
Rentapia rugosa
Epidalea calamita
Schismaderma carens
Churamiti maridadi
Nectophrynoides viviparus
Nectophrynoides tornieri
Nectophrynoides minutus
Bufotes siculus
Bufotes boulengeri
Bufotes pewzowi
Bufotes oblongus
Bufotes variabilis
Bufotes viridis
Bufotes balearicus
Strauchbufo raddei
Pedostibes tuberculosus
Adenomus kelaartii
Xanthophryne koynayensis
Duttaphrynus dhufarensis
Duttaphrynus stomaticus
Duttaphrynus hololius
Duttaphrynus stuarti
Duttaphrynus crocus
Duttaphrynus himalayanus
Duttaphrynus atukoralei
Duttaphrynus scaber
Duttaphrynus brevirostris
Duttaphrynus melanostictus
Duttaphrynus parietalis
Sabahphrynus maculatus
Ingerophrynus philippinicus
Ingerophrynus galeatus
Ingerophrynus macrotis
Ingerophrynus celebensis
Ingerophrynus divergens
Ingerophrynus biporcatus
Ghatophryne ornata
Pelophryne misera
Pelophryne brevipes
Pelophryne signata
Didynamipus sjostedti
Nimbaphrynoides occidentalis
Nimbaphrynoides liberiensis
Ansonia muelleri
Ansonia mcgregori
Ansonia guibei
Ansonia fuliginea
Ansonia albomaculata
Ansonia torrentis
Ansonia longidigita
Ansonia leptopus
Ansonia minuta
Ansonia platysoma
Ansonia spinulifer
Ansonia hanitschi
Ansonia malayana
Ansonia penangensis
Ansonia latirostra
Ansonia tiomanica
Ansonia endauensis
Ansonia siamensis
Ansonia inthanon
Ansonia kraensis

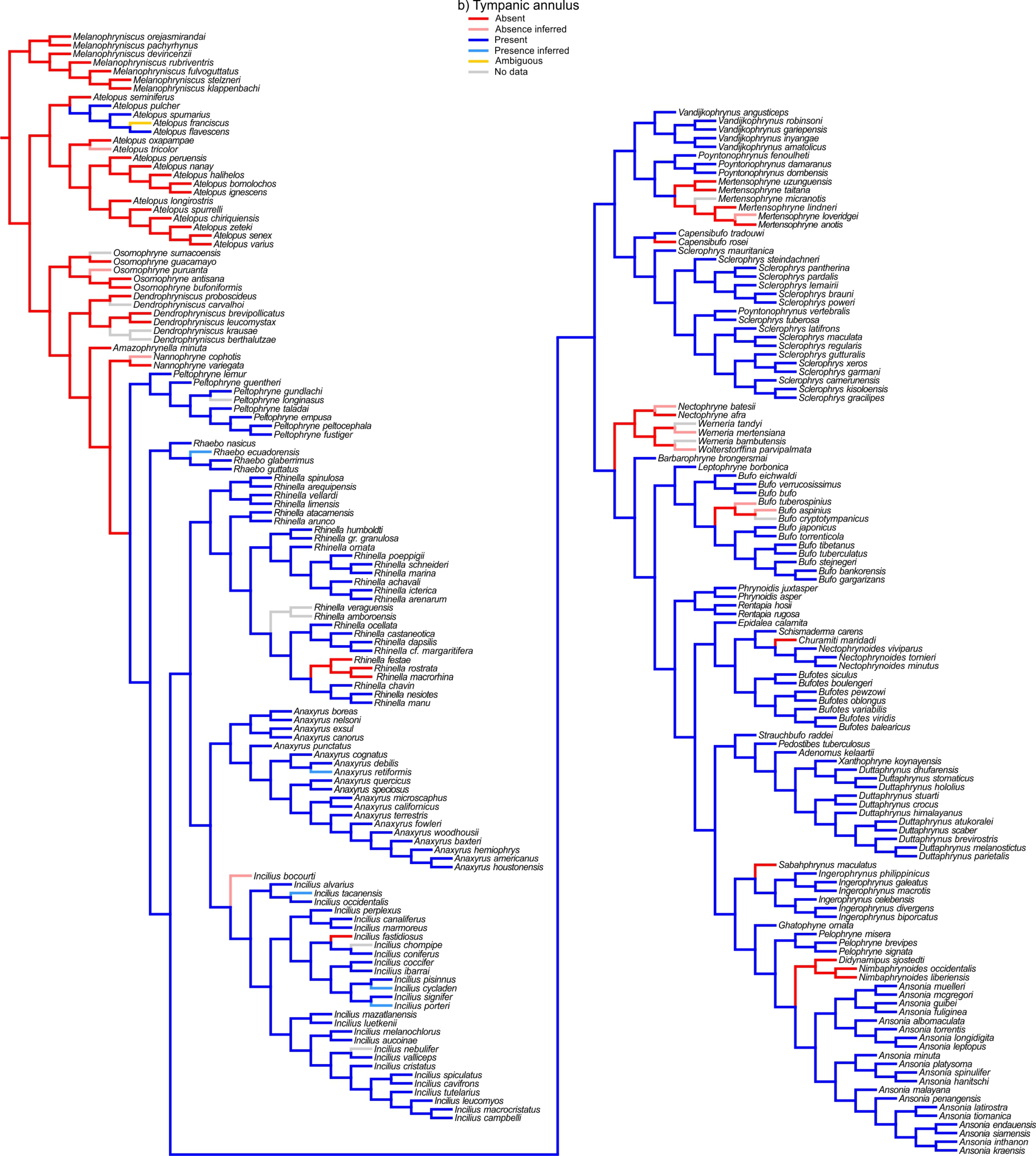
b) Tympanic annulus
Absent
Absence inferred
Present
Presence inferred
Ambiguous
No data
Melanophryniscus orejasmirandai
Melanophryniscus pachyrhynus
Melanophryniscus devincenzii
Melanophryniscus rubriventris
Melanophryniscus fulvoguttatus
Melanophryniscus stelzneri
Melanophryniscus klappenbachi
Atelopus seminiferus
Atelopus pulcher
Atelopus spumarius
Atelopus franciscus
Atelopus flavescens
Atelopus oxapampae
Atelopus tricolor
Atelopus peruensis
Atelopus nanay
Atelopus halihelos
Atelopus bomolochos
Atelopus ignescens
Atelopus longirostris
Atelopus spurrelli
Atelopus chiriquiensis
Atelopus zeteki
Atelopus senex
Atelopus varius
Osornophryne sumacoensis
Osornophryne guacamayo
Osornophryne puruanta
Osornophryne antisana
Osornophryne bufoniformis
Dendrophryniscus proboscideus
Dendrophryniscus carvalhoi
Dendrophryniscus brevipollicatus
Dendrophryniscus leucomystax
Dendrophryniscus krausae
Dendrophryniscus berthalutzae
Amazophrynella minuta
Nannophryne cophotis
Nannophryne variegata
Peltophryne lemur
Peltophryne guentheri
Peltophryne gundlachi
Peltophryne longinasus
Peltophryne taladai
Peltophryne empusa
Peltophryne peltocephala
Peltophryne fustiger
Rhaebo nasicus
Rhaebo ecuadorensis
Rhaebo glaberrimus
Rhaebo guttatus
Rhinella spinulosa
Rhinella arequipensis
Rhinella vellardi
Rhinella limensis
Rhinella atacamensis
Rhinella arunco
Rhinella humboldti
Rhinella gr. granulosa
Rhinella ornata
Rhinella poeppigii
Rhinella schneideri
Rhinella marina
Rhinella achavali
Rhinella icterica
Rhinella arenarum
Rhinella veraguensis
Rhinella amboroensis
Rhinella ocellata
Rhinella castaneotica
Rhinella dapsilis
Rhinella cf. margaritifera
Rhinella festae
Rhinella rostrata
Rhinella macrorhina
Rhinella chavin
Rhinella nesiotes
Rhinella manu
Anaxyrus boreas
Anaxyrus nelsoni
Anaxyrus exsul
Anaxyrus canorus
Anaxyrus punctatus
Anaxyrus cognatus
Anaxyrus debilis
Anaxyrus retiformis
Anaxyrus quercicus
Anaxyrus speciosus
Anaxyrus microscaphus
Anaxyrus californicus
Anaxyrus terrestris
Anaxyrus fowleri
Anaxyrus woodhousii
Anaxyrus baxteri
Anaxyrus hemiophrys
Anaxyrus americanus
Anaxyrus houstonensis
Incilius bocourti
Incilius alvarius
Incilius tacanensis
Incilius occidentalis
Incilius perplexus
Incilius canaliferus
Incilius marmoreus
Incilius fastidiosus
Incilius chompipe
Incilius coniferus
Incilius coccifer
Incilius ibarrai
Incilius pisinnus
Incilius cycladen
Incilius signifer
Incilius porteri
Incilius mazatlanensis
Incilius luetkenii
Incilius melanochlorus
Incilius aucoinae
Incilius nebulifer
Incilius valliceps
Incilius cristatus
Incilius spiculatus
Incilius cavifrons
Incilius tutelarius
Incilius leucomyos
Incilius macrocristatus
Incilius campbelli
Vandijkophrynus angusticeps
Vandijkophrynus robinsoni
Vandijkophrynus gariepensis
Vandijkophrynus inyangae
Vandijkophrynus amatolicus
Poyntonophrynus fenoulheti
Poyntonophrynus damaranus
Poyntonophrynus dombensis
Mertensophryne uzunguensis
Mertensophryne taitana
Mertensophryne micranotis
Mertensophryne lindneri
Mertensophryne loveridgei
Mertensophryne anotis
Capensibufo tradouwi
Capensibufo rosei
Sclerophrys mauritanica
Sclerophrys steindachneri
Sclerophrys pantherina
Sclerophrys pardalis
Sclerophrys lemairii
Sclerophrys brauni
Sclerophrys poweri
Poyntonophrynus vertebralis
Sclerophrys tuberosa
Sclerophrys latifrons
Sclerophrys maculata
Sclerophrys regularis
Sclerophrys gutturalis
Sclerophrys xeros
Sclerophrys garmani
Sclerophrys camerunensis
Sclerophrys kisoloensis
Sclerophrys gracilipes
Nectophryne batesii
Nectophryne afra
Werneria tandyi
Werneria mertensiana
Werneria bambutensis
Wolterstorffina parvipalmata
Barbarophryne brongersmai
Leptophryne borbonica
Bufo eichwaldi
Bufo verrucosissimus
Bufo bufo
Bufo tuberospinius
Bufo aspinius
Bufo cryptotympanicus
Bufo japonicus
Bufo torrenticola
Bufo tibetanus
Bufo tuberculatus
Bufo stejnegeri
Bufo bankorensis
Bufo gargarizans
Phrynoidis juxtasper
Phrynoidis asper
Rentapia hosii
Rentapia rugosa
Epidalea calamita
Schismaderma carens
Churamiti maridadi
Nectophrynoides viviparus
Nectophrynoides tornieri
Nectophrynoides minutus
Bufotes siculus
Bufotes boulengeri
Bufotes pewzowi
Bufotes oblongus
Bufotes variabilis
Bufotes viridis
Bufotes balearicus
Strauchbufo raddei
Pedostibes tuberculosus
Adenomus kelaartii
Xanthophryne koynayensis
Duttaphrynus dhufarensis
Duttaphrynus stomaticus
Duttaphrynus hololius
Duttaphrynus stuarti
Duttaphrynus crocus
Duttaphrynus himalayanus
Duttaphrynus atukoralei
Duttaphrynus scaber
Duttaphrynus brevirostris
Duttaphrynus melanostictus
Duttaphrynus parietalis
Sabahphrynus maculatus
Ingerophrynus philippinicus
Ingerophrynus galeatus
Ingerophrynus macrotis
Ingerophrynus celebensis
Ingerophrynus divergens
Ingerophrynus biporcatus
Ghatophryne ornata
Pelophryne misera
Pelophryne brevipes
Pelophryne signata
Didynamipus sjostedti
Nimbaphrynoides occidentalis
Nimbaphrynoides liberiensis
Ansonia muelleri
Ansonia mcgregori
Ansonia guibei
Ansonia fuliginea
Ansonia albomaculata
Ansonia torrentis
Ansonia longidigita
Ansonia leptopus
Ansonia minuta
Ansonia platysoma
Ansonia spinulifer
Ansonia hanitschi
Ansonia malayana
Ansonia penangensis
Ansonia latirostra
Ansonia tiomanica
Ansonia endauensis
Ansonia siamensis
Ansonia inthanon
Ansonia kraensis

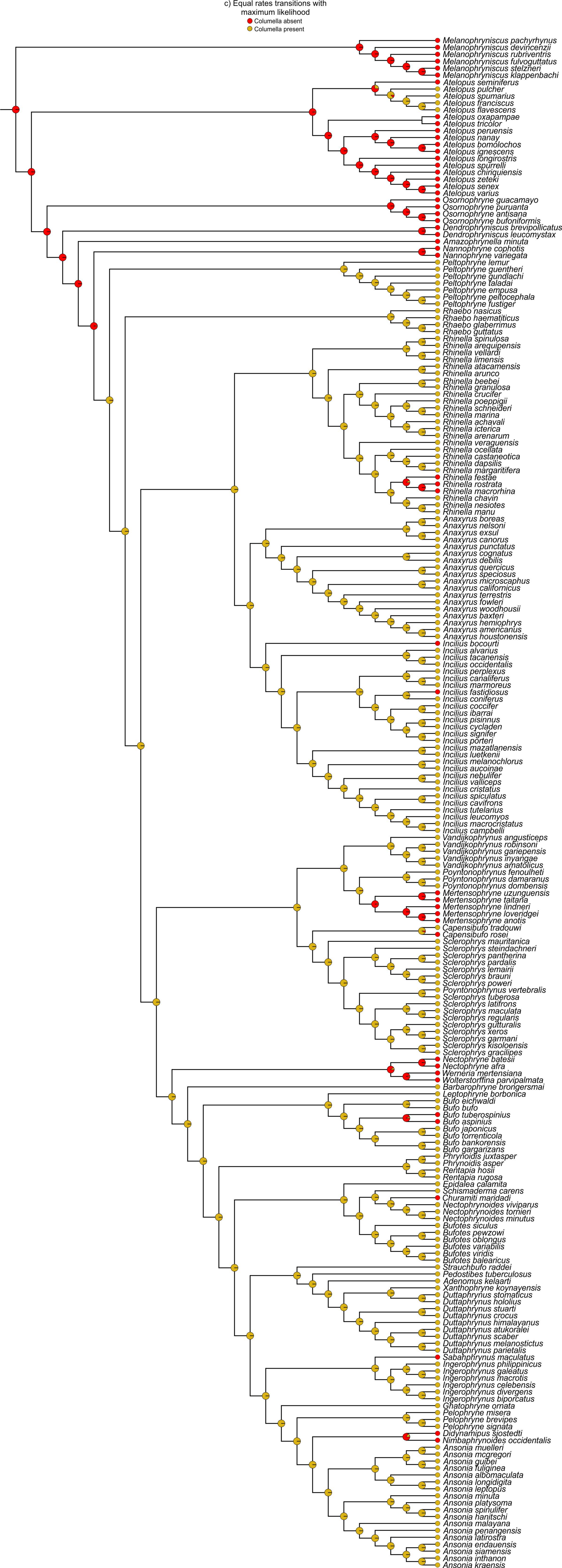

c) Equal rates transitions with maximum likelihood
Columella absent
Columella present
Melanophryniscus pachyrhynus
Melanophryniscus devincenzii
Melanophryniscus rubriventris
Melanophryniscus fulvoguttatus
Melanophryniscus stelzneri
Melanophryniscus klappenbachi
Atelopus seminiferus
Atelopus pulcher
Atelopus spumarius
Atelopus franciscus
Atelopus flavescens
Atelopus oxapampae
Atelopus tricolor
Atelopus peruensis
Atelopus nanay
Atelopus bomolochos
Atelopus ignescens
Atelopus longirostris
Atelopus spurrelli
Atelopus chiriquiensis
Atelopus zeteki
Atelopus senex
Atelopus varius
Osornophryne guacamayo
Osornophryne puruanta
Osornophryne antisana
Osornophryne bufoniformis
Dendrophryniscus brevipollicatus
Dendrophryniscus leucomystax
Amazophrynella minuta
Nannophryne cophotis
Nannophryne variegata
Peltophryne lemur
Peltophryne guentheri
Peltophryne gundlachi
Peltophryne taladai
Peltophryne empusa
Peltophryne peltocephala
Peltophryne fustiger
Rhaebo nasicus
Rhaebo haematiticus
Rhaebo glaberrimus
Rhaebo guttatus
Rhinella spinulosa
Rhinella arequipensis
Rhinella vellardi
Rhinella limensis
Rhinella atacamensis
Rhinella arunco
Rhinella beebei
Rhinella granulosa
Rhinella crucifer
Rhinella poeppigii
Rhinella schneideri
Rhinella marina
Rhinella achavali
Rhinella icterica
Rhinella arenarum
Rhinella veraguensis
Rhinella ocellata
Rhinella castaneotica
Rhinella dapsilis
Rhinella margaritifera
Rhinella festae
Rhinella rostrata
Rhinella macrorhina
Rhinella chavin
Rhinella nesiotes
Rhinella manu
Anaxyrus boreas
Anaxyrus nelsoni
Anaxyrus exsul
Anaxyrus canorus
Anaxyrus punctatus
Anaxyrus cognatus
Anaxyrus debilis
Anaxyrus quercicus
Anaxyrus speciosus
Anaxyrus microscaphus
Anaxyrus californicus
Anaxyrus terrestris
Anaxyrus fowleri
Anaxyrus woodhousii
Anaxyrus baxteri
Anaxyrus hemiophrys
Anaxyrus americanus
Anaxyrus houstonensis
Incilius bocourti
Incilius alvarius
Incilius tacanensis
Incilius occidentalis
Incilius perplexus
Incilius canaliferus
Incilius marmoreus
Incilius fastidiosus
Incilius coniferus
Incilius coccifer
Incilius ibarrai
Incilius pisinnus
Incilius cycladen
Incilius signifer
Incilius porteri
Incilius mazatlanensis
Incilius luetkenii
Incilius melanochlorus
Incilius aucoinae
Incilius nebulifer
Incilius valliceps
Incilius cristatus
Incilius spiculatus
Incilius cavifrons
Incilius tutelarius
Incilius leucomyos
Incilius macrocristatus
Incilius campbelli
Vandijkophrynus angusticeps
Vandijkophrynus robinsoni
Vandijkophrynus gariepensis
Vandijkophrynus inyangae
Vandijkophrynus amatolicus
Poyntonophrynus fenoulheti
Poyntonophrynus damaranus
Poyntonophrynus dombensis
Mertensophryne uzunguensis
Mertensophryne taitana
Mertensophryne lindneri
Mertensophryne loveridgei
Mertensophryne anotis
Capensibufo tradouwi
Capensibufo rosei
Sclerophrys mauritanica
Sclerophrys steindachneri
Sclerophrys pantherina
Sclerophrys pardalis
Sclerophrys lemairii
Sclerophrys brauni
Sclerophrys poweri
Poyntonophrynus vertebralis
Sclerophrys tuberosa
Sclerophrys latifrons
Sclerophrys maculata
Sclerophrys regularis
Sclerophrys gutturalis
Sclerophrys xeros
Sclerophrys garmani
Sclerophrys kisoloensis
Sclerophrys gracilipes
Nectophryne batesii
Nectophryne afra
Werneria mertensiana
Wolterstorffina parvipalmata
Barbarophryne brongersmai
Leptophryne borbonica
Bufo eichwaldi
Bufo bufo
Bufo tuberospinius
Bufo aspinius
Bufo japonicus
Bufo torrenticola
Bufo bankorensis
Bufo gargarizans
Phrynoidis juxtasper
Phrynoidis asper
Rentapia hosii
Rentapia rugosa
Epidalea calamita
Schismaderma carens
Churamiti maridadi
Nectophrynoides viviparus
Nectophrynoides tornieri
Nectophrynoides minutus
Bufotes siculus
Bufotes pewzowi
Bufotes oblongus
Bufotes variabilis
Bufotes viridis
Bufotes balearicus
Strauchbufo raddei
Pedostibes tuberculosus
Adenomus kelaarti
Xanthophryne koynayensis
Duttaphrynus stomaticus
Duttaphrynus hololius
Duttaphrynus stuarti
Duttaphrynus crocus
Duttaphrynus himalayanus
Duttaphrynus atukoralei
Duttaphrynus scaber
Duttaphrynus melanostictus
Duttaphrynus parietalis
Sabahphrynus maculatus
Ingerophrynus philippinicus
Ingerophrynus galeatus
Ingerophrynus macrotis
Ingerophrynus celebensis
Ingerophrynus divergens
Ingerophrynus biporcatus
Ghatophryne ornata
Pelophryne misera
Pelophryne brevipes
Pelophryne signata
Didynamipus sjostedti
Nimbaphrynoides occidentalis
Ansonia muelleri
Ansonia mcgregori
Ansonia guibei
Ansonia fuliginea
Ansonia albomaculata
Ansonia longidigita
Ansonia leptopus
Ansonia minuta
Ansonia platysoma
Ansonia spinulifer
Ansonia hanitschi
Ansonia malayana
Ansonia penangensis
Ansonia latirostra
Ansonia endauensis
Ansonia siamensis
Ansonia inthanon
Ansonia kraensis

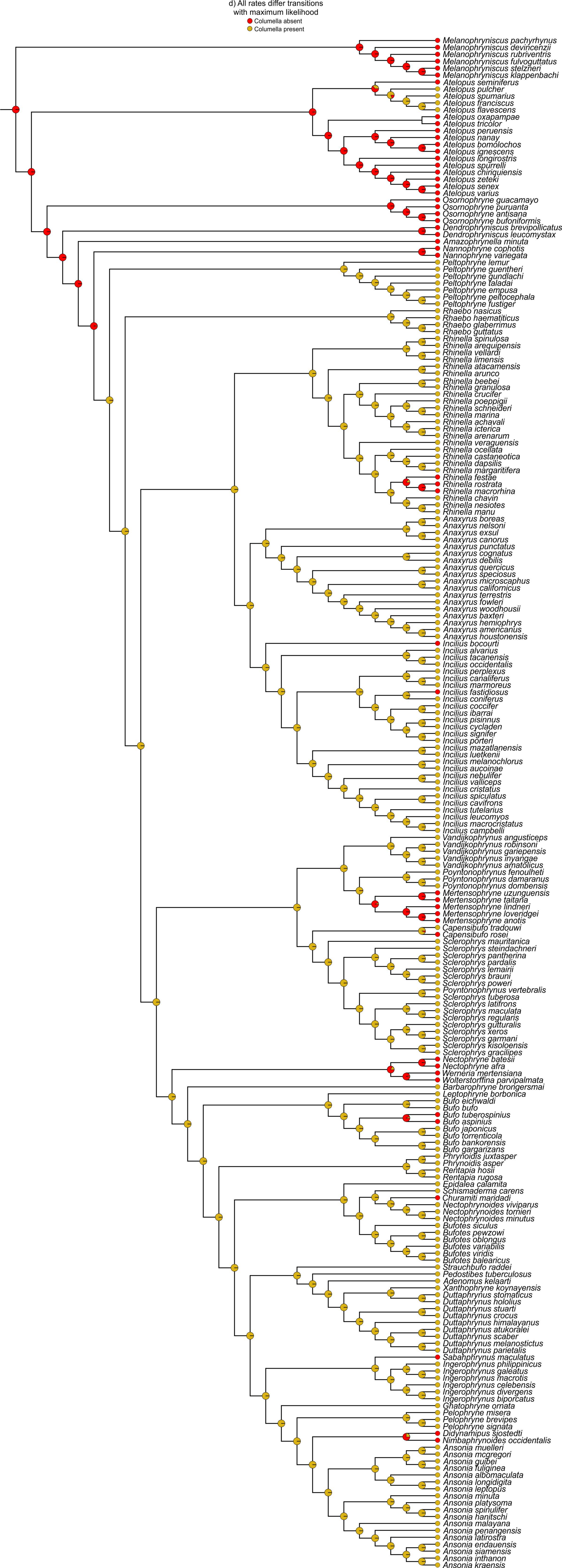
d) All rates differ transitions
with maximum likelihood
Columella absent
Columella present
Melanophryniscus pachyrhynus
Melanophryniscus devincenzii
Melanophryniscus rubriventris
Melanophryniscus fulvoguttatus
Melanophryniscus stelzneri
Melanophryniscus klappenbachi
Atelopus seminiferus
Atelopus pulcher
Atelopus spumarius
Atelopus franciscus
Atelopus flavescens
Atelopus oxapampae
Atelopus tricolor
Atelopus peruensis
Atelopus nanay
Atelopus bomolochos
Atelopus ignescens
Atelopus longirostris
Atelopus spurrelli
Atelopus chiriquiensis
Atelopus zeteki
Atelopus senex
Atelopus varius
Osornophryne guacamayo
Osornophryne puruanta
Osornophryne antisana
Osornophryne bufoniformis
Dendrophryniscus brevipollicatus
Dendrophryniscus leucomystax
Amazophrynella minuta
Nannophryne cophotis
Nannophryne variegata
Peltophryne lemur
Peltophryne guentheri
Peltophryne gundlachi
Peltophryne taladai
Peltophryne empusa
Peltophryne peltocephala
Peltophryne fustiger
Rhaebo nasicus
Rhaebo haematiticus
Rhaebo glaberrimus
Rhaebo guttatus
Rhinella spinulosa
Rhinella arequipensis
Rhinella vellardi
Rhinella limensis
Rhinella atacamensis
Rhinella arunco
Rhinella beebei
Rhinella granulosa
Rhinella crucifer
Rhinella poeppigii
Rhinella schneideri
Rhinella marina
Rhinella achavali
Rhinella icterica
Rhinella arenarum
Rhinella veraguensis
Rhinella ocellata
Rhinella castaneotica
Rhinella dapsilis
Rhinella margaritifera
Rhinella festae
Rhinella rostrata
Rhinella macrorhina
Rhinella chavin
Rhinella nesiotes
Rhinella manu
Anaxyrus boreas
Anaxyrus nelsoni
Anaxyrus exsul
Anaxyrus canorus
Anaxyrus punctatus
Anaxyrus cognatus
Anaxyrus debilis
Anaxyrus quercicus
Anaxyrus speciosus
Anaxyrus microscaphus
Anaxyrus californicus
Anaxyrus terrestris
Anaxyrus fowleri
Anaxyrus woodhousii
Anaxyrus baxteri
Anaxyrus hemiophrys
Anaxyrus americanus
Anaxyrus houstonensis
Incilius bocourti
Incilius alvarius
Incilius tacanensis
Incilius occidentalis
Incilius perplexus
Incilius canaliferus
Incilius marmoreus
Incilius fastidiosus
Incilius coniferus
Incilius coccifer
Incilius ibarrai
Incilius pisinnus
Incilius cycladen
Incilius signifer
Incilius porteri
Incilius mazatlanensis
Incilius luetkenii
Incilius melanochlorus
Incilius aucoinae
Incilius nebulifer
Incilius valliceps
Incilius cristatus
Incilius spiculatus
Incilius cavifrons
Incilius tutelarius
Incilius leucomyos
Incilius macrocristatus
Incilius campbelli
Vandijkophrynus angusticeps
Vandijkophrynus robinsoni
Vandijkophrynus gariepensis
Vandijkophrynus inyangae
Vandijkophrynus amatolicus
Poyntonophrynus fenoulheti
Poyntonophrynus damaranus
Poyntonophrynus dombensis
Mertensophryne uzunguensis
Mertensophryne taitana
Mertensophryne lindneri
Mertensophryne loveridgei
Mertensophryne anotis
Capensibufo tradouwi
Capensibufo rosei
Sclerophrys mauritanica
Sclerophrys steindachneri
Sclerophrys pantherina
Sclerophrys pardalis
Sclerophrys lemairii
Sclerophrys brauni
Sclerophrys poweri
Poyntonophrynus vertebralis
Sclerophrys tuberosa
Sclerophrys latifrons
Sclerophrys maculata
Sclerophrys regularis
Sclerophrys gutturalis
Sclerophrys xeros
Sclerophrys garmani
Sclerophrys kisoloensis
Sclerophrys gracilipes
Nectophryne batesii
Nectophryne afra
Werneria mertensiana
Wolterstorffina parvipalmata
Barbarophryne brongersmai
Leptophryne borbonica
Bufo eichwaldi
Bufo bufo
Bufo tuberospinius
Bufo aspinius
Bufo japonicus
Bufo torrenticola
Bufo bankorensis
Bufo gargarizans
Phrynoidis juxtasper
Phrynoidis asper
Rentapia hosii
Rentapia rugosa
Epidalea calamita
Schismaderma carens
Churamiti maridadi
Nectophrynoides viviparus
Nectophrynoides tornieri
Nectophrynoides minutus
Bufotes siculus
Bufotes pewzowi
Bufotes oblongus
Bufotes variabilis
Bufotes viridis
Bufotes balearicus
Strauchbufo raddei
Pedostibes tuberculosus
Adenomus kelaarti
Xanthophryne koynayensis
Duttaphrynus stomaticus
Duttaphrynus hololius
Duttaphrynus stuarti
Duttaphrynus crocus
Duttaphrynus himalayanus
Duttaphrynus atukoralei
Duttaphrynus scaber
Duttaphrynus melanostictus
Duttaphrynus parietalis
Sabahphrynus maculatus
Ingerophrynus philippinicus
Ingerophrynus galeatus
Ingerophrynus macrotis
Ingerophrynus celebensis
Ingerophrynus divergens
Ingerophrynus biporcatus
Ghatophryne ornata
Pelophryne misera
Pelophryne brevipes
Pelophryne signata
Didynamipus sjostedti
Nimbaphrynoides occidentalis
Ansonia muelleri
Ansonia mcgregori
Ansonia guibei
Ansonia fuliginea
Ansonia albomaculata
Ansonia longidigita
Ansonia leptopus
Ansonia minuta
Ansonia platysoma
Ansonia spinulifer
Ansonia hanitschi
Ansonia malayana
Ansonia penangensis
Ansonia latirostra
Ansonia endauensis
Ansonia siamensis
Ansonia inthanon
Ansonia kraensis

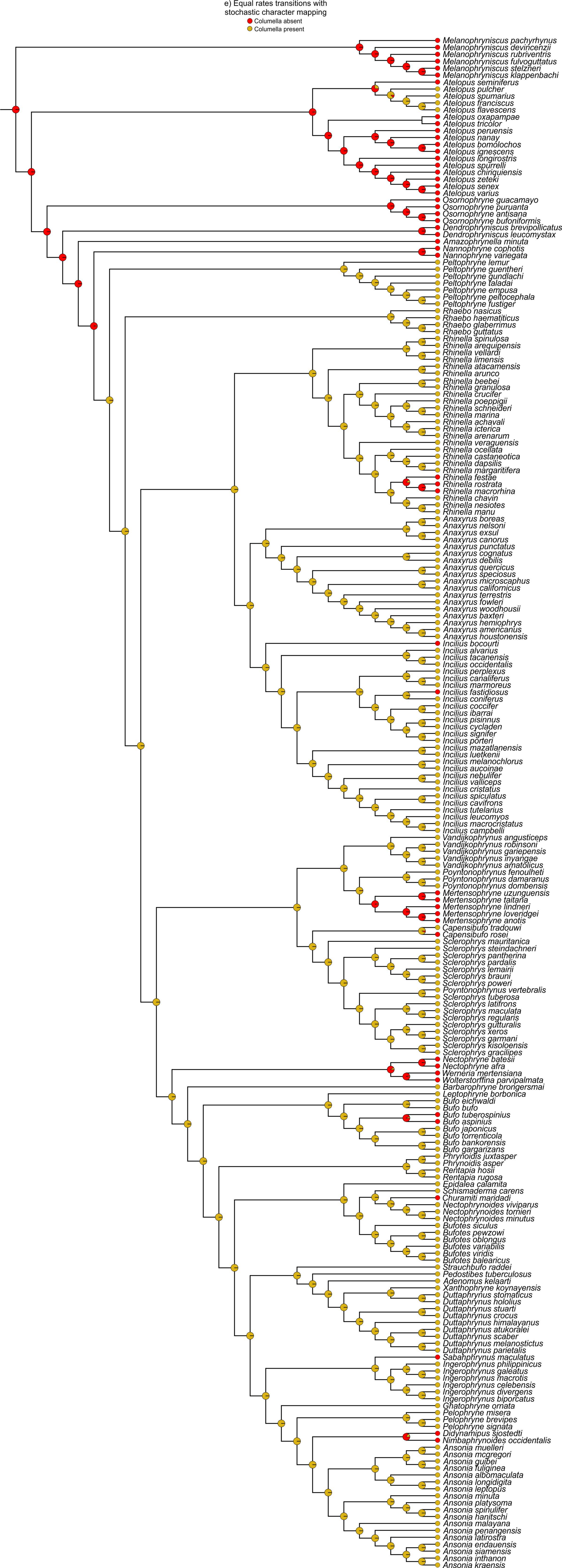
e) Equal rates transitions with
stochastic character mapping
Columella absent
Columella present
Melanophryniscus pachyrhynus
Melanophryniscus devincenzii
Melanophryniscus rubriventris
Melanophryniscus fulvoguttatus
Melanophryniscus stelzneri
Melanophryniscus klappenbachi
Atelopus seminiferus
Atelopus pulcher
Atelopus spumarius
Atelopus franciscus
Atelopus flavescens
Atelopus oxapampae
Atelopus tricolor
Atelopus peruensis
Atelopus nanay
Atelopus bomolochos
Atelopus ignescens
Atelopus longirostris
Atelopus spurrelli
Atelopus chiriquiensis
Atelopus zeteki
Atelopus senex
Atelopus varius
Osornophryne guacamayo
Osornophryne puruanta
Osornophryne antisana
Osornophryne bufoniformis
Dendrophryniscus brevipollicatus
Dendrophryniscus leucomystax
Amazophrynella minuta
Nannophryne cophotis
Nannophryne variegata
Peltophryne lemur
Peltophryne guentheri
Peltophryne gundlachi
Peltophryne taladai
Peltophryne empusa
Peltophryne peltocephala
Peltophryne fustiger
Rhaebo nasicus
Rhaebo haematiticus
Rhaebo glaberrimus
Rhaebo guttatus
Rhinella spinulosa
Rhinella arequipensis
Rhinella vellardi
Rhinella limensis
Rhinella atacamensis
Rhinella arunco
Rhinella beebei
Rhinella granulosa
Rhinella crucifer
Rhinella poeppigii
Rhinella schneideri
Rhinella marina
Rhinella achavali
Rhinella icterica
Rhinella arenarum
Rhinella veraguensis
Rhinella ocellata
Rhinella castaneotica
Rhinella dapsilis
Rhinella margaritifera
Rhinella festae
Rhinella rostrata
Rhinella macrorhina
Rhinella chavin
Rhinella nesiotes
Rhinella manu
Anaxyrus boreas
Anaxyrus nelsoni
Anaxyrus exsul
Anaxyrus canorus
Anaxyrus punctatus
Anaxyrus cognatus
Anaxyrus debilis
Anaxyrus quercicus
Anaxyrus speciosus
Anaxyrus microscaphus
Anaxyrus californicus
Anaxyrus terrestris
Anaxyrus fowleri
Anaxyrus woodhousii
Anaxyrus baxteri
Anaxyrus hemiophrys
Anaxyrus americanus
Anaxyrus houstonensis
Incilius bocourti
Incilius alvarius
Incilius tacanensis
Incilius occidentalis
Incilius perplexus
Incilius canaliferus
Incilius marmoreus
Incilius fastidiosus
Incilius coniferus
Incilius coccifer
Incilius ibarrai
Incilius pisinnus
Incilius cycladen
Incilius signifer
Incilius porteri
Incilius mazatlanensis
Incilius luetkenii
Incilius melanochlorus
Incilius aucoinae
Incilius nebulifer
Incilius valliceps
Incilius cristatus
Incilius spiculatus
Incilius cavifrons
Incilius tutelarius
Incilius leucomyos
Incilius macrocristatus
Incilius campbelli
Vandijkophrynus angusticeps
Vandijkophrynus robinsoni
Vandijkophrynus gariepensis
Vandijkophrynus inyangae
Vandijkophrynus amatolicus
Poyntonophrynus fenoulheti
Poyntonophrynus damaranus
Poyntonophrynus dombensis
Mertensophryne uzunguensis
Mertensophryne taitana
Mertensophryne lindneri
Mertensophryne loveridgei
Mertensophryne anotis
Capensibufo tradouwi
Capensibufo rosei
Sclerophrys mauritanica
Sclerophrys steindachneri
Sclerophrys pantherina
Sclerophrys pardalis
Sclerophrys lemairii
Sclerophrys brauni
Sclerophrys poweri
Poyntonophrynus vertebralis
Sclerophrys tuberosa
Sclerophrys latifrons
Sclerophrys maculata
Sclerophrys regularis
Sclerophrys gutturalis
Sclerophrys xeros
Sclerophrys garmani
Sclerophrys kisoloensis
Sclerophrys gracilipes
Nectophryne batesii
Nectophryne afra
Werneria mertensiana
Wolterstorffina parvipalmata
Barbarophryne brongersmai
Leptophryne borbonica
Bufo eichwaldi
Bufo bufo
Bufo tuberospinius
Bufo aspinius
Bufo japonicus
Bufo torrenticola
Bufo bankorensis
Bufo gargarizans
Phrynoidis juxtasper
Phrynoidis asper
Rentapia hosii
Rentapia rugosa
Epidalea calamita
Schismaderma carens
Churamiti maridadi
Nectophrynoides viviparus
Nectophrynoides tornieri
Nectophrynoides minutus
Bufotes siculus
Bufotes pewzowi
Bufotes oblongus
Bufotes variabilis
Bufotes viridis
Bufotes balearicus
Strauchbufo raddei
Pedostibes tuberculosus
Adenomus kelaarti
Xanthophryne koynayensis
Duttaphrynus stomaticus
Duttaphrynus hololius
Duttaphrynus stuarti
Duttaphrynus crocus
Duttaphrynus himalayanus
Duttaphrynus atukoralei
Duttaphrynus scaber
Duttaphrynus melanostictus
Duttaphrynus parietalis
Sabahphrynus maculatus
Ingerophrynus philippinicus
Ingerophrynus galeatus
Ingerophrynus macrotis
Ingerophrynus celebensis
Ingerophrynus divergens
Ingerophrynus biporcatus
Ghatophryne ornata
Pelophryne misera
Pelophryne brevipes
Pelophryne signata
Didynamipus sjostedti
Nimbaphrynoides occidentalis
Ansonia muelleri
Ansonia mcgregori
Ansonia guibei
Ansonia fuliginea
Ansonia albomaculata
Ansonia longidigita
Ansonia leptopus
Ansonia minuta
Ansonia platysoma
Ansonia spinulifer
Ansonia hanitschi
Ansonia malayana
Ansonia penangensis
Ansonia latirostra
Ansonia endauensis
Ansonia siamensis
Ansonia inthanon
Ansonia kraensis

f) Equal rates transitions + "eared" ancestor with stochastic character mapping

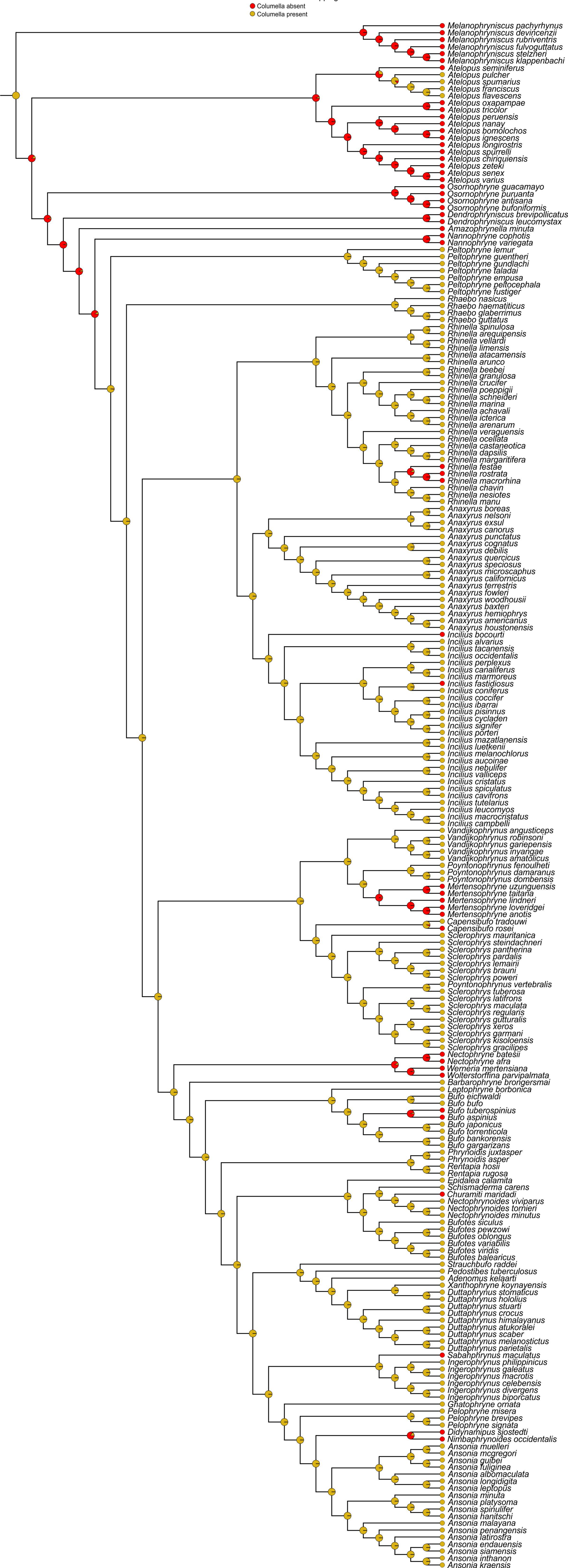

g) Dollo's law (no regains) with stochastic character mapping

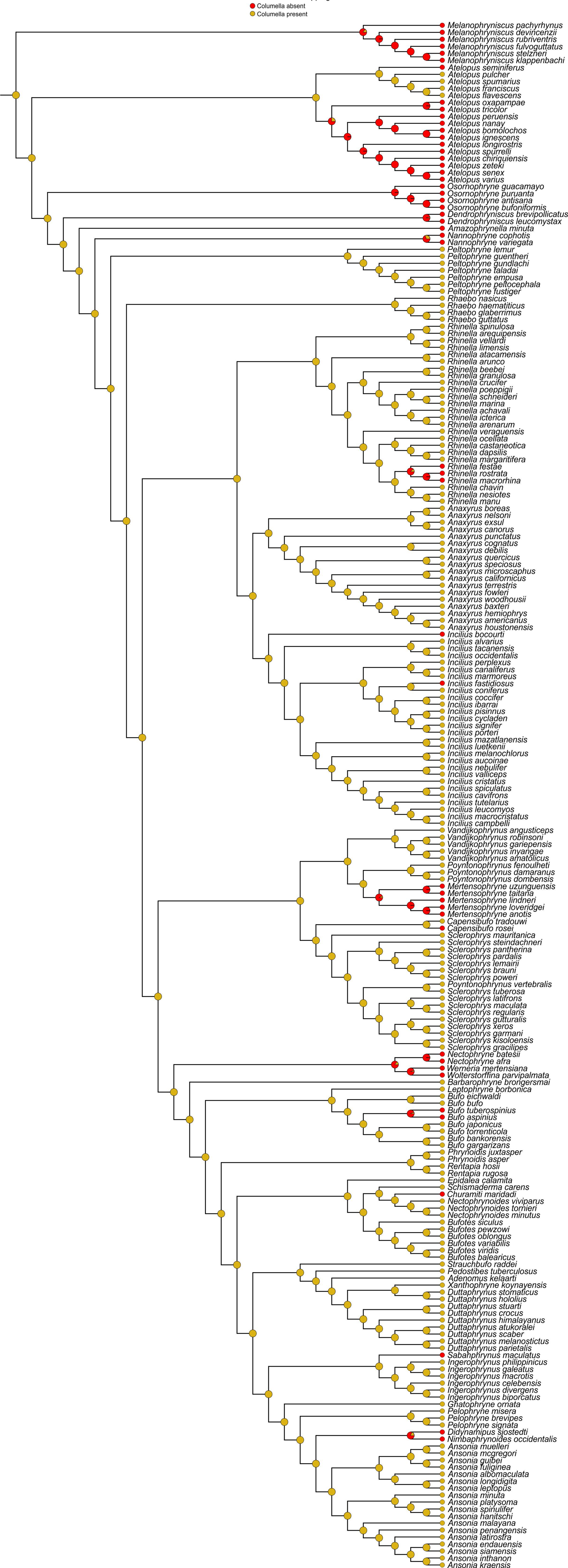

S3.2—Comparison of estimated tympanic middle ear transformations across parsimony, maximum likelihood analyses and Bayesian stochastic mutational mapping analyses on phylogenies.

For the maximum likelihood analyses we tested models in which transition rates from presence/absence of columella were allowed to differ across the tree (ARD) and a model in which transition rates were equal (ER). The equal transition rates model had a lower AIC value and was therefore used in all Bayesian models. All the unconstrained analyses (parsimony, ARD and ER maximum likelihood, ER SIMMAP) support an ancestor lacking columella with two subsequent regains in Bufonidae: one within *Atelopus* and one within the sister clade of *Nannophryne*. When the ancestor is constrained to having a complete middle ear, the SIMMAP model supports only one regain of the middle ear within *Atelopus*. When no regains are allowed (Dollo's law), the SIMMAP model supports 17 total losses of columella. N/A: not applicable.

**Model**	**AIC**	**# of losses**	**# of regains**	**Total transitions**	**Support for earless ancestor (Yes/No, %)**
Parsimony	N/A	10	2	12	Yes, N/A
ARD	108.362	10	2	12	Yes, 99.499%
ER	106.849	10	2	12	Yes, 99.592%
ER SIMMAP	N/A	10	2	12	Yes, 99.995%
ER + "eared" ancestor SIMMAP	N/A	12	2	14	No by design
Dollo's Law SIMMAP	N/A	17	0	17	Not possible

Section S4. Detail of the clades or species of Bufonidae where tympanic middle ear transformations occurred

S4.1—Detail of the clades or species of Bufonidae where tympanic middle ear transformations occurred according to the ancestral state reconstructions shown in figure 2 and Section S3.

- Tympanic membrane:

Within Bufonidae a regain of this structure occurred in the sister clade of *Nannophryne*. Subsequent independent losses occurred at least 25 times in the following taxa: at least four times (and up to five, depending on alternative resolution of the ambiguity) within *Incilius,* three times in *Ansonia,* three times in *Rhinella,* at least once (and up to four times) in the clade *Leptophryne borbonica* + *Bufo,* once in each of the clades *Didynamipus* + *Nimbaphrynoides*, *Nectophryne* + *Werneria* + *Wolterstorffina*, and *Mertensophryne*, and in each of the species *Adenomus kelaartii*, *Anaxyrus exsul*, *Capensibufo rosei*, *Churamiti maridadi*, *Duttaphrynus himalayanus*, *Epidalea calamita*, *Peltophryne longinasus*, *Poyntonophrynus damaranus*, *Rhaebo nasicus*, *Sabahphrynus maculatus*, and *Vandijkophrynus robinsoni.*

- Tympanic annulus and columella:

From the ancestral condition there were two independent reappearances of these structures: (a) within a derived clade of *Atelopus*, the *Atelopus flavescens-spumarius* clade, composed by *A. flavescens*, *A. franciscus*, *A. pulcher*, and *A. spumarius*, and (b) in the sister clade of *Nannophryne*. Ten independent secondary losses occurred within the last clade in the following taxa: *Bufo tuberospinius* + *B. aspinius* + *B. cryptotympanicus*, *Capensibufo rosei*, *Churamiti maridadi*, *Didynamipus* + *Nimbaphrynoides*, *Incilius bocourti*, *I. fastidiosus*, *Mertensophryne*, *Nectophryne* + *Werneria* + *Wolterstorffina*, *Rhinella festae* + *R. macrorhina* + *R. rostrata*, and *Sabahphrynus maculatus*.

S4.2—Inferred transformations in tympanic middle ear structures and relationships in bufonids analyzed in published phylogenetic analyses but not included by Pyron (2014).

- Taxa that affect the number of transformations inferred inferred and disscussed on Section S4.1:

(a) *Frostius erythrophthalmus.* This species (and also its closely related species *F. pernambucensis*), has a tympanic membrane, tympanic annulus, and columella, and was recovered as the sister taxon of *Oreophrynella* by Peloso *et al*. (2012) and Ron *et al*. (2015). The conditions of *Frostius* add one independent regain of tympanic middle ear structures in Bufonidae.

(b) *Rhaebo colomai* and *R. olallai*. Ron *et al*. (2015) synonymized the genus *Andinophryne* with *Rhaebo* based on results of a phylogenetic analysis. *Rhaebo colomai* and *R. olallai* were recovered nested within the remaining species of *Rhaebo*. Both species lack a tympanic membrane but retain a tympanic annulus and columella. This situation left unresolved the number of independent losses of tympanic membrane within *Rhaebo* (once or twice) but does not modify the number of steps for the two transformation series.

(c) *Parapelophryne scalpta*. This species was recovered as sister taxon of *Bufo* in the phylogenetic analysis of Matsui *et al*. (2015). It lacks a tympanic membrane but retains a tympanic annulus and columella. The lack of a tympanic membrane in this species affects the ancestral character reconstruction of this character, because it resolves the ambiguities in *Bufo* + *Leptophryne borbonica*. Thus, the lack of tympanic membrane is the plesiomorphic condition for the clade *Leptophryne* + *Parapelophryne* + *Bufo*, with three independent regains in *Bufo*. The ancestral character reconstructions for tympanic annulus and columella are not modified.

(d) *Bufotes latastii*, *B. luristanicus*, *B. pseudoraddei*, and *B. surdus* were included in a phylogenetic analysis by Portik and Papenfuss (2015), in which it was recovered nested within a monophyletic *Bufotes*. The lack of a differentiated tympanic membrane in *B. surdus* adds one additional step only in this transformation series.

(e) The clade *Bufoides meghalayanus* + *Blythophryne beryet* was recovered as the sister clade of *Xanthophryne koynayensis* by Chandramouli *et al*. (2016). The lack of a differentiated tympanic membrane in *B. meghalayanus* adds one additional step in this transformation series. The conditions for tympanic annulus and columella in *Bufoides meghalayanus* are unknown.

- Taxa that do not affect the number of transformations inferred and disscussed on Section S4.1:

(a) *Oreophrynella.* Species of this monophyletic genus (Kok *et al*. 2012) lack tympanic middle ear structures and have been recovered as the sister clade of *Atelopus* in the phylogenetic analyses of Pramuk (2006) and van Bocxlaer *et al*. (2010).

(b) *Amazophrynella amazonicola*, *A. bokermanni, A. javierbustamantei*, *A. manaos, A. matses*, and *A. vote*. All of these species lack at least the tympanic membrane. Phylogenetic studies for these species were done by Rojas *et al*. (2014, 2015, 2016) who recovered *Amazophrynella* as monophyletic.

(c) *Adenomus kandianus*. This species lacks a tympanic membrane but possesses a tympanic annulus and columella. It is the sister taxon to *A. kelaartii* according to Meegaskumbura *et al*. (2015).

(d) *Sclerophrys arabica*, *S. asmarae*, *S. blandfordii*, *S. capensis*, *S. dodsoni*, *S. pentoni*, *Duttaphrynus dhufarensis* and *D. olivaceus.* All these species were included in the phylogenetic analysis of Portik and Papenfuss (2015).

(e) *Ansonia lumut* and *A. vidua*, which were recovered nested in *Ansonia* by Chan *et al*. (2014) and Hertwig *et al*. (2014) respectively. Both species possess a complete tympanic middle ear.

(f) *Rhinella alata*, which possesses a complete tympanic middle ear, is recovered nested in the *R. margaritifera* group by Santos *et al*. (2015).

(g) *Rhinella azarai*, *R. bernardoi*, *R. bergi*, *R. centralis*, *R. dorbignyi*, *R. fernandezae*, *R. major*, *R. merianae*, *R. mirandaribeiroi*, and *R. pygmaea* were recovered within a monophyletic *R. granulosa* group; *R. henseli* as sister taxon of *R. crucifer*; and *R. sternosignata* as sister taxon of the clade composed by the species of the *R. margaritifera* + *R. veraguensis* groups by Pereyra *et al*. (2016). All of these species possess complete tympanic middle ears.

S4.3—References

Chan K. O., Wodd, P. L., Anuar, S., Muin, M. A., Quah, E. S. H., Sumaril, A. X. & Grismer, L. L. (2014). A new species of upland Stream Toad of the genus *Ansonia* Stoliczka, 1870 (Anura: Bufonidae) from northeastern Peninsular Malaysia. *Zootaxa* **3764,** 427–440.

Chandramouli S. R., Vasudevan, K., Harikrishnan, S., Dutta, S. K., Janani, S. J., Sharma, R., Das, I. & Aggarwal, R. K. (2016). A new genus and species of arboreal toad with phytotelmonous larvae, from the Andaman Islands, India (Lissamphibia, Anura, Bufonidae). *ZooKeys* **555,** 57–90.

Hertwig S., Min, P., Haas, A. & Das, I. (2014). Dressed in black. A New *Ansonia* Stoliczka, 1870 (Lissamphibia: Anura: Bufonidae) from Gunung Murud, Sarawak, east Malaysia (Borneo). *Zootaxa* **3814,** 419–431.

Kok P., MacCulloch, R., Means, D., Roelants, K., Van Bocxlaer, I. & Bossuyt, F. (2012). Low genetic diversity in tepui summit vertebrates. *Current Biology* **22,** R589–R590.

Matsui M., Eto, K., Lau, M., Liu, W. & Nishikawa, K. (2015). Unexpected phylogenetic position of *Parapelophryne* among Southeast Asian bufonids as revealed by mitochondrial DNA sequence (Amphibia, Anura, Bufonidae). *Current Herpetology* **34,** 182–187.

Peloso P. L. V., Faivovich, J., Grant, T., Gasparini, J. L. & Haddad, C. F. B. (2012). An extraordinary new species of *Melanophryniscus* (Anura, Bufonidae) from southeastern Brazil. *American Museum Novitates* **3762,** 1–31.

Pereyra M. O., Baldo, D., Blotto, B. L., Iglesias, P. P., Thomé, M. T. C., Haddad, C. F. B., Barrio-Amorós, C., Ibáñez, R. & Faivovich, J. (2016). Phylogenetic relationships of toads of the *Rhinella granulosa* group (Anura: Bufonidae): a molecular perspective with comments on hybridization and introgression. *Cladistics* **32,** 36–53.

Portik D. M. & Papenfuss, T. J. (2015). Historical biogeography resolves the origins of endemic arabian toad lineages (Anura: Bufonidae): evidence for ancient vicariance and dispersal events with the horn of Africa and South Asia. *BMC Evolutionary Biology* **15,** 152[1–19].

Rojas R., Carvalho, V., Gordo, M., Ávila, R., Farias, I. & Hrbek, T. (2014). A new species of *Amazophrynella* (Anura: Bufonidae) from the southwestern part of the Brazilian Guiana Shield. *Zootaxa* **3753,** 79–95.

Rojas R., Carvalho, V., Ávila, R., Farias, I., Gordo, M. & Hrbek, T. (2015). Two new species of *Amazophrynella* (Amphibia: Anura: Bufonidae) from Loreto, Peru. *Zootaxa* **3946,** 79–103.

Ron S. R., Mueses-Cisneros, J. J., Gutiérrez-Cárdenas, P. D. A., Rojas-Rivera, A., Lynch, R. L., Rocha, C. F. D. & Galarza, G. (2015). Systematics of the endangered toad genus *Andinophryne*

(Anura: Bufonidae): phylogenetic position and synonymy under the genus *Rhaebo*. *Zootaxa* **3947,** 347–366.

Santos S., Ibañez, R. & Ron, S. (2015). Systematics of the *Rhinella margaritifera* complex (Anura, Bufonidae) from western Ecuador and Panama with insights in the biogeography of *Rhinella alata*. *ZooKeys* **501,** 109–145.

van Bocxlaer I., Loader, S. P., Roelants, K., Biju, S. D., Menegon, M. & Bossuyt, F. (2010). Gradual adaptation toward a range-expansion phenotype initiated the global radiation of toads. *Science* **327,** 679–682.

Section S5. Taxonomic distribution of mating systems in Bufonidae

5.1—Taxonomic distribution of mating system in species of Bufonidae.

The distinctions among mating systems are somewhat artificial (see Wells, 2007) and the reproductive mode for many taxa are not well described. Thus we arbitrarily consider prolonged breeders as opposite to species with scramble competition, since we are interested only in the taxonomic distribution of the latter. We consider occurrence of scramble competition when the reproduction is explosive, there is fighting between males for females (but not for the territory) and/or non-choice for pairs. See figure in the last page for the taxonomic distribution of mating systems among the species of Bufonidae included in the phylogenetic analysis of Pyron (2014). Abbreviations: DB, Diego Baldo; LAC, Luis A. Coloma; MOP, Martín O. Pereyra.

Genus (number of species)	Species	Mating system	Source
Adenomus (2 sp.)	*A. kandianus*	scramble	Meegaskumbura *et al*., 2015
Altiphrynoides (2 sp.)	*A. malcolmi*	scramble	Grandison, 1978
Amazophrynella (6 sp.)	*A. minuta*	prolonged	Moreira & Lima, 1996
Anaxyrus (22 sp.)	*A. americanus*	scramble	Wells, 1977
	A. californicus	prolonged	AmphibiaWeb
	A. canorus	scramble	Wright & Wright, 1995
	A. cognatus	scramble	Wells, 1977; Degenhardt *et al*., 1996
	A. debilis	prolonged	Degenhardt *et al*., 1996
	A. exsul	scramble	Wells, 1977
	A. fowleri	prolonged	Wells, 1977; Given, 2002
	A. microscaphus	prolonged	Degenhardt *et al*., 1996
	A. nelsoni	prolonged	Wright & Wright, 1995
	A. punctatus	prolonged	Degenhardt *et al*., 1996
	A. quercicus	prolonged	Wright & Wright, 1995
	A. retiformis	prolonged	Bogert, 1962
	A. terrestris	scramble? explosive	Bartlett & Bartlett, 1999
	A. woodhousii	scramble	Wells, 1977

Ansonia (28 sp.)	*A. malayana*	prolonged	Dring, 1979
Atelopus (96 sp.)	*A. elegans*	scramble	LAC pers. obs.
	A. ignescens	scramble	Peters, 1973
	A. laetissimus	scramble	Granda-Rodriguez *et al*., 2009
	A. oxyrhynchus	cf. scramble	Dole & Duran, 1974
	enex	prolonged	Savage, 2002
	A. varius	scramble? explosive	Crump, 1988
Barbarophryne (1 sp.)	*B. brongersmai*	scramble	Martinez del Marmol Marin & Jimenez Robles, 2013
Blytophryne (1 sp.)	No data		
Bufo (17 sp.)	*B. bankorensis*	prolonged	Pope, 1931
	B. bufo	scramble	Davies & Halliday, 1978; Hoghlund & Robertson, 1988
	B. gargarizans	scramble	Liu, 1950
	B. stejnegeri	prolonged	Fei *et al*., 2009
	B. tibetanus	prolonged	Fei *et al*., 2009
	B. torrenticola	scramble? explosive	Tsuji & Kawamichi, 1996
	B. verrucosissimus	prolonged	Kuzmin, 1999
Bufoides (1 sp.)	No data		
Bufotes (14 sp.)	*B. boulengeri*	explosive	
	B. viridis	prolonged	Salvador, 1996
Capensibufo (2 sp.)	*C. rosei*	scramble	Minter *et al*., 2004, Wells, 1977
Churamiti (1 sp.)	No data		
Dendrophryniscus (10 sp.)	*D. leucomystax*	scramble? explosive	Zina, 2010
Dydinamipus (1 sp.)	No data		
Duttaphrynus (27 sp.)	*D. crocus*	scramble? explosive	Wogan *et al*., 2003
	D. melanostictus	scramble? explosive	Khan, 2000 in AmphibiaWeb
		prolonged	Church, 1960

Epidalea (1 sp.)	*E. calamita*	prolonged	Sinsch, 1992
Frostius (2 sp.)	No data		
Ghathphryne (2 sp.)	No data		
Incilius (40 sp.)	*I. alvarius*	scramble	Blair & Pettus, 1954, Wells, 1977
	I. coniferus	prolonged	Savage, 2002
	I. fastidiosus	scramble	Han & Fu, 2013
	I. luetkenii	prolonged	Savage, 2002
	I. melanochlorus	prolonged	Savage, 2002
	I. nebulifer	prolonged	Savage, 2002
	I. valliceps	prolonged	Wells, 1977
Ingerophrynus (12 sp.)	*I. biporcatus*	prolonged	Márquez & Eekhout, 2006
	I. parvus	prolonged	Shahriza *et al.*, 2012
Laurentophryne (1 sp.)	No data		
Leptophryne (2 sp.)	No data		
Melanophryniscus (26 sp.)	*M. alipioi*	scramble	DB pers. obs.
	M. krauczuki	scramble	DB pers. obs.
	M. langonei	scramble	DB pers. obs.
	M. devincenzii	scramble	DB pers. obs.
	M. cambaraensis	scramble	Santos & Grant, 2010
	M. macrogranulosus	scramble	Zaffaroni Caorsi *et al*., 2014
	M. klappenbachi	scramble	MOP pers. obs.
	M. fulvoguttatus	scramble	DB pers. obs.
	M. atroluteus	scramble	MOP pers. obs.
	M. cupreuscapularis	scramble	DB pers. obs.
	M. rubriventris	scramble	Goldberg *et al.*, 2006
	M. stelzneri	scramble	DB pers. obs.
	M. fulvogutattus	scramble	DB pers. obs.
Mertensophryne (14 sp.)	*M. taitana*	prolonged	Stewart, 1967
Metaphryniscus (1 sp.)	No data		
Nannophryne (4 sp.)	*N. variegata*	scramble	Formas & Pugin 1978
Nectophryne (2 sp.)	No data		

Nectophrynoides (13 sp.)	*N. asperginis*	scramble	Lee *et al.*, 2006
Nimbaphrynoides (1 sp.)	No data		
Oreophrynella (9 sp.)	No data		
Osornophryne (11 sp.)	No data		
Parapelophryne (1 sp.)	No data		
Pedostibes (2 sp.)	No data		
Pelophryne (11 sp.)	No data		
Peltophryne (12 sp.)	*P. fustiger*	prolonged	Schwartz, 1991
	P. peltocephala	prolonged	Valdes, 1988
Phrynoidis (2 sp.)	*P. juxtaspera*	prolonged	Inger & Bacon, 1968
Poyntonophrynus (10 sp.)	*P. damaranus*	scramble	Minter *et al.*, 2004
Pseudobufo (1 sp.)	No data		
Rentapia (3 sp.)	No data		
Rhaebo (13 sp.)	*R. guttatus*	scramble	Machado & Bernarde, 2011
	R. haematiticus	prolonged	Savage, 2002
Rhinella (90 sp.)	*R. arenarum*	scramble	MOP pers. obs.
	R. azarai	scramble	Pereyra *et al.*, 2016
	R. bergi	scramble	Pereyra *et al.*, 2016
	R. castaneotica	prolonged	Caldwell & de Araújo2004
	R. chavin	prolonged	Lehr *et al.*, 2001
	R. icterica	scramble	MOP pers. obs.
	R. fernandezae	scramble	Pereyra *et al.*, 2016
	R. major	scramble	Pereyra *et al.*, 2016
	R. margaritifera	scramble	Wells, 1979, Hodl, 1990
	R. marina	scramble	Wells, 1977
	R. ornata	scramble	DB pers. obs.
	R. ocellata	prolonged	Caldwell & Shepard, 2007
	R. rubescens	scramble	Arantes *et al*., 2015
	R. rumbolli	scramble	DB and MOP pers. obs.
	R. schneideri	scramble	Arantes *et al*., 2015
Sabahphrynus (1 sp.)	No data		
Schismaderma (1 sp.)	*S. carens*	scramble? explosive	Tandy & Keith, 1972

Sclerophrys (44 sp.)	*S. capensis*	prolonged (female choice)	Minter *et al.*, 2004
		scramble	Tandy & Keith, 1972
	S. garmani	prolonged	Keith, 1968; Minter *et al.*, 2004
	S. gutturalis	scramble	Passmore, 1981; du Preez & Carruters, 2009
	S. kisoloensis	prolonged	Keith, 1968
	S. maculata	prolonged	Tandy & Keith, 1972
	S. mauritanica	prolonged	Salvador, 1996
	S. pantherina	scramble	Poynton & Lambiris, 1998
	S. pardalis	scramble	Poynton & Lambiris, 1998
	S. regularis	scramble	Wells, 1977
	S. xeros	prolonged	Tandy *et al.*, 1976
Strauchbufo (1 sp.)	*S. raddei*	scramble	Liu, 1950
Truebella (2 sp.)	No data		
Vandijkophrynus (5 sp.)	*V. angusticeps*	scramble	Minter *et al.*, 2004, du Preez & Carruters, 2009
	V. gariepensis	prolonged	Tandy & Keith, 1972
Werneria (6 sp.)	No data		
Wolterstorffina (3 sp.)	No data		
Xanthophryne (2 sp.)	*X. koynayensis*	scramble	Gaitonde *et al.*, 2016
	X. tigerina	scramble	Gaitonde *et al.*, 2016

5.2—References

AmphibiaWeb (2016). Information on amphibian biology and conservation. [web application]. 2016. Berkeley, California: AmphibiaWeb. Available: http://amphibiaweb.org/. (20 January 2016).

Arantes Í. D. C., Vasconcellos, M. M., Boas, T. C., Veludo, L. B. & Colli, G. R. (2015). Sexual dimorphism, growth, and longevity of two toad species (Anura, Bufonidae) in a Neotropical Savanna. *Copeia* **103,** 329–342.

Bartlett R. D. & Bartlett, P. P. (1999). *A field guide to Texas reptiles & amphibians*. Gulf Pub Co.

Blair W. F. & Peters, D. (1954). The mating call and its significance in the Colorado River toad (*Bufo alvarius* Girard). *The Texas Journal of Science* **6,** 72–77.

Bogert C. M. (1962). Isolation mechanisms in toads of the *Bufo debilis* group in Arizona and western Mexico. *American Museum Novitates* **2100,** 1–37.

Caldwell J. P. & de Araújo, M. C. (2004). Historical and ecological factors influence survivorship in two clades of phytotelm-breeding frogs (Anura: Bufonidae, Dendrobatidae). *Miscellaneous Publications Museum of Zoology University of Michigan* **193,** 11–21.

Caldwell J. P. & Shepard, D. B. (2007). Calling site fidelity and call structure of a neotropical toad, *Rhinella ocellata* (Anura: Bufonidae). *Journal of Herpetology* **41,** 611–621.

Church G. (1960). The invasion of Bali by *Bufo melanostictus*. *Herpetologica* **16,** 15–21.

Crump M. L. (1988). Aggression in harlequin frogs: male-male competition and a possible conflict of interest between the sexes. *Animal Behaviour* **36,** 1064–1077.

Davies N. B. & Halliday, T. R. (1978). Deep croaks and fighting assessment in toads *Bufo bufo*. *Nature* **274,** 683–685.

Degenhardt W. G., Painter, C. W. & Price, A. H. (1996). *Amphibians and reptiles of New Mexico*. University of New Mexico Press, Albuquerque.

Dole J. W. & Durant, P. (1974). Movements and seasonal activity of *Atelopus oxyrhynchus* (Anura: Atelopodidae) in a Venezuelan cloud forest. *Copeia* **1974,** 230–235.

Dring J. C. M. (1979). Amphibians and reptiles from northern Trengganu, Malaysia, with descriptions of two new geckos: *Cnemaspis* and *Cyrtodactylus*. *Bulletin of the British Museum (Natural History) Zoology* **34,** 181–241.

Du Preez L. H. & Carruthers, V. C. (2009). *A complete guide to the frogs of southern Africa*. Struik Nature, Cape Town.

Fei L., Hu, S.-Q., Ye, C.-Y. & Huang, Z.-Z. (2009). *Fauna Sinica. Amphibia. Volume 2 & 3, Anura*. Chinese Academy of Science. Science Press, Beijing.

Formas J.R. & Pugín E. (1978) Tadpoles of *Eupsophus roseus* and *Bufo variegatus* in southern Chile. *Journal of Herpetology* **12,** 243–246.

Gaitonde N., Giri, V., & Kunte, K. (2016). 'On the rocks': reproductive biology of the endemic toad *Xanthophryne* (Anura: Bufonidae) from the Western Ghats, India. *Journal of Natural History* http://dx.doi.org/10.1080/00222933.2016.1200686.

Given M. F. (2002). Interrelationships among calling effort, growth rate and chorus tenure in *Bufo fowleri*. *Copeia* **2002,** 979–987.

Goldberg F. J., Quinzio, S. & Vaira, M. (2006). Oviposition-site selection by the toad *Melanophryniscus rubriventris* in an unpredictable environment in Argentina. *Canadian Journal of Zoology* **84,** 699–705.

Granda H., Del Portillo, A. & Renjifo, J. M. (2008). Uso de hábitat en *Atelopus laetissimus* (Anura: Bufonidae) en una localidad de la Sierra Nevada de Santa Marta, Colombia. *Herpetotropicos* **4,** 87–93.

Grandison A. G. C. (1978). The occurrence of *Nectophrynoides* (Anura Bufonidae) in Ethiopia. A new concept of the genus with a description of a new species. *Monitore Zoologico Italiano* **6,** 119–172.

Han X. & Fu, J. (2013). Does life history shape sexual size dimorphism in anurans? A comparative analysis. *BMC Evolutionary Biology* **13,** 27[1–11].

Hödl W. (1990). Reproductive diversity in Amazonian lowland frogs. In *Fortschrine der Zoologie*, vol. 38 (ed. W. Hanke), pp. 41–60. Gusrav Fischer Verlag, Stuttgart . New York.

Höglund J. & Robertson, J. G. M. (1988). Chorusing behaviour, a density dependent alternative mating strategy on male common toads (*Bufo bufo*). *Ethology* **79,** 324–332.

Inger R. F. & Bacon, J. P. (1968). Annual reproduction and clutch size in rain forest frogs from Sarawak. *Copeia* **1968,** 602–606.

Keith R. (1968). A new species of *Bufo* from Africa, with comments on the toads of the *Bufo regularis* complex. *American Museum Novitates* **2345,** 1–22.

Khan, M. S. (2000) *Duttaphrynus melanostictus*. In: AmphibiaWeb (2016): Information on amphibian biology and conservation. [web application]. 2016. Berkeley, California: AmphibiaWeb. Available: http://amphibiaweb.org/. (20 January 2016).

Kuzmin S. L. (1999). *Amphibians of the Former Soviet Union*. Pensoft series Faunistica, No. 12, Pensoft, Sofia, Moscow.

Lee S., Zippel, K., Ramos, L. & Searle, J. (2006). Captive-breeding programme for the Kihansi spray toad *Nectophrynoides asperginis* at the Wildlife Conservation Society, Bronx, New York. *International Zoo Yearbook* **40,** 241–253.

Lehr E., Köhler, G., Aguilar, C. & Ponce, E. (2001). New species of *Bufo* (Anura: Bufonidae) from Central Peru. *Copeia* **2001,** 216–223.

Liu C.-C. (1950). Amphibians of Western China. *Fieldiana: Zoology Memoirs* **2,** 1–400 + 10 plates.

Machado R. A. & Bernarde, P. S. (2011). Multiple and heterospecific amplexi between the toads *Rhaebo guttatus* and *Rhinella marina* (Anura: Bufonidae). *Herpetology Notes* **4,** 167–169.

Márquez R. & Eekhout, X. R. (2006). Advertisement calls of six species of anurans from Bali, Republic of Indonesia. *Journal of Natural History* **40,** 571–588.

Martinez del Mármol G. & Jiménez Robles, O. (2013). *Barbarophryne brongersmai* (Hoogmoed, 1972) en Marruecos y Sahara Occidental. Disponible en http://www.moroccoherps.com/ficha/Barbarophryne_brongersmai/.

Meegaskumbura M., Senevirathne, G., Wijayathilaka, N., Jayawardena, B., Bandara, C., Manamendra-Arachchi, K. & Pethiyagoda, R. (2015). The Sri Lankan torrent toads (Bufonidae: Adenominae: *Adenomus*): species boundaries assessed using multiple criteria. *Zootaxa* **3911,** 245–261.

Minter L. R., Burger, M., Harrison, J. A., Braack, H. H., Bishop, P. J. & Klopfer, D. (2004). *Atlas and red data book of the frogs of South Africa, Lesotho, and Swaziland*. Smithsonian Institution and the Avian Demography Unit, Washington, DC.

Moreira G. & Lima, A. P. (1991). Seasonal patterns of juvenile recruitment and reproduction in four species of leaf litter frogs in central Amazonia. *Herpetologica* **47,** 295–300.

Passmore N. I. (1981). Sound levels of mating calls of some African frogs. *Herpetologica* **37,** 166–171.

Pereyra M. O., Baldo, D., Blotto, B. L., Iglesias, P. P., Thomé, M. T. C., Haddad, C. F. B., Barrio-Amorós, C., Ibáñez, R. & Faivovich, J. (2016). Phylogenetic relationships of toads of the *Rhinella granulosa* group (Anura: Bufonidae): a molecular perspective with comments on hybridization and introgression. *Cladistics* **32,** 36–53.

Peters J. A. (1973). The frog genus *Atelopus* in Ecuador (Anura: Bufonidae). *Smithsonian Contributions to Zoology* **145,** 1–49.

Pope C. H. (1931). Notes on Amphibians from Fukien, Hainan and other parts of China. *Bulletin of the American Museum of Natural History* **61,** 307–611.

Poynton J. C. & Lambiris, A. J. L. (1998). On *Bufo pantherinus* A. Smith, 1828 (Anura: Bufonidae), the leopard toad of the southwestern Cape, South Africa, with the designation of a neotype. *African Journal of Herpetology* **47,** 3–12.

Pyron R. A. (2014). Biogeographic analysis reveals ancient continental vicariance and recent oceanic dispersal in amphibians. *Systematic Biology* **63,** 779–797

Salvador A. (1996). *Amphibians of nortwest Africa*. Smithsonian Herpetological Information Service, Washington, D. C.

Santos R. R. & Grant, T. (2010). Diel pattern of migration in a poisonous toad from Brazil and the evolution of chemical defenses in diurnal amphibians. *Evolutionary Ecology* **25,** 249–258.

Savage J. M. (2002). *The Amphibians and Reptiles of Costa Rica*. University of Chicago Press, Chicago.

Schwartz A. & Henderson, R. W. (1991). *Amphibians and Reptiles of the West Indies: descriptions, distributions and natural history*. University Press of Florida, Gainesville.

Shahriza S., Ibrahim, J. & Anuar, M. S. S. (2012). Breeding activities of *Ingerophrynus parvus* (Anura: Bufonidae) in Kedah, Malaysia. *Sains Malaysiana* **41,** 1431–1435.

Sinsch U. (1992). Structure and dynamic of a natterjack toad metapopulation (*Bufo calamita*). *Oecologia* **90,** 489–499.

Stewart M. M. (1967). *Amphibians of Malawi*. State University of New York, Albany.

Tandy M. & Keith, R. (1972). *Bufo* of Africa. In *Evolution in the genus Bufo* (ed. W. F. Blair), pp. 119–170. University of Texas Press, Austin.

Tandy M., Tandy, J., Keith, R. & Duff-Mackay, A. (1976). A new species of *Bufo* (Anura: Bufonidae) from Africa's dry savannas. *Texas Memorial Museum (Austin)* **24,** 1–20.

Tsuji H. & Kawamichi, T. (1996). Breeding activity of a stream-breeding toad, *Bufo torrenticola*. *Japanese Journal of Herpetology* **16,** 117–128.

Valdes A. (1988). Systematic comments on *Peltophryne peltocephala* (Anura: Bufonidae) in the cuban archipielago. *Caribbean Journal of Science* **24,** 39–43.

Wells K. D. (1977). The social behaviour of anuran amphibians. *Animal Behaviour* **25,** 666–693.

Wells K. D. (1979). Reproductive behavior and male mating success in a neotropical toad, *Bufo typhonius*. *Biotropica* **11,** 301–307.

Wells K. D. (2007) *The ecology and behaviour of amphibians*. The University of Chicago Press, Chicago.

Wogan G. O. U., Win, H., Thin, T., Lwin, K. S., Shein, A. K., Kyi, S. W. & Tun, H. (2003). A new species of *Bufo* (Anura: Bufonidae) from Myanmar (Burma), and redescription of the little-known species *Bufo stuarti* Smith 1929. *Proceedings of the California Academy of Science* **54,** 141–153.

Wright A. H. & Wright, A. A. (1995). *Handbook of Frogs and Toads of the United States and Canada. Third edition*. Comstock Publishing Associates, Ithaca & London.

Zaffaroni Caorsi V., Colombo, P., Freire, M. D., Amaral, I. B., Zank, C., Borges-Martins, M. & Grant, T. (2014). Natural history, coloration pattern and conservation status of the threatened South Brazilian red bellied toad, *Melanophryniscus macrogranulosus* Braun, 1973 (Anura, Bufonidae). *Herpetology Notes* **7,** 585–598.

Zina J. (2010). Estudo comparativo da taxocenose de anuros de quatro Municípios do Lagamar Paulista, Universidade Estadual Paulista.
